# Neuroimaging markers and disability scales in multiple sclerosis: A systematic review and meta-analysis

**DOI:** 10.1371/journal.pone.0312421

**Published:** 2024-12-05

**Authors:** Omid Mirmosayyeb, Mohammad Yazdan Panah, Yousef Mokary, Mohammad Mohammadi, Elham Moases Ghaffary, Vahid Shaygannejad, Bianca Weinstock-Guttman, Robert Zivadinov, Dejan Jakimovski

**Affiliations:** 1 Department of Neurology, Jacobs Comprehensive MS Treatment and Research Center, Jacobs School of Medicine and Biomedical Sciences, University at Buffalo, State University of New York, Buffalo, NY, United States of America; 2 Isfahan Neurosciences Research Center, Isfahan University of Medical Sciences, Isfahan, Iran; 3 Pharmacy School, University of Missouri-Kansas City, Kansas City, MO, United States of America; 4 Department of Neurology, Buffalo Neuroimaging Analysis Center, Jacobs School of Medicine and Biomedical Sciences, University at Buffalo, State University of New York, Buffalo, NY, United States of America; 5 Center for Biomedical Imaging at the Clinical Translational Science Institute, University at Buffalo, State University of New York, Buffalo, NY, United States of America; King’s College Hospital NHS Trust: King’s College Hospital NHS Foundation Trust, UNITED KINGDOM OF GREAT BRITAIN AND NORTHERN IRELAND

## Abstract

**Background:**

Multiple sclerosis (MS) is a central nervous system disorder marked by progressive neurological impairments. Magnetic resonance imaging (MRI) parameters are key paraclinical measures that play a crucial role in the diagnosis, prognosis, and monitoring of MS-related disability. This study aims to analyze and summarize the existing literature on the correlation between MRI parameters and disability in people with MS (pwMS).

**Methods:**

The PubMed/MEDLINE, Embase, Scopus, and Web of Science databases were searched from inception to July 19, 2024, and a meta-analysis was carried out using R software version 4.4.0 and the random effects model used to determine the pooled correlation coefficient, with its 95% confidence interval (CI), between MRI measurements and disability scales.

**Results:**

Among 5741 studies, 383 studies with 39707 pwMS were included. The meta-analysis demonstrated that Expanded Disability Status Scale (EDSS) had significant correlations with cervical cord volume (r = -0.51, 95% CI: -0.62 to -0.38, I^2^ = 0%, *p*-heterogeneity = 0.86, *p*-value<0.01), cortical lesion volume (r = 0.45, 95% CI: 0.36 to 0.53, I^2^ = 68%, *p*-heterogeneity<0.01, *p*-value<0.01), brain volume (r = -0.40, 95% CI: -0.47 to -0.33, I^2^ = 41%, *p*-heterogeneity = 0.05, *p*-value<0.05), and grey matter volume (GMV) (r = -0.36, 95% CI: -0.49 to -0.21, I^2^ = 0%, *p*-heterogeneity = 0.53, *p*-value<0.01), respectively.

**Conclusion:**

This study offers evidence suggesting that cortical lesion volume, brain volume, GMV, and MRI measurements of the spinal cord may constitute reliable indicators for assessing disability in pwMS.

## 1. Introduction

Multiple sclerosis (MS) is one of the leading causes of non-traumatic disability among young adults [1], affecting approximately 2.8 million people globally in 2024 [2,3]. MS is known to cause remarkable structural changes in the brain and spinal cord because of demyelination and subsequent neuronal damage within the central nervous system (CNS) [4]. People with multiple sclerosis (pwMS) face a spectrum of physical and cognitive impairments, which generally accumulate over time [5].

Expanded Disability Status Scale (EDSS) is widely used to grade disability in MS and spans from 0, or normal neurological examination, to 10, which signifies death caused by MS. To determine EDSS scores, eight functional scales including sensory, cerebellar, bowel, and bladder, visual, brainstem, pyramidal, cerebral are evaluated [6,7]. Disability measurements have been shown to correlate with imaging metrics in MS [8,9].

Likewise, magnetic resonance imaging (MRI) allows for visualizing the pathology of MS [10] and monitoring the progression of the disease [11]. The demand for MRI scans has escalated in recent years as a result of advancements in diagnostic criteria, enabling early and accurate diagnosis, estimating the risk of developing MS, evaluating the progression of the disease, monitoring the safety and efficacy of disease-modifying therapies (DMTs), and make treatment decisions for pwMS [12,13].

Brain MRI plays a vital role in identifying lesions [14] and atrophy [15], which are associated with physical disability [16], cognitive impairment [17], disease progression [18], and response to DMTs [19] in MS. Spinal cord MRI also plays a fundamental role in diagnosing and tracking disease progression of pwMS [20,21].

While MS was previously considered primarily a disease affecting the white matter (WM), recent studies highlight the importance of grey matter (GM) involvement, particularly in cortical lesions and GM atrophy [22]. These MRI parameters are instrumental in detecting, monitoring, and predicting future disability in MS. However, previous studies have shown mixed and controversial results regarding the correlation between MRI findings and EDSS scores [18,23,24].

Alternatively, previous meta-analyses have focused on limited MRI brain regions, limited brain lesions, and only EDSS as a disability assessment [8,25,26]. Additionally, in previous studies, normalized and non-normalized studies were combined in the analysis [8,25,26]. A predominant limitation of prior meta-analyses in this field is the absence of separate assessments of these correlations across distinct subtypes of MS, specifically relapsing-remitting MS (RRMS) vs. progressive MS (PMS) [8,25,26]. On the other hand, previous original studies have demonstrated a wide range of correlations between different brain regions, as well as spinal cord regions, with other disability measurements, rather than EDSS, such as Time-25 Food Walk (T25FW), 9-Hole Peg Test (9HPT), and Multiple Sclerosis Functional Composite (MSFC) [27–30]. Notably, these types of associations between other MRI metrics and disability measurements have not been investigated in a systematic review and meta-analysis so far. This systematic review and meta-analysis aimed to provide a more comprehensive understanding of the relationship between diverse brain and spinal cord MRI parameters and their correlation with disability scores across various assessments in pwMS, ultimately offering a more definitive evaluation of this connection.

## 2. Methods

This comprehensive review and meta-analysis adhered to the guidelines specified by the Preferred Reporting Items for Systematic Reviews and Meta-Analyses (PRISMA) [31].

### 2.1. Search strategies

To conduct a systematic search, we systematically searched MEDLINE (PubMed), Embase, Scopus, and Web of Science from inception to July 19, 2024, to identify all relevant studies. We used the terms syntax and the Medical Subject Headings (MeSH) related to MS, MRI measurements, and disability, as well as the correlation between them (S1 File).

### 2.2. Study design

A literature search and study screening was conducted by two independent reviewers, [MYP] and [EMG], using EndNote software version 20 [32]. Additionally, to identify studies missing from the initial search query, the two reviewers reviewed references from previous reviews and included studies. In cases where the abstract indicated a potential relevance to the review question, additional full-text articles were obtained for examination. When there was disagreement between two reviewers, a third reviewer [OM] was consulted to settle the issue.

### 2.3. Inclusion and exclusion criteria

The inclusion criteria for this study contained English-language, peer-reviewed studies that adopted cross-sectional, case-control, or cohort study designs. The participant pool in these studies comprised patients with a confirmed diagnosis of MS, as defined by the McDonald Criteria from 2001 to 2017. For the studies before 2001, clinical and radiologic findings were described and considered as confirmatory diagnosis of MS. Furthermore, only studies that reported correlation coefficients (either Spearman or Pearson) between MRI parameters of the volume and lesions of brain and spinal cord structures and various disability measurements including EDSS [33], T25FW [34], 9HPT [35,36], MSFC [34,37] Multiple Sclerosis Severity Score (MSSS) [38], and Functional Somatic Symptoms (FSS) [39], were included.

The exclusion criteria specifically targeted studies that did not meet the methodological rigor or relevance required for this analysis. We excluded non-English publications, case reports, case series, and gray literature, i.e., conference abstracts, letters, editorials, protocols, correspondences, and review articles. Moreover, in-vivo, in-vitro and animal studies were omitted. This methodological approach ensured a comprehensive and relevant analysis of the correlation between MRI features and disability in MS patients.

### 2.4. Data extraction

The desired data of articles was independently extracted by [MYP] and [YM] and then verified by [DJ] in S2 File. Data was extracted from the relevant studies, including the first author, country, publication year, study design, baseline demographic characteristics of pwMS, MS type, EDSS [33], disease duration, MRI device and type, disability measurement method, key findings of the correlation coefficient between MRI parameters and disability measurement, and the risk of bias assessment score.

### 2.5. Risk of bias assessment

The assessment of the methodological quality of the included studies was conducted independently by [MYP] and [MM], utilizing the Newcastle-Ottawa Scale (NOS) for evaluation [40]. The baseline data from patients was used; consequently, the cross-sectional checklist was employed for the quality assessment. The studies were evaluated for risk of bias in three areas: selection of the studies, comparability, and outcome. NOS scores of 0 to 4 indicate low-quality, 5 to 6 moderate-quality, and 7 to 10 high-quality studies. Additionally, any disagreements were resolved by a third researcher [OM].

### 2.6. Data analysis

Our systematic review precisely calculated the aggregated associations between MRI indicators and methods measuring disability in MS, given that a minimum of five studies reported on the outcome. Initially, we converted all correlations into z-scores and employed the inverse variance approach to determine the overall effect size. These findings were then transformed back to their original state for presentation. We also conducted subgroup analyses based on MS type (RRMS vs. PMS vs. mixed). It is essential to note that PMS encompassed all progressive MS types, including primary progressive MS (PPMS) and secondary progressive MS (SPMS), while the mixed group comprised studies that did not separately report MS-type data.

All analytical procedures were carried out using R statistical software, version 4.4.0. Additionally, a sensitivity analysis was conducted for analyses involving more than ten studies to identify any outliers. We performed Egger’s and Begg’s tests for analyses with over ten studies to assess potential publication bias; the funnel plots for these analyses were created additionally. Considering the potential heterogeneity across studies, a random-effects model was applied to all the studies.

## 3. Result

### 3.1. Study selection

**Fig 1:**
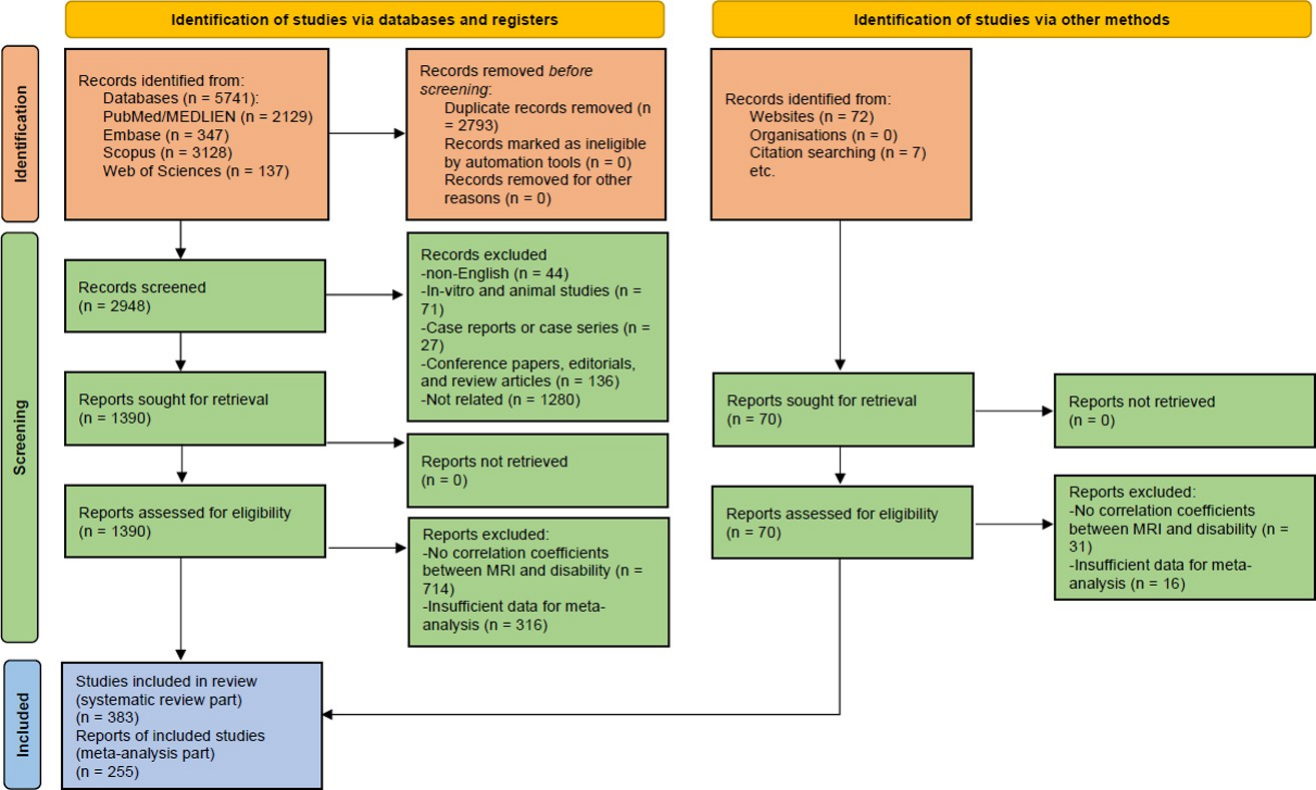
PRISMA 2020 flow diagram of searching databases and study selection process.

The comprehensive literature search identified 5741 articles. However, after eliminating duplicate studies, we were left with 2948 unique articles subjected to further screening based on their titles and abstracts. The initial screening process identified 1390 studies as meeting the criteria for eligibility. Data for 1007 of the articles were found to be insufficient, leading to their disqualification. After conducting a full-text review, 383 and 255 studies were eligible for inclusion in our qualitative and quantitative synthesis (Fig 1).

### 3.2. Study characteristics

The studies included in the review provided data on 39707 pwMS, with a female-to-male ratio of 2.1:1. The mean ± standard deviation (SD) age, disease duration, and EDSS of pwMS were 42.2 ± 4.7 years, 10.1 ± 6.6 years, and 3.1 ± 1.4, respectively. The included studies comprised 169 cross-sectional studies, 133 case-control studies, and 81 cohort studies. S2 File provides a summary of the included studies. Additionally, the results of meta-analyses are shown in S3 File.

### 3.3. Correlations between brain lesions and disability

**Fig 2:**
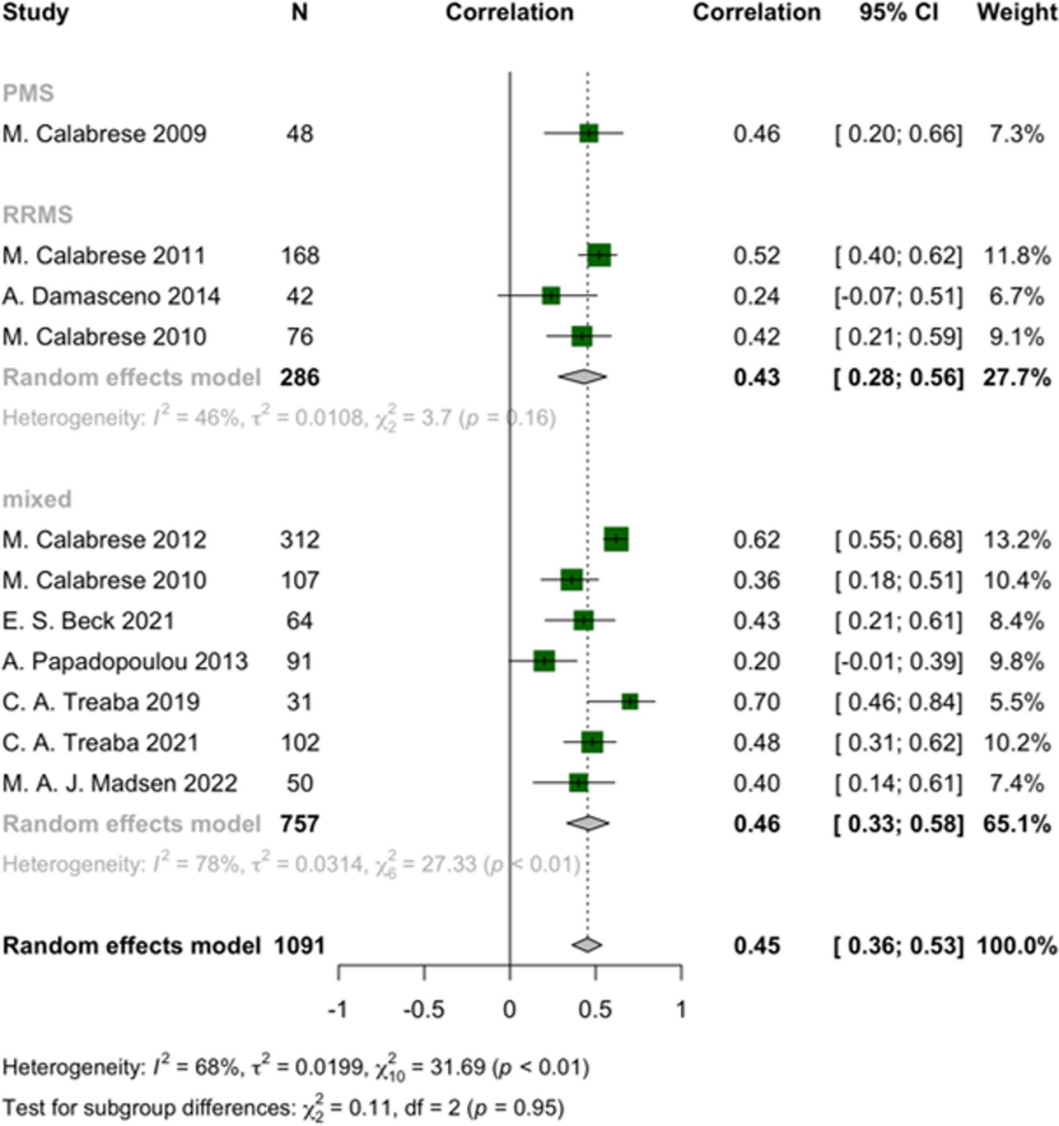
Forest plot of EDSS and cortical lesion volume correlation in pwMS.

In descending order of effect size, pooled correlations indicated that EDSS had a significant direct correlation with cortical lesion volume (r = 0.45, 95% CI: 0.36 to 0.53, I^2^ = 68%, *p*-heterogeneity < 0.01, z-score = 8.82, *p*-value < 0.01), WM lesion volume (r = 0.43, 95% CI: 0.34 to 0.51, I^2^ = 67%, *p*-heterogeneity < 0.01, z-score = 8.65, *p*-value < 0.01), T1 lesion count (r = 0.39, 95% CI: 0.27 to 0.49, I^2^ = 38%, *p*-heterogeneity = 0.14, z-score = 6.3, *p*-value < 0.01), cortical lesion count (r = 0.37, 95% CI: 0.24 to 0.48, I^2^ = 73%, *p*-heterogeneity < 0.01, z-score = 5.38, *p*-value < 0.01), and T1 lesion volume (T1LV) (r = 0.35, 95% CI: 0.31 to 0.40, I^2^ = 56%, *p*-heterogeneity < 0.01, z-score = 13.91, *p*-value < 0.01), respectively (Fig 2 and S4 File). The subgroup analysis of lesion volumes and their correlation with EDSS scores revealed distinct patterns between RRMS and PMS. In RRMS, brain lesion volume showed a strong correlation with EDSS (r = 0.39, 95% CI: 0.33 to 0.44, z = 12.05, *p*-value < 0.01), indicating a significant relationship between brain lesions and disability. For T1 lesion volume, the correlation remained strong in RRMS (r = 0.35, 95% CI: 0.27 to 0.43, z = 7.76, *p*-value < 0.01). T2 lesion volume also demonstrated a significant correlation in RRMS (r = 0.30, 95% CI: 0.25 to 0.35, z = 11.06, *p*-value < 0.01). In contrast, PMS showed minimal or non-significant correlations across all lesion types: brain lesion volume (r = 0.08, 95% CI: -0.09 to 0.25, z = 0.94, *p*-value = 0.35), T1 lesion volume (r = 0.10, 95% CI: -0.24 to 0.42, z = 0.59, *p*-value = 0.56), and T2 lesion volume (r = 0.17, 95% CI: -0.03 to 0.36, z = 1.65, *p*-value = 0.10). The robustness of our findings is further validated by sensitivity analyses, which revealed no outliers, and by assessments of publication bias, which showed no evidence of such bias (S5 and S6 Files).

In addition to pooled correlations of EDSS, T2 lesion volume (T2LV) also displayed significant relationships with MSFC (r = -0.33, 95% CI: -0.39 to 0.-0.26, I^2^ = 0%, *p*-heterogeneity = 0.7, z-score = -9.38, *p*-value < 0.01) and 9HPT (r = 0.30, 95% CI: 0.19 to 0.40, I^2^ = 77%, *p*-heterogeneity < 0.01, z-score = 4.81, *p*-value < 0.01) in pwMS, respectively (S4 File). The subgroup analysis of T2 lesion volume and its correlation with T25FW in MS subtypes revealed significant differences between RRMS and PMS. In RRMS, T2 lesion volume showed a moderate and significant correlation with T25FW (r = 0.26, 95% CI: 0.15 to 0.36, z = 4.72, *p*-value < 0.01), indicating that higher lesion burden is associated with worse walking speed in this subtype. Conversely, in PMS, the correlation was minimal and not statistically significant (r = 0.03, 95% CI: -0.36 to 0.41, z = 0.13, *p*-value = 0.9), suggesting that T2 lesion volume does not closely relate to walking disability in PMS. The robustness of our findings is further validated by sensitivity analyses, which revealed no outliers, and by assessments of publication bias, which showed no evidence of such bias. (S5 and S6 Files)

### 3.4. Correlations between brain volume structures and disability

**Fig 3:**
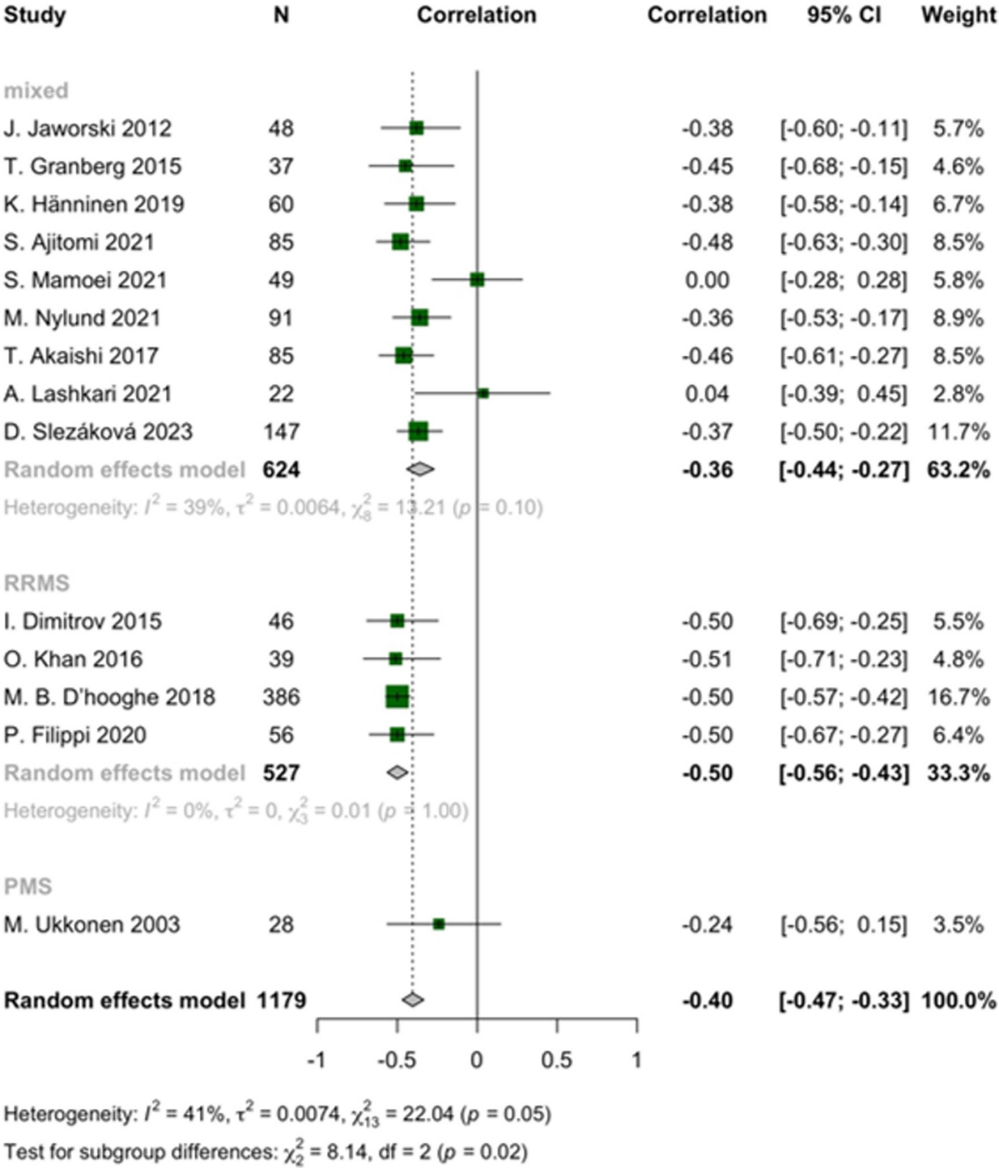
Forest plot of EDSS and brain volume correlation in pwMS.

In descending order of effect size, pooled correlations demonstrated that greater EDSS was negatively correlated with brain volume (r = -0.40, 95% CI: -0.47 to -0.33, I^2^ = 41%, *p*-heterogeneity = 0.05, z-score = -10.46, *p*-value < 0.01), brain parenchymal fraction (BPF) (r = -0.37, 95% CI: -0.41 to -0.32, I^2^ = 54%, *p*-heterogeneity < 0.01, z-score = -13.95, *p*-value < 0.01), and grey matter volume (GMV) (r = -0.36, 95% CI: -0.49 to -0.21, I^2^ = 0%, *p*-heterogeneity = 0.53, z-score = -11.18, *p*-value < 0.01), respectively. Conversely, EDSS exhibited a significant direct relationship with third ventricular width (3VW) (r = 0.35, 95% CI: 0.29 to 0.40, I^2^ = 0%, *p*-heterogeneity = 0.85, z-score = 10.97, *p*-value < 0.01) in pwMS, respectively (Fig 3 and S4 File). The strength of our results is additionally supported by sensitivity analyses, which identified no outliers, and by evaluations of publication bias, which indicated an absence of such bias (S5 and S6 Files).

Among other measures of disability of pwMS, pooled correlations showed that 9HPT had a negative significant association with normalized brain volume (r = -0.32, 95% CI: -0.45 to -0.18, I^2^ = 77%, *p*-heterogeneity < 0.01, z-score = -4.45, *p*-value < 0.01). Furthermore, a negative significant relationship was observed between T25FW and grey matter fraction (GMF) (r = -0.32, 95% CI: -0.43 to -0.20, I^2^ = 20%, *p*-heterogeneity = 0.29, z-score = -4.97, *p*-value < 0.01) (S4 File). The strength of our results is additionally supported by sensitivity analyses, which identified no outliers, and by evaluations of publication bias, which indicated an absence of such bias (S5 and S6 Files).

### 3.5. Correlations between spinal cord MRI measurements and disability

**Fig 4:**
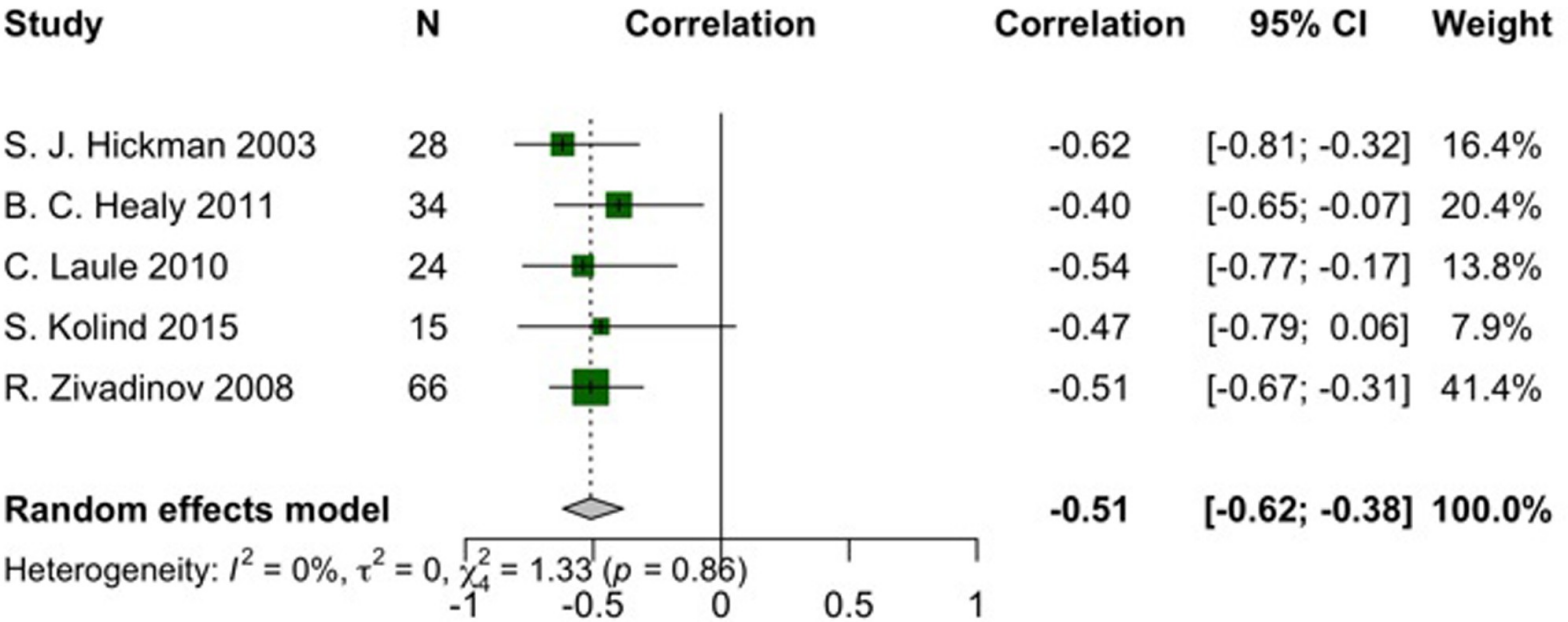
Forest plot of EDSS and cervical cord volume correlation in pwMS.

**Fig 5:**
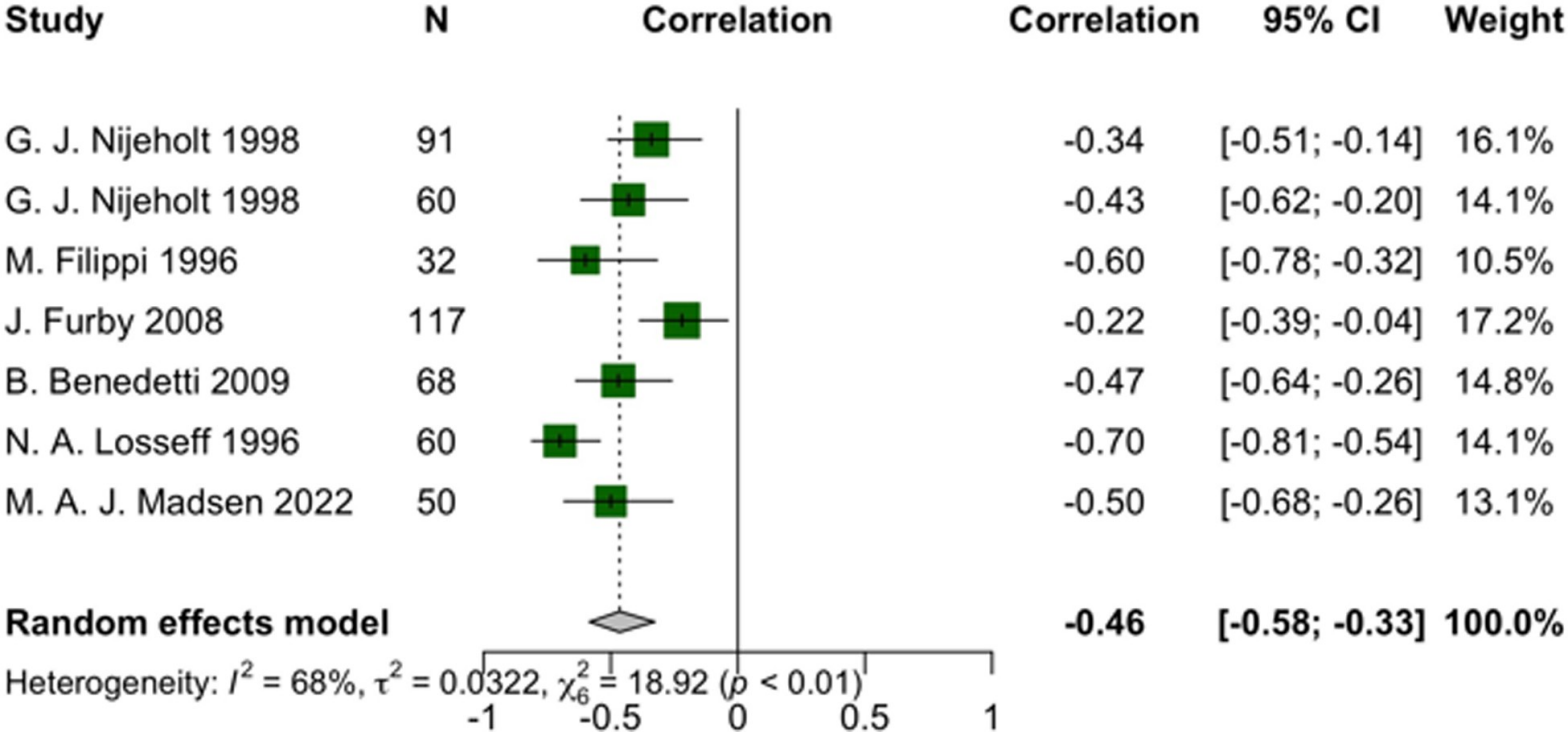
Forest plot of EDSS and spinal cord cross-sectional area correlation in pwMS.

In descending order of effect size, among pooled correlations, the EDSS indicated a significant negative relationship with cervical cord volume (r = -0.51, 95% CI: -0.62 to -0.38, I^2^ = 0%, *p*-heterogeneity = 0.86, z-score = -6.94, *p*-value < 0.01) and spinal cord cross-sectional area (SCCA) (r = -0.46, 95% CI: -0.58 to -0.33, I^2^ = 68%, *p*-heterogeneity < 0.01, z-score = -6, *p*-value < 0.01), respectively (Figs 4 and 5 and S4 File). The reliability of our findings is further substantiated by sensitivity analyses that detected no outliers, alongside publication bias evaluations, which confirmed the lack of bias (S5 and S6 Files).

Detailed results of meta-analyses on correlation coefficients between different kinds of disability and various MRI findings, as well as subgroup analysis based on MS type (RRMS or PMS), are presented in S3 and S4 Files. The reliability of our findings is further substantiated by sensitivity analyses that detected no outliers, alongside publication bias evaluations, which confirmed the lack of bias (S5 and S6 Files).

### 3.6. Sensitivity analysis

We have conducted a Leave-one-out analysis for all analyses with more than ten studies. The results showed that no study significantly influenced our findings in all analyses (S5 File).

### 3.7. Publication bias

We performed Egger’s and Begg’s tests for analyses with more than ten studies to assess the probable publication bias. The results showed no evidence of publication bias in the majority of the meta-analyses. Details of the results of Egger’s and Begg’s tests are demonstrated in S6 File. A symmetrical distribution was found in the funnel plots of most analyses, indicating that publication bias was not present. Details are available in S7 File.

### 3.8. Risk of bias assessment

According to the findings of this review, the risk of bias for all studies was found to be moderate, with a mean and SD score of 6.3 ± 1.2. The results of the quality assessment were summarized in S2 File. Despite the lack of evidence indicating a direct relationship between a moderate risk of bias and the invalidity of research findings, it suggests certain limitations.

## 4. Discussion

The findings of this comprehensive meta-analysis revealed considerable correlations between MRI measures, including brain lesion volume, brain structure volume, and spinal cord evaluations, and the degree of disability, using a variety of measurements, in pwMS. These results suggest that MRI parameters may potentially provide a reliable marker for monitoring disease progression and forecasting the clinical outcome in MS. Also, this integration of imaging measures into routine practice may increase the accuracy of assessments of disability and improve strategies for patient management.

### 4.1. Association between brain lesions and disability in MS

The current study identifies cortical lesions as having a significant correlation with disability in MS. In this review, we explored the overall relationships between cortical lesion volume, the number of cortical lesions, and disability. Our results suggests that cortical lesion volume may serve as a more effective indicator of disability in pwMS. These findings align with a recent systematic review [8], although some of other reviews did not evaluate these specified MRI measurements [25,41]. Another systematic review and meta-analysis demonstrated that the risk of disability progression in pwMS significantly increases when the lesion count exceeds ten [26]. These lesions, often caused by inflammatory processes in the cerebral sulci, have been consistently linked to cumulative neurological disability [42–45]. Despite some previous studies reporting insignificant associations, the majority of the evidence supports the role of cortical pathology as a critical factor in disability progression [46–48]. Beck et al. showed that all subtypes of cortical lesions were more prevalent in PMS [49]. It was shown that a considerable number of patients with PPMS exhibited cortical lesions [50]. Several studies have proposed that cortical lesion formation persists during the progressive stages of MS [45,51].

Meanwhile, Calabrese et al. [42] believed that cortical pathology appears to exhibit an independent relationship with the clinical phenotypes of MS. The difference in cortical lesion load at baseline can be explained by the varying durations of disease [42]. The predictive value of cortical lesions on the subsequent development of irreversible tissue damage in the GM and the accumulation of fixed clinical disability has been established [43]. Cortical involvement has the potential to serve as an extraneous and independent measure for tracking the progression and monitoring the evolution of disease [52].

Furthermore, our study revealed that WMLV was an MRI parameter strongly associated with disability in MS. This finding is contrast with recent systematic review and meta-analysis [8]. This may be attributed to the inclusion of a larger number of studies and patients in our review, which demonstrates a significant and stronger overall correlation. WMLV plays a vital role in the cortical atrophy development process [53]. The differences in cortical thinning observed during the disease progression appear to be linked with the WMLV [54]. Previous research has indicated that in the early stages of MS, disability is primarily influenced by subcortical WM lesions, whereas in the more advanced stages, disability is primarily determined by the extent of cortical pathology [55–57]. The progression of WM and cortical lesions in MS is believed to occur independently of each other [45]. During the course of MS, both white and GM abnormalities were observed to worsen, but the relative dominance of these pathological processes seemed to change. In the early relapsing phases of the disease, the increase in focal WMLV was more prominent; however, in secondary progression, the total GMV became the primary focus of deterioration [55,58]. Numerous research investigations have demonstrated a notable positive association between WMLV and EDSS scores in individuals with RRMS [55,59,60]. In patients with RRMS, WM lesions are believed to result primarily from adaptive immunity responses, such as the entry of lymphocytes into the CNS [61].

Moreover, disability in MS exhibited significant relationships with the number and T1 lesion volume. There is agreement between our findings and those of previous systematic reviews regarding T1 lesion volume [8,25]. MRI and histopathological correlative studies have demonstrated that MS lesions that exhibit hypointensity on T1-weighted images, commonly known as "black holes," represent the more severely damaged areas within the overall MS-related lesion burden in the brain [62]. As the role of neurodegeneration in the pathophysiology of MS has gained prominence, the formation of chronic or persistent Tl-hypointense lesions (black holes) has been used as markers of axonal loss and neuronal destruction to measure disease activity [63]. Previous studies revealed that the presence of T1-weighted hypointense lesions is strongly indicative of subsequent MS confirmation [64]. Consistent with our research, a systematic review and meta-analysis revealed weak to moderate correlations between T1 hypointense lesion volume and EDSS [25]. Several strategies have been proposed to enhance black hole detection and strengthen the association with disability, such as the restriction of black hole measurements according to their intensity or relaxation time thresholds [65].

### 4.2. Correlation between brain volume structures and disability in MS

This systematic review and meta-analysis represented the first exploration of the correlations between brain volume and normalized brain volume with disability, thereby facilitating a more precise estimation of the overall correlation that prior meta-analyses failed to consider, which reported a non-significant association [8]. In contrast, our findings indicated that both brain volume and normalized brain volume demonstrate significant correlations with disability when analyzed separately. Brain volume loss in pwMS is not merely a consequence of aging but also a result of neurodegeneration attributable to the disease [15]. Studies have shown a strong correlation between brain atrophy and clinical disease progression, which may serve as an important predictor of EDSS worsening [15,66–68]. In the clinical management of pwMS, routine brain volume measures have proven to be highly valuable in the early assessment of treatment responses and in predicting disease evolution [69]. Contrary to our findings, in a recent meta-analysis, a non-significant association between brain volume and EDSS scores may be due to the fact that normalized brain volumes were not considered. In isolation, our study revealed a significant association between normalized brain volumes and EDSS [70].

GMV was one of the MRI measures of brain volume structures with an approximately high magnitude association with disability in our study. This finding aligns with previous meta-analyses [8]; however, our study incorporates a greater number of studies, resulting in stronger correlations for both GMV and normalized GMV. Previous studies have shown a highly significant correlation between GMV and BPV with EDSS [71]. In advanced stages of MS, the most significant correlations are observed, and cortical atrophy begins prior to the noticeable emergence of clinical symptoms [71,72]. A higher EDSS score has been linked to an increased extent of atrophy [73,74]. According to current research, grey matter atrophy is responsible for the loss of brain volume rather than WM atrophy [15]. The occurrence of GM atrophy in pwMS, which in conjunction with other atrophic brain regions, may indicate a combination of processes including demyelination, neurite transaction, and reduced synapse or glial densities [71]. The loss of GMV is a more reliable indicator of disability progression, especially in patients with PMS, compared to changes in WM [75].

It was found that in pwMS, BPF is generally associated with a greater extent of brain atrophy than in controls [76]. Brain atrophy and the development of secondary progressive disease are influenced by various factors, including not only destructive WM lesions in the brain but also GM factors such as cortical lesions, innate immunity, meningeal pathology, and neurodegeneration [77,78]. A five-year cohort study by Bakhshi et al. demonstrated a correlation between baseline BPF and the subsequent 5-year change in disability in MS [77]. According to Bakhshi et al., the validity of BPF is higher than that of GMF in comparison. Due to the more common segmentation errors and variability associated with measuring GMF, BPF may be more reliable technical compared to GMF [79].

The current review expands upon previous systematic reviews and meta-analyses that primarily concentrated on volumetric MRI [8,25,41] by additionally evaluating the third ventricle, which may serve as a marker of disability in pwMS. Many studies showed that the 3VW, as a sign of brain atrophy, could potentially serve as the most valuable indicator for evaluating the physical disability, whole-brain volume, and GMV in MS [80]. The widening of the third ventricle may result from atrophy of adjacent structures, particularly the thalamus. It can potentially lead to diverse neurological symptoms [81,82]. Neurodegenerative processes are a significant factor contributing to the accumulation of long-term disability in MS [83–85]. 3VW may be a more reliable measure of neurodegeneration in MS [86]. Although several approaches have been developed for monitoring brain atrophy in pwMS, these techniques have not yet been integrated into clinical practice [81].

This meta-analysis filled the gap of prior systematic reviews and meta-analyses [8,25,41] by separately estimating the correlation between MRI measurements and disability in PMS and RRMS, thereby elucidating the differences in these correlations across MS phenotypes. Brain volume analysis and its relationship with disability demonstrated a clear difference between RRMS and PMS, indicating different underlying disease mechanisms. In RRMS, brain atrophy appears more closely related to disability, reflecting active neurodegeneration driven by inflammation that considerably influences disease progression. By comparison, the association of brain volume loss with a disability is weaker in PMS, suggesting that other factors, like spinal cord involvement, may be more important. These findings underscore the need for appropriate treatment strategies, targeting early brain atrophy in RRMS, while a broad strategy to address the complex neurodegeneration should be more effective in PMS.

### 4.3. Relationships between spinal cord MRI measurements and disability in MS

This review represents the first meta-analysis of various MRI measurements of the spinal cord in relation to disability in MS. Previous studies have shown that there is a robust correlation between cervical cord volume (CCV) and disability in MS, which underscores the importance of CCV as a predictor of disability in this debilitating condition [27,87,88]. The volume of the cervical spinal cord offers insights into Wallerian degeneration, the swelling of neurons, and the atrophy process. Progressive demyelination was found to contribute to the development of cervical cord abnormalities in PPMS [89]. Zivadinov et al. [90] found that cervical cord atrophy measurement offers valuable insights into disability that cannot be obtained from brain MRI metrics. Measures at the pontine level in brain MRI may be an effective approach for evaluating upper cervical cord atrophy when only brain images are available [91]. Additionally, medulla oblongata volume may serve as a promising biomarker for assessing spinal cord damage in MS [92]. From the evidence presented, it appears that brain stem measurements in brain MRI can serve as a viable alternative to spinal cord MRI in assessing disability in MS.

Strong evidence supports the role of the SCCA in forecasting disability progression in MS [93–95]. According to Benedetti et al., the assessment of intrinsic cord pathology is a valuable method to enhance the comprehension of the mechanisms accountable for the development of fixed disability in this condition [96]. Previous studies have demonstrated a robust association between alterations in the SCCA and disability progression over a mid-to-long-term period [97,98]. SCCA, as an independent predictor of clinical impairment, holds significant value as an integral component of a comprehensive MRI measure for neurodegeneration [99].

## 5. Strengths and limitations

The limitations intrinsic to meta-analysis techniques made it impossible to incorporate cohort or longitudinal studies into the combined correlation coefficient. Therefore, in our meta-analysis, all studies were treated as cross-sectional, utilizing only baseline data. The insights from cohort studies play a vital role in understanding the ongoing association between MRI parameters and disability progression over time. A survey by D’hooghe et al. revealed that brain volumetric measurements appeared to be a promising tool for correlation between EDSS in pwMS [100]. Calabrese et al. demonstrated that baseline cortical lesion volume was correlated with EDSS change in patients with RRMS and SPMS [43]. According to Fisniku et al., the association between GM atrophy and long-term disability in pwMS patients was observed [101]. Cervical cord atrophy has been shown to correlate with EDSS progression and can be a predictor of disability [102]. These studies have shown that structural and functional changes in the brain and spinal cord can affect disability changes of pwMS during follow-up [30,86,103–105]. Future meta-analyses are required to pool longitudinal studies results and elucidate the possible relationships between MRI measures and disability progression in MS.

This study possesses strengths and limitations. This systematic literature review and meta-analysis was conducted by incorporating various factors and involving a large number of studies on pwMS. Most of the conventional MRI measurements of the brain were incorporated into our study. We also incorporated various MRI measurements of the spinal cord that have not yet been systematically reviewed or analyzed in a meta-analysis and these correlations were identified the most remarkable magnitude correlation with disability in MS. Additionally, various types of disabilities associated with MS, including EDSS, T25FW, 9HPT, and MSFC, were included in this review. Furthermore, when sufficient data were available, these correlations were aggregated separately for people with RRMS and PMS, by conducting subgroup analyses, to identify any differences in the correlations between these groups. We also considered normalized volumes of MRI measurements in the analyses since intracranial volume can be a covariate in volumetric procedures. Although previous systematic reviews and meta-analyses [8,25,26,41] have investigated the correlation between MRI indices and EDSS in pwMS, this is the first comprehensive systematic review and meta-analysis to assess the relationship between different measures of disability and different MRI parameters in pwMS. A recent systematic review and meta-analysis [8] was done to survey the association between MRI features and disability in MS. Still, the number of included studies, MRI parameters, and measures of disabilities were limited compared to our study.

On the other hand, the absence of standardization and utilization of various regression models (such as linear, generalized linear, and generalized estimating equations) hindered the pooling of data from various studies to calculate a single correlation coefficient. Furthermore, discrepancies were observed in the reporting patterns across studies, with some studies providing adjusted beta coefficients while others utilized direct or partial correlation coefficients. In some studies, important covariates such as age, gender, disease duration, treatments, and first EDSS were not adjusted for, while in others, they were. Thus, we employed a maximally adjusted correlation of included studies to derive the most accurate pooled correlation. Furthermore, there were disparities in MRI protocols, lesion segmentation techniques, and study designs that could not be taken into account during the analyses. The included studies used different MRI types (mostly 1.5T and 3T), which may affect their findings. Nevertheless, scanners of varying strengths can be utilized interchangeably without considerable differences [106–108]. The findings of our research were fairly consistent despite the diversity of the literature. Future longitudinal studies are warranted to investigate the role of MRI measurements, particularly spinal cord metrics, in predicting disability and its progression. Additionally, more research is needed on the association between advanced MRI methods, such as functional MRI (fMRI) and diffusion tensor imaging (DTI), in MS.

## 6. Conclusion

The findings of our comprehensive systematic review and meta-analysis revealed consistent and significant relationships between MRI measurements of the brain and spinal cord and disability in MS. Furthermore, this study provides valuable insights that cortical lesion volume, brain volume, GMV, and MRI measurements of the spinal cord may be the accurate indicators of underlying pathology correlated with the disability in pwMS. Practitioners could potentially employ these MRI metrics of brain and spinal cord measurements for monitoring and assessing the disability of pwMS and the efficacy of treatments.

## Supporting information

S1 Checklist PRISMA 2020 checklist for a systematic review and meta-analysis. (DOCX)

S1 File Customized search syntax we used in each database. (DOCX)

S2 File Main characteristics of the included studies. (DOCX)

S3 File Results of meta-analyses of disability and MRI measurements correlation in pwMS. (DOCX)

S4 File Forest plots of disability and MRI measurements correlation in pwMS. (DOCX)

S5 File Sensitivity analyses of disability and MRI measurements correlation in pwMS. (DOCX)

S6 File Results of publication bias within different meta-analysis. (DOCX)

S7 File Funnel plots of disability and MRI measurements correlation in pwMS. (DOCX)

S8 File Results of databases searching. (XLSX)
