# Supplementary material for: Neuroimaging markers and disability scales in multiple sclerosis: A systematic review and meta-analysis
Source: PLoS One. 2024 Dec 5;19(12):e0312421. doi: 10.1371/journal.pone.0312421 (PMC11620670; doi:10.1371/journal.pone.0312421)
Supplement: S1 File — (DOCX) [file pone.0312421.s002.docx]

Supplementary 1. Customized syntax we used in each database.

| N | DATA BANK |
| --- | --- |
|  | PUBMED |
| 1. | ((Sclerosis[tiab] AND multiple[tiab]) OR (sclerosis[tiab] AND disseminated[tiab]) OR "disseminated sclerosis"[tiab] OR "multiple sclerosis"[tiab] OR "acute fulminating"[tiab]) AND ((Imaging[tiab] AND "Magnetic Resonance" [tiab]) OR "NMR Imaging"[tiab] OR (Imaging[tiab] AND NMR[tiab]) OR "MR Tomography"[tiab] OR "NMR Tomography"[tiab] OR (Tomography[tiab] AND NMR[tiab]) OR (Tomography[tiab] AND MR[tiab]) OR "Magnetic Resonance Image"[tiab] OR (Image[tiab] AND "Magnetic Resonance" [tiab]) OR "Magnetic Resonance Images"[tiab] OR ("Resonance Image"[tiab] AND Magnetic[tiab]) OR "Magnetization Transfer Contrast Imaging"[tiab] OR "MRI Scans"[tiab] OR "MRI Scan"[tiab] OR (Scan[tiab] AND MRI[tiab]) OR (Scans[tiab] AND MRI[tiab]) OR "MR imaging"[tiab] OR "corpus callosum"[tiab] OR "third ventricle"[tiab] OR thalamus[tiab] OR "gray matter"[tiab] OR "grey matter"[tiab] OR "white matter"[tiab] OR "caudate"[tiab] OR "temporal lobe"[tiab] OR "globus pallidus"[tiab] OR cerebellum[tiab] OR "Basal ganglia" OR "neocortical atrophy"[tiab] OR cortex[tiab] OR "cortical atrophy"[tiab] OR "brain volume"[tiab] OR amygdala[tiab] OR "brain parenchymal fraction"[tiab] OR "brain lesion volume"[tiab] OR "brain lesion load"[tiab] OR "brain stem"[tiab] OR "cerebellar volume"[tiab] OR "cortical volume"[tiab] OR "hippocampus"[tiab] OR insula[tiab] OR neocortex[tiab] OR "nucleus accumbens"[tiab] OR putamen[tiab] OR "T1-lesion"[tiab] OR "T2-lesion"[tiab] OR "temporal pole"[tiab] OR atrophy) AND ("Expanded Disability Status Scale"[tiab] OR "Patient determined disease steps"[tiab] OR disability[tiab] OR "disease progression"[tiab] OR "Confirmed disability progression"[tiab] OR "12-Item MS walking scale"[tiab] OR "12 Item MS walking scale"[tiab] OR "twelve Item MS walking scale"[tiab] OR "2-minute walk test"[tiab] OR "2 minute walk test"[tiab] OR "two minute walk test"[tiab] OR "6-minute walk test"[tiab] OR "6 minute walk test"[tiab] OR "six minute walk test"[tiab] OR "Timed Up and GO"[tiab] OR "Dynamic gait index"[tiab] OR "physical ponent summery"[tiab] OR "Six spot step test"[tiab] OR "Timed 25-Foot Walk"[tiab] OR "Timed 25 Foot Walk"[tiab] OR "Nine-Hole Peg Test"[tiab] OR "Nine Hole Peg Test"[tiab] OR "9-Hole Peg Test"[tiab] OR "9 Hole Peg Test"[tiab]) |
|  | EMBASE |
| 2. | ((Sclerosis:ti,ab AND multiple:ti,ab) OR (sclerosis:ti,ab AND disseminated:ti,ab) OR 'disseminated sclerosis':ti,ab OR 'multiple sclerosis':ti,ab OR 'acute fulminating':ti,ab) AND ((Imaging:ti,ab AND 'Magnetic Resonance':ti,ab) OR 'NMR Imaging':ti,ab OR (Imaging:ti,ab AND NMR:ti,ab) OR 'MR Tomography':ti,ab OR 'NMR Tomography':ti,ab OR (Tomography:ti,ab AND NMR:ti,ab) OR (Tomography:ti,ab AND MR:ti,ab) OR 'Magnetic Resonance Image':ti,ab OR (Image:ti,ab AND 'Magnetic Resonance':ti,ab) OR 'Magnetic Resonance Images':ti,ab OR ('Resonance Image':ti,ab AND Magnetic:ti,ab) OR 'Magnetization Transfer Contrast Imaging':ti,ab OR 'MRI Scans':ti,ab OR 'MRI Scan':ti,ab OR (Scan:ti,ab AND MRI:ti,ab) OR (Scans:ti,ab AND MRI:ti,ab) OR 'MR imaging':ti,ab OR 'corpus callosum':ti,ab OR 'third ventricle':ti,ab OR thalamus:ti,ab OR 'gray matter':ti,ab OR 'grey matter':ti,ab OR 'white matter':ti,ab OR 'caudate':ti,ab OR 'temporal lobe':ti,ab OR 'globus pallidus':ti,ab OR cerebellum:ti,ab OR 'Basal ganglia':ti,ab OR 'neocortical atrophy':ti,ab OR cortex:ti,ab OR 'cortical atrophy':ti,ab OR 'brain volume':ti,ab OR amygdala:ti,ab OR 'brain parenchymal fraction':ti,ab OR 'brain lesion volume':ti,ab OR 'brain lesion load':ti,ab OR 'brain stem':ti,ab OR 'cerebellar volume':ti,ab OR 'cortical volume':ti,ab OR 'hippocampus':ti,ab OR insula:ti,ab OR neocortex:ti,ab OR 'nucleus accumbens':ti,ab OR putamen:ti,ab OR 'T1-lesion':ti,ab OR 'T2-lesion':ti,ab OR 'temporal pole':ti,ab OR atrophy:ti,ab) AND ('Expanded Disability Status Scale':ti,ab OR 'Patient determined disease steps':ti,ab OR disability:ti,ab OR 'disease progression':ti,ab OR 'Confirmed disability progression':ti,ab OR '12-Item MS walking scale':ti,ab OR '12 Item MS walking scale':ti,ab OR 'twelve Item MS walking scale':ti,ab OR '2-minute walk test':ti,ab OR '2 minute walk test':ti,ab OR 'two minute walk test':ti,ab OR '6-minute walk test':ti,ab OR '6 minute walk test':ti,ab OR 'six minute walk test':ti,ab OR 'Timed Up and GO':ti,ab OR 'Dynamic gait index':ti,ab OR 'physical ponent summery':ti,ab OR 'Six spot step test':ti,ab OR 'Timed 25-Foot Walk':ti,ab OR 'Timed 25 Foot Walk':ti,ab OR 'Nine-Hole Peg Test':ti,ab OR 'Nine Hole Peg Test':ti,ab OR '9-Hole Peg Test':ti,ab OR '9 Hole Peg Test':ti,ab) |
|  | SCOPUS |
| 3. | TITLE-ABS ( ( sclerosis AND multiple ) OR ( sclerosis AND disseminated ) OR "disseminated sclerosis" OR "multiple sclerosis" OR "acute fulminating" ) AND TITLE-ABS ( ( imaging AND "Magnetic Resonance" ) OR "NMR Imaging" OR ( imaging AND nmr ) OR "MR Tomography" OR "NMR Tomography" OR ( tomography AND nmr ) OR ( tomography AND mr ) OR "Magnetic Resonance Image" OR ( image AND "Magnetic Resonance" ) OR "Magnetic Resonance Images" OR ( "Resonance Image" AND magnetic ) OR "Magnetization Transfer Contrast Imaging" OR "MRI Scans" OR "MRI Scan" OR ( scan AND mri ) OR ( scans AND mri ) OR "MR imaging" OR "corpus callosum" OR "third ventricle" OR thalamus OR "gray matter" OR "grey matter" OR "white matter" OR "caudate" OR "temporal lobe" OR "globus pallidus" OR cerebellum OR "Basal ganglia" OR "neocortical atrophy" OR cortex OR "cortical atrophy" OR "brain volume" OR amygdala OR "brain parenchymal fraction" OR "brain lesion volume" OR "brain lesion load" OR "brain stem" OR "cerebellar volume" OR "cortical volume" OR "hippocampus" OR insula OR neocortex OR "nucleus accumbens" OR putamen OR "T1-lesion" OR "T2-lesion" OR "temporal pole" OR atrophy ) AND TITLE-ABS ( "Expanded Disability Status Scale" OR "Patient determined disease steps" OR disability OR "disease progression" OR "Confirmed disability progression" OR "12-Item MS walking scale" OR "12 Item MS walking scale" OR "twelve Item MS walking scale" OR "2-minute walk test" OR "2 minute walk test" OR "two minute walk test" OR "6-minute walk test" OR "6 minute walk test" OR "six minute walk test" OR "Timed Up and GO" OR "Dynamic gait index" OR "physical ponent summery" OR "Six spot step test" OR "Timed 25-Foot Walk" OR "Timed 25 Foot Walk" OR "Nine-Hole Peg Test" OR "Nine Hole Peg Test" OR "9-Hole Peg Test" OR "9 Hole Peg Test" ) |
|  | WOS |
| 4. | TI=((Sclerosis AND multiple) OR (sclerosis AND disseminated) OR "disseminated sclerosis" OR "multiple sclerosis" OR "acute fulminating") AND TI=((Imaging AND "Magnetic Resonance") OR "NMR Imaging" OR (Imaging AND NMR) OR "MR Tomography" OR "NMR Tomography" OR (Tomography AND NMR) OR (Tomography AND MR) OR "Magnetic Resonance Image" OR (Image AND "Magnetic Resonance") OR "Magnetic Resonance Images" OR ("Resonance Image" AND Magnetic) OR "Magnetization Transfer Contrast Imaging" OR "MRI Scans" OR "MRI Scan" OR (Scan AND MRI) OR (Scans AND MRI) OR "MR imaging" OR "corpus callosum" OR "third ventricle" OR thalamus OR "gray matter" OR "grey matter" OR "white matter" OR "caudate" OR "temporal lobe" OR "globus pallidus" OR cerebellum OR "Basal ganglia" OR "neocortical atrophy" OR cortex OR "cortical atrophy" OR "brain volume" OR amygdala OR "brain parenchymal fraction" OR "brain lesion volume" OR "brain lesion load" OR "brain stem" OR "cerebellar volume" OR "cortical volume" OR "hippocampus" OR insula OR neocortex OR "nucleus accumbens" OR putamen OR "T1-lesion" OR "T2-lesion" OR "temporal pole" OR atrophy) AND TI=("Expanded Disability Status Scale" OR "Patient determined disease steps" OR disability OR "disease progression" OR "Confirmed disability progression" OR "12-Item MS walking scale" OR "12 Item MS walking scale" OR "twelve Item MS walking scale" OR "2-minute walk test" OR "2 minute walk test" OR "two minute walk test" OR "6-minute walk test" OR "6 minute walk test" OR "six minute walk test" OR "Timed Up and GO" OR "Dynamic gait index" OR "physical component summary" OR "Six spot step test" OR "Timed 25-Foot Walk" OR "Timed 25 Foot Walk" OR "Nine-Hole Peg Test" OR "Nine Hole Peg Test" OR "9-Hole Peg Test" OR "9 Hole Peg Test") |
