# Supplementary material for: Neuroimaging markers and disability scales in multiple sclerosis: A systematic review and meta-analysis
Source: PLoS One. 2024 Dec 5;19(12):e0312421. doi: 10.1371/journal.pone.0312421 (PMC11620670; doi:10.1371/journal.pone.0312421)
Supplement: S2 File — (DOCX) [file pone.0312421.s003.docx]

| **First Author** | **Country** | **Years** | **Study design** | **Sample size/**  **F: M/**  **Age; mean (SD)** | **MS type (n)** | **EDSS** | **DD**  **(Year)**  **mean (SD)** | **MRI device/ MRI type** | **Disability measurement method** | **Key finding(s)** | **QA** |
| --- | --- | --- | --- | --- | --- | --- | --- | --- | --- | --- | --- |
| M. Kim  (1) | Korea | 2024 | Cross-sectional | 53  41:12  32.3 (11.8) | NR | 2 (0-6)** | 2.9 (4.2) | NR  1.5 T | EDSS | EDSS was not correlated to total lesion load in MS. | 6 |
| K. A. Koenig  (2) | USA | 2024 | Cross-sectional | 80  61:19  51.9 (8.1) | RRMS: 64  SPMS: 16 | 3.7 (1-6.5)** | 16 (1-42)‡ | Siemens  7 T | EDSS  T25FW  9HPT  MSFC | MSFC was related to BPF and lesion volume among pwMS. | 6 |
| P. C. G. Molenaar  (3) | Netherland | 2024 | Case-control | 92  67:25  48.5 (38.8-54.3)* | RRMS: 55  SPMS: 27  PPMS: 10 | 3.5 (2.5-4)* | 9.2 (8.2) | General Electric  3 T | EDSS  T25FW  9HPT | 9HPT was correlated to brain lesion among pwMS, while EDSS and T25FW were not. | 8 |
| F. Romano`  (4) | Italy | 2024 | Case-control | 60  38:22  43.7 (11.4) | RRMS: 44  SPMS: 16 | 4 (2-4.5)* | 15.4 (8.8-21.3)* | NR  3 T | EDSS  T25FW  9HPT | Higher T1/T2 ratio values in the globus pallidus region were associated with greater disability severity in pwMS. | 7 |
| E. Vanheule  (5) | Belgium | 2024 | Cross-sectional | 84  53:31  46.4 (11.8) | RRMS: 69  SPMS: 4  PPMS: 11 | 3.5 (1.6) | 61 (39.2)^m^ | Philips  3 T | EDSS  T25FW  9HPT | 9HPT was significantly correlated to paramagnetic rim lesion count in pwMS. | 5 |
| J. Sun  (6) | China | 2024 | Cross-sectional | 324  122:202  35 (10.9) | RRMS: 324 | 2.5 (1.7) | 47.6 (60.6) | NR | EDSS | There were relationships between EDSS and regional GM volumes in MS. | 9 |
| X. Wang  (7) | China | 2024 | Case-control | 163  126:37  39.2 (10.6) | RRMS: 163 | 2 (1-2.5)* | 3.2 (1.1-8)* | Siemens  3 T | EDSS | EDSS was correlated with normalized choroid plexus volume among pwMS. | 8 |
| Y. Xie  (8) | China | 2024 | Case-control | 65  41:24  35.4 (12.1) | RRMS: 65 | 3 (1.5) | 3.7 (4.3) | General Electric  3 T | EDSS | EDSS was positively correlated with choroid plexus volume in MS. | 6 |
| R. Zivadinov  (9) | USA | 2024 | Cohort | 732  362:370  44.6 (8) | NR | 4.7 (1.2) | 6.5 (3.9) | NR | EDSS  T25FW  9HPT | T2LL was mildly correlated with EDSS, T25FW, and 9HPT in pwMS. | 8 |
| J. G. Harper  (10) | UK | 2024 | Cohort | 62  48:14  37.2 (10.9) | RRMS: 62 | 2 (0-6)** | NR | Siemens  3 T | EDSS | EDSS was associated with WM lesion count in MS. | 6 |
| M. Barnett  (11) | Australia | 2023 | Cohort | 282  198:84  46 (21.1-75.9)‡ | RRMS: 258  SPMS: 17  PPMS: 3  CIS: 1  RIS: 3 | 1.5 (0-7)**  for n=315 | 13.1 (0.7-41.8)‡ | NR | EDSS | EDSS had significant association with normalized volume of brain volume, thalamic volume, and grey matter volume in MS. | 8 |
| A. Calvi  (12) | UK | 2023 | Cohort | 61  42:19  34.4 (14.1-64.9)** | RRMS: 55  CIS: 6 | 1.5 (0-4.5)** | 0.4 (0.1-16.6)** | Siemens  3 T | EDSS | Paramagnetic rim lesions and slowly expanding lesions had correlations with disability in MS. | 7 |
| L. R.J. Ruiter  (13) | Netherlands | 2023 | Case-control | 234  170:64  52.8 (0.9) | RRMS: 146  SPMS: 55  PPMS: 33 | 3.5 (2.5-4)* | 15.7 (8.7) | General Electric  3 T | EDSS  T25FW  9HPT | Physical disability was most strongly associated with the volumes of the spinal cord and deep gray matter in MS. | 9 |
| N. Bergsland  (14) | USA | 2023 | Case-control | 174  129:45  47.5 (10.8) | RRMS: 118  PMS: 56 | 2.5 (1.5-5.5)* | 15.2 (10) | General Electric  3 T | EDSS | Normalized choroid plexus volume was associated with EDSS in MS | 9 |
| M. Hamzaoui  (15) | France | 2023 | Case-control | 36  21:15  48 (11) | RRMS: 12  SPMS: 13  PPMS: 11 | 4.5 (1.5) | 9 (6) | Siemens  3 T | EDSS | No significant association was found between NAWM and cortical binding and EDSS in MS. | 6 |
| A. Hartmann  (16) | Brazil | 2023 | Cross-sectional | 95  60:35  47.1 (14.1) | RRMS: 73  SPMS: 13  PPMS: 9 | 3.9 (2.4) | 17.5 (12) | Siemens  1.5 T | EDSS | While total lesion load was correlated with disability in RRMS patients, this relationship was not significant in SPMS and PPMS patients. | 6 |
| A. Hofmann  (17) | Austria, UK | 2023 | Cross-sectional | 98  57:41  38 (10.5) | RMS: 76  SPMS: 22 | 2 (1-3.5)* | 6 (2.8-11.3)* | Siemens, Vida  3 T | EDSS  MSSS | There was significant relationship between EDSS and paramagnetic rim lesion in MS. | 7 |
| S. Noteboom  (18) | Netherland | 2023 | Case-control | 329  224:105  48 (11) | RRMS: 242  PMS: 87 | 3 (2.5-4.5)* | 14.5 (8.4) | General Electric  3 T | EDSS | Brain volumes obtained from 3D-FLAIR and 3D-T1 imaging demonstrate comparable associations with disability in MS. | 9 |
| L. I. C. Peño  (19) | Spain | 2023 | Cross-sectional | 41  27:14  43.9 (11.1) | RRMS: 31  SPMS: 6  PPMS: 4 | 2.6 (1.9) | 8 (1.1) | NR | EDSS | T2LL and GM volume had relationships with EDSS in MS. | 6 |
| G. Pontillo  (20) | Italy | 2023 | Case-control | 117  85:32  40.6 (11.9) | RRMS: 85  SPMS: 22  PPMS: 10 | 3 (2-5.2)* | 12.7 (8.3) | Siemens  3 T | EDSS | T2LL and GM volume were associated with disability among pwMS. | 9 |
| D. Slezáková  (21) | Slovakia | 2023 | Cross-sectional | 147  101:46  42.1 (11) | RRMS: 123  SPMS: 21  PPMS: 3 | 3 (1-7)** | 6.1 (4.3) | NR | EDSS | EDSS was correlated to brain and GM volumes in MS. | 7 |
| M. R. Vidakovi´c  (22) | Croatia | 2023 | Cross-sectional | 23  14:9  41.6 (8.8) | RRMS: 25 | 2.5 (3.5)* | 9.3 (5.7) | Siemens  1.5 T | EDSS | EDSS was correlated to lesions count in right corticospinal tract among pwMS. | 5 |
| F. Steffen  (23) | Germany | 2022 | Cohort | 153  109:44  35.2 (13) | RRMS: 153 | 1.3 (1.2) | 8.4 (4.3) | Siemens  3 T | EDSS | There were relationships between number of T2 lesions and gadolinium enhancing lesion in MS. | 7 |
| M. Filippi  (24) | UK | 1994 | Cohort | 38  NR  NR | NR | NR | NR | Picker  0.5 T | EDSS | Lesion load was associated with the severity of disability in MS patients. | 5 |
| M. Filippi  (25) | Italy | 1996 | Case-control | 32  19:13  38.4 (9.1) | SPMS: 17  BMS: 15 | SPMS:  5.5 (3.5-6)**  BMS:  1 (0-3)** | SPMS:  7 (4-18)**  BMS:  14 (11-28)** | NR  1.5 T | EDSS | EDSS and brain MRI lesion load did not show a relationship.  A negative correlation was found between the EDSS score and both the spinal cord cross-sectional area and the transverse diameter. | 5 |
| B. Genç  (26) | Turkey | 2023 | Cross-sectional | 65  NR  40.2  (12) | RRMS: 65 | 1.5  (3)* | NR | Philips  3 T | EDSS | Posterior hypothalamus, and total hypothalamus volume were negatively associated with TLV and EDSS scores. | 7 |
| A. Gass  (27) | UK | 1994 | Case-control | 43  NR  RRMS:  28.7  (22-44)‡  SPMS:  42.1  (29-51)‡  PPMS:  42.4  (32-48)‡  Benign:  45.8  (31-60)‡ | RRMS: 11  SPMS: 11  PPMS: 10  BMS: 11 | RRMS:  2.9  (1.5-6.5)‡  SPMS:  6.1  (5-8)‡  PPMS:  4.7  (3-7)‡  Benign:  2.7  (1.5-3)‡ | RRMS:  3  (1.5-4.5)‡  SPMS:  11.9  (4-22)‡  PPMS:  10.2  (5-22)‡  Benign:  21.4  (14-32)‡ | General Electric  1.5 T | EDSS | There was an overall correlation of total lesion area with disability. Also, there was no correlation between EDSS and average NAWM MTR. | 4 |
| M. Filippi  (28) | UK | 1995 | Cohort | 281  161:120  39.7  (10.3) | RRMS: 84  BMS: 30  SPMS: 144  PPMS: 23 | 3.5  (0-7.5)** | 8  (1-46)** | Picker  0.5 T | EDSS | The number of all active lesions were associated with the changes of disability severity. | 7 |
| C. Gasperin  (29) | UK | 1996 | Case-control | 41  NR  41.2 (8.3)‡‡ | RRMS: 10  SPMS: 11  PPMS: 10  BMS: 10 | 5.8 (2)‡‡ | 8.9 (7)‡‡ | General Electric  1.5 T | EDSS | A macroscopic lesion is more important than a microscopic abnormality in the NAWM when it comes to the development of MS disability | 5 |
| M. L. Gawne-Cain  (30) | UK | 2015 | Cross-sectional | 56  27:29  Age:  RRMS: 42 (31-55)**  SPMS: 42 (29-57)**  PPMS: 42 (29-57)**  BMS: 40 (34-59)** | RRMS: 15  SPMS: 20  PPMS: 7  BMS: 14 | RRMS: 3 (1-6.5)**  SPMS: 6 (4.8-8)**  PPMS: 7 (6-8)**  BMS: 2 (0-3)** | RRMS: 9 (2-14)**  SPMS: 9.5 (5-21)**  PPMS: 7 (3-12)**  BMS: 11 (10-31)** | General Electric  1.5 T | EDSS  FSS | It was faster and easier to acquire and process fFLAIR images than CSE images, however, the images were not significantly more reproducible nor correlated with disability than CSE images. Brain lesion volume was significantly correlated with EDSS in both CSE (r= 0.49) and fFLAIR (r= 0.44). | 5 |
| D. Berg  (31) | Germany | 2000 | Case-control | 74  48:26  42 (18-70)‡ | RRMS: 34  SPMS: 32  PPMS: 8 | 5.5 (1-8)** | 9.4 (0.2-35)‡ | NR  1.5 T | EDSS | The diameter of the third ventricle correlates with disability status and may also indicate the progression of the disease. | 5 |
| Y. Ge  (32) | USA | 2000 | Case-control | 36  27:9  37.8 (23-52)‡ | RRMS: 27  SPMS: 9 | 2.5 (1-6.5)** | 3.3 (0.5-20)** | General Electric  1.5 T | EDSS | EDSS scores and brain atrophy were better correlated in SPMS patients than in RRMS. | 4 |
| R. Bakshi  (33) | USA | 2001 | Case-control | 114  89:43  43 (9) | RRMS: 80  SPMS: 34 | 3 (0-9)^ | 9.6 (9) | NR  1.5 T | EDSS | There was a strong correlation between brain atrophy and physical disability in MS patients. | 7 |
| R. Bakshi  (33) | USA | 2001 | Case-control | 36  NR  NR | RRMS: 14  SPMS: 22 | 3 (1-8.5)^ | NR | NR  1.5 T | EDSS | There was a strong correlation between brain atrophy and physical disability in MS patients. | 5 |
| S.J. Hickman  (34) | UK | 2001 | Cross-sectional | 9  NR  42 (18-54)** | RRMS: 2  SPMS: 7 | 8 (3-8.5)** | 13 (3-29)** | General Electric  1.5 T | EDSS | The presence of infratentorial T1 hypointense lesions was common among patients with MS, which may contribute significantly to their disability. | 5 |
| N.F. Kalkers  (35) | Netherlands | 2001 | Case-control | 137  85:52  41.7  (9.6) | RRMS: 80  SPMS: 36  PPMS: 21 | 2.5  (1.5-5.5)* | 11.4  (8.4) | Siemens  1 T, 1.5 T | EDSS  MSFC  T25FW  9HPT | Early in the disease, focal demyelination was important to determine disability; however, later in the disease, residual brain volume was more important to determine disability. | 8 |
| M. Filippi  (36) | Italy | 2001 | Cross-sectional | 78  42:36  41.6  (9.5) | RRMS: 28  SPMS: 20  PPMS: 30 | 5  (0-8.5)** | 10  (1-28)** | Siemens  1.5 T | EDSS | T2 lesion volume, and T1 lesion volume were associated with EDSS score. | 5 |
| R. Bakshi  (37) | USA | 2002 | Case-control | 60  41:19  42 (9) | RRMS: 42  SPMS: 18 | 3.7 (1.9) | 10.6 (9.4) | Philips  NR | EDSS | As compared to conventional MRI findings, GM T2 hypointensity was a more powerful indicator of disability and clinical course in MS. | 6 |
| O. Ciccarelli  (38) | UK | 2002 | Cross-sectional | 41  24:14  45 (34-62)** | RRMS: 29  SPMS: 7  CIS: 5 | 2 (0-8)** | NR | General Electric  1.5 T | EDSS  MSFC  T25FW  9HPT | T2 lesion loads demonstrated significant correlations with EDSS (FSE, r= 0.53 and 3D-fFLAIR, r= 0.51), T25FW (FSE, r= 0.48 and 3D-fFLAIR, r= 0.48), and 9HPT (FSE, r= 0.54 and 3D-fFLAIR, r= 0.52). There was a greater correlation between T1LL and EDSS compared to either T2LL measure. T1LL and MSFC scores only showed a trend relationship. | 5 |
| C.M. Griffin  (39) | UK | 2002 | Case-control | 27  19:8  35.7 (24-54)‡ | RRMS: 27 | 1 (0-3)** | 1.7 (1-3)** | General Electric  1.5 T | EDSS  MSFC  9HPT  T25FW | T25FW was associated with T2LL and T1LL. Lesion load measures and EDSS did not correlate. | 6 |
| M. Hardmeier  (40) | Switzerland | 2003 | Cohort | 138  NR  38.5  (7.1) | RRMS: 138 | 3.5  (2-5.5)** | 7.6  (5.4) | NR | EDSS | BPF and T2LL were associated with EDSS. Changes in BPF did not correlate with changes of clinical and MRI measurements | 8 |
| R. A. Bermel  (41) | USA | 2003 | Case-control | 78  51:27  42.5 (8.3) | RRMS: 60  SPMS: 18 | 3.4 (1.8) | 11.8 (8.7) | Philips  1.5 T | EDSS | EDSS in MS patients was correlated with BPF, third ventricle width, and T2 lesion volume. | 7 |
| C. Caon  (42) | USA | 2003 | Cross-sectional | 190  142:48  40.2 (9.3) | RRMS: 163  SPMS: 27 | 3.2 (1.7) | 8.5 (7.2) | NR | EDSS | The intercaudate ratio in MS was related to disability and disease duration. | 8 |
| N. De Stefano  (43) | Italy | 2003 | Case-control | 90  60:30  38 (18-67)** | RRMS: 65  PPMS: 25 | 2.6 (1.6) | 7.2 (6.8) | NR | EDSS | A strong correlation existed between NCV and EDSS in PPMS compared to RRMS. | 6 |
| F. Agosta  (44) | Italy | 2007 | Case-control | 18  12:6  39  (24-55)‡ | RRMS: 18 | 3.5  (1-5)** | 8  (1-14)‡ | NR  1.5 T | EDSS | There is a moderate association between cervical cord GM injury and the degree of disability. | 5 |
| J. Dehmeshki  (45) | UK | 2003 | Case-control | 30  10:20  40.7 (25-51)‡ | PPMS: 30 | 5.5 (2-7)** | 7.3 (2-19)‡ | General Electric  1.5 T | EDSS | NAGM MTR and EDSS had a significant association. | 6 |
| S. J. Hickman  (46) | UK | 2003 | Case-control | 28  11:17  46.3€ | RRMS: 6  SPMS: 6  PPMS: 12  BMS: 4 | NR | 12.5€ | General Electric  1.5 T | EDSS | Baseline spinal cord volume and EDSS were significantly associated. | 6 |
| C. J. Archibald  (47) | Canada | 2004 | Case-control | 20  15:5  48.5 (7.8) | RRMS: 7  SPMS: 13 | 4.2 (1.9) | 4.6 (3.8) | General Electric  3 T | EDSS | Both whole brain T2 lesion volume and whole brain T1 lesion volume were not significantly correlated with EDSS scores. | 5 |
| G. R. Davies  (48) | UK | 2004 | Case-control | 38  28:10  36.3 (24-56)‡ | RRMS: 38 | 1.5 (0-3)** | 1.9 (0.5-3.7)‡ | General Electric  1.5 T | EDSS  MSFC | According to the correlation between EDSS and GM MTR, an abnormal NAGM MTR in the early stages of RRMS may be of clinical significance. | 6 |
| L. K. Fisniku  (49) | UK | 2008 | Cohort | 73  49:24  51.3  (7.1) | RRMS: 33  SPMS: 11  CIS: 29 | 2.5  (0-8)** | 20  (1.5) | General Electric  1.5 T | EDSS  MSFC  9HPT  T25FW | GM fraction was a better predictor of disability than T2 lesion load. GM atrophy was related to long term disability. | 7 |
| B. Bejarano  (50) | Spain | 2011 | Cohort | 51  33:18  35.1 (8.9) | RRMS: 26  SPMS: 3  PPMS: 4  PRMS: 2  CIS: 16 | 2  (0-6)** | 5.9  (7.4) | Siemens  1.5 T | EDSS  MSFC  MSSS  TWT | The neural network was the most accurate classifier for predicting the change of EDSS during two years follow up. | 5 |
| C. O. Brandão  (51) | Brazil | 2006 | Case-control | 50  36:14  30 (9.6) | RRMS: 50 | 2.7 (1.1) | 10 (5.8) | Elscint  2 T | EDSS | It was found that brain TLV and total plaque count were statistically significant correlates with EDSS. | 6 |
| M. Calabrese  (52) | Italy | 2011 | Cohort | 168  120:48  32.3  (7.2) | RRMS: 168 | 1.5  (0-4)** | 6.8  (2.4) | Philips  1.5 T | EDSS | NAGM-FA changes could predict the changes of EDSS. | 7 |
| J. Cohen-Adad  (53) | USA | 2011 | Case-control | 17  11:6  39.8  (11.8) | RRMS: 10  SPMS: 7 | 3.5  (1-6.5)** | 11.7  (7.8) | Siemens  3 T, 7 T | EDSS | There was a correlation between EDSS and T2*-weighted  signal averaged in the precentral gyrus. | 6 |
| A. B. Cohen  (54) | USA | 2011 | Cross-sectional | 21  NR  40.9  (8) | RRMS:  18  SPMS: 1  PPMS: 1  CIS: 1 | 1.6  (1.6) | 8.3  (7.5) | General Electric  3 T | EDSS  T25FWT | Cervical spinal cord was associated with disability. Spinal cord lesion and volume was associated with brain lesion and volume. | 7 |
| S. Hannoun  (55) | France | 2012 | Case-control | 41  26:15  37.2  (7.6) | RRMS: 23  SPMS: 18 | 3.7  (2) | 10.1  (6.2) | Siemens  1.5 T | EDSS  MSFC | Concerning all  MR imaging metrics, the best predictor of disability was  the FA measurement in the caudate. | 7 |
| H. Hildebrandt  (56) | Germany | 2006 | Cross-sectional | 45  29:16  38.9 (23-63)‡ | RRMS: 45 | 2.6 (0-6)‡ | 5 (0.1-20)‡ | Philips  1.5 T | EDSS  MSFC  T25FW  9HPT | The BPF and VF were moderately correlated with the EDSS as well as the upper extremity motor performance. | 7 |
| M. Calabrese  (57) | Italy | 2007 | Case-control | 380  284:96  37.4 (7.9) | RRMS: 163  SPMS: 101  CIS: 116 | 2.7 (0-7)‡ | 5.6 (5.1) | Philips  1.5 T | EDSS | EDSS scores and the number of ICLs were also significantly associated in all MS groups, RRMS, and SPMS. | 7 |
| A. Damasceno  (58) | Brazil | 2014 | Case-control | 42  32:10  30.5  (6.6) | RRMS: 42 | 2.5  (0-4)** | 6.4  (4.9) | Phillips  3 T | EDSS  9HPT  T25FW | Cerebellar intracortical lesions were associated with EDSS and T25FW.  Brain cortical volume and cerebellar GM volume were associated with T25FW. | 7 |
| V. Janardhan  (59) | USA | 2007 | Cross-sectional | 145  112:33  43 (9.3) | RRMS: 92  SPMS: 49  NR | 3.5 (0-8.5)‡ | 9.6 (0-38)‡ | General Electric  1.5 T | EDSS | A common feature of T1-weighted MR images of MS is hyperintense plaques, which are associated with brain atrophy, disability, and disease progression. | 7 |
| R. Bakshi  (60) | USA | 2008 | Cohort | 103  73:30  42.7 (9.1) | RRMS: 62  SPMS: 33  PPMS: 8 | 3.3 (0-8.5)‡ | 14.1 (9.2) | NR  1.5 T | EDSS | BPF, T2LV, T1:T2, and MRDSS were associated with EDSS in PwMS. | 8 |
| J. Furby  (61) | UK | 2008 | Cross-sectional | 117  84:33  50.4 (6.9) | SPMS: 117 | 6 (0.5)* | 19.9 (8.9) | General Electric  1.5 T | EDSS | A measure of atrophy, particularly of the whole brain and spinal cord, may serve as a useful disease marker in SPMS. | 9 |
| J. Bellmann-Strobl  (62) | Germany | 2009 | Cohort | 17  10:7  33 (7.9) | RRMS: 17 | NR | NR | Siemens  1.5 T | EDSS  MSFC  9HPT | The correlation between MTI and MRS with clinical scores indicated that these measurements can be used to monitor the progression of the disease. | 5 |
| M. Deppe  (63) | Germany | 2014 | Case-control | 30  NR  36 (26-56)** | RRMS: 30 | 1.5 (1-5.5)** | 6 (1.1-22.1)** | Siemens  3 T | EDSS | WM lesion load and GM volume did not correlate with the EDSS.  EDSS was related to increased cortical extrinsic curvature. | 6 |
| M. Calabrese  (64) | Italy | 2009 | Case-control | 48  28:20  41.2 (5.1) | PPMS: 48 | 4 (3-6.5)‡ | 4 (3.2) | Philips  1.5 T | EDSS | CL number and CL volume were correlated with EDSS in MS. | 6 |
| T. Hayton  (65) | UK | 2009 | Cross-sectional | 113  80:33  50.2 (29-60)‡ | SPMS: 113 | 6 (6-6.5)* | 19.9 (3-41)‡ | General Electric  1.5 T | EDSS | In NAWM, GM, and lesions, significant correlations were observed between MSFC composites and mean MTRs. | 8 |
| K. C. Gao  (66) | USA | 2014 | Cross-sectional | 83  46:37  NR | RRMS: 30  SPMS: 17  PPMS: 29  CIS: 6  RIS: 1 | 3.5 (2-6)* | NR | Siemens  3 T | EDSS  9HPT  T25FW | Concerning clinical measurements, disability scores such as 9HPT was more associated with BFW fraction than EDSS. | 7 |
| H. Kearney  (67) | UK | 2013 | Cross-sectional | 159  111:48  52 (8.8) | RRMS: 92  SPMS: 67 | 4 (0-8)** | 26.2 (6.7) | Phillips, Siemens, General Electric  1.5 T, 3 T | EDSS | Disability was related to atrophy of spinal cord, brain T2 lesion load, and brain GM atrophy. | 8 |
| S. Datta  (68) | USA | 2015 | Cross-sectional | 924  674:250  37.5 (9.6) | RRMS: 924 | 1.9 (1.1) | 1.6 (3.2) | Phillips  1.5 T, 3 T | EDSS | Thalamus, amygdala and pons volumes were associated with EDSS. | 8 |
| S. Dupuy  (69) | USA | 2015 | Cross-sectional | 45  33:12  42.3 (8.3) | RRMS: 37  SPMS: 8 | 3.5 (2) | 11 (7.8) | Phillips  1.5 T | EDSS  T25FW | T1 spin-echo lesion volume correlated with EDSS score whereas T1 gradient echo and FLAIR lesion volume did not. No significant correlation was found between lesion volume and T25FW. The correlation between EDSS and T1 gradient echo lesion count was 0.19. The correlation between EDSS, T1 gradient echo volume and flair lesion volume was 0.23 and 0.24, respectively. T25FW correlated with T1 gradient echo lesion volume (r =0.25) and flair lesion volume (r= 0.24). | 5 |
| B. Benedetti  (70) | Italy | 2009 | Case-control | 68  46:22  SPMS:  46.5 (32–68)‡  BMS:  45.2 (27–62)‡ | SPMS: 28  BMS: 40 | SPMS:  6 (4–8.5)**  BMS:  2 (0–3)** | SPMS:  17 (5–41)‡  BMS:  22.1 (15–32)‡ | NR  1.5 T | EDSS | EDSS was associated with T2LV and cord CSA. Cervical cord lesions and EDSS did not correlate. | 6 |
| A. Giorgio  (71) | Austria | 2010 | Cross-sectional | 45  33:12  29 (19-45)** | RRMS: 45 | 1.5 (0-4)** | 3 (0.8-27)** | Philips  1.5 T | EDSS | There was no significant correlation between T2LV and EDSS. | 4 |
| J. Martola  (72) | Sweden | 2010 | Cohort | 37  26:11  42 (10.3) | RRMS: 16  SPMS: 17  PPMS: 4 | NR | (1-33)^^ | General Electric  1.5 T | EDSS  MSSS | In both baseline and follow-up analyses, the relationship between brain parenchymal volume or supratentorial ventricular volume and EDSS was weak to moderate. | 6 |
| J. V. Gonyea  (73) | USA | 2015 | Cross-sectional | 23  17:6  43 (9) | RRMS: 13  CIS: 10 | 1.7 (0.9) | 6.1€ | Phillips  3 T | EDSS | There was a negative association between EDSS and T1rho and T2 NAGM. There was no correlation between EDSS and mean T1q, T2 of NAWM, T1q in WM lesions, T2 WM lesion. | 5 |
| F. Bonneville  (74) | USA | 2002 | Cross-sectional | 49  36:13  39 (24-55)‡ | RRMS: 49 | 2 (0-6)‡ | 8.7 (0.8-23.5)‡ | NR  1.5 T | EDSS | No correlation was found between either T2-weighted lesion volumes or EDSS scores and whole-brain N-acetylaspartate levels. Lesion volume was weakly correlated with EDSS score. | 5 |
| M. Calabrese  (75) | Italy | 2012 | Cross-sectional | 312  222:90  36.4 (10.8) | RRMS: 157  SPMS: 31  PPMS: 44  BMS: 45  PMS: 35 | RRMS:  2 (1-5.5)‡  SPMS:  4.5 (2.5-6)‡  PPMS:  4 (3-6.5)‡  BMS:  1.5 (1-3)‡  PMS:  1.5 (0-2.5)‡ | 6.9 (6.3) | Philips  1.5 T | EDSS | EDSS was associated with the number of CLs, CL volume, GM fraction, and WM lesion number. | 8 |
| Z. Caramanos  (76) | Canada | 2012 | Cross-sectional | 110  77:33  40.1 (10.1) | RRMS: 82  SPMS: 28 | RRMS:  2 (0-7.5)**  SPMS:  6.5 (3.5-9.5)** | 10.9 (8.2) | Philips  1.5 T | EDSS | The CWM-LL and clinical disability as measured by the EDSS had a large, non-plateauing relationship. | 8 |
| S. L. Dupuy  (77) | USA | 2016 | Cohort | 16  14:2  47.7 (7.5) | RRMS: 16 | 1.5 (0-2.5)‡ | 15 (10.3) | General  Electric  3 T | EDSS  T25FW | EDSS and T25FW were not related to cervical spinal cord area. | 5 |
| K. Hackmack  (78) | Germany | 2012 | Cross-sectional | 40  20:20  35.5 (7.4) | RRMS: 40 | 2 (0-5)** | 6.8 (6.4) | Siemens  1.5 T | EDSS  T25FW  9HPT | T25FW and 9HPT were correlated with T1LL and T2LL in PwMS. | 5 |
| B. C. Healy  (79) | USA | 2011 | Case-control | 34  26:8  41.6 (8.9) | RRMS: 26  SPMS: 4  PPMS: 2  CIS: 2 | 1.9 (0-6.5)‡ | 8.4 (8.6) | General Electric  3 T | EDSS  T25FW | Neurologic disability and MRI-based spinal cord volumes were correlated in MS. | 6 |
| J. Jaworski  (80) | Poland | 2012 | Cross-sectional | 48  27:21  36.2 (11.4) | RRMS: 34  SPMS: 14 | 3.6 (1-7.5)‡ | 6.4 (5.5) | General Electric  1.5 T | EDSS  MSSS | EDSS had a significant relationship with brain volume, BPF, and T1 lesion load. | 6 |
| S. L. Dupuy  (81) | USA | 2016 | Cohort | 28  21:7  45.1 (9.3) | RRMS: 26  CIS: 2 | 1.2 (1.1) | 10.7 (8) | Siemens  3 T | EDSS | No associations were found between deep GM or T2 lesion volume and disability measurements such as EDSS or T25FW. | 6 |
| J. M. Abalo-Lojo  (82) | Spain | 2014 | Case-control | 88  62:26  39.2 (9.6) | RRMS: 68  SPMS: 9  CIS: 11 | NR | NR | Siemens  1.5 T | EDSS | There was a significant correlation between bicaudate ratio load and EDSS in MS patients. | 6 |
| M. Grothe  (83) | Germany | 2016 | Cross-sectional | 213  140:73  41.3 (12) | RRMS: 163  SPMS: 50 | RRMS:  1.5 (0-6)**  SPMS:  6 (3.5-9) | 8 (7.7) | Siemens  3 T | EDSS | Cerebellar GM volume loss correlated with increasing EDSS and white matter lesion volume. | 8 |
| R. Bakshi  (84) | USA | 2014 | Case-control | 55  38:17  41.1 (9) | RRMS: 46  SPMS: 4  PPMS: 1  CIS: 4 | 1.6 (1.7) | 8.3 (7.4) | General Electric  3 T | EDSS | In MS patients, BPF, UCCA, and MRDSS2 were significantly correlated with EDSS. | 5 |
| E. A. Hubbard  (85) | USA | 2016 | Cross-sectional | 69  52:15  50.2 (8.9) | RRMS: 43  SPMS: 9  PPMS: 5  NR: 12 | 4.8 (1.9) | 11.9 (8.2) | Siemens  3 T | EDSS  T25FW | White matter volume correlated with EDSS and T25FW whereas GM volume only was associated with T25FW. | 6 |
| M. Daams  (86) | Netherlands | 2014 | Case-control | 196  132:64  53.4 (9.6) | RRMS: 125  SPMS: 49  PPMS: 22 | 4 (1-8)** | 19.9 (7) | General Electric  3 T | EDSS  T25FW  9HPT | MUCCA was correlated with EDSS, T25FW, and 9HPT. Cervical cord lesions were associated with EDSS and T25FW. | 8 |
| A. Burgetova  (87) | Czech Republic | 2017 | Cross-sectional | 115  NR  47.4 (7) | RRMS: 91  SPMS: 24 | 3.2 (2.1) | 11.8 (10) | Philips  1.5 T | EDSS | EDSS positively correlated with T1 and T2 lesion volume whereas negatively was associated with BPF and CCV. | 8 |
| D. M. Harrison  (88) | USA | 2012 | Case-control | 117  80:37  45.2 (11.7) | RRMS: 66  SPMS: 30  PPMS: 21 | 3.5 (0-8)** | 10.9 (10.1) | Philips  3 T | EDSS  MSFC  T25FW  9HPT | EDSS correlated with optic radiation MTR while was not associated with other MTR factors among all MS patients. MSFC was related to MTR factors except supratentorial MTR in all MS patients. | 9 |
| E. Kantorová  (89) | Slovakia | 2014 | Case-control | 41  28:13  36.1 (9.9) | RRMS: 37  SPMS: 2  CIS: 2 | 2.5 (1.1) | 6.1 (1) | NR  1.5 T | EDSS | EDSS was significantly associated with brain atrophy and new T2 lesions in MS patients. | 5 |
| R. Chu  (90) | USA | 2017 | Case-control | 14  11:3  50.2 (8.2) | RRMS/CIS: 10  SPMS: 3  PRMS: 1 | 3.1 (2.1) | 18.4 (10.7) | General Electric  Siemens  1.5 T  3 T | EDSS  T25FW | EDSS correlated negatively with normalized volumes of deep GM in thalamus, globus pallidus, and putamen. | 7 |
| M. Amann  (91) | Switzerland | 2015 | Cross-sectional | 71  50:21  47.9 (23-70)‡ | RRMS: 59  SPMS: 12 | 3 (0-7.5)** | 17.1 (4-50)‡ | Siemens  1.5 T | EDSS  T25FW  9HPT | Lesional MTR was more important than MTR of normal-appearing brain structures in predicting outcomes of physical disability in relapse-onset MS. | 6 |
| N. Bergsland  (92) | Italy | 2015 | Cross-sectional | 51  28:23  42 (10.9) | RRMS: 51 | 4 (0-7)** | 10.4 (8.4) | Siemens  1.5 T | EDSS | EDSS showed a correlation with lesional CST volume in MS. | 6 |
| N. E. Fritz  (93) | USA | 2017 | Case-control | 29  17:12  48.7 (11.5) | RRMS: 29 | 4 (1-6.5)** | 11.9 (8.7) | Philips  3 T | EDSS  FSS  T25FW | EDSS was associated significantly with spinal cord area, unlike corticospinal tract magnetization transfer ratio. T25FW correlated with both corticospinal tract magnetization transfer ratio and spinal cord area. | 6 |
| E. Bernitsas  (94) | USA | 2014 | Cross-sectional | 150  97:53  41.3 (8.5) | RRMS: 93  PMS: 57 | 3.8 (0-7.5)‡ | 11.2 (4.5) | Siemens  3 T | EDSS | In both progressive and RRMS, CSA-C2 is strongly correlated with clinical disability. | 8 |
| V. Biberacher  (95) | Germany | 2014 | Case-control | 182  127:55  36 (19-66)‡ | RRMS: 182 | 1.5 (0-5.5)** | 4.5 (4.2) | Philips  3 T | EDSS  MSFC  T25FW  9HPT | UCCA of MS patients and their CV scores were correlated with their EDSS scores. | 8 |
| M. Daams  (96) | Netherlands | 2015 | Case-control | 195  131:64  53.4 (9.6) | RRMS: 124  SPMS: 49  PPMS: 22 | 4 (1-8)** | 19.9 (7) | General Electric  3 T | EDSS  T25FW  9HPT | There were correlations between MRI measures and disability in PwMS. | 9 |
| E. Dell’Oglio  (97) | USA | 2015 | Case-control | 61  42:19  41 (8.6) | RRMS: 51  SPMS: 5  PPMS: 1  CIS: 4 | 1.6 (0-8)‡ | 8.3 (7.2) | General Electric  3 T | EDSS  T25FW | There was a relationship between BPF and EDSS in MS patients. | 6 |
| O. Galego  (98) | Portugal | 2015 | Cross-sectional | 19  14:5  55.7 (7.6) | PPMS: 19 | 6 (3.5-8)** | 13 (8.8) | Siemens  1.5 T | EDSS | There was no direct relationship between physical disability and brain volume loss in PPMS. | 6 |
| T. Granberg  (99) | Sweden | 2015 | Cohort | 37  26:11  42 (10) | RRMS: 23  SPMS: 11  PPMS: 3 | 4.5 (0-8)** | 11 (8.5) | General Electrics  1.5 T | EDSS | CCA was a highly reliable and fast-acting biomarker that correlated significantly better with EDSS than CCI and volumetric measurements. | 7 |
| T. Granberg  (100) | Sweden | 2015 | Case-control | 37  26:11  42 (10) | RRMS: 23  SPMS: 11  PPMS: 3 | 4.5 (0-8)** | 11 (8.5) | General Electrics  1.5 T | EDSS | In time perspectives approaching two decades, the normalized corpus callosum area outperforms volumetric measurements in predicting disability. | 7 |
| D. M. Harrison  (101) | USA | 2015 | Case-control | 36  20:16  42.6 (10) | RRMS: 30  SPMS/PPMS: 6 | 3 (1-6.5)** | 9.8 (7.5) | Philips  7 T | EDSS  T25FW  9HPT | As a result, CLs appear to be associated with cognitive and physical disability in MS in vivo. | 7 |
| H. Kearney  (102) | UK | 2014 | Case-control | 83  49:34  42.5 (11.16) | RRMS: 33  SPMS: 29  CIS: 21 | RRMS:  2.5 (0-6)**  SPMS:  6.5 (4-8.5)**  PPMS:  1 (0-3)** | 9.8 (11) | Philips  3 T | EDSS | There was a significant contribution to physical disability of relapse-onset MS and SPMS caused by pathological involvement of spinal cord GM. | 6 |
| M. Calabrese  (103) | Italy | 2010 | Cohort | 107  72:35  RRMS:  34.2 (18-55)‡  SPMS:  41.1 (22-55)‡ | RRMS: 76  SPMS: 31 | RRMS:  2 (1.5-3.5)**  SPMS:  5 (2.5-6)** | RRMS:  4.8 (2-10)‡  SPMS:  11.6 (5-14)‡ | Siemens  1.5 T | EDSS | Baseline cortical lesion volume was correlated with EDSS change in RRMS and SPMS. | 8 |
| Y. Ge  (104) | USA | 2001 | Case-control | 18  15:3  34.1 (26-52)‡ | RRMS: 18 | 0-6.5^^ | 5.3 (1-15)‡ | General Electric  1.5 T | EDSS | There was an inverse association between EDSS and MTR histogram peak height. T2LL was not correlated with EDSS. | 7 |
| I. Dimitrov  (105) | Bulgaria | 2015 | Cross-sectional | 46  33:13  38.6 (9.1) | RRMS: 46 | NR | NR | NR | EDSS | Higher disability in RRMS patients were associated with lower GM and WM volumes and certain subcortical structures, as well as a higher load of T2 lesions. | 5 |
| E. C. Arpín  (106) | Spain | 2016 | Cohort | 29  17:12  40.5 (9.9) | RRMS: 19 | 3.3 (1.7) | 11.9 (5.4) | NR  1.5 T | EDSS | There was no correlation between the final CCI and the final EDSS. | 4 |
| R. Chu  (107) | USA | 2015 | Cross-sectional | 26  18:8  43 (21-55)‡ | RRMS: 22  SPMS/PPMS: 4 | 2 (0-6.5)‡ | 9.5 (8.6) | Philips  1.5 T  General Electric  3 T | EDSS | In MS patients, BPV-1.5T and BPV-3T showed a moderate to significant correlation with both EDSS (r= -0.43 and r= -0.49, respectively) score and T25FW (r= -0.46 and r= -0.56, respectively). | 6 |
| R. M. Gracien  (108) | Germany | 2016 | Case-control | 22  18:4  34.7 (8.7) | RRMS: 22 | 1.4 (0-3)‡ | 4 (6.5) | Siemens  3 T | EDSS | Cortical T1 relaxation time was correlated with EDSS in MS. | 6 |
| C. Ammitzbøll  (109) | Denmark | 2017 | Cross-sectional | 93  52:41  50 (45-54)* | SPMS: 56  PPMS: 37 | 5 (4.5-6)* | 9 (5-15)* | Siemens  3 T | EDSS  T25FW  9HPT | T2 lesion volume was correlated with EDSS. T2 lesion volume, T2 lesion MTR, NAWM MTR, and cortical GM MTR correlated with 9HPT. T25FW was associated with T2 lesion volume. | 6 |
| F.X. Aymerich  (110) | Spain | 2017 | Cohort | 31  12:19  51 (33-61)** | PPMS: 31 | 5.5 (3-6.5)** | 11.7 (2-33)‡ | Siemens  1.5 T | EDSS | Cervical cord atrophy correlated with EDSS progression and it can be predictive of disability. The annualized mean cervical cord area loss rate could predict the long term EDSS change. | 7 |
| J. D. Dworkin  (111) | USA | 2017 | Cross-sectional | 60  38:22  38 (9) | RRMS: 44  SPSM: 13  PPMS: 1  Idiopathic MS: 2 | NR | NR | General Electric  1.5 T | EDSS | There were not any significant associations between lesion load, lesion count, and disability measurements such as EDSS. | 6 |
| S. Klineova  (112) | USA | 2016 | Cohort | 13  7:6  53 (13) | RRMS: 5  SPMS: 6  PPMS: 2 | NR | 11 (7.9) | Siemens  3 T | T25FW | T25FW was significantly associated with GMV, WMV, and NBV. | 7 |
| E. Ciampi  (113) | Spain | 2016 | Cohort | 38  23:15  36.8 (20-60)‡ | RRMS: 36  SPMS: 2 | 4 (1-6.5)** | 10.3 (2-32)‡ | Siemens  1.5 T | EDSS | The EDSS change status and regional GM changes were not statistically significant. | 7 |
| L. L. Gonçalves  (114) | Brazil | 2018 | Cross-sectional | 24  14:10  28.8 (7.9) | RRMS: 24 | 2.5 (0-5)** | 1.4 (0.6-6.6)* | General Electric  3 T | EDSS  MSFC  T25FW  9HPT | There was a correlation between MSFC and corpus callosum index. There were no associations between corpus callosum index and EDSS, T25FW. | 7 |
| C. C. Hemond  (115) | USA | 2018 | Cross-sectional | 61  42:19  41 (8.6) | RRMS: 51  SPMS: 5  PPMS: 1  CIS: 4 | 1.6 (1.7) | 8.3 (7.2) | General Electric  3 T | EDSS  T25FW | EDSS correlated with brain parenchymal fraction and normalized brain parenchymal volume. T25FW did not correlate with brain parenchymal fraction and normalized brain parenchymal volume. | 7 |
| F. Khalid  (116) | USA | 2018 | Cohort | 16  16:0  33 (4.1) | RRMS: 15  CIS: 1 | 1 (1) | 7.2 (4.8) | General Electric  1.5 T | EDSS  T25FW | There were no associations between EDSS and following prepregnancy parameters: T2LV, T1LV, number of Gd+ lesions, BPF, cGMF. | 5 |
| O. Khan  (117) | USA | 2016 | Cross-sectional | 39  26:13  56.2 (6.4) | RRMS: 39 | 3.6 (2.5) | 27.3 (4.7) | NR  1.5 T, 3 T | EDSS  FSS | EDSS was significantly correlated with brain volume, GM volume, T1 lesion volume, and T2 lesion volume. | 6 |
| A. Al-Radaideh  (118) | Jordan | 2018 | Case-control | 30  17:13  31.2 (20-53)‡ | RRMS: 30 | 2.6 (2.2) | 46 (14.1) | Siemens  3 T | EDSS  FSS | EDSS correlated negatively with cortical thickness, normalized cortical volume and is positively associated with T2 lesion load. | 6 |
| T. charalambous  (119) | UK | 2018 | Cross-sectional | 122  86:36  48 (11) | RRMS: 58  SPMS: 36  PPMS: 28 | 5.5 (0-8.5)** | 15 (10) | Philips  3 T | EDSS | Higher EDSS score correlated with reduced volumes of NABV, GM, CGM, DGM and NAWM. Higher lesion load was associated with EDSS score. | 8 |
| M. B. D’hooghe  (120) | Belgium | 2018 | Cohort | 470  318:152  46.4 (11.3) | RRMS: 470 | 4.2 (2.1) | 14.5 (10) | General Electric  Siemens  Philips | EDSS | Brain volumetric measurements was a promising tool for prediction of EDSS in MS patients. | 8 |
| D. Jakimovski  (121) | USA | 2018 | Cross-sectional | 71  51:20  52.8 (12.9) | RRMS: 51  SPMS: 20 | 2.5 (1.5-4.5)* | 19.2 (10.7) | General Electric  3 T | EDSS  T25FW | The loss of cortical and nuclei-specific GM volumes contributed greatly to walking disability. | 7 |
| X. F. Du  (122) | China | 2019 | Case-control | 26  15:11  36.6 (10.8) | RRMS: 26 | 3.6 (1.8) | 22 (33) | Siemens  3 T | EDSS | EDSS correlated negatively with fractional amplitude of low-frequency fluctuation in the bilateral supplementary motor area. | 6 |
| S. Grahl  (123) | Germany | 2019 | Case-control | 231  152:79  36 (10.7) | RRMS: 231 | 1.5 (1.2) | 4 (5.2) | Philips, Siemens  3 T | EDSS | White matter lesion volume correlated negatively with thalamic volume and positively with EDSS. | 8 |
| M. A. Abdelhafeez  (124) | Egypt | 2019 | Cross-sectional | 673  488:185  34.4 (8.8) | RRMS: 530  SPMS: 104  PPMS: 19  CIS: 20 | 3 (0-8)** | 8.3 (5.5) | Philips  1.5 T | EDSS | In Egyptian MS patients, infratentorial lesions, confluent brain lesions, and T1 hypointense lesions were associated with disability. | 8 |
| H.N. Beadnall  (125) | Australia | 2019 | Cohort | 102  82:20  30.4 (7.9) | RRMS: 99  SPMS: 2  CIS: 1 | 2 (1.8)* | 7.3 (7.4) | General Electric  3 T | EDSS | EDSS at follow-up was significantly correlated with baseline NBV in MS patients. The baseline EDSS and NBV were negatively correlated, as measured by SIENAX (r= -0.148) and icobrain (r= -0.152) | 8 |
| S. Y. Huang  (126) | USA | 2019 | Cross-sectional | 30  24:6  43.9 (11.2) | RRMS: 23  SPMS: 5  PPMS: 2 | 3 (1.9) | 10.8 (6.7) | Siemens  3 T | EDSS  MSFC | Axon diameter and corpus callosum area correlated with EDSS. Thinning of corpus callosum correlated with poor function on the MSFC. | 7 |
| K. Hänninen  (127) | Finland | 2019 | Cohort | 60  41:19  42.6 (10.9) | RRMS: 24  SPMS: 36 | RRMS:  1.2 (0-4)**  SPMS:  4.5 (2-6.5)** | RRMS:  0.8 (0.7-1.6)‡  SPMS:  19.7 (5.4-35.7)‡ | Siemens  3 T | EDSS | EDSS had significant correlation with BP, GM, WM lesion, putamen, hippocampus, and thalamus volumes at 2-year follow-up. | 7 |
| K. W. Andersen  (128) | Denmark | 2020 | Case-control | 40  NR  46.3 (11.5)*** | RRMS: 26  PPMS: 14 | 3 (0-7)** | NR | Philips  3 T | EDSS | Whole brain lesion load did not correlate with EDSS. microscopic fractional anisotropy negatively correlated with EDSS. | 5 |
| N. Blindenbacher  (129) | Germany | 2020 | Cohort | 66  40:26  34 (8.6) | RRMS: 34  CIS: 32 | 1.5 (0-4.5)** | 1.5 (1.3) | Siemens  3 T | EDSS  MSFC  T25FW  9HPT | The number of susceptibility weighted imaging hypointense core or ring lesions per patient were associated with higher EDSS score and the change of EDSS at follow up. | 5 |
| D. V. Cabrera  (130) | Canada | 2019 | Case-control | 28  18:10  48 (12) | RRMS: 11  SPMS: 9  PPMS: 8 | 4.8 (1.7) | 13 (10) | NR  4.7 T | EDSS | Total lesion volume and lesion count did not correlate with EDSS. | 6 |
| L. Carmisciano  (131) | Italy | 2020 | Cross-sectional | 65  47:18  43.1 (10.7) | RRMS: 46  SPMS: 6  PPMS: 13 | 2 (0-6.5)** | 7 (3-11)* | Philips  3 T | EDSS  T25FW  9HPT | EDSS correlated with T2 lesion volume and normalized brain volume. | 6 |
| V. Karami  (132) | Iran | 2020 | Cross-sectional | 30  16:14  45 (14) | RRMS: 30 | 2.6 (1-4)‡ | 5 (NR) | Siemens  3 T | EDSS | EDSS was associated with volumes of T2 weighted lesion, black holes lesion, and T1 enhancing lesion. | 4 |
| A. Kerbrat  (133) | France | 2020 | Cohort | 290  185:105  42.3 (12.2) | RRMS: 198  SPMS: 39  PPMS: 31  CIS: 22 | 2 (0-8)** | 9 (9.8) | Philips, Siemens  3 T | EDSS  9HPT  T25FW | The risk of EDSS progression during two years follow up correlated with the baseline corticospinal tract spinal cord lesion volume. | 8 |
| E. Colato  (134) | UK | 2021 | Cohort | 988  622:366  46.7 (7.7) | SPMS: 988 | 6 (3-7.5)** | NR | NR  1.5 T, 3 T | EDSS  9HPT | Some of the GM region patterns could be promising measurements to predict disability. | 8 |
| Y. Duan  (135) | China | 2021 | Case-control | 37  23:14  33.8 (11.2) | RRMS: 37 | 2.5 (1-3)* | 3 (1-5.3)* | Philips  3 T | EDSS | Subcortical GM, brain stem, cerebral white matter volume were associated with EDSS. | 7 |
| M. H. de la Cruz  (136) | Italy | 2020 | Cohort | 86  51:35  40.4 (12.4) | RRMS: 75  SPMS: 8  PPMS: 3 | 2 (1-3.5)* | 8.7 (4.4-16.3)* | NR  3 T | EDSS | Follow up EDSS did not correlate with baseline lesion volume, normalized brain volume, and normalized GM volume. | 6 |
| S. Ajitomi  (137) | Japan | 2021 | Cross-sectional | 85  64:21  40.7 (8.9) | RRMS: 69  PMS: 16 | 2 (1-3)* | 9 (5.1-14.9)* | Siemens  1.5 T | EDSS  MSSS | Brain volume, GM volume, third ventricular width, lateral ventricle width, bicaudate ratio, and corpus callosum index correlated with EDSS. | 6 |
| O. Al-iedani  (138) | Australia | 2022 | Cross-sectional | 98  75:23  44 (10) | RRMS: 98 | 2 (1.2) | 8.2 (6.3) | Siemens  3 T | EDSS  MSSS | EDSS correlated with GM volume, total brain volume, and CSF volume. | 6 |
| E. S. Beck  (139) | USA | 2021 | Cross-sectional | 64  40:24  49.5 (11.3) | RRMS: 45  SPMS: 15  PPMS: 4 | RRMS: 1.5 (0-6.5)**  PMS: 6 (2-7.5) | 14.4 (11.2) | NR  3 T, 7 T | EDSS  MSSS  T25FW  9HPT | Cortical lesion, leukocortical lesion, intracortical, subpial, and white matter lesion volume correlated with disability measurements such as EDSS, 9HPT, and T25FW. | 7 |
| T. Akaishi  (140) | Japan | 2020 | Cross-sectional | 42  28:14  26.7 (8.3) | RRMS: 30  SPMS: 10  PPMS: 2 | 2 (1-3.5)* | 12.4 (7.5) | Philips  3 T | EDSS  MSSS | GM atrophy, WM atrophy, and number of T1-hypointense areas were associated with EDSS in MS. | 6 |
| R. Bakshi  (141) | USA | 2020 | Cohort | 200  148:52  39.5 (8.2) | RRMS: 200 | 1.3 (1.1) | 7.4 (6.6) | General Electric  1.5 T | EDSS | T2LV, PBF, and normalized BPV were associated with EDSS at 5-year follow-up. | 8 |
| F. M. Boonstra  (142) | Australia | 2022 | Cohort | 59  51:8  40.6 (10.9) | RRMS: 41  CIS: 18 | 0 (0-1)** | 6.2 (7.5) | Siemens  3 T | EDSS | Brain parenchymal fraction and lesion fraction correlated with EDSS. | 5 |
| R. Brandstadter  (143) | USA | 2020 | Cohort | 184  123:61  34.3 (7.5) | RRMS: 164  CIS: 20 | 1 (0-1.5)* | 2.8 (1.4-4.6)* | Siemens  3 T | EDSS | Correlations were found between performance metrics and MRI markers in MS. | 8 |
| I. Adibi  (144) | Iran | 2021 | Cross-sectional | 20  14:6  37.8 (9.4) | RRMS: 20 | 2.3 (1.5) | 3.8 (1.3) | Siemens  1.5 T | EDSS | In RRMS patients, no correlation was found between the C2/C3 SC-CSA and EDSS. | 5 |
| E. AKIL  (145) | Turkey | 2021 | Case-control | 83  62:21  35.8 (10.5) | RRMS: 83 | 3 (1.7) | 5.3 (5) | Philips  1.5 T | EDSS | A statistically significant positive correlation existed between MRI lesion localization and EDSS score. | 5 |
| I. Brusini  (146) | Sweden | 2022 | Cohort | 631  449:182  41 (11) | RRMS: 450  SPMS: 128  PPMS: 15 | Aera: 2 (1-3)*  Avanto: 1.5 (0-2.8)*  Trio: 2 (1.5-3)*  Testing data: 2.5 (1.5-3.5)* | 4.8 (5.9) | Siemens  NR | EDSS | Both Flair segmentation and freesurfer algorithm indicated that corpus callosum volume correlated with baseline and follow up EDSS. EDSS showed correlation with normalized corpus callosum volume for flair specific (r = -0.13), T1 weighted specific (r = -0.15), flair (r = -0.21), and T1 weighted (r = -0.12) pipeline. | 8 |
| A. Al-Radaideh  (147) | Jordan | 2020 | Case-control | 30  17:13  20- 53^^ | RRMS: 30 | 2.6 (0-6.5)‡ | 46 (14.1)^m^ | Siemens  3 T | EDSS | EDSS was correlated with normalized thalamus volume among RRMS. | 6 |
| N. Bergsland  (148) | USA | 2020 | Cross-sectional | 108  78:30  51.2 (10.4) | RRMS: 68  SPMS: 34  PPMS: 6 | 3 (1.5-5.9)* | 19.3 (10) | General Electric  3 T | EDSS  T25FW  9HPT | EDSS and 9HPT were correlated with thalamic volume and all nuclei groups. Total thalamic and ventral volumes were associated with T25FW. | 8 |
| J. Cao  (149) | China | 2022 | Case-control | 35  24:11  32.9 (10) | NR | 3 (1.3) | 3 (1-5.6)* | General Electric  3 T | EDSS | EDSS correlated with GM volume, brain parenchymal volume, and regional GM volumetry. | 6 |
| W. Guenter  (150) | Poland | 2022 | Case-control | 93  67:26  41 (33-50)* | RRMS: 60  SPMS: 25  PPMS: 8 | 3 (2-4)* | 9 (4-16)* | Philips  1.5 T | EDSS | Third ventricle width, age, and disease duration predict significantly EDSS. EDSS was associated with third ventricular width. | 6 |
| S. M. H. Rúa  (151) | USA | 2021 | Cross-sectional | 103  69:34  41.7 (10.9) | RRMS: 96  SPMS: 7 | 1 (3)* | 8.5 (7.5) | General Electric  3 T | EDSS | EDSS showed inverse associations with cortical thickness and normalized thalamic volume. | 8 |
| M. Ganzetti  (152) | USA, Spain | 2023 | Case-control | 62  42:20  39.7 (7.5) | RRMS: 55  SPMS: 4  PPMS: 3 | 2.5 (1.4) | 9.5 (6.6) | Siemens  3 T | EDSS  9HPT  T25FW | EDSS correlated negatively with putamen, globus pallidus, insula, lateral occipital, and cerebral white matter. | 6 |
| J. S. Graves  (153) | Switzerland | 2022 | Cohort | 69  47:22  39.4 (7.8) | RRMS: 62  SPMS: 4  PPMS: 3 | 2.4 (1.3) | 9.1 (6.5) | NR | EDSS  9HPT  MSIS | Drawing a shape test was utilized to assess the upper extremity dysfunction and was associated with all MS disease measurements. | 7 |
| H. Jang  (154) | USA | 2020 | Case-control | 31  21:10  55.8 (26-75)‡ | NR | NR | NR | NR | EDSS | There was a low correlation between EDSS, both normal appearing white matter, and lesion load. | 5 |
| K. Kocsis  (155) | Hungary | 2021 | Cross-sectional | 79  55:24  42.2 (9.8) | RRMS: 79 | 2 (0-6)** | 12.3 (7.4) | General Electric  3 T | EDSS | EDSS correlated with total lesion volume and normalized brain volume, whereas it was not associated with lesion count. | 5 |
| S. Kolind  (156) | UK | 2012 | Case-control | 17  11:6  51 (35-60)‡ | PPMS: 17 | 5.5 (1.5-6.5)** | 9 (4-19)‡ | Siemens  1.5 T | EDSS | Disability was explained by a special region of cerebral white matter, which showed a method to evaluate the changes of tissue within the brain. EDSS correlated with NAWM MWF variance. | 5 |
| S. Kolind  (157) | UK | 2015 | Case-control | 15  4:11  52 (41-67)‡ | PPMS: 15 | 5 (2.5-6.5)** | 6 (2-17)‡ | General Electric  1.5 T | EDSS  MSFC  9HPT  T25FW | MSFC and 9HPT correlated with ventricular cerebrospinal fluid and T25FW was associated with cervical cord volume. | 5 |
| N. Krajnc  (158) | Austria | 2022 | Cross-sectional | 75  40:35  37 (9) | RRMS: 57  PMS: 18 | 2 (1-3.5)* | 6 (2-12)* | Siemens  3 T | EDSS  MSSS | There were associations between iron rim lesion counts, EDSS, and multiple sclerosis severity scale. | 6 |
| C. Lapucci  (159) | Italy | 2022 | Cross-sectional | 45  30:15  37.4 (6.8) | RRMS: 29  SPMS: 16 | 3.5 (2) | NR | General Electric  3 T | EDSS | T1 and T2 weighted lesion volume correlated with EDSS. | 6 |
| C. Lapucci  (160) | Italy | 2020 | Cross-sectional | 60  35:25  39.2 (7.8) | RRMS: 21  PMS: 16  CIS: 23 | 2.5 (1-5.5)* | NR | General Electric  1.5 T, 3 T | EDSS | EDSS correlated with T1-spin and gradient echo lesion number and volume. The correlation between EDSS, T1-gradient echo hypointense lesion count, and volume were 0.55 and 0.53, respectively. | 5 |
| R. Megna  (161) | Italy | 2018 | retrospective | 241  NR  NR | RRMS: 241 | 2.4 (0.8) | 4.1 (5) | Philips  1.5 T | EDSS | Physical disability showed associations with tissue damage in GM. | 7 |
| H. Larassati  (162) | Indonesia | 2022 | Cross-sectional | 33  27:6  33 (10) | RRMS: 24  SPMS: 9 | 3 (1-8)** | NR | General Electric  1.5 T | EDSS | There were associations between fractional anisotropy and apparent diffusion coefficient of normal appearing white matter with EDSS in MS patients. | 5 |
| L. E. Lee  (163) | Canada | 2021 | Case-control | 65  45:20  RRMS:  44 (26-61)‡  SPMS:  57 (48-65)‡ | RRMS: 35  SPMS: 30 | RRMS:  2.5 (1-6)**  SPMS:  4 (2-7.5)** | RRMS:  11 (0.3-48)‡  SPMS:  22 (1-43)‡ | Philips  3 T | EDSS  9HPT  T25FW | EDSS correlated with myelin heterogeneity index in global white matter, dorsal column, and lateral funiculi. 9HPT was associated with myelin heterogeneity index in the whole cervical cord, global white matter, dorsal column, and lateral funiculi. | 5 |
| A. L. Dopico  (164) | USA | 2021 | Cross-sectional | 45  28:17  48.7 (22.6-63.5)** | RRMS: 33  SPMS: 4  PPMS: 8 | 3 (1-6.5)** | 8.3 (0.7-38.4)** | Philips  7 T | EDSS  T25FW  9HPT | EDSS and T25FW correlated negatively with cortical thickness and cortical GM fraction. 9HPT was associated with brain parenchyma fraction, white matter lesion fraction, cortical GM fraction, and cortical thickness. | 6 |
| H. Liu  (165) | China | 2016 | Case-control | 18  13:5  35 (10.6) | RRMS: 18 | 3.9 (1.8) | 2.3 (3.2) | General Electric  3 T | EDSS | EDSS indicated a negative association with the beta value of caudate head. | 6 |
| Y. Liu  (166) | China | 2015 | Cross-sectional | 24  16:8  35.2 (23-52)‡ | NR | 3.7 (1-7.5)** | 1.7 (0.2-5.8)** | General Electric  3 T | EDSS | There was a correlation between amplitude of low-frequency fluctuation and EDSS in the right supramarginal gyrus. | 6 |
| S. Llufriu  (167) | Spain | 2012 | Case-control | 21  12:9  37.2 (6.9) | RRMS: 21 | 2 (0-6)** | 9.5 (5.3) | Siemens  3 T | EDSS  MSFC | Corpus callosum damage resulted in physical disability and cognitive impairment via disconnection mechanism. | 6 |
| N. A. Losseff  (168) | UK | 1996 | Case-control | 60  34:26  41 (21-63)‡ | RRMS: 15  SPMS: 15  PPMS: 15  BMS: 15 | RRMS:  2.5 (1-6)**  SPMS:  7 (4.5-8.5)**  PPMS:  4 (2-7.5)**  Benign:  2 (1-3)** | RRMS:  4.1 (1-10)‡  SPMS:  15.9 (4-30)‡  PPMS:  10.4 (2.5-24)‡  Benign:  14 (10-20)‡ | General Electric  1.5 T | EDSS | Spinal cord area indicated the associations with EDSS and functional system subscores of EDSS. | 5 |
| P. Lu  (169) | Switzerland | 2021 | Cross-sectional | 123  71:52  44.7 (14) | RRMS: 84  PMS: 39 | 2.5 (0-8)** | NR | Siemens  3 T | EDSS | The mixture of neurite density index and the isotropic and the intra-axonal compartment from the microstructural Bayesian approach revealed the association with EDSS. | 6 |
| H. Lundell  (170) | Denmark | 2016 | Case-control | 54  30:24  RRMS:  40 (25-59)‡  SPMS:  48 (30-62)‡  PPMS:  40 (27-55)‡ | RRMS: 22  SPMS: 23  PPMS: 9 | RRMS:  3.5 (0-6.5)‡  SPMS:  5.5 (3.5-6.5)‡  PPMS:  4 (3.5-6.5)‡ | RRMS:  9 (3-27)‡  SPMS:  15 (6-43)‡  PPMS:  3 (2-10)‡ | Siemens  3 T | EDSS  MSIS | Among all MS patients, EDSS and MSIS were associated negatively with cross-sectional area spinal cord. | 5 |
| G. J. L. a  Nijeholt  (171) | Netherlands | 1999 | Case-control | 65  29:36  45 (24-66)** | RRMS: 14  SPMS: 34  PPMS: 17 | 4 (0-8)** | 6 (1-29)** | Siemens  1 T | EDSS | Spinal cord cross-sectional area and spinal cord MTR were associated negatively with EDSS. | 5 |
| M. Lyksborg  (172) | Denmark | 2014 | Case-control | 34  22:12  43.7 (11.1) | RRMS: 19  SPMS: 15 | 4 (1.7) | 15.2 (10) | Siemens  3 T | EDSS | Regional anatomical connectivity mapping indicated a correlation with EDSS.   \|  \| \| --- \| | 6 |
| S. Mamoei  (173) | Denmark | 2021 | Cohort | 49  26:23  51.2 (7.8) | NR | 4.9 (1.3) | 16.4 (7.1) | Phillips, Siemens  1.5 T, 3 T | EDSS  T25FW  9HPT | There was a correlation between disability measurements such as T25FW and EDSS and central motor conduction times. | 7 |
| M. Margoni  (174) | Italy | 2020 | Cross-sectional | 15  12:3  14.9 (2.2) | RRMS: 15 | 1.5 (1-2.2)* | 0.7 (1.2) | Philips  3 T | EDSS | Cerebellar GM lesion, as a prevalent marker in pediatric multiple sclerosis, did not correlate with physical and cognitive disability. | 5 |
| C. E. Markowitz  (175) | USA | 2009 | Cohort | 16  15:1  42.5 (11.7) | RRMS: 16 | 2.6 (1) | 4.3 (4.5) | Siemens  1.5 T | EDSS | EDSS correlated with proton density brain parenchyma histogram peak location, proton density GM histogram peak location, and proton density pure white matter histogram peak height. | 5 |
| D. Mistri  (176) | Italy | 2022 | Cross-sectional | 106  65:41  34.4 (26.1-44.1)* | RRMS: 106 | 1.5 (1-2)* | 10.3 (6.4) | Philips  3 T | EDSS  9HPT  T25FW | The z-scores of T25FW and 9HPT correlated negatively with T2 and T1 lesion volume positively were associated with normalized volume of brain, GM, white matter, and deep GM. | 7 |
| A. B. Mohamed  (177) | Egypt | 2022 | Cross-sectional | 80  44:36  33.2 (8.6) | RRMS: 80 | 2.4 (1.2) | 4.6 (4.6) | Philips  1.5 T | EDSS | Non‑conventional quantitative MRI techniques should be utilized to identify early disability in MS. EDSS correlated with apparent diffusion coefficient. | 5 |
| G. Nair  (178) | USA | 2013 | Case-control | 39  21:18  49 (12) | RRMS: 14  SPMS: 10  PPMS: 15 | 1-7^^ | 13 (11) | Siemens  3 T | EDSS | EDSS was associated better with cord lesion volume through T1-MPRAGE sequence compared to other sequences. | 5 |
| Y. Nakamura  (179) | Japan | 2018 | Cross-sectional | 341  244:97  36.1 (9.2) | RRMS: 341 | 2.2 (1.4) | 7.7 (7) | 1.5 T | EDSS | EDSS correlated with T2 lesion volume in Japanese MS patients. In both Caucasian and Japanese patients, normalized volume of brain, cortical GM, and deep GM were associated with EDSS. Normalized thalamic volume was negatively associated with EDSS. | 8 |
| V. Nazarov  (180) | Russia | 2019 | Cohort | 92  60:32  32.5 (18-65)** | NR | 2 (0-6.5)** | 1.8 (0-39.3)** | NR  1.5 T | EDSS | No correlation was found between EDSS and brain volume changes. | 6 |
| M. Nylund  (181) | Finland | 2021 | Cross-sectional | 91  70:21  44.9 (9.6) | RRMS: 67  SPMS: 24 | 3 (2-3.5)* | 12.1 (7.2-15.8)* | Philips  3T | EDSS  MSSS | Worsen disability correlated with lower volumes of normal appearing white matter, brain and higher T1 lesion load. | 5 |
| K. Okada  (182) | Japan | 2019 | Cross-sectional | 40  32:8  38.5 (19-64)** | RRMS: 40 | 1 (0-3)** | 3.5 (1-24)** | General Electric  3 T | EDSS | There was a positive correlation between EDSS and third ventricle width. | 5 |
| L. Orbach  (183) | Israel | 2017 | Cross-sectional | 271  187:84  33.1 (0.4) | RRMS: 271 | 1.6 (0.1) | 5 (0.2) | General Electric  3 T | EDSS | EDSS correlated negatively with cortical thickness. Markers such as cortical GM atrophy may be an useful marker to manage the MS patients. | 8 |
| C. Oreja-Guevara  (184) | Italy | 2006 | Cohort | 22  15:7  36.6 (25-50)‡ | RRMS: 22 | 1.2 (0-3.5)** | 10.4 (1-23)‡ | NR  1.5 T | EDSS | EDSS was associated with GM and white matter magnetization transfer ratio. There was a correlation between normal appearing white matter magnetization transfer ratio and EDSS changes during follow up. | 6 |
| R. Ouellette  (185) | Sweden | 2020 | Cohort | 71  49:22  40.9 (10.2) | RRMS: 53  SPMS: 15  PPMS: 3 | 2 (2) | 12.2 (8.4) | Siemens  3 T | EDSS | EDSS score at two years follow up was associated with normal appearing white matter volume, whole brain myelin content, and the lesion volume fraction. | 5 |
| D. Paling  (186) | UK | 2013 | Cross-sectional | 70  41:29  45.8 (11.2) | RRMS: 27  SPMS: 23  PPMS: 20 | 5.5 (0-8.5)** | 14 (9.4) | Phillips  3 T | EDSS | EDSS correlated with GM fraction but was not associated with WM fraction. MSFC did not correlate with WM fraction. | 7 |
| D.W. Paty  (187) | Canada | 1993 | Cohort | 327  229:98  1.6 MIU:  35.3 (0.7)  8 MIU:  35.2 (0.6)  placebo:  36 (0.6) | RRMS: 327 | 1.6 MIU:  2.9 (0.1)  8 MIU:  3 (0.1)  placebo:  2.8 (0.1) | 1.6 MIU:  4.7 (0.4)  8 MIU:  4.7 (0.4)  placebo:  3.9 (0.3) | General  Electric, Fonar, Philips, Picker, and Siemens  0.15-1.5 T | EDSS | There was a correlation between baseline and follow up lesion area with baseline and follow up EDSS. | 7 |
| C. A. Pérez  (188) | USA | 2020 | Cohort | 188  148:40  36.3 (11.3) | RRMS: 160  SPMS: 22  PPMS: 6 | Hispanic:  2 (NR)**  Caucasian:  1 (NR)** | 13.4 (8.5) | Philips  3 T | EDSS | Normalized thalamic volume could be used as a predictor of disability worsening. | 8 |
| M. Petracca  (189) | USA | 2016 | Cross-sectional | 19  11:8  40 (11.2) | RRMS: 19 | 2 (0-5.5)** | 9.1 (7.4) | Siemens  3 T | EDSS | There was an inverse correlation between EDSS and global GM intracellular sodium volume fraction. | 7 |
| A. Pietroboni  (190) | Italy | 2023 | Cohort | 59  21:38  36 (28-46)* | RRMS: 44  PPMS: 9  CIS: 6 | 2 (1-2.5)* | 0.08 (0-1.2)* | Philips  3 T | EDSS | The changes of EDSS score were associated with NAWM-QSM and NAWM-volume fraction at baseline. NAWM-QSM could predict the change of EDSS score. | 5 |
| M. Platten  (191) | Sweden | 2021 | Cohort | 353  272;81  39 (11) | RRMS: 194  SPMS: 92  PPMS: 18  NR: 49 | 2 (1-3)* | 1.1 (4.1) | Siemens  1.5 T | EDSS | Increasing disability correlated with more angled corpus callosum and corpus callosum became thinner alongside with the increase of physical disability. | 8 |
| S. Ramanathan  (192) | Australia | 2013 | Cohort | 63  44:19  40 (30.8-50.8)* | RRMS: 53  SPMS: 10 | 1.5 (1-3.5)* | 3 (2-6)* | General Electric  1.5 T | EDSS | Follow up EDSS correlated weakly with brain and spinal lesion load. | 5 |
| H. Rasoanandrianina  (193) | France | 2020 | Case-control | 19  11:8  46 (26-70)** | RRMS: 13  SPMS: 6 | 2.5 (1-7)** | 9.7 (4-31)** | Siemens  3 T | EDSS  9HPT  T25FW | There was a correlation between brain lesion volume and T25FW. | 5 |
| R. Righart  (194) | Germany | 2017 | Cohort | 168  115:53  30.8 (19.1-39.2)‡ | RRMS: 140  CIS: 28 | 1 (0-3.5)** | 2.6 (0.1-15.1)** | Philips  3 T | EDSS  MSFC  T25FW  9HPT | 9HPT and EDSS were associated negatively with T1/T2 weighted ratio in the posterior cingulate. | 8 |
| M.A. Rocca  (195) | Italy | 2011 | Case-control | 333  210:123  41.8 (19-76)‡ | RRMS:101  SPMS: 79  PPMS:75  BMS: 58  CIS: 20 | RRMS:  3 (0-6.5)**  SPMS:  6 (2.5-8)**  PPMS:  5.5 (2-8)**  BMS:  2 (0-3)**  CIS:  2 (0-4)** | 13.6 (0.01-48)‡ | NR  1.5 T | EDSS | Brain T2 lesion volume, number of cervical lesions, and normalized cervical cord cross sectional area correlated with EDSS. | 9 |
| M. Rovaris  (196) | Italy | 2002 | Cohort | 239  NR  34 (7.4) | RRMS: 239 | 2.3 (1.1) | 8.1 (5.5) | NR | EDSS | Changes of EDSS score did not correlate with the number of new T2 lesions accumulated during the nine month follow up. Changes of EDSS score were associated with baseline T1 and T2 lesion volume. | 7 |
| S. Ruggieri  (197) | Italy | 2020 | Case-control | 49  36:13  35 (8.1) | RRMS: 34  SPMS: 15 | 2.5 (1-5.5)** | 7.8 (6.4-NR)‡ | Siemens  3 T | EDSS  25FW | EDSS revealed associations with volumes of white matter, GM, and brain lesions. Higher T25FW and EDSS correlated with reduction in cerebellar lobular volumes. | 7 |
| M. Sailer  (198) | UK | 2001 | Cohort | 29  16:13  38.2 (6.6) | RRMS: 13  SPMS: 16 | 6 (2-7)** | 8.8 (6.3) | General Electric | EDSS | The change of T1 lesion volume was associated more strongly with EDSS change than the change of T2 lesion volume. | 5 |
| A. Saini  (199) | Canada | 2021 | Cross-sectional | 532  367:165  49.4 (13.1) | RRMS: 334  SPMS: 118  PPMS: 61  PRMS: 10  CIS: 9 | 3.2 (1.5-6)* | 16.1 (12.1) | NR | EDSS | EDSS indicated an association with the number of spinal cord lesions. | 8 |
| E. Sbardella  (200) | Italy | 2013 | Case-control | 36  26:10  34 (8) | RRMS: 36 | 2.5 (1-4.5)** | 7.4 (6.1) | Siemens  3 T | EDSS  9HPT  T25FW | White matter tract damage and GM atrophy were involved in upper limb motion dysfunction. EDSS and T25FW did not correlate with lesion volume, WM/ICV, and GM/ICV. | 6 |
| P. Schmalbrock  (201) | USA | 2015 | Cross-sectional | 29  28:1  43.4 (9.7) | RRMS: 29 | 3 (1.5) | 6.5 (5.5) | Philips  7 T | EDSS | Quantitative susceptibility mapping and R2 were markers for tissue changes in the basal ganglia in MS patients. | 4 |
| M. M. Schoonheim  (202) | Austria | 2021 | Case-control | 502  340:172  43.8 (12.1) | RRMS: 349  SPMS: 53  PPMS: 41  CIS: 61 | 2.4 (0-8)** | 12.1 (8.7) | Siemens  3 T | EDSS | EDSS correlated with T2 lesion volume, and normalized volumes of thalamus and cortex. | 8 |
| K. Schreiber  (203) | Denmark | 2001 | Cross-sectional | 119  71:48   \| 42.9 (4.5) \| \| --- \| | NR | 6 (1-9.5)** | 13.8 (5-24)‡ | Fonar  0.3 T | EDSS | EDSS correlated with total lesion area, ventricle brain ratio, and corpus callosum brain ratio. | 8 |
| J. Sharma  (204) | USA | 2003 | Cross-sectional | 52  38:14  42.8 (8.5) | RRMS: 43  SPMS: 9 | 3.4 (1.9) | 11.6 (8.7) | Phillips  1.5 T | EDSS | EDSS correlated with brain parenchymal fraction at 2D and 3D semiautomated and automated MRI segmentation. The correlation between BPF and EDSS for 3D semiautomated method was -0.47 while this correlation for 3D automated was -0.33. | 6 |
| K. Shinoda  (205) | Japan | 2017 | Cross-sectional | 92  72:20  43.3 (13) | RRMS: 74  SPMS: 18 | 2.6 (2.1) | 13.7 (9.6) | Philips  3 T | EDSS  MSSS | The number of intracortical lesions, leukocortical lesions, and total number of cortical lesions correlated with EDSS. | 5 |
| A. C. H. Silemek  (206) | Germany | 2020 | Cross-sectional | 33  20:13  40.9 (9.7) | RRMS: 33 | 2 (0-4)** | 10.4 (8.1) | Siemens  3 T | EDSS  T25FW  9HPT | T25FW and EDSS correlated with both GM and brain volumes. | 5 |
| J. H. Simon  (207) | USA | 1997 | Cohort | 301  221:80  Interferon beta: 36.7 (0.57)‡‡  placebo: 36.9 (0.64)‡‡ | RRMS: 301 | Interferon beta: 2.4 (0.06)‡‡  placebo: 2.3 (0.07)‡‡ | Interferon beta: 6.6 (0.46)‡‡  placebo: 6.4 (0.49)‡‡ | NR  1 T, 1.5 T | EDSS | Baseline EDSS correlated with T2 lesion volume positively but was not associated with the number of GD lesions. The change in EDSS did not correlate with change in T2 lesion volume, GD lesion number, and at year 2 follow-up. | 7 |
| B. Spano  (208) | Italy | 2010 | Cross-sectional | 10  8:2  44.5 (6.5) | BMS: 10 | 1.7 (1-3)** | 17.1 (4.5) | Siemens  3 T | EDSS  MSFC | EDSS score correlated with the GM of the left motor cortex. MSFC score was associated with the right motor cortex. | 5 |
| J.M. Stankiewicz  (209) | USA | 2009 | Cross-sectional | 32  NR  42 (21-54)** | RRMS: 26  SPMS: 4  PPMS: 1  CIS: 1 | RRMS:  1 (0-2.5)**  SPMS:  6 (6-6.5)**  PPMS:  NR  CIS:  NR | 5.8 (0.2 -29)** | General Electric, Philips  1.5 T, 3 T | EDSS  T25FW  FSS | There was a weak correlation between spinal lesions and disability. The correlation between EDSS and cervical T2 lesion load was 0.36 at 1.5 T. | 5 |
| V.L. Stevenson  (210) | UK | 1998 | Cohort | 28  NR  45.1 (27-65)‡ | RRMS: 6  SPMS: 6  PPMS: 12  BMS: 4 | RRMS:  3.2 (1.5-6.5)**  SPMS:  7.2 (6-8)**  PPMS:  5.7 (3-8.5)**  BMS:  2.2 (2-3)** | RRMS:  5.6 (2-9)‡  SPMS:  19.3 (17-24)‡  PPMS:  10.9 (4-22)‡  BMS:  17.3 (13-22)‡ | General Electric  1.5 T | EDSS | There was a negative correlation between EDSS score and spinal cord area. Serial evaluation of spinal cord atrophy was a useful marker to examine treatment efficacy. | 5 |
| M. Strik  (211) | Netherlands | 2020 | Case-control | 222  165:57  45.3 (10.4) | RRMS: 189  SPMS: 33 | High disability:  6.5 (6-8)**  Low disability:  2.5 (0-3.5)** | 13.6 (8.2) | General Electric  3 T | EDSS  FSS | Local functional network efficiency of the of the left primary somatosensory cortex correlated with EDSS, and pyramidal, brainstem, and sensory FSS. | 7 |
| M. Strik  (212) | Australia | 2021 | Case-control | 28  23:5  41.7 (10) | RRMS: 28 | 1.5 (1-1.5)* | 6.5 (3.9) | Siemens  7 T | EDSS  FSS | There was an association between lower limb motor dysfunction and the changes of neuronal activity in sensorimotor regions. | 7 |
| S. Sugijono  (213) | Indonesia | 2020 | Cross-sectional | 30  24:6  32 (20-61)** | RRMS: 22  SPMS: 8 | 3.5 (0-8)** | NR | General Electric  1.5 T | EDSS | There was an inverse correlation between corpus callosum index and EDSS score. | 5 |
| C. Thaler  (214) | Germany | 2015 | Cross-sectional | 40  26:14  36.9 (10.6) | RRMS: 37  SPMS: 2  PPMS: 1 | 2.2 (1.5) | 6.8 (6.5) | Siemens  3 T | EDSS  MSFC | EDSS correlated with lesion number. There were associations between lesion volume, EDSS, and MSFC. | 5 |
| A. Theodorsdottir  (215) | Denmark | 2022 | Cross-sectional | 68  44:24  54.4 (8.1) | SPMS: 68 | 4.9 (1.4) | 23.1 (8.3) | Philips  3 T | EDSS  9HPT  MSIS  T25FW | MSIS indicated associations with atrophy of CNS regions and functional motor tests. In comparison with EDSS, MSIS was more sensitive to sensory and cerebellar functions. | 6 |
| S. Tommasin  (216) | Italy | 2021 | Cohort | 163  104:59  39.6 (10.2) | RRMS: 122  PMS: 41 | 3 (0-7.5)** | 9.9 (8) | Siemens  3 T | EDSS | Thalamic and T2 lesion volume were vital predictors of disability progression. | 8 |
| S. Tommasin  (217) | Italy | 2020 | Case-control | 119  91:28  38.8 (10) | RRMS: 91  SPMS: 28 | 2 (0-7.5)** | 8.6 (8) | Siemens  3 T | EDSS  9HPT  25FW | EDSS correlated with the degree of centrality of the executive control network and default mode network. | 8 |
| C. A. Treaba  (218) | USA | 2019 | Cohort | 31  23:8  40.8 (9.7) | RRMS: 20  SPMS: 11 | RRMS:  2 (1-4)**  SPMS:  5 (2-6.5)** | 10.9 (9.8) | Siemens  3 T, 7 T | EDSS | EDSS changes correlated with the number and volume of total cortical lesion, intracortical lesion, and leukocortical lesion. | 6 |
| C. A. Treaba  (219) | USA | 2021 | Case-control | 102  77:25  42 (9) | RRMS: 76  SPMS: 26 | 2.2 (0-8)** | NR | Siemens  3 T, 7 T | EDSS | Cortical thickness, volumes of cortical lesion, and white matter lesion correlated with EDSS. | 8 |
| L. Truyen  (220) | Netherlands | 1996 | Cohort | 46  28:18  35 (21-53)** | RRMS: 29  SPMS: 17 | RRMS:  2 (0-6)**  SPMS:  2.5 (1.5-6)** | 7 (1-24)** | Technicare  0.6 T | EDSS | EDSS correlated with T1/T2 lesion load ratio and T1 lesion load at end point follow up. | 6 |
| J. K. Udupa  (221) | USA | 2001 | Cross-sectional | 100  NR  NR | NR | NR | NR | NR | EDSS | There was no association between T2 lesion volume and EDSS. Among RRMS patients, peak height normalized by the volume of the brain parenchyma correlated with EDSS. | 4 |
| M. Ukkonen  (222) | Finland | 2003 | Case-control | 28  14:14  51 (9) | PPMS: 28 | 4.9 (2-8)‡ | 12 (9) | General Electric  1.5 T | EDSS  AI | There was evidence of atrophy in the spinal cord and brain among PPMS patients which were associated with disability. | 5 |
| L. Vaithianathar  (223) | UK | 2002 | Case-control | 25  16:9  40 (35.5-44.5)* | RRMS: 25 | 3 (2-3.5)* | 6.4 (3-8.5)* | Siemens  1.5 T | EDSS | T1 measurements in the pyramidal tracts was a promising tool for monitoring the disability and disease progression. Total lesion volume did not correlate with EDSS. Pyramidal tract T1 was associated with FSS and EDSS. | 5 |
| A. M. Valcarcel  (224) | USA | 2018 | Cohort | 40  28:12  50.4 (9.9) | RRMS: 32  SPMS: 8 | 2.3 (1.6) | 14.5 (4.6) | Siemens  3 T | EDSS  T25FW | Using methods for Inter-Modal Segmentation Analysis, the correlation between EDSS and T1 lesion volume was 0.34 and the association between T1/T2 lesion volume and EDSS was 0.3. Using method for Inter-Modal Segmentation Analysis, the correlation between T2 lesion volume and EDSS was 0.3. T25FW showed correlation with T1 lesion volume (r = -0.05) and T2 lesion volume (r = -0.05) in using the method for Inter-Modal Segmentation Analysis. | 6 |
| M. A. A. van Walderveen  (225) | Netherlands | 1995 | Cohort | 48  36:12  33.2 (21-62)‡ | RRMS: 33  SPMS: 14  PPMS: 1 | 2.2 (NR) | 4 (0-25)* | Technicare  0.6 T | EDSS | An increase in disability correlated positively with increases in MRI lesion volume. Relapses count correlated with the number of active lesions. | 5 |
| M.A.A. van Walderveen  (226) | Netherlands | 2001 | Cross-sectional | 138  93:45  RRMS:  36 (22-58)**  SPMS: 43 (30-65)**  PPMS:  48 (20-69)** | RRMS: 52  SPMS: 44  PPMS: 42 | RRMS:  1 (0-7)**  SPMS:  5 (1.5-7.5)**  PPMS:  6 (2-7.5)** | RRMS:  5 (0-30)**  SPMS: 10 (1-40)**  PPMS:  7 (1-35)** | Siemens  1 T | EDSS | EDSS showed associations with T1 and T2 lesion volumes. | 7 |
| M. Vaneckova  (227) | Czech Republic | 2022 | Cross-sectional | 99  69:30  41.3 (11.8) | Early MS: 47  PMS: 52 | Early MS:  2 (1-3.5)**  PMS:  5.5 (3.5-6.5)** | 10.4 (11.1) | Siemens  3 T | EDSS | Among all MS patients, the periventricular gradient in NAWM was a useful marker to examine the disease activity. EDSS did not correlate with lesion volume and count. | 7 |
| J Versijpt  (228) | Netherlands | 2005 | Case-control | 22  13:9  42.6 (11) | RRMS: 13  SPMS: 7  PPMS: 2 | 4 (2) | 9.3 (8.3) | Siemens  1.5 T | EDSS | Brain atrophy was related to normal appearing white matter and T2 lesional inflammation as explained by microglial activation. Disability indicated the association with brain atrophy. | 5 |
| A. Vidal-Jordana  (229) | Spain | 2020 | Cross-sectional | 90  61:29  38 (10.2) | RRMS: 90 | 2 (1.5)* | 8.1 (8.8) | NR | EDSS | There were inverse correlations between EDSS, BPF, GMF, and SCA and positive association between EDSS and lesion volume. | 7 |
| R. R. Voskuhl  (230) | Germany | 2020 | Cross-sectional | 89  52:37  41.5 (12) | RRMS: 79  SPMS: 10 | 2.3 (1.4) | 8.5 (7.3) | Siemens  3 T | EDSS  9HPT  T25FW | There were inverse associations between 9HPT scores, thalamus, and putamen volumes. Among female patients, 9HPT scores did not correlate with thalamus and putamen volumes. | 7 |
| K. Weier  (231) | Switzerland | 2012 | Cross-sectional | 202  140:62  44 (20-67)‡ | RRMS: 147  SPMS: 39  PPMS: 10  CIS: 6 | 3 (0-7.5)‡ | 14 (0-48)‡ | Siemens  1.5 T | EDSS | Number of focal lesions, signs of atrophy correlated with EDSS score. | 7 |
| J. Wen  (232) | USA | 2015 | Cross-sectional | 29  22:7  54.4 (10.4) | RRMS: 10  SPMS: 9  PPMS: 10 | 4.5 (1.7) | NR | Siemens  3 T | EDSS  9HPT  T25FW | T25FW revealed inverse associations with ΔNBV and age-adjusted GM volume. T25FW, EDSS, and 9HPT did not correlate with cortical GM ΔR2*. | 5 |
| N C. Wetter  (233) | USA | 2015 | Cross-sectional | 52  41:11  51 (8.4) | RRMS: 40  SPMS: 7  PPMS: 3  NR: 2 | 5.5 (2.8) | 46 (12.1) | Siemens  3 T | EDSS  T25FW | Correlation was revealed between all MRI parameters and disability measurements except between GM volume, EDSS, and T25FW. | 7 |
| B. Xiang  (234) | USA | 2018 | Case-control | 44  30:14  54.6 (10.5) | RRMS: 15  SPMS: 16  PPMS: 13 | 4.6 (1.9) | 17.7 (9.3) | Siemens  3 T | EDSS  T25FW  9HPT | Spinal cord cross sectional area was considered as a useful radiomarker to monitor the changes in motor functions. EDSS and T25FW correlated with ΔCSA at C1. | 4 |
| B. Xiang  (235) | USA | 2022 | Cohort | 33  25:8  56.2 (7.4) | RRMS: 11  SPMS: 18  PPMS: 4 | 4.6 (2.1) | NR | Siemens  3 T | EDSS  T25FW  9HPT | Macromolecular proton fraction in lesions showed associations with disability measurements such as EDSS, T25FW, and 9HPT. Lesion volume did not correlate with disability values. | 5 |
| Ö. Yaldizli  (236) | Switzerland | 2013 | Cross-sectional | 113  75:38  47.8 (10.7) | RRMS: 84  SPMS: 22  PPMS: 5  CIS: 2 | 3.2 (1.7) | 20.5 (9.1) | Siemens  1.5 T | EDSS | Corpus callosum index was not associated with disability. | 8 |
| V. Yarnykh  (237) | USA | 2015 | Cross-sectional | 30  19:11  51.3 (10) | RRMS: 18  SPMS: 12 | 4.4 (2.4) | 10.2 (7.1) | Philips  3 T | EDSS  MSFC  9HPT  T25FW | Macromolecular proton fraction in all normal appearing tissue correlated with disability measurements such as EDSS and MSFC. | 5 |
| V.L. Yarnykh  (238) | USA | 2018 | Case-control | 30  19:11  51.3 (10) | RRMS: 18  SPMS: 12 | 4.4 (2.4) | 10.2 (7.1) | Philips  3 T | EDSS  9HPT  T25FW  MSFC | Subcortical GM demyelination showed associations with disability and disease subtypes. | 5 |
| F. Yousuf  (239) | USA | 2016 | Cross-sectional | 51  35:16  40.7 (9.1) | RRMS: 43  SPMS: 4  PPMS: 1  CIS: 3 | 1.6 (1.7) | 8.3 (7) | General Electric  3 T | EDSS  T25FW | EDSS showed weak to moderate associations with brain parenchymal fraction and upper cervical cord area. | 6 |
| A. Zacharzewska-Gondek  (240) | Poland | 2018 | Case-control | 66  48:18  37.5 (19-61)‡ | RRMS: 66 | 2.2 (1-7)‡ | 2.2 (0.08-17.5)‡ | General Electric  1.5 T | EDSS | There was a positive correlation between EDSS and lesion load. | 5 |
| L. Zhang  (241) | China | 2020 | Case-control | 18  12:6  31.2 (22-42)‡ | RRMS: 18 | NR | NR | General Electric  3 T | EDSS | In comparison with MTR, inhomogeneous magnetization transfer correlated more closely with EDSS and could be used as a useful marker in MS patients. | 5 |
| Y. Zhang  (242) | Canada | 2006 | Case-control | 17  15:2  42 (28-58)‡ | RRMS: 16  NR: 1 | 3.5 (1.5-7.5)** | 5 (1-24)** | General Electric  1.5 T, 3 T | EDSS | EDSS correlated with signal intensity in the caudate nucleus and globus pallidus. | 4 |
| F. Zhou  (243) | China | 2014 | Case-control | 23  16:7  42.1 (20-58)‡ | RRMS: 23 | 1.5 (1-2.5)** | 2.1 (0.12-12.5)‡ | Siemens  3 T | EDSS | There was no correlation between EDSS and thalamic amplitude of low frequency fluctuation measures. | 5 |
| Q. Zhu  (244) | China | 2022 | Case-control | 48  31:17  33.1 (9.2) | RRMS: 48 | 2.2 (1.3) | 5.4 (5.2) | Siemens  3 T | EDSS | T1 and T2 lesion volumes showed no associations with EDSS. | 6 |
| R. Zivadinov  (245) | Italy | 2000 | Cross-sectional | 63  43:20  35.4 (9.1) | RRMS: 63 | 1.5 (0-5)** | 5.8 (3.3) | NR  1.5 T | EDSS | Low disability score was correlated directly with the severity of diffuse brain damage. Also, EDSS correlated with BPF. | 6 |
| J. Zurawski  (246) | USA | 2019 | Cross-sectional | 62  42:20  46.3 (8.8) | RRMS: 53  SPMS: 7  CIS: 2 | 1.9 (1.5) | 13.5 (7.2) | Siemens  3 T | EDSS  T25FW  9HPT | Cervical cord atrophy correlated more closely with disability markers in comparison with total cervical or brain lesion load. | 6 |
| G. O. Nygaard  (247) | Norway | 2015 | Case-control | 44  32:12  35.1 (7.3) | RRMS: 44 | 1.8 (0.8) | 1.3 (0.9) | NR | EDSS  9HPT | 9HPT showed a correlation with white matter lesion volume. | 6 |
| H. ERTAŞOĞLU TOYDEMİR  (248) | Turkey | 2015 | Case-control | 23  17:6  30.5 (6.5) | RRMS: 19  PPMS: 1  CIS: 3 | 1.3 (0-3.5)‡ | 4.5 (0.5) | General Electric  1.5 T | EDSS | EDSS and CC areas were significantly correlated negatively in the MS group. | 5 |
| M. S. Pitombeira  (249) | Brazil | 2022 | Cross-sectional | 47  31:16  41.3 (10.6) | RRMS: 28  SPMS: 8  PPMS: 11 | 3.5 (1-7.5)** | 10.4 (6.3) | NR | EDSS  9HPT  T25FW | In both white and GM, loss of myelin in T2 lesion and structures near to ventricles correlated with disability. | 7 |
| T. Hayton  (250) | UK | 2012 | Cohort | 118  85:33  50.6 (7) | SPMS: 118 | 6 (4-7.5)** | 20.1 (3-41)‡ | General Electric  1.5 T | EDSS  MSIS | T1/T2 lesion volume ratio and central cerebral volume correlated with the physical component of MSIS. | 7 |
| C M. Griffin  (251) | UK | 2001 | Case-control | 28  19:9  35.5 (NR) | RRMS: 28 | 1 (0-3)** | 1.7 (1-3)** | General Electric  1.5 T | EDSS  MSFC  9HPT  T25FW | No correlations were found between disability measurements such as EDSS and MSFC and normal appearing white and GM volume ratio. | 5 |
| J. Dehmeshki  (252) | UK | 2001 | Case-control | 42  NR  NR | RRMS: 11  SPMS: 11  PPMS: 10  BMS: 10 | NR | NR | General Electric  1.5 T | EDSS | EDSS showed an association with MTR histogram parameters such as peak location and peak height. | 4 |
| N.F. Kalkers  (253) | Netherlands | 2001 | Case-control | 79  51:28  46.5 (10.7) | RRMS: 16  SPMS: 26  PPMS: 26  BMS: 11 | 4.3 (2.1) | 11.5 (7) | Siemens  1 T | EDSS | MTR histogram parameters such as peak height and peak location correlated negatively with EDSS. | 5 |
| G. R. Davies  (254) | UK | 2005 | Cohort | 23  19:4  37 (27-55)‡ | RRMS: 23 | 1 (0-3)** | 1.9 (0.5-3.7)** | General Electric  1.5 T | EDSS  9HPT  MSFC  T25FW | The rate of thalamic MTR change did not correlate with EDSS at year two follow up. | 6 |
| C. Laule  (255) | Canada | 2010 | Case-control | 24  8:16  51 (32-66)‡ | PPMS: 24 | 6 (3-6.5)** | 8 (2-20)‡ | General Electric  1.5 T | EDSS | There was an association between a smaller baseline CCV and a larger baseline EDSS | 5 |
| R. H. Lazeron  (256) | Netherlands | 2000 | Case-control | 39  NR  40 (10.6) | RRMS: 24  SPMS: 13  PPMS: 2 | 3 (0-6)** | 10.3 (8.7) | NR | EDSS | There was a correlation between the EDSS and the number of non-cortical lesions found on fast-FLAIR, particularly in the basal ganglia. | 5 |
| C. Y. Lee  (257) | China | 2017 | Case-control | 17  12:5  41 (9.4) | RRMS: 17 | 2 (1.5-4)* | 6 (2.5-11)* | Philips  3 T | EDSS  FSS | The relationship between EDSS and whole brain volume or T2LV was not statistically significant | 7 |
| A. Lema  (258) | UK | 2016 | Cross-sectional | 134  107:27  39 (25-66)** | RRMS: 128  SPMS: 6 | 3.5 (2-6)** | 3 (0-16)** | Siemens  3 T | EDSS | For the whole brain and WML MTR, significant associations were found with the EDSS and T25FW | 8 |
| Lin X  (259) | UK | 2003 | Case-control | 97  67:30  Age:  RRMS:  36 (32.5-42)**  SPMS:  43 (37-47.7)** | RRMS: 49  SPMS: 48 | RRMS:  2.5 (1.5-3.5)**  SPMS:  5.5 (4-6)** | RRMS:  6 (3-9)**  SPMS:  11.5 (8.2-17.7)** | Siemens  1.5 T | EDSS | EDSS was correlated with normalized supratentorial brain volume and normalized upper cervical cord volume in PwMS. | 7 |
| Z. Liptak  (260) | USA | 2008 | Case-control | 45  35:10  45.7 (10.1) | RRMS: 32  SPMS: 8  PPMS: 5 | 2.5 (0-8)** | 7.1 (7.6) | General Electric  1.5 T | EDSS | MOV, UCCV, and BPF were associated with EDSS in MS. | 5 |
| Y. Liu  (261) | China | 2015 | Cross-sectional | 35  27:8  33.1 (9.2) | RRMS: 35 | 3.3 (0-7.5)‡ | 3.7 (3.2) | Siemens  3 T | EDSS | There was a significant correlation between EDSS and UCCA. | 6 |
| S. Ljubisavljevic  (262) | Serbia | 2013 | Cross-sectional | 57  45:12  40 (23-58)** | RRMS: 57 | NR | 84 (1-396)^m^** | Siemens  1.5 T | EDSS | T2 weighted lesion number and EDSS correlated significantly in RRMS. | 5 |
| L. Locatelli  (263) | USA | 2004 | Cross-sectional | 39  25:14  42.6 (9.7) | RRMS: 39 | 2.1 (0-5.5)‡ | 11 (5.4) | Philips  1.5 T | EDSS | EDSS was significantly correlated with BPF, BPAV, T1LL, and T2LL. | 7 |
| H. Lund  (264) | Denmark | 2012 | Cross-sectional | 50  38:12  35.9 (9.4) | RRMS: 49  SPMS: 1 | 2.5 (0-6)** | NR | NR | EDSS | There was no association between total T2 lesion load and the neurological measure EDSS. | 5 |
| A. Maarouf  (265) | France | 2022 | Case-control | 46  34:12  47 (8.6) | RRMS: 16  SPMS: 9  BMS: 21 | 3 (2.1) | 18.7 (5.6) | Siemens  3 T | EDSS | GMSA extent and EDSS did not show a linear correlation | 5 |
| A. MacKenzie-Graham  (266) | USA | 2016 | Cross-sectional | 133  133:0  37.4 (7.5) | RRMS: 133 | 2 (1.5-3)* | 3.1 (4.3) | NR | EDSS  T25FW  9HPT  MSFC | The voxelwise loss of GM in different sites was associated with certain MS disabilities. | 9 |
| S. Magon  (267) | Canada | 2014 | Cross-sectional | 118  83:35  44.8 (10.4) | RRMS: 118 | 2.5 (0-7)** | 15.2 (8.6) | NR | EDSS  FSS | EDSS scores were related to thalamic shape, as found in the displacement-based analysis. | 8 |
| C. Mainero  (268) | Italy | 2001 | Cross-sectional | 23  17:6  37.1 (8.9) | RRMS: 21  SPMS: 2 | 2 (0-6)** | 5 (1-21)** | NR  1.5 T | EDSS | EDSS showed a correlation with T2LV among PwMS. | 6 |
| A. Minneboo  (269) | Netherlands | 2007 | Cohort | 89  55:34  36.5 (29.6-43.5)* | RRMS: 74  PPMS: 15 | 2 (2-3)* | 1.6 (0.7-4.1)* | Siemens  1 T | EDSS | There was an association between NBV and EDSS in MS. | 6 |
| P. D. Molyneux  (270) | UK | 2001 | Cross-sectional | 688  NR  NR | NR | NR | NR | NR | EDSS | TLV was correlated with EDSS in MS. | 7 |
| R. W. Motl  (271) | USA | 2015 | Cross-sectional | 63  47:16  50 (9.1) | RRMS: 46  SPMS/PPMS: 15 | 5.5 (3.0)* | 11.4 (8.3) | Erlangen  3 T | EDSS  T25FW | The pallidum and possibly the caudate may be correlated with walking performance in MS. | 7 |
| R. W. Motl  (272) | USA | 2016 | Cross-sectional | 79  53:26  46.6 (8.6) | RRMS: 52  SPMS/PPMS: 27 | 3.5 (3.5)* | 12.2 (8.7) | General Electric  3 T | EDSS  T25FW | Volumes of the thalamus, caudate, putamen, and pallidum were significantly correlated with T25FW speed. | 5 |
| Y. Nakamura  (273) | Japan | 2020 | Cross-sectional | 140  100:40  45 (12.8) | RRMS: 111  SPMS: 20  PPMS: 9 | 3.1 (2.6) | 14.4 (9.8) | NR | EDSS  FSS | There was an association between cervical CS-SCA and disability in patients with MS. | 8 |
| A. S. Nielsen  (274) | USA | 2013 | Cross-sectional | 27  19:8  43 (7.9) | RRMS: 8  SPMS: 9  CIS: 10 | 3 (0-6)** | 9.3 (7.3) | Siemens  7 T | EDSS | CL count was significantly correlated with disability as measured by the EDSS | 5 |
| J. F. Nielsen  (275) | Denmark | 2004 | Case-control | 20  12:8  41 (24-53)** | RRMS: 20 | 3.5 (1.5-6)** | 7 (1-20)* | Philips  1.5 T | EDSS | EDSS scores were weakly correlated with the number of MRI-evident lesions. | 6 |
| G. J. Nijeholt  (276) | Netherlands | 1998 | Cross-sectional | 91  54:37  RRMS:  35 (25-57)**  SPMS:  46 (30-65)**  PPMS:  45 (20-69)** | RRMS: 28  SPMS: 32  PPMS: 31 | RRMS:  1 (0-4.5)**  SPMS:  4.75 (2-6.5)**  PPMS:  4.5 (2-6)** | RRMS:  5 (1-30)**  SPMS:  9 (1-40)**  PPMS:  6 (1-24)** | Siemens  1 T | EDSS  FSS | There were associations between MRI parameters and disability among PwMS. | 6 |
| J. Oh  (277) | USA | 2014 | Case-control | 133  86:47  44 (12) | RRMS: 74  SPMS: 36  PPMS: 19  CIS: 4 | 3.5 (2-6)* | 10 (9) | Philips  3 T | EDSS  MSFC | There were correlations between disability measurement and normalization of spinal cord volume. | 8 |
| A. Ozturk  (278) | USA | 2010 | Cohort | 53  33:20  46 (22-66)‡ | RRMS: 28  SPMS: 13  PPMS: 12 | 3.5 (0-7)** | 11 (0-42)‡ | Philips  3 T | EDSS  T25FW  9HPT  MSFC | The EDSS scores were unrelated to the whole-brain volume, normalized callosal volume, and fraction of lesioned areas. | 6 |
| O. OZTURK  (279) | Turkey | 2021 | Case-control | 58  44:14  37.1 (10.2) | NR | NR | 5.8 (5.4) | Philips  3 T | EDSS  T25FW  9HPT | There were associations between functional disability and normalized cerebellar volume in MS. Brain and GM volume were correlated with T25FW. | 6 |
| A. Paolillo  (280) | Italy | 2002 | Cohort | 68  47:21  30.5 (7.3) | RRMS: 68 | 2 (1-5)** | 5 (2.9) | Toshiba  0.5 T | EDSS | There was a strong correlation between baseline T2LL and baseline T1LL and changes in EDSS scores at 6-year follow-up. | 5 |
| A. Papadopoulou  (281) | Switzerland | 2013 | Cross-sectional | 91  58:33  49.1 (1.8) | RRMS/CIS: 65  SPMS/PPMS: 26 | 3.6 (1.8) | 17.4 (9.4) | Siemens  1.5 T | EDSS | An association between the WM lesion volume and EDSS was found in MS patients. | 6 |
| M. Pardini  (282) | UK | 2015 | Case-control | 71  44:27  46.2 (10.3) | RRMS: 44  SPMS: 27 | 4.5 (1-8.5)** | 15.4 (10) | Philips  3 T | EDSS | BPF, NAWM MTR, and cervical cord area had significant associations with EDSS in PwMS. | 6 |
| A. Pichler  (283) | Austria | 2012 | Cohort | 42  26:16  23.2 (9.7) | NR | 1.5 (1) | 1.3 (1.7) | Philips  1.5 T | EDSS | A significant correlation was observed between T1LL and EDSS at follow-up in pMS. | 6 |
| M. R. Piras  (284) | Italy | 2003 | Cohort | 12  10:2  32.5 (8) | RRMS: 12 | 1.8 (1.3) | 7.7 (6.3) | NR | EDSS | There were significant correlations between EDSS and total MRI score, fourth ventricle, and cerebellum at follow-up | 5 |
| A. H. Poonawalla  (285) | USA | 2010 | Cross-sectional | 126  101:25  40.7 (20-64)** | RRMS: 126 | 1.5 (0-6.5)** | NR | Philips  3 T | EDSS | T1LL showed a significant association with EDSS among PwMS. | 7 |
| C.C. Quattrocchi  (286) | Italy | 2010 | Cross-sectional | 20  13:7  47 (26-70)** | RRMS: NR  SPMS: NR | RRMS:  4.0 €  SPMS:  7.1 € | 23 (8-39)** | Siemens  3 T | EDSS | Whole brain, brainstem, and cerebellum were not associated with EDSS in PwMS. | 5 |
| L. Ramio´-Torrenta  (287) | UK | 2005 | Case-control | 43  19:24  45.2 (10.8) | PPMS: 43 | 4.5 (3-7)** | 3.3 (0.8) | General Electric  1.5 T | EDSS  T25FW  9HPT  MSFC | There were abnormalities in MTR of NAWM and NAGM in early PPMS. Both were associated with disability. | 5 |
| W. Rashid  (288) | Austria | 2012 | Case-control | 28  22:6  36.2 (26-56)‡ | RRMS: 28 | 1.5 (0-3.5)** | 1.6 (0.5-3)** | General Electric  1.5 T | EDSS | Subjects with MS showed a negative correlation between EDSS and BPV. | 6 |
| F. Riahi  (289) | Canada | 1998 | Cross-sectional | 39  NR  NR | RRMS: 39 | NR | NR | Philips  1.5 T | EDSS | Cerebral T2 weighted lesion volume had a better correlation with EDSS compared to corticospinal tract lesion load in MS. | 6 |
| S. D. Roosendaal  (290) | Netherlands | 2011 | Cross-sectional | 977  627:350  43.6 (10.7) | RRMS: 657  SPMS: 125  PPMS: 50  CIS: 95 | 2.6 (1.8) | 10.4 (8.8) | Siemens  1.5 T | EDSS | MS physical impairment was better explained by GM volume than by WM volume. | 9 |
| M. Rovaris  (291) | Italy | 2008 | Case-control | 226  114:112  49.4 (10.1) | PPMS: 226 | 5.5 (2-8)** | 9.6 (5.9) | Siemens  General Electric  NR | EDSS | EDSS was associated with NBV, CSA, WBT, NAGM MTR among PwMS. | 8 |
| M. Rovaris  (292) | Italy | 2010 | Cohort | 369  259:110  42.7 (10) | RRMS: 187  BMS: 182 | RRMS:  1.5 (0-3)**  BMS:  2 (0-3)** | 14.5 (8.3) | Siemens  General Electric  1.5 T | EDSS | There was an association between BMS patients with higher brain LV and worsening locomotor disability after a short period of follow-up. | 7 |
| R. A. Rudick  (293) | USA | 2006 | Cohort | 30  NR  36.3 (6) | RRMS: 30 | 2.2 (0.8) | 6.1 (5.8) | NR | EDSS  MSFC | At the follow-up visit, changes in T2 lesion volumes were associated with MSFC scores. | 6 |
| R. A. Rudick  (294) | USA | 2008 | Cohort | 70  52:8  43 (9.1) | RRMS: 36  SPMS: 27  CIS: 7 | 3 (2.2) | 9.5 (8.6) | Siemens  1.5 T | EDSS  9HPT  MSFC | The MSFC disability progression had a closer connection to brain atrophy than the EDSS disability progression. | 6 |
| S. Ruggieri  (295) | USA | 2015 | Cross-sectional | 26  14:12  50.9 (31-65)‡ | PPMS: 26 | 4 (1.5-6)** | 8.8 (4.6) | Phillips  3 T | EDSS  T25FW  9HPT | Normalized brain and WM volume was associated with 9HPT, significantly. T2LL correlated to T25FW, significantly. | 6 |
| J. Rusz  (296) | Czech Republic | 2019 | Case-control | 123  92:31  44 (11) | RRMS: 97  SPMS: 15  PPMS: 8  CIS: 3 | 3.6 (1-6.5)‡ | 14.4 (7.6) | Siemens  3 T | EDSS  T25FW  9HPT | WM fraction, GM fraction, and BPF had a significant association with disability in MS. | 8 |
| M. Sailer  (297) | Germany | 2003 | Case-control | 20  10:10  36.9 (7.5) | RRMS: 11  SPMS: 9 | 4 (1-7)** | 6.1 (3.6) | General Electric  1.5 T | EDSS | There was a significant correlation between EDSS score and cortical thickness, but not with T1 and T2 lesion volumes. | 6 |
| R.S. Samson  (298) | UK | 2013 | Case-control | 61  36:25  48.1 (10.4) | RRMS: 31  SPMS: 14  PPMS: 16 | RRMS:  2.5 (1-6.5)**  SPMS:  6.5 (4.5-8.5)**  PPMS:  6 (1.5-6.5)** | 14.7 (10.8) | Phillips  3 T | EDSS  T25FW  9HPT | The presence of abnormal MTRs in the cortex was more clinically significant in progressive MS compared to relapse-onset MS. | 5 |
| M. P. Sanfilipo  (299) | USA | 2005 | Case-control | 41  32:9  39.8 (6.6) | RRMS: 35  SPMS: 6 | 3.2 (1-8)‡ | 9.5 (6.3) | Phillips  1.5 T | EDSS  T25FW | EDSS and T25FW were correlated with MRI parameters in PwMS. | 7 |
| M. Saraste  (300) | Switzerland | 2020 | Case-control | 79  60:19  48.3 (43-53)* | RRMS: 56  SPMS: 23 | 3 (2-3.7)* | 14.9 (9-19)* | Philips  3 T | EDSS | NAWM, T1LL, and T2LL were not correlated with EDSS, significantly. | 7 |
| J. Sastre-Garriga  (301) | UK | 2004 | Case-control | 43  19:24  46 (22-65)** | PPMS: 43 | 4.5 (3-7)** | 3.3 (2-5)‡ | General Electric  1.5 T | EDSS  T25FW  9HPT  MSFC | WM atrophy in the PPMS appeared to have a stronger correlation to clinical outcome and focal damage to the WM than GM atrophy. | 6 |
| R. Schlaeger  (302) | USA | 2014 | Cross-sectional | 113  84:29  51.6 (10.1) | RMS: 116  PPMS/SPMS/PRMS: 25 | RMS:  (0-5.0)^^  Mild MS:  (0-2.5)^^  SPMS/PPMS/PRMS:  (2.0-8.0)^^ | 17.6 (9.5) | Siemens  3 T | EDSS  T25FW  9HPT | WM area, GM area. and UCCA at C2 and C3 showed significant correlations with disability measurements in PwMS. | 8 |
| R. Schlaeger  (303) | USA | 2015 | Cross-sectional | 142  86:56  51 (10.3) | RRMS: 99  SPMS: 31  PPMS: 12 | NR | 16.8 (9.4) | NR  3 T | EDSS  T25FW  9HPT | Disability in PwMS was associated with SC GM area, SC WM area, and TCA. | 8 |
| N. Shiee  (304) | USA | 2012 | Cross-sectional | 60  45:15  43 (20-68)‡ | RRMS: 43  SPMS: 9  PPMS: 8 | 2.7 (0-6.5)** | 8 (0-30)‡ | Phillips  3 T | EDSS  25FWT  9HPT  MSFC | Despite lower WM volume being associated with greater disability, WM volume was on average within normal ranges in MS patients. | 7 |
| F. Song  (305) | China | 2008 | Cross-sectional | 29  17:12  39 (23-58)‡ | RRMS: 13  SPMS: 16 | 5 (1-8)** | 8.3 (1-21)‡ | Philips  1.5 T | EDSS | Patients with SPMS showed a significant correlation between UCCA and EDSS, but patients with RRMS did not. | 6 |
| M. P. Sormani  (306) | Italy | 2009 | Cohort | 906  634:272  28 (7) | RRMS: 548  SPMS: 358 | 3.5 (0-7)** | 10 (7) | NR | EDSS | T2LV change was associated with EDSS at follow-up. | 8 |
| P. Sowa  (307) | Norway | 2018 | Cross-sectional | 77  60:17  27 (16-53)** | RRMS: 77 | 2 (0-6.5)** | 9 (1-32)** | General Electric  3 T | EDSS | Correlations between EDSS and brain volume, normalized WM volume, and WML volume were found. | 7 |
| J. M. Stankiewicz  (308) | USA | 2009 | Cross-sectional | 32  22:10  41.7 (8.7) | RRMS: 25  SPMS: 4  PPMS: 2  CIS: 1 | 2.1 (0-8)‡ | 9.3 (8) | General Electric  3 T  Philips  1.5 T | EDSS  T25FW | 1.5T and 3T FLLV score and EDSS score showed a moderately significant association (r= 0.25 and r= 0.15, respectively). | 5 |
| R. C. Tam  (309) | Canada | 2011 | Cross-sectional | 24  18:6  52.9 (7.5) | RRMS: 13  SPMS: 11 | 5 (2.2) | 24.7 (5.1) | Philips  3 T | EDSS | The correlation between black holes and EDSS was greatly impacted by intensity variations. | 5 |
| M. C. Tartaglia  (310) | Italy | 2016 | Cross-sectional | 60  NR  40.3 (8.6) | RRMS: 46  SPMS: 14 | 3.3 (2.2) | 10.5 (8.2) | Philips  1.5 T | EDSS  FSS | There was a significant correlation between T2LL and EDSS. | 5 |
| S. Tauhid  (311) | USA | 2015 | Cross-sectional | 100  75:25  45.5 (9.7) | RRMS: 76  SPMS: 12  PPMS: 6  CIS: 6 | 2.2 (0-7.5)‡ | 12.1 (8.1) | General Electric  1.5 T | EDSS | The EDSS was significantly correlated with BPV, T1LL, and T1/T2 lesion volume ratio. | 8 |
| E. Tavazzi  (312) | USA | 2007 | Case-control | 432  346:86  44.4 (10.2) | RRMS: 294  SPMS: 123  PPMS: 15 | 3.3 (0-8.5)‡ | 12 (8.6) | General Electric  1.5 T | EDSS  9HPT | Significant correlations were found between EDSS and T1LL, T2LL, BPF, GMF, and WMF. | 8 |
| C.W. Tjoa  (313) | USA | 2005 | Cross-sectional | 47  32:15  42.4 (8.5) | RRMS: 41  SPMS: 6 | 3.4 (1-7)‡ | 11.1 (1-43)‡ | Philips  1.5 T | EDSS  T25FW | In addition to traditional lesion and atrophy measures, dentate nucleus T2 hypointensity was also independently associated with ambulatory impairment and disability. | 6 |
| F. Tovar-Moll  (314) | Brazil | 2015 | Cross-sectional | 25  18:7  43.3 (8) | RRMS: 16  SPMS: 9 | RRMS:  2.2 (0-6)**  SPMS:  6 (1.5-7.5)** | 13.1 (6.3) | General Electric  3 T | EDSS  T25FW | Each of the disability scores used for MS patients was associated with both global and regional cBHs lesion volumes. | 5 |
| A. Traboulsee  (315) | Canada | 2003 | Case-control | 95  68:27  41.5 (8.3) | RRMS: 70  SPMS: 25 | 2.5 (0-8.5)** | 10.7 (7.7) | General Electric  1.5 T | EDSS | All T2 lesion loads, lesion MTRs, and BPFs for MS patients were significantly correlated with the EDSS. | 6 |
| A. Trufanov  (316) | Russia | 2021 | Cross-sectional | 68  53:15  32.4 (5.8) | RRMS: 40  SPMS: 28 | 2.6 (1.4) | 3.6 (3.4) | Siemens  3 T | EDSS | The thalamic pulvinar nuclei and the geniculate bodies on the left showed correlations with the EDSS. | 5 |
| C. Tur  (317) | UK | 2010 | Case-control | 47  19:28  45.1 (10.6) | PPMS: 47 | 4.5 (1.5-7)** | 3.4 (0.8) | General Electric  1.5 T | EDSS  T25FW  9HPT | There was a significant association between EDSS and T2LL in PwMS. | 5 |
| T. Uher  (318) | Australia | 2018 | Cross-sectional | 1052  734:318  38.1 (8.8) | RRMS: 1052 | 2 (0-6.5)** | NR | Philips  1.5 T | EDSS | T1LL, T2LL, and BPF were significantly associated with EDSS. | 8 |
| L. Vaithianathar  (319) | UK | 2002 | Cross-sectional | 15  NR  RRMS:  38 (33-49)*  SPMS:  51 (41-53)* | RRMS: 8  SPMS: 7 | RRMS:  3.5 (2.5-4)*  SPMS:  5 (4-6)* | RRMS:  6.5 (2-13)*  SPMS:  20 (12-30)* | Siemens  1.5 T | EDSS  T25FW | Correlations between the median cervical cord T1 and the normalized UCCA were significant both with the EDSS and T25FW. | 6 |
| A. O. Varog˘lu  (320) | Turkey | 2009 | Case-control | 27  27:0  33.3 (11.7) | RRMS: 14  SPMS: 13 | 4 (1.8) | 5.6 (4.2) | Siemens  1.5 T | EDSS | A correlation was found between cerebellum volumes and EDSS scores among patients with RRMS and SPMS. | 5 |
| M.M. Vellinga  (321) | Netherlands | 2009 | Cross-sectional | 325  202:123  41.5 (11) | RRMS: 205  SPMS: 65  PPMS: 55 | 3 (2-5)* | 6.3 (1.4-12)* | NR | EDSS  T25FW  9HPT | MS disability was associated with lesion load throughout the brain. | 7 |
| M. Wilson  (322) | UK | 2001 | Cross-sectional | 22  16:6  40 (24-55)‡ | RRMS: 15  SPMS: 7 | 3.2 (0-5.5)‡ | 11.1 (1.5-28)‡ | Siemens  1.5 T | EDSS | Total lesion load and T2LL were associated with EDSS in PwMS. | 5 |
| M. Wilson  (323) | UK | 2003 | Case-control | 25  17:8  40 (26-54)‡ | RRMS: 25 | 3 (1-6)‡ | 7 (1-21)‡ | Siemens  1.5 T | EDSS | There was no significant correlation between the volume of T2 lesions and the EDSS. | 5 |
| G.F. Wu  (324) | USA | 2007 | Cross-sectional | 45  34:11  44 (11) | RRMS: 42  SPMS: 3 | 2 (0-6)** | 5 (1-21)** | General Electric  Siemens  1.5 T | EDSS  MSFC | There was no significant correlation between BPF and EDSS scores, nor between T2LL and EDSS scores. | 6 |
| A. Wuschek  (325) | Germany | 2022 | Cross-sectional | 161  112:49  35 (19.1-57.8)** | RRMS: 143  CIS: 18 | 1.5 (0-8.5)** | 0.06 (0-1.3)** | Philips  3 T | EDSS | The SC lesion volume was associated with EDSS in MS patients with lesions on SC MRI. | 8 |
| Ö. Yaldizli  (326) | Switzerland | 2010 | Cross-sectional | 169  126:43  42 (11.3) | RRMS: 145  SPMS: 24 | 2.6 (0.7) | 10.9 (8.8) | Siemens  1.5 T | EDSS | CCI and presence of T1 lesions were correlated with EDSS at diagnosis while T2LL were not. | 8 |
| T. Yamamoto  (327) | Japan | 2016 | Cross-sectional | 34  27:7  43 (10) | NR | 2.8 (2.2) | 11.9 (7) | General Electric  1.5 T | EDSS | EDSS was associated with length of cervical and thoracolumbar cord. | 5 |
| F. Yousuf  (328) | USA | 2017 | Cohort | 53  41:12  43.6 (10.1) | RRMS: 53 | 1 (1.3) | 13 (8.5) | General Electric  1.5 T | EDSS | In MS patients, disability was not significantly correlated with T2LL, BPF, and cGMF. | 6 |
| R. Zivadinov  (329) | USA | 2004 | Cohort | 30  20:10  34.4 (8.5) | RRMS: 30 | 1.5 (1-1.5)* | 4.9 (4.8) | Philips  1.5 T | EDSS | No correlation was found between changes in BPF and changes in EDSS during the study. | 6 |
| R. Zivadinov  (330) | USA | 2008 | Case-control | 66  48:14  41.2 (12.4) | RRMS: 34  SPMS: 14  PPMS: 7  CIS: 11 | 3.1 (2.1) | 11.8 (10.7) | General Electric  1.5 T | EDSS | EDSS was correlated with CCAV, CCF, cervical T2LV, BPF, and T1LV, significantly. | 7 |
| R. Zivadinov  (331) | Italy | 2003 | Cross-sectional | 45  30:15  42 (11.2) | RRMS: 45 | 2 (1-5)** | 12.8 (7.5) | Philips  1.5 T | EDSS | Patients with MS showed coefficients of correlation between BPF and EDSS. | 6 |
| D. K. B. Li  (332) | Canada | 2006 | Cross-sectional | 1312  879:433  29.7 (8) | RRMS: 463  SPMS: 673  CIS: 176 | 4 (0-6.5)** | 118.4 (102.8)^m^ | NR | EDSS | There was a significant relationship between T2 BOD and disability plateaued over time. | 8 |
| G. D. E. Papini  (333) | Italy | 2017 | Case-control | 53  30:23  45 (9) | RRMS: 39  SPMS: 11  PPMS: 2  NR: 1 | 2 (1-7)** | NR | Siemens  NR | EDSS | Summation of the diameters of the right and left foramina of jugular showed a borderline significant correlation with EDSS. | 5 |
| A. Barreiro-González  (334) | Spain | 2022 | Case-control | 50  41:9  45.1 (10.6) | RRMS: 31  SPMS/PPMS: 19 | 3 (1-7)** | 15.5 (8.2) | Philips  3 T | EDSS | There was a correlation between EDSS and SC volume and brain lesion volume among PwMS.. | 5 |
| N.F. Kalkers  (335) | Netherlands | 2001 | Cross-sectional | 134  78:56  43 (11) | RRMS: 64  SPMS: 41  PPMS: 29 | 3 (1.5-6)* | 11.2 (8.6) | Siemens  1.0 T, 1.5 T | EDSS  T25FW  9HPT  MSFC | T1LL and T2LL showed significant correlations with 9HPT. | 7 |
| M. Bross  (336) | USA | 2021 | Cross-sectional | 65  43:22  42.5 (10.1) | RRMS: 65 | African Americans: 2.5 (0-6.5)**  Caucasian Americans:  1.5 (0-8)** | 8.9 (8.1) | Siemens  3 T | EDSS  T25FW  9HPT | Among total MS patients, T25FW correlated with thalamus, putamen, pallidum, hippocampus, amygdala, accumbens, brain stem, and deep GM volumes. | 6 |
| M.A. Clarke  (337) | USA | 2022 | Cross-sectional | 18  12:6  45.5 (14.6) | RRMS: 11  SPMS: 3  CIS: 4 | 1 (0-6.5)** | 11.5 (10.6) | Philips  3 T | EDSS  T25FW | EDSS and T25FW correlated with T1 and T2 lesion volume in hub- and hub+ tract. | 5 |
| C. H. Coffman  (338) | USA | 2022 | Cohort | 43  28:15  41 (10) | RRMS: 43 | 1.7 (1-4)* | 10.2 (8.6) | Siemens  3 T | EDSS | EDSS correlated with ring lesion count but was not associated with ring lesion volumes. | 5 |
| P. Filippi  (339) | Slovakia | 2020 | Cross-sectional | 56  38:18  41.4 (11.1) | RRMS: 56 | 3.2 (2.5) | 10 (8.5) | Philips  3 T | EDSS | EDSS correlated with brain volume, GM volume, FLAIR and T1 lesion volume, and brain atrophy. | 5 |
| C. Lukas  (340) | Germany | 2013 | Cross-sectional | 440  287:153  NR | RRMS: 311  SPMS: 92  PPMS: 37 | Center A:  4 (2.5-5)*  Center B:  3 (2-4)* | Center A:  9 (4-16)*  Center B:  11 (6-19)* | Siemens  1.5 T | EDSS  T25FW  9HPT | EDSS, T25FW, and 9HPT had significant correlation with UCCA among PwMS. | 8 |
| R. Zivadinov  (341) | USA | 2005 | Cross-sectional | 34  25:9  39.7 (11.6) | RRMS: 34 | 1.5 (1-5.5)** | 10.6 (9) | Philips  1.5 T | EDSS | There was not a significant correlation between BPF and EDSS in Buffalo (r= -0.37), Trieste (r= -0.36), and SIENAX (r= -0.16) methods. | 6 |
| S. Cappelle  (342) | Spain | 2020 | Cohort | 132  79:53  34.8 (8.3) | NR | NR | 3.8 (6.1) | NR  1.5 T, 3 T | EDSS | EDSS was correlated with CCI, normalized CCA, and width of third ventricle at 1-year follow-up. | 7 |
| P. O’Connor  (343) | Canada | 1998 | Cohort | 50  NR  NR | RRMS: 50 | 2 (1.5)* | NR | General Electric  1.5 T | EDSS | MRI lesion volume was associated with EDSS at year 1 and 2. | 4 |
| M.V. Spampinato  (344) | USA | 2017 | Cohort | 40  33:7  42.9 (12.5) | RRMS: 32  SPMS: 2  PPMS: 1  NR: 5 | 3.4 (1.9) | 8 (6.4) | Siemens  1.5 T | EDSS | There was no significant correlation between T2LV and EDSS at 12 months. | 7 |
| J. H. Simon  (345) | USA | 1999 | Cohort | 237  178:59  35.6 (7) | RRMS: 237 | 2.3 (0.8) | 6.3 (5.6) | NR | EDSS | Measures of brain atrophy provide valuable insight into the progression of MS. | 7 |
| M. A. J. Madsen  (346) | Denmark | 2022 | Cross-sectional | 50  33:17  45.1 (12.1) | RRMS: 37  SPMS: 13 | 3.5 (0-6.5)** | 10 (0-35)** | Philips  7 T | EDSS  FSS | EDSS was significantly correlated with total cortical lesion count, total cortical lesion volume, spinal cord CSA, and infratentorial lesion volume in pwMS. | 7 |
| E. Giugni  (347) | Italy | 1997 | Cross-sectional | 85  58:27  33.3 (8.9) | RRMS: 54  SPMS: 31 | 3 (1.7) | 6.8 (4.8) | Toshiba  0.5 T | EDSS | T1 and T2 weighted images showed a weak correlation between disability and total lesion volume in RRMS. The T2 weighted image of the infratentorial lesion volume was the only marker of disability in SPMS. | 5 |
| K. M. Hasan  (348) | USA | 2012 | Case-control | 54  39:15  41.7 (9.6) | RRMS: 54 | 1.6 (1.5) | 9.3 (8.7) | Philips  3 T | EDSS | EDSS and whole brain lesion load showed strongest correlations with qMRI metrics in regions such as the corpus callosum. | 6 |
| G. Iannucci  (349) | Italy | 1999 | Case-control | 72  43:29  34.3 (8.3) | RRMS: 44  SPMS: 28 | 4 (1-7)** | 6 (1-24)** | NR  1.5 T | EDSS  FSS | EDSS was significantly associated with T1LV, T2LV, T1 lesion count, and T2 lesion count among pwMS. | 6 |
| A. H. Maghzi  (350) | USA | 2014 | Cohort | 40  29:11  36 (9.3) | RRMS/CIS: 40 | 2 (0-5.5)** | 7.5 (4.9)^m^ | General Electric  3 T | EDSS  T25FW  9HPT | Long-term clinical outcomes were associated with lesion volume and brain volume in early MS. | 7 |
| K. M. Hasan  (351) | USA | 2009 | Case-control | 32  24:8  41.9 (8.5) | RRMS: 32 | 1.7 (0-6.5)** | 9 (9) | Philips  3 T | EDSS | Normalized brain lesion volume and caudate volume had significant correlation with EDSS in pwMS. | 6 |
| M. Boaventura  (352) | Spain | 2022 | Cohort | 22  14:8  36.5 (6.8) | RRMS: 22 | 1.5 (0-8.5)** | 8.9 (7.7) | Siemens  3 T | EDSS | The clinical disability of MS was associated with lower T1-w/T2-w values. | 7 |
| S. Mammi  (353) | Italy | 1996 | Cross-sectional | 130  91:39  38 (9.2) | RRMS: 36  SPMS: 42  PPMS: 13  BMS: 39 | 3.1 (0-8)‡ | 9.8 (1-27)‡ | NR  1.5 T | EDSS | EDSS was significantly correlated with total lesion volume in all pwMS, but it increased when only RRMS and SPMS were included. | 7 |
| Y. Miki  (354) | USA | 1999 | Cohort | 18  14:4  40.8 (26-53)‡ | RRMS: 18 | NR | 3.2 (4.3-10.7)^m^‡ | General Electric  1.5 T | EDSS | There was not a significant relationship between change in T2LV and change in EDSS among RRMS patients. | 5 |
| P. D. Molyneux  (355) | UK | 1998 | Cohort | 73  52:21  33 (15-61)** | RRMS: 46  SPMS: 27 | 3.5 (0-8)** | 5 (1-28)** | General Electric  1.5 T  Siemens  1.5 T  Technicare  0.6 T  Toshiba  0.5 T | EDSS | A measurement of the annual lesion load provides an effective method for monitoring ongoing disease activity in MS. | 6 |
| M. Rizkallah  (356) | Egypt | 2021 | Cross-sectional | 31  23:8  27.5 (4.9) | RRMS: 31 | 3.6 (1-6.5)‡ | NR | General Electric  1.5 T | EDSS | A significant positive correlation was observed between WM lesions volume and EDSS. However, EDSS had negatives relationships with thalamus and putamen volume. | 5 |
| H. Yokote  (357) | Japan | 2021 | Cross-sectional | 32  25:7  42.4 (13) | RRMS: 24  SPMS: 5  CIS: 3 | 1.7 (0-7.5)** | 10.5 (7) | Toshiba  1.5 T | EDSS | EDSS was significantly associated with NCGMV and NBV in pwMS. | 7 |
| G. A. Bateman  (358) | Australia | 2021 | Cross-sectional | 103  79:24  47.8 (13) | RRMS: 90  SPMS: 11  PPMS: 2 | 3.2 (2) | 9.8 (9) | Siemens  1.5 T | EDSS | There was a moderate negative correlation between the EDSS and GM volume | 7 |
| C. Till  (359) | Canada | 2011 | Case-control | 31  24:7  16.3 (2.3) | RRMS: 31 | 1 (0-4)** | 4.3 (3.1) | General Electric  1.5 T | EDSS | EDSS was significantly associated with CCA among RRMS patients. | 6 |
| H. Yokote  (360) | Japan | 2017 | Cross-sectional | 23  17:6  44 (11) | RRMS: 19  SPMS: 3  PPMS: 1 | 2 (0-8)** | 12 (8.1) | General Electric  1.5 T | EDSS | Change of EDSS was significantly correlated with NBV, NCGMV, and T2LV in pwMS. | 7 |
| Y. Zhang  (361) | Canada | 2010 | Cohort | 8  6:2  41.8 (4.3) | RRMS: 8 | 2.7 (1.4) | 11.8 (6.3) | General Electric  3 T | EDSS | Monitoring disability of pwMS may benefit from measuring deep GM T2 hypointensity at high field MRI. | 6 |
| D. Pareto  (362) | Spain | 2020 | Cross-sectional | 22  15:7  41.1 (6) | RRMS: 22 | 2.4 (0-6)‡ | 5.6 (0-20)‡ | Siemens  3 T | EDSS | EDSS had relationships with MTR and T1/T2 mean values in the lesion mask. | 6 |
| Y. Ge  (363) | USA | 2001 | Cross-sectional | 30   24:6  34.9 (26-52) ‡ | RRMS: 30 | 2 (NR) | 3.8 (1-14.9) ‡ | General Electric  1.5 T | EDSS | Fractional WM and GM were not associated with EDSS. | 6 |
| N. Bergsland  (364) | Czech Republic | 2012 | Cross-sectional | 177  139:38  30.7 (7.8) | RRMS: 177 | 2 (0-4)** | 5.4 (4.8) | Philips  1.5 T | EDSS | The reduction of the thalamus and normalized cortical volumes were associated with the increase of EDSS score. | 8 |
| A. Parry (365) | UK | 2002 | Case-control | 24  16:8  46 (30-58)** | RRMS: 13  SPMS: 11 | 4 (1.5-6)** | 15 (5-24)** | NR  3 T | EDSS | The black hole lesion volumes correlated with EDSS. | 5 |
| D. A. Rudko (366) | Canada | 2014 | Case-control | 25  18:7  37.3 (6.1) | RRMS: 21  CIS: 4 | 1.7 (0-6) ‡ | 5.6 (5.4) | NR  7 T | EDSS | Quantitative susceptibility and R2* maps enable the detection of demyelination and iron buildup in MS which demonstrates a robust association with clinical disability. | 5 |
| T. Akaishi (367) | Japan | 2017 | Cross-sectional | 85  61:24  39.1 (10.1) | RRMS: 72  SPMS: 11  PPMS: 2 | 2 (0-9)** | NR | Philips  3 T | EDSS | EDSS correlated with whole brain and GM volumes. | 5 |
| D. M. Harrison (368) | USA | 2014 | Case-control | 34  18:16  43 (10) | RRMS: 28  SPMS/PPMS: 6 | 3 (1-6.5)** | 10 (7) | Philips  7 T | EDSS  T25FW  9HPT  MSFC | Normalized WM lesion, BPF, and WM volumes were associated with EDSS. | 7 |
| S. G. M. Edwards (369) | UK | 2001 | Cross-sectional | 40  27:13  RRMS: 32.9 (21-47) ‡  SPMS: 40.1 (29-50) ‡ | RRMS: 20  SPMS: 20 | RRMS: 1.68(0-3.5) ‡  SPMS: 5.5 (3.5-6.5) ‡ | RRMS: 5.6 (1-17) ‡  SPMS: 11.8 (3-28) ‡ | Siemens  1.5 T | EDSS | EDSS correlated with normalized ventricular volume and was not associated with normalized WM, GM, and corpus callosum volumes. | 6 |
| M.K. Houtchens (370) | USA | 2007 | Case-control | 79  60:19  42 (9.6) | RRMS: 62  SPMS: 16  PPMS: 1 | 3.4 (2) | 9.7 (7.1) | General Electric  1.5 T | EDSS  T25FW | EDSS correlated with thalamic atrophy. | 6 |
| C. Thaler (371) | Italy | 2021 | Case-control | 47  17:30  38.5 (10.7) | RRMS: 47 | 2 (0-6)** | 6.5 (6) | Siemens  3 T | EDSS  T25FW  9HPT | T2 lesion, normalized brain, and GM volumes correlated with EDSS. | 7 |
| A. Lashkari (372) | USA | 2021 | Case-control | 22  17:5  38.5 (2.1) ‡‡ | RRMS/SPMS: NR | 2.8 (2.3) ‡‡ | 9.8 (7.8) ‡‡ | General Electric  3 T | EDSS | The normalized lateral ventricle and hippocampus volumes were associated with EDSS. | 6 |
| J. Grimaud (373) | UK | 1999 | Cross-sectional | 15  NR  36 (29-45)** | RRMS: 3  SPMS: 6  BMS: 6 | 4.5 (1-9.5)** | NR | General Electric  1.5 T | EDSS | There were associations between EDSS, and MTR lesion and T1 lesion load. | 5 |
| J.H. Simon (374) | USA | 2000 | Cohort | 160  125:35  36.3 (6.9) | RRMS: 160 | 2.3 (0.8) | 6 (5.2) | General Electric  1.5 T | EDSS | There was a correlation between the change of T1 hypointense lesion volume and the change of EDSS during two years follow up. | 7 |
| S. Cader (375) | UK | 2007 | Cross-sectional | 15  8:7  43 (27-52)** | RRMS: 13  SPMS: 2 | 2.5 (1-4)** | 8 (2-14)** | Siemens  1.5 T | EDSS | A correlation was found between EDSS and T2 lesion load. | 5 |
| P. M. Matthews (376) | Canada | 1996 | Cross-sectional | 29  12:17  42.6 (10.3) | RRMS: 11  SPMS: 18 | 5.5 (1.1) | 15.3 (7.9) | Philips  1.5 T | EDSS | No correlation was found between EDSS and lesion volume. | 4 |
| L. Kappos  (377) | Germany | 1988 | Cohort | 74  NR  NR | RRMS: 38  RPMS: 15  PMS: 21 | 2.9 (0.2) | 8.6 (1.3) | Siemens  1 T | EDSS | There was a correlation between EDSS and lesion volume. | 4 |
| F. L. Chiang (378) | USA | 2021 | Cross-sectional | 20  12:8  36 (9) | RRMS: 20 | 4 (1-8)** | 7.8 (5.4) | Siemens  3 T | EDSS | EDSS did not correlate with normalized lesion, WM, GM, and whole brain volumes. | 6 |
| M. J. de la Peña (379) | Spain | 2019 | Cross-sectional | 28  18:10  45 (39-52)** | RRMS: 20  SPMS: 8 | 1 (0.3-3.6)** | 8 (5-14)** | General Electric  1.5 T | EDSS | EDSS correlated negatively with thalamus transit time and cerebral blood flow in the frontal cortex. | 5 |
| P. A. Narayana (380) | USA | 2013 | Cross-sectional | 250  188:62  38.2 (9) | RRMS: 250 | 1.8 (1.1) | 1.5 (3) | General Electric, Siemens, Philips  1.5 and 3 T | EDSS | There was no association between EDSS and average cortical thickness. | 7 |
| K. Morgen (381) | Germany | 2007 | Case-control | 19  14:5  32.4 (8.2) | RRMS: 19 | 1.5 (1.1) | 1.6 (1.4) | Siemens  1.5 T | EDSS | WM lesion load was associated positively with EDSS. | 5 |
| N. Ramezani (382) | Iran | 2023 | Cross-sectional | 126  97:29  36.6 (8.2) | RRMS: 83  SPMS: 43 | RRMS: 1 (1-2)*  SPMS:  3.5 (3-4)* | 7.7 (6) | Siemens  1.5 T | EDSS  9HPT | Thalamic asymmetry index and normalized white matter volumes correlated with EDSS. | 7 |
| S. Tauhid (383) | USA | 2014 | Cross-sectional | 175  124:51  42.7 (9.1) | RRMS: 115  SPMS:42  CIS: 18 | 2.5 (2.3) | 9.6 (8.6) | NR  1.5 T | EDSS | T2 lesion volume and BPF correlated with EDSS. | 7 |

Table S1. Main characteristics of the included studies.

‡ Mean (range), ‡‡ Mean (SE), * Median (IQR), ** Median (range), ***Median (std), ^ Mode (range), a.u. Arbitrary units, ^z^: z score, ^^Range, € Mean, ^m^ Months.

BFW: Brain Free Water, BMS: Benign Multiple Sclerosis, BOD: Borden of Disease, BP: Brain Parenchymal, BPF: Brain Parenchymal Fraction, BPV: Brain Parenchymal Volume, cBH: Chronic Black Hole, CCA: Corpus Callosum Area, CCAV: Cervical Cord Absolute Volume, CCF: Cervical Cord Fraction, CCI: Corpus Callosum Index, CCV: Corpus Callosum Volume, CGM: Cortical Gray Matter, CGMF: Cortical Gray Matter Fraction, CIS: Clinically Isolated Syndrome, CSA: Cross Sectional cord Area, CSF: Cerebrospinal Fluid, CS-SCA: Cross-sectional Spinal Cord Area, CSE: Conventional Spin Echo, CST: Corticospinal Tract, CV: Coefficient of Variation, CWM-LL: Cerebral White Matter Lesion Load, DGM: Deep Gay Matter, EDSS: Expanded Disability Status Scale, fFLAIR: fast Fluid Attenuated Inversion Recovery, FLLV: Fluid Attenuated Inversion Recovery Lesion Volume, FSE: Fast Spin Echo, FSS: Functional Systems Scores, GM: Gray Matter, GMF: Grey Matter Fraction, GMSA: Grey-matter Sodium Abnormalities, ICL: Intracortical Lesion, ICV: Intracranial Volume, MOV: Medulla Oblongata Volume, MR: Magnetic Resonance, MRI: Magnetic Resonance Imaging, MRS: Magnetic Resonance Spectroscopy, MS: Multiple Sclerosis, MSFC: Multiple Sclerosis Functional Composite, MSSS: Multiple Sclerosis Severity Score, MTI: Magnetization Transfer Imaging, MTR: Magnetization transfer ratio, MUCCA: Mean Upper Cervical Cord Area, NABT: Normal-appearing Brain Tissue, NABV: Normal Appearing Brain Volume, NAGM: Normal Appearing Gray Matter, NAWM: Normal Appearing White Matter, NBV: Normalized Brain Volume, NCV: Normalized Cortical Volume, NCGMV: Normalized Cortical Grey Matter Volume, 9HPT: Nine-Hole Peg Test, NR: Not Reported, pMS: Pediatric-onset Multiple Sclerosis, PPMS: Primary Progressive, PMS: Progressive MS, PwMS: Patients with Multiple Sclerosis, qMRI: Quantitative Magnetic Resonance Imaging, QSM: Quantitative Susceptibility Mapping, RMS: Relapsing Multiple Sclerosis, RRMS: Relapsing Remitting Multiple Sclerosis, SC: Spinal Cord, SCA: Spinal Cord Cross sectional area, SPMS: Secondary Progressive Multiple Sclerosis, T: Tesla, T1LL: T1 Lesion Load, T1LV: T1 Lesion volume, T2LL: T2 Lesion Load, T2LV: T2 Lesion Volume, T25FW: Timed 25-Foot Walk WM: White Matter, TCA: Total Cord Area, TLV: Total Lesion Volume, UCCA: Upper Cervical Cord Cross-sectional Area, UCCV: Upper Cervical Cord Volume, VF: Ventricular Fraction, WBT: Whole Brain Tissue, WM: White Matter, WMF: White Matter Fraction, WML: White Matter Lesion.

**References:**

1. Kim M, Seo JW, Kim MS, Lee KH, Kim M. White matter tract density index is associated with disability in multiple sclerosis. Neurobiol Dis. 2024;198:106548.

2. Koenig KA, Sakaie KE, Ontaneda D, Mahajan KR, Oh SH, Nakamura K, et al. High-resolution diffusion tensor imaging of the fornix predicts memory function in multiple sclerosis. Mult Scler J Exp Transl Clin. 2024;10(2):20552173241240937.

3. Molenaar PCG, Noteboom S, van Nederpelt DR, Krijnen EA, Jelgerhuis JR, Lam KH, et al. Digital outcome measures are associated with brain atrophy in patients with multiple sclerosis. J Neurol. 2024.

4. Romanò F, Valsasina P, Pagani E, De Simone A, Parolin E, Filippi M, et al. Structural and functional correlates of disability, motor and cognitive performances in multiple sclerosis: Focus on the globus pallidus. Mult Scler Relat Disord. 2024;86:105576.

5. Vanheule E, Cambron M, Dobai A, Casselman JW. Rim lesions in MS at 3T: clinical correlation and possible radiological alternatives for daily practice at lower field strength. J Neuroradiol. 2024;51(4):101165.

6. Sun J, Guo M, Chai L, Xu S, Lizhu Y, Li Y, et al. Distinct virtual histology of grey matter atrophy in four neuroinflammatory diseases. Brain. 2024.

7. Wang X, Wang X, Yan Z, Yin F, Li Y, Liu X, et al. Enhanced choroid plexus segmentation with 3D UX-Net and its association with disease progression in multiple sclerosis. Mult Scler Relat Disord. 2024;88:105750.

8. Xie Y, Zhu H, Yao Y, Liu C, Wu S, Zhang Y, et al. Enlarged choroid plexus in relapsing-remitting multiple sclerosis may lead to brain structural changes through the glymphatic impairment. Mult Scler Relat Disord. 2024;85:105550.

9. Zivadinov R, Pei J, Clayton D, Goldman DE, Winger RC, Cabatingan MS, et al. Evolution of atrophied T2 lesion volume in primary-progressive multiple sclerosis: results from the phase 3 ORATORIO study. J Neurol Neurosurg Psychiatry. 2024;95(6):536-43.

10. Harper JG, York EN, Meijboom R, Kampaite A, Thrippleton MJ, Kearns PKA, et al. Quantitative T(1) brain mapping in early relapsing-remitting multiple sclerosis: longitudinal changes, lesion heterogeneity and disability. Eur Radiol. 2024;34(6):3826-39.

11. Barnett M, Wang D, Beadnall H, Bischof A, Brunacci D, Butzkueven H, et al. A real-world clinical validation for AI-based MRI monitoring in multiple sclerosis. NPJ Digit Med. 2023;6(1):196.

12. Calvi A, Clarke MA, Prados F, Chard D, Ciccarelli O, Alberich M, et al. Relationship between paramagnetic rim lesions and slowly expanding lesions in multiple sclerosis. Mult Scler. 2023;29(3):352-62.

13. de Ruiter LRJ, Loonstra FC, Jelgerhuis JR, Coerver EME, Toorop AA, van Leeuwen ICE, et al. Association of volumetric MRI measures and disability in MS patients of the same age: Descriptions from a birth year cohort. Mult Scler Relat Disord. 2023;71:104568.

14. Bergsland N, Dwyer MG, Jakimovski D, Tavazzi E, Benedict RHB, Weinstock-Guttman B, et al. Association of Choroid Plexus Inflammation on MRI With Clinical Disability Progression Over 5 Years in Patients With Multiple Sclerosis. Neurology. 2023;100(9):e911-e20.

15. Hamzaoui M, Garcia J, Boffa G, Lazzarotto A, Absinta M, Ricigliano VAG, et al. Positron Emission Tomography with [(18) F]-DPA-714 Unveils a Smoldering Component in Most Multiple Sclerosis Lesions which Drives Disease Progression. Ann Neurol. 2023;94(2):366-83.

16. Hartmann A, Noro F, Bahia PRV, Fontes-Dantas FL, Andreiuolo RF, Lopes FCR, et al. The clinical-radiological paradox in multiple sclerosis: myth or truth? Arq Neuropsiquiatr. 2023;81(1):55-61.

17. Hofmann A, Krajnc N, Dal-Bianco A, Riedl CJ, Zrzavy T, Lerma-Martin C, et al. Myeloid cell iron uptake pathways and paramagnetic rim formation in multiple sclerosis. Acta Neuropathol. 2023;146(5):707-24.

18. Noteboom S, van Nederpelt DR, Bajrami A, Moraal B, Caan MWA, Barkhof F, et al. Feasibility of detecting atrophy relevant for disability and cognition in multiple sclerosis using 3D-FLAIR. J Neurol. 2023;270(11):5201-10.

19. Peño LIC, De Silanes De Miguel CL, de Torres L, Ortiz ME, Moreno MJG, Rodeño BO, et al. Brain Atrophy and Physical and Cognitive Disability in Multiple Sclerosis. Basic Clin Neurosci. 2023;14(2):311-6.

20. Pontillo G, Petracca M, Monti S, Quarantelli M, Lanzillo R, Costabile T, et al. Clinical correlates of R1 relaxometry and magnetic susceptibility changes in multiple sclerosis: a multi-parameter quantitative MRI study of brain iron and myelin. Eur Radiol. 2023;33(3):2185-94.

21. Slezáková D, Kadlic P, Jezberová M, Boleková V, Valkovič P, Minar M. Brain volume loss in multiple sclerosis is independent of disease activity and might be prevented by early disease-modifying therapy. Neurol Neurochir Pol. 2023;57(3):282-8.

22. Rogić Vidaković M, Ćurković Katić A, Pavelin S, Bralić A, Mikac U, Šoda J, et al. Transcranial Magnetic Stimulation Measures, Pyramidal Score on Expanded Disability Status Scale and Magnetic Resonance Imaging of Corticospinal Tract in Multiple Sclerosis. Bioengineering (Basel). 2023;10(10).

23. Steffen F, Uphaus T, Ripfel N, Fleischer V, Schraad M, Gonzalez-Escamilla G, et al. Serum Neurofilament Identifies Patients With Multiple Sclerosis With Severe Focal Axonal Damage in a 6-Year Longitudinal Cohort. Neurol Neuroimmunol Neuroinflamm. 2023;10(1).

24. Filippi M, Horsfield MA, Morrissey SP, MacManus DG, Rudge P, McDonald WI, et al. Quantitative brain MRI lesion load predicts the course of clinically isolated syndromes suggestive of multiple sclerosis. Neurology. 1994;44(4):635-41.

25. Filippi M, Campi A, Colombo B, Pereira C, Martinelli V, Baratti C, et al. A spinal cord MRI study of benign and secondary progressive multiple sclerosis. J Neurol. 1996;243(7):502-5.

26. Genç B, Şen S, Aslan K, İncesu L. Volumetric changes in hypothalamic subunits in patients with relapsing remitting multiple sclerosis. Neuroradiology. 2023;65(5):899-905.

27. Gass A, Barker GJ, Kidd D, Thorpe JW, MacManus D, Brennan A, et al. Correlation of magnetization transfer ratio with clinical disability in multiple sclerosis. Ann Neurol. 1994;36(1):62-7.

28. Filippi M, Paty DW, Kappos L, Barkhof F, Compston DA, Thompson AJ, et al. Correlations between changes in disability and T2-weighted brain MRI activity in multiple sclerosis: a follow-up study. Neurology. 1995;45(2):255-60.

29. Gasperini C, Horsfield MA, Thorpe JW, Kidd D, Barker GJ, Tofts PS, et al. Macroscopic and microscopic assessments of disease burden by MRI in multiple sclerosis: relationship to clinical parameters. J Magn Reson Imaging. 1996;6(4):580-4.

30. Gawne-Cain ML, O'Riordan JI, Coles A, Newell B, Thompson AJ, Miller DH. MRI lesion volume measurement in multiple sclerosis and its correlation with disability: a comparison of fast fluid attenuated inversion recovery (fFLAIR) and spin echo sequences. J Neurol Neurosurg Psychiatry. 1998;64(2):197-203.

31. Berg D, Mäurer M, Warmuth-Metz M, Rieckmann P, Becker G. The correlation between ventricular diameter measured by transcranial sonography and clinical disability and cognitive dysfunction in patients with multiple sclerosis. Arch Neurol. 2000;57(9):1289-92.

32. Ge Y, Grossman RI, Udupa JK, Wei L, Mannon LJ, Polansky M, et al. Brain atrophy in relapsing-remitting multiple sclerosis and secondary progressive multiple sclerosis: longitudinal quantitative analysis. Radiology. 2000;214(3):665-70.

33. Bakshi R, Benedict RH, Bermel RA, Jacobs L. Regional brain atrophy is associated with physical disability in multiple sclerosis: semiquantitative magnetic resonance imaging and relationship to clinical findings. J Neuroimaging. 2001;11(2):129-36.

34. Hickman SJ, Brierley CM, Silver NC, Moseley IF, Scolding NJ, Compston DA, et al. Infratentorial hypointense lesion volume on T1-weighted magnetic resonance imaging correlates with disability in patients with chronic cerebellar ataxia due to multiple sclerosis. J Neurol Sci. 2001;187(1-2):35-9.

35. Kalkers NF, Bergers E, Castelijns JA, van Walderveen MA, Bot JC, Adèr HJ, et al. Optimizing the association between disability and biological markers in MS. Neurology. 2001;57(7):1253-8.

36. Filippi M, Cercignani M, Inglese M, Horsfield MA, Comi G. Diffusion tensor magnetic resonance imaging in multiple sclerosis. Neurology. 2001;56(3):304-11.

37. Bakshi R, Benedict RH, Bermel RA, Caruthers SD, Puli SR, Tjoa CW, et al. T2 hypointensity in the deep gray matter of patients with multiple sclerosis: a quantitative magnetic resonance imaging study. Arch Neurol. 2002;59(1):62-8.

38. Ciccarelli O, Brex PA, Thompson AJ, Miller DH. Disability and lesion load in MS: a reassessment with MS functional composite score and 3D fast FLAIR. J Neurol. 2002;249(1):18-24.

39. Griffin CM, Dehmeshki J, Chard DT, Parker GJ, Barker GJ, Thompson AJ, et al. T1 histograms of normal-appearing brain tissue are abnormal in early relapsing-remitting multiple sclerosis. Mult Scler. 2002;8(3):211-6.

40. Hardmeier M, Wagenpfeil S, Freitag P, Fisher E, Rudick RA, Kooijmans-Coutinho M, et al. Atrophy is detectable within a 3-month period in untreated patients with active relapsing remitting multiple sclerosis. Arch Neurol. 2003;60(12):1736-9.

41. Bermel RA, Sharma J, Tjoa CW, Puli SR, Bakshi R. A semiautomated measure of whole-brain atrophy in multiple sclerosis. J Neurol Sci. 2003;208(1-2):57-65.

42. Caon C, Zvartau-Hind M, Ching W, Lisak RP, Tselis AC, Khan OA. Intercaudate nucleus ratio as a linear measure of brain atrophy in multiple sclerosis. Neurology. 2003;60(2):323-5.

43. De Stefano N, Matthews PM, Filippi M, Agosta F, De Luca M, Bartolozzi ML, et al. Evidence of early cortical atrophy in MS: relevance to white matter changes and disability. Neurology. 2003;60(7):1157-62.

44. Agosta F, Pagani E, Caputo D, Filippi M. Associations between cervical cord gray matter damage and disability in patients with multiple sclerosis. Arch Neurol. 2007;64(9):1302-5.

45. Dehmeshki J, Chard DT, Leary SM, Watt HC, Silver NC, Tofts PS, et al. The normal appearing grey matter in primary progressive multiple sclerosis: a magnetisation transfer imaging study. J Neurol. 2003;250(1):67-74.

46. Hickman SJ, Coulon O, Parker GJ, Barker GJ, Stevenson VL, Chard DT, et al. Application of a B-spline active surface technique to the measurement of cervical cord volume in multiple sclerosis from three-dimensional MR images. J Magn Reson Imaging. 2003;18(3):368-71.

47. Archibald CJ, Wei X, Scott JN, Wallace CJ, Zhang Y, Metz LM, et al. Posterior fossa lesion volume and slowed information processing in multiple sclerosis. Brain. 2004;127(Pt 7):1526-34.

48. Davies GR, Ramió-Torrentà L, Hadjiprocopis A, Chard DT, Griffin CM, Rashid W, et al. Evidence for grey matter MTR abnormality in minimally disabled patients with early relapsing-remitting multiple sclerosis. J Neurol Neurosurg Psychiatry. 2004;75(7):998-1002.

49. Fisniku LK, Chard DT, Jackson JS, Anderson VM, Altmann DR, Miszkiel KA, et al. Gray matter atrophy is related to long-term disability in multiple sclerosis. Ann Neurol. 2008;64(3):247-54.

50. Bejarano B, Bianco M, Gonzalez-Moron D, Sepulcre J, Goñi J, Arcocha J, et al. Computational classifiers for predicting the short-term course of Multiple sclerosis. BMC Neurol. 2011;11:67.

51. Brandão CO, Ruocco HH, Farias AS, Oliveira C, Cendes F, Damasceno BP, et al. Intrathecal immunoglobulin G synthesis and brain injury by quantitative MRI in multiple sclerosis. Neuroimmunomodulation. 2006;13(2):89-95.

52. Calabrese M, Rinaldi F, Seppi D, Favaretto A, Squarcina L, Mattisi I, et al. Cortical diffusion-tensor imaging abnormalities in multiple sclerosis: a 3-year longitudinal study. Radiology. 2011;261(3):891-8.

53. Cohen-Adad J, Benner T, Greve D, Kinkel RP, Radding A, Fischl B, et al. In vivo evidence of disseminated subpial T2* signal changes in multiple sclerosis at 7 T: a surface-based analysis. Neuroimage. 2011;57(1):55-62.

54. Cohen AB, Neema M, Arora A, Dell'oglio E, Benedict RH, Tauhid S, et al. The relationships among MRI-defined spinal cord involvement, brain involvement, and disability in multiple sclerosis. J Neuroimaging. 2012;22(2):122-8.

55. Hannoun S, Durand-Dubief F, Confavreux C, Ibarrola D, Streichenberger N, Cotton F, et al. Diffusion tensor-MRI evidence for extra-axonal neuronal degeneration in caudate and thalamic nuclei of patients with multiple sclerosis. AJNR Am J Neuroradiol. 2012;33(7):1363-8.

56. Hildebrandt H, Hahn HK, Kraus JA, Schulte-Herbrüggen A, Schwarze B, Schwendemann G. Memory performance in multiple sclerosis patients correlates with central brain atrophy. Mult Scler. 2006;12(4):428-36.

57. Calabrese M, De Stefano N, Atzori M, Bernardi V, Mattisi I, Barachino L, et al. Detection of cortical inflammatory lesions by double inversion recovery magnetic resonance imaging in patients with multiple sclerosis. Arch Neurol. 2007;64(10):1416-22.

58. Damasceno A, Damasceno BP, Cendes F. The clinical impact of cerebellar grey matter pathology in multiple sclerosis. PLoS One. 2014;9(5):e96193.

59. Janardhan V, Suri S, Bakshi R. Multiple sclerosis: hyperintense lesions in the brain on nonenhanced T1-weighted MR images evidenced as areas of T1 shortening. Radiology. 2007;244(3):823-31.

60. Bakshi R, Neema M, Healy BC, Liptak Z, Betensky RA, Buckle GJ, et al. Predicting clinical progression in multiple sclerosis with the magnetic resonance disease severity scale. Arch Neurol. 2008;65(11):1449-53.

61. Furby J, Hayton T, Anderson V, Altmann D, Brenner R, Chataway J, et al. Magnetic resonance imaging measures of brain and spinal cord atrophy correlate with clinical impairment in secondary progressive multiple sclerosis. Mult Scler. 2008;14(8):1068-75.

62. Bellmann-Strobl J, Stiepani H, Wuerfel J, Bohner G, Paul F, Warmuth C, et al. MR spectroscopy (MRS) and magnetisation transfer imaging (MTI), lesion load and clinical scores in early relapsing remitting multiple sclerosis: a combined cross-sectional and longitudinal study. Eur Radiol. 2009;19(8):2066-74.

63. Deppe M, Marinell J, Krämer J, Duning T, Ruck T, Simon OJ, et al. Increased cortical curvature reflects white matter atrophy in individual patients with early multiple sclerosis. Neuroimage Clin. 2014;6:475-87.

64. Calabrese M, Rocca MA, Atzori M, Mattisi I, Bernardi V, Favaretto A, et al. Cortical lesions in primary progressive multiple sclerosis: a 2-year longitudinal MR study. Neurology. 2009;72(15):1330-6.

65. Hayton T, Furby J, Smith KJ, Altmann DR, Brenner R, Chataway J, et al. Grey matter magnetization transfer ratio independently correlates with neurological deficit in secondary progressive multiple sclerosis. J Neurol. 2009;256(3):427-35.

66. Gao KC, Nair G, Cortese IC, Koretsky A, Reich DS. Sub-millimeter imaging of brain-free water for rapid volume assessment in atrophic brains. Neuroimage. 2014;100:370-8.

67. Kearney H, Rocca MA, Valsasina P, Balk L, Sastre-Garriga J, Reinhardt J, et al. Magnetic resonance imaging correlates of physical disability in relapse onset multiple sclerosis of long disease duration. Mult Scler. 2014;20(1):72-80.

68. Datta S, Staewen TD, Cofield SS, Cutter GR, Lublin FD, Wolinsky JS, et al. Regional gray matter atrophy in relapsing remitting multiple sclerosis: baseline analysis of multi-center data. Mult Scler Relat Disord. 2015;4(2):124-36.

69. Dupuy SL, Tauhid S, Kim G, Chu R, Tummala S, Hurwitz S, et al. MRI detection of hypointense brain lesions in patients with multiple sclerosis: T1 spin-echo vs. gradient-echo. Eur J Radiol. 2015;84(8):1564-8.

70. Benedetti B, Rocca MA, Rovaris M, Caputo D, Zaffaroni M, Capra R, et al. A diffusion tensor MRI study of cervical cord damage in benign and secondary progressive multiple sclerosis patients. J Neurol Neurosurg Psychiatry. 2010;81(1):26-30.

71. Giorgio A, Palace J, Johansen-Berg H, Smith SM, Ropele S, Fuchs S, et al. Relationships of brain white matter microstructure with clinical and MR measures in relapsing-remitting multiple sclerosis. J Magn Reson Imaging. 2010;31(2):309-16.

72. Martola J, Bergström J, Fredrikson S, Stawiarz L, Hillert J, Zhang Y, et al. A longitudinal observational study of brain atrophy rate reflecting four decades of multiple sclerosis: a comparison of serial 1D, 2D, and volumetric measurements from MRI images. Neuroradiology. 2010;52(2):109-17.

73. Gonyea JV, Watts R, Applebee A, Andrews T, Hipko S, Nickerson JP, et al. In vivo quantitative whole-brain T1 rho MRI of multiple sclerosis. J Magn Reson Imaging. 2015;42(6):1623-30.

74. Bonneville F, Moriarty DM, Li BS, Babb JS, Grossman RI, Gonen O. Whole-brain N-acetylaspartate concentration: correlation with T2-weighted lesion volume and expanded disability status scale score in cases of relapsing-remitting multiple sclerosis. AJNR Am J Neuroradiol. 2002;23(3):371-5.

75. Calabrese M, Poretto V, Favaretto A, Alessio S, Bernardi V, Romualdi C, et al. Cortical lesion load associates with progression of disability in multiple sclerosis. Brain. 2012;135(Pt 10):2952-61.

76. Caramanos Z, Francis SJ, Narayanan S, Lapierre Y, Arnold DL. Large, nonplateauing relationship between clinical disability and cerebral white matter lesion load in patients with multiple sclerosis. Arch Neurol. 2012;69(1):89-95.

77. Dupuy SL, Khalid F, Healy BC, Bakshi S, Neema M, Tauhid S, et al. The effect of intramuscular interferon beta-1a on spinal cord volume in relapsing-remitting multiple sclerosis. BMC Med Imaging. 2016;16(1):56.

78. Hackmack K, Weygandt M, Wuerfel J, Pfueller CF, Bellmann-Strobl J, Paul F, et al. Can we overcome the 'clinico-radiological paradox' in multiple sclerosis? J Neurol. 2012;259(10):2151-60.

79. Healy BC, Arora A, Hayden DL, Ceccarelli A, Tauhid SS, Neema M, et al. Approaches to normalization of spinal cord volume: application to multiple sclerosis. J Neuroimaging. 2012;22(3):e12-9.

80. Jaworski J, Psujek M, Janczarek M, Szczerbo-Trojanowska M, Bartosik-Psujek H. Total-tau in cerebrospinal fluid of patients with multiple sclerosis decreases in secondary progressive stage of disease and reflects degree of brain atrophy. Ups J Med Sci. 2012;117(3):284-92.

81. Dupuy SL, Tauhid S, Hurwitz S, Chu R, Yousuf F, Bakshi R. The Effect of Dimethyl Fumarate on Cerebral Gray Matter Atrophy in Multiple Sclerosis. Neurol Ther. 2016;5(2):215-29.

82. Abalo-Lojo JM, Limeres CC, Gómez MA, Baleato-González S, Cadarso-Suárez C, Capeáns-Tomé C, et al. Retinal nerve fiber layer thickness, brain atrophy, and disability in multiple sclerosis patients. J Neuroophthalmol. 2014;34(1):23-8.

83. Grothe M, Lotze M, Langner S, Dressel A. The role of global and regional gray matter volume decrease in multiple sclerosis. J Neurol. 2016;263(6):1137-45.

84. Bakshi R, Neema M, Tauhid S, Healy BC, Glanz BI, Kim G, et al. An expanded composite scale of MRI-defined disease severity in multiple sclerosis: MRDSS2. Neuroreport. 2014;25(14):1156-61.

85. Hubbard EA, Wetter NC, Sutton BP, Pilutti LA, Motl RW. Diffusion tensor imaging of the corticospinal tract and walking performance in multiple sclerosis. J Neurol Sci. 2016;363:225-31.

86. Daams M, Weiler F, Steenwijk MD, Hahn HK, Geurts JJ, Vrenken H, et al. Mean upper cervical cord area (MUCCA) measurement in long-standing multiple sclerosis: relation to brain findings and clinical disability. Mult Scler. 2014;20(14):1860-5.

87. Burgetova A, Dusek P, Vaneckova M, Horakova D, Langkammer C, Krasensky J, et al. Thalamic Iron Differentiates Primary-Progressive and Relapsing-Remitting Multiple Sclerosis. AJNR Am J Neuroradiol. 2017;38(6):1079-86.

88. Harrison DM, Shiee N, Bazin PL, Newsome SD, Ratchford JN, Pham D, et al. Tract-specific quantitative MRI better correlates with disability than conventional MRI in multiple sclerosis. J Neurol. 2013;260(2):397-406.

89. Kantorová E, Ziak P, Kurča E, Koyšová M, Hladká M, Zeleňák K, et al. Visual Evoked Potential and Magnetic Resonance Imaging are More Effective Markers of Multiple Sclerosis Progression than Laser Polarimetry with Variable Corneal Compensation. Front Hum Neurosci. 2014;8:10.

90. Chu R, Hurwitz S, Tauhid S, Bakshi R. Automated segmentation of cerebral deep gray matter from MRI scans: effect of field strength on sensitivity and reliability. BMC Neurol. 2017;17(1):172.

91. Amann M, Papadopoulou A, Andelova M, Magon S, Mueller-Lenke N, Naegelin Y, et al. Magnetization transfer ratio in lesions rather than normal-appearing brain relates to disability in patients with multiple sclerosis. J Neurol. 2015;262(8):1909-17.

92. Bergsland N, Laganà MM, Tavazzi E, Caffini M, Tortorella P, Baglio F, et al. Corticospinal tract integrity is related to primary motor cortex thinning in relapsing-remitting multiple sclerosis. Mult Scler. 2015;21(14):1771-80.

93. Fritz NE, Keller J, Calabresi PA, Zackowski KM. Quantitative measures of walking and strength provide insight into brain corticospinal tract pathology in multiple sclerosis. Neuroimage Clin. 2017;14:490-8.

94. Bernitsas E, Bao F, Seraji-Bozorgzad N, Chorostecki J, Santiago C, Tselis A, et al. Spinal cord atrophy in multiple sclerosis and relationship with disability across clinical phenotypes. Mult Scler Relat Disord. 2015;4(1):47-51.

95. Biberacher V, Boucard CC, Schmidt P, Engl C, Buck D, Berthele A, et al. Atrophy and structural variability of the upper cervical cord in early multiple sclerosis. Mult Scler. 2015;21(7):875-84.

96. Daams M, Steenwijk MD, Wattjes MP, Geurts JJ, Uitdehaag BM, Tewarie PK, et al. Unraveling the neuroimaging predictors for motor dysfunction in long-standing multiple sclerosis. Neurology. 2015;85(3):248-55.

97. Dell'Oglio E, Ceccarelli A, Glanz BI, Healy BC, Tauhid S, Arora A, et al. Quantification of global cerebral atrophy in multiple sclerosis from 3T MRI using SPM: the role of misclassification errors. J Neuroimaging. 2015;25(2):191-9.

98. Galego O, Gouveia A, Batista S, Moura C, Machado E. Brain atrophy and physical disability in primary progressive multiple sclerosis: A volumetric study. Neuroradiol J. 2015;28(3):354-8.

99. Granberg T, Bergendal G, Shams S, Aspelin P, Kristoffersen-Wiberg M, Fredrikson S, et al. MRI-Defined Corpus Callosal Atrophy in Multiple Sclerosis: A Comparison of Volumetric Measurements, Corpus Callosum Area and Index. J Neuroimaging. 2015;25(6):996-1001.

100. Granberg T, Martola J, Bergendal G, Shams S, Damangir S, Aspelin P, et al. Corpus callosum atrophy is strongly associated with cognitive impairment in multiple sclerosis: Results of a 17-year longitudinal study. Mult Scler. 2015;21(9):1151-8.

101. Harrison DM, Roy S, Oh J, Izbudak I, Pham D, Courtney S, et al. Association of Cortical Lesion Burden on 7-T Magnetic Resonance Imaging With Cognition and Disability in Multiple Sclerosis. JAMA Neurol. 2015;72(9):1004-12.

102. Kearney H, Schneider T, Yiannakas MC, Altmann DR, Wheeler-Kingshott CA, Ciccarelli O, et al. Spinal cord grey matter abnormalities are associated with secondary progression and physical disability in multiple sclerosis. J Neurol Neurosurg Psychiatry. 2015;86(6):608-14.

103. Calabrese M, Rocca MA, Atzori M, Mattisi I, Favaretto A, Perini P, et al. A 3-year magnetic resonance imaging study of cortical lesions in relapse-onset multiple sclerosis. Ann Neurol. 2010;67(3):376-83.

104. Ge Y, Grossman RI, Udupa JK, Babb JS, Kolson DL, McGowan JC. Magnetization transfer ratio histogram analysis of gray matter in relapsing-remitting multiple sclerosis. AJNR Am J Neuroradiol. 2001;22(3):470-5.

105. Dimitrov I, Georgiev R, Kaprelyan A, Usheva N, Grudkova M, Drenska K, et al. Brain and lesion volumes correlate with edss in relapsing-remitting multiple sclerosis. Journal of IMAB–Annual Proceeding Scientific Papers. 2015;21(4):1015-8.

106. Arpín EC, Sobrino TG, Vivero CD, del Campo Amigo Jorrín M, Regal AR, González JP, et al. Changes in brain atrophy indices in patients with relapsing-remitting multiple sclerosis treated with natalizumab. Neurodegener Dis Manag. 2016;6(1):5-12.

107. Chu R, Tauhid S, Glanz BI, Healy BC, Kim G, Oommen VV, et al. Whole Brain Volume Measured from 1.5T versus 3T MRI in Healthy Subjects and Patients with Multiple Sclerosis. J Neuroimaging. 2016;26(1):62-7.

108. Gracien RM, Jurcoane A, Wagner M, Reitz SC, Mayer C, Volz S, et al. Multimodal quantitative MRI assessment of cortical damage in relapsing-remitting multiple sclerosis. J Magn Reson Imaging. 2016;44(6):1600-7.

109. Ammitzbøll C, Dyrby TB, Lyksborg M, Schreiber K, Ratzer R, Romme Christensen J, et al. Disability in progressive MS is associated with T2 lesion changes. Mult Scler Relat Disord. 2018;20:73-7.

110. Aymerich FX, Auger C, Alonso J, Alberich M, Sastre-Garriga J, Tintoré M, et al. Cervical Cord Atrophy and Long-Term Disease Progression in Patients with Primary-Progressive Multiple Sclerosis. AJNR Am J Neuroradiol. 2018;39(2):399-404.

111. Dworkin JD, Linn KA, Oguz I, Fleishman GM, Bakshi R, Nair G, et al. An Automated Statistical Technique for Counting Distinct Multiple Sclerosis Lesions. AJNR Am J Neuroradiol. 2018;39(4):626-33.

112. Klineova S, Farber R, Saiote C, Farrell C, Delman BN, Tanenbaum LN, et al. Relationship between timed 25-foot walk and diffusion tensor imaging in multiple sclerosis. Mult Scler J Exp Transl Clin. 2016;2:2055217316655365.

113. Ciampi E, Pareto D, Sastre-Garriga J, Vidal-Jordana A, Tur C, Río J, et al. Grey matter atrophy is associated with disability increase in natalizumab-treated patients. Mult Scler. 2017;23(4):556-66.

114. Gonçalves LI, Dos Passos GR, Conzatti LP, Burger JLP, Tomasi GH, Zandoná M, et al. Correlation between the corpus callosum index and brain atrophy, lesion load, and cognitive dysfunction in multiple sclerosis. Mult Scler Relat Disord. 2018;20:154-8.

115. Hemond CC, Chu R, Tummala S, Tauhid S, Healy BC, Bakshi R. Whole-brain atrophy assessed by proportional- versus registration-based pipelines from 3T MRI in multiple sclerosis. Brain Behav. 2018;8(8):e01068.

116. Khalid F, Healy BC, Dupuy SL, Chu R, Chitnis T, Bakshi R, et al. Quantitative MRI analysis of cerebral lesions and atrophy in post-partum patients with multiple sclerosis. J Neurol Sci. 2018;392:94-9.

117. Khan O, Seraji-Bozorgzad N, Bao F, Razmjou S, Caon C, Santiago C, et al. The Relationship Between Brain MR Spectroscopy and Disability in Multiple Sclerosis: 20-Year Data from the U.S. Glatiramer Acetate Extension Study. J Neuroimaging. 2017;27(1):97-106.

118. Al-Radaideh A, Athamneh I, Alabadi H, Hbahbih M. Cortical and Subcortical Morphometric and Iron Changes in Relapsing-Remitting Multiple Sclerosis and Their Association with White Matter T2 Lesion Load : A 3-Tesla Magnetic Resonance Imaging Study. Clin Neuroradiol. 2019;29(1):51-64.

119. Charalambous T, Tur C, Prados F, Kanber B, Chard DT, Ourselin S, et al. Structural network disruption markers explain disability in multiple sclerosis. Journal of Neurology, Neurosurgery & Psychiatry. 2019;90(2):219-26.

120. D'Hooghe M B, Gielen J, Van Remoortel A, D'Haeseleer M, Peeters E, Cambron M, et al. Single MRI-Based Volumetric Assessment in Clinical Practice Is Associated With MS-Related Disability. J Magn Reson Imaging. 2019;49(5):1312-21.

121. Jakimovski D, Weinstock-Guttman B, Hagemeier J, Vaughn CB, Kavak KS, Gandhi S, et al. Walking disability measures in multiple sclerosis patients: Correlations with MRI-derived global and microstructural damage. J Neurol Sci. 2018;393:128-34.

122. Du XF, Liu J, Hua QF, Wu YJ. Relapsing-Remitting Multiple Sclerosis Is Associated With Regional Brain Activity Deficits in Motor- and Cognitive-Related Brain Areas. Front Neurol. 2019;10:1136.

123. Grahl S, Pongratz V, Schmidt P, Engl C, Bussas M, Radetz A, et al. Evidence for a white matter lesion size threshold to support the diagnosis of relapsing remitting multiple sclerosis. Mult Scler Relat Disord. 2019;29:124-9.

124. Abdelhafeez MA, Zamzam DA, Foad MM, Swelam MS, Abdelnasser A, Aref HA, et al. Magnetic resonance imaging markers of disability in Egyptian multiple sclerosis patients. Mult Scler Relat Disord. 2019;36:101417.

125. Beadnall HN, Wang C, Van Hecke W, Ribbens A, Billiet T, Barnett MH. Comparing longitudinal brain atrophy measurement techniques in a real-world multiple sclerosis clinical practice cohort: towards clinical integration? Ther Adv Neurol Disord. 2019;12:1756286418823462.

126. Huang SY, Fan Q, Machado N, Eloyan A, Bireley JD, Russo AW, et al. Corpus callosum axon diameter relates to cognitive impairment in multiple sclerosis. Ann Clin Transl Neurol. 2019;6(5):882-92.

127. Hänninen K, Viitala M, Paavilainen T, Karhu JO, Rinne J, Koikkalainen J, et al. Thalamic Atrophy Without Whole Brain Atrophy Is Associated With Absence of 2-Year NEDA in Multiple Sclerosis. Front Neurol. 2019;10:459.

128. Andersen KW, Lasič S, Lundell H, Nilsson M, Topgaard D, Sellebjerg F, et al. Disentangling white-matter damage from physiological fibre orientation dispersion in multiple sclerosis. Brain Commun. 2020;2(2):fcaa077.

129. Blindenbacher N, Brunner E, Asseyer S, Scheel M, Siebert N, Rasche L, et al. Evaluation of the 'ring sign' and the 'core sign' as a magnetic resonance imaging marker of disease activity and progression in clinically isolated syndrome and early multiple sclerosis. Mult Scler J Exp Transl Clin. 2020;6(1):2055217320915480.

130. Valdés Cabrera D, Stobbe R, Smyth P, Giuliani F, Emery D, Beaulieu C. Diffusion tensor imaging tractography reveals altered fornix in all diagnostic subtypes of multiple sclerosis. Brain Behav. 2020;10(1):e01514.

131. Carmisciano L, Signori A, Pardini M, Novi G, Lapucci C, Nesi L, et al. Assessing upper limb function in multiple sclerosis using an engineered glove. Eur J Neurol. 2020;27(12):2561-7.

132. Karami V, Mahdavifar R, Habibzadeh A, Nabavi SM. Identification of Multiple Sclerosis lesion subtypes and their quantitative assessments with EDSS using neuroimaging. Network Modeling Analysis in Health Informatics and Bioinformatics. 2020;9(1):38.

133. Kerbrat A, Gros C, Badji A, Bannier E, Galassi F, Combès B, et al. Multiple sclerosis lesions in motor tracts from brain to cervical cord: spatial distribution and correlation with disability. Brain. 2020;143(7):2089-105.

134. Colato E, Stutters J, Tur C, Narayanan S, Arnold DL, Gandini Wheeler-Kingshott CAM, et al. Predicting disability progression and cognitive worsening in multiple sclerosis using patterns of grey matter volumes. J Neurol Neurosurg Psychiatry. 2021;92(9):995-1006.

135. Duan Y, Zhuo Z, Li H, Tian D-C, Li Y, Yang L, et al. Brain structural alterations in MOG antibody diseases: a comparative study with AQP4 seropositive NMOSD and MS. Journal of Neurology, Neurosurgery &amp; Psychiatry. 2021;92(7):709-16.

136. Hidalgo de la Cruz M, Valsasina P, Gobbi C, Gallo A, Zecca C, Bisecco A, et al. Longitudinal cortical thinning progression differs across multiple sclerosis phenotypes and is clinically relevant: A multicentre study. Mult Scler. 2021;27(6):827-40.

137. Ajitomi S, Fujimori J, Nakashima I. Usefulness of two-dimensional measurements for the evaluation of brain volume and disability in multiple sclerosis. Mult Scler J Exp Transl Clin. 2022;8(1):20552173211070749.

138. Al-Iedani O, Lea R, Ribbons K, Ramadan S, Lechner-Scott J. Neurometabolic changes in multiple sclerosis: Fingolimod versus beta interferon or glatiramer acetate therapy. J Neuroimaging. 2022;32(6):1109-20.

139. Beck ES, Maranzano J, Luciano NJ, Parvathaneni P, Filippini S, Morrison M, et al. Cortical lesion hotspots and association of subpial lesions with disability in multiple sclerosis. Mult Scler. 2022;28(9):1351-63.

140. Akaishi T, Takahashi T, Fujihara K, Misu T, Mugikura S, Abe M, et al. Number of MRI T1-hypointensity corrected by T2/FLAIR lesion volume indicates clinical severity in patients with multiple sclerosis. PLoS One. 2020;15(4):e0231225.

141. Bakshi R, Healy BC, Dupuy SL, Kirkish G, Khalid F, Gundel T, et al. Brain MRI Predicts Worsening Multiple Sclerosis Disability over 5 Years in the SUMMIT Study. J Neuroimaging. 2020;30(2):212-8.

142. Boonstra FM, Clough M, Strik M, van der Walt A, Butzkueven H, White OB, et al. Longitudinal tracking of axonal loss using diffusion magnetic resonance imaging in multiple sclerosis. Brain Commun. 2022;4(2):fcac065.

143. Brandstadter R, Ayeni O, Krieger SC, Harel NY, Escalon MX, Katz Sand I, et al. Detection of subtle gait disturbance and future fall risk in early multiple sclerosis. Neurology. 2020;94(13):e1395-e406.

144. Adibi I, Najafi A, Merajifar F, Ramezani N, Nouri H, Jalilvand N, et al. Quantitative Magnetic Resonance Imaging Analysis of Early Markers of Upper Cervical Cord Atrophy in Multiple Sclerosis and Neuromyelitis Optica Spectrum Disorder. Mult Scler Int. 2021;2021:9917582.

145. Akil E, Alp R, Aluclu MU, Acar A, Kaplan I. Serum endocan levels in multiple sclerosis relapse and remission. Eur Rev Med Pharmacol Sci. 2021;25(11):4091-8.

146. Brusini I, Platten M, Ouellette R, Piehl F, Wang C, Granberg T. Automatic deep learning multicontrast corpus callosum segmentation in multiple sclerosis. J Neuroimaging. 2022;32(3):459-70.

147. Al-Radaideh A, Athamneh I, Alabadi H, Hbahbih M. Deep gray matter changes in relapsing-remitting multiple sclerosis detected by multi-parametric, high-resolution magnetic resonance imaging (MRI). Eur Radiol. 2021;31(2):706-15.

148. Bergsland N, Benedict RHB, Dwyer MG, Fuchs TA, Jakimovski D, Schweser F, et al. Thalamic Nuclei Volumes and Their Relationships to Neuroperformance in Multiple Sclerosis: A Cross-Sectional Structural MRI Study. J Magn Reson Imaging. 2021;53(3):731-9.

149. Cao J, Xu X, Zhu J, Wu P, Pang H, Fan G, et al. Rapid quantification of global brain volumetry and relaxometry in patients with multiple sclerosis using synthetic magnetic resonance imaging. Quant Imaging Med Surg. 2022;12(6):3104-14.

150. Guenter W, Betscher E, Bonek R. Predictive Value of the Third Ventricle Width for Neurological Status in Multiple Sclerosis. J Clin Med. 2022;11(10).

151. Hurtado Rúa SM, Kaunzner UW, Pandya S, Sweeney E, Tozlu C, Kuceyeski A, et al. Lesion features on magnetic resonance imaging discriminate multiple sclerosis patients. Eur J Neurol. 2022;29(1):237-46.

152. Ganzetti M, Graves JS, Holm SP, Dondelinger F, Midaglia L, Gaetano L, et al. Neural correlates of digital measures shown by structural MRI: a post-hoc analysis of a smartphone-based remote assessment feasibility study in multiple sclerosis. J Neurol. 2023;270(3):1624-36.

153. Graves JS, Ganzetti M, Dondelinger F, Lipsmeier F, Belachew S, Bernasconi C, et al. Preliminary validity of the Draw a Shape Test for upper extremity assessment in multiple sclerosis. Ann Clin Transl Neurol. 2023;10(2):166-80.

154. Jang H, Ma YJ, Chang EY, Fazeli S, Lee RR, Lombardi AF, et al. Inversion Recovery Ultrashort TE MR Imaging of Myelin is Significantly Correlated with Disability in Patients with Multiple Sclerosis. AJNR Am J Neuroradiol. 2021;42(5):868-74.

155. Kocsis K, Szabó N, Tóth E, Király A, Faragó P, Kincses B, et al. Two Classes of T1 Hypointense Lesions in Multiple Sclerosis With Different Clinical Relevance. Front Neurol. 2021;12:619135.

156. Kolind S, Matthews L, Johansen-Berg H, Leite MI, Williams SC, Deoni S, et al. Myelin water imaging reflects clinical variability in multiple sclerosis. Neuroimage. 2012;60(1):263-70.

157. Kolind S, Seddigh A, Combes A, Russell-Schulz B, Tam R, Yogendrakumar V, et al. Brain and cord myelin water imaging: a progressive multiple sclerosis biomarker. Neuroimage Clin. 2015;9:574-80.

158. Krajnc N, Bsteh G, Kasprian G, Zrzavy T, Kornek B, Berger T, et al. Peripheral Hemolysis in Relation to Iron Rim Presence and Brain Volume in Multiple Sclerosis. Front Neurol. 2022;13:928582.

159. Lapucci C, Schiavi S, Signori A, Sbragia E, Bommarito G, Cellerino M, et al. The role of disconnection in explaining disability in multiple sclerosis. Eur Radiol Exp. 2022;6(1):23.

160. Lapucci C, Romano N, Schiavi S, Saitta L, Uccelli A, Boffa G, et al. Degree of microstructural changes within T1-SE versus T1-GE hypointense lesions in multiple sclerosis: relevance for the definition of "black holes". Eur Radiol. 2020;30(7):3843-51.

161. Megna R, Alfano B, Lanzillo R, Costabile T, Comerci M, Vacca G, et al. Brain tissue volumes and relaxation rates in multiple sclerosis: implications for cognitive impairment. J Neurol. 2019;266(2):361-8.

162. Larassati H, Pandelaki J, Estiasari R, Prihartono J, Firdausia S, Yunus RE, et al. Diffusion magnetic resonance imaging of normal-appearing white matter in multiple sclerosis: correlation with brain volume and clinical disability. J Cent Nerv Syst Dis. 2022;14:11795735221098147.

163. Lee LE, Vavasour IM, Dvorak A, Liu H, Abel S, Johnson P, et al. Cervical cord myelin abnormality is associated with clinical disability in multiple sclerosis. Mult Scler. 2021;27(14):2191-8.

164. Lema Dopico A, Choi S, Hua J, Li X, Harrison DM. Multi-layer analysis of quantitative 7 T magnetic resonance imaging in the cortex of multiple sclerosis patients reveals pathology associated with disability. Mult Scler. 2021;27(13):2040-51.

165. Liu H, Chen H, Wu B, Zhang T, Wang J, Huang K, et al. Functional cortical changes in relapsing-remitting multiple sclerosis at amplitude configuration: a resting-state fMRI study. Neuropsychiatr Dis Treat. 2016;12:3031-9.

166. Liu Y, Meng B, Zeng C, Wang J, Li Y, Yin P, et al. Abnormal Baseline Brain Activity in Patients With Multiple Sclerosis With Simple Spinal Cord Involvement Detected by Resting-State Functional Magnetic Resonance Imaging. J Comput Assist Tomogr. 2015;39(6):866-75.

167. Llufriu S, Blanco Y, Martinez-Heras E, Casanova-Molla J, Gabilondo I, Sepulveda M, et al. Influence of corpus callosum damage on cognition and physical disability in multiple sclerosis: a multimodal study. PLoS One. 2012;7(5):e37167.

168. Losseff NA, Webb SL, O'Riordan JI, Page R, Wang L, Barker GJ, et al. Spinal cord atrophy and disability in multiple sclerosis. A new reproducible and sensitive MRI method with potential to monitor disease progression. Brain. 1996;119 ( Pt 3):701-8.

169. Lu PJ, Barakovic M, Weigel M, Rahmanzadeh R, Galbusera R, Schiavi S, et al. GAMER-MRI in Multiple Sclerosis Identifies the Diffusion-Based Microstructural Measures That Are Most Sensitive to Focal Damage: A Deep-Learning-Based Analysis and Clinico-Biological Validation. Front Neurosci. 2021;15:647535.

170. Lundell H, Svolgaard O, Dogonowski AM, Romme Christensen J, Selleberg F, Soelberg Sørensen P, et al. Spinal cord atrophy in anterior-posterior direction reflects impairment in multiple sclerosis. Acta Neurol Scand. 2017;136(4):330-7.

171. Lycklama à Nijeholt GJ, Castelijns JA, Lazeron RH, van Waesberghe JH, Polman CH, Uitdehaag BM, et al. Magnetization transfer ratio of the spinal cord in multiple sclerosis: relationship to atrophy and neurologic disability. J Neuroimaging. 2000;10(2):67-72.

172. Lyksborg M, Siebner HR, Sørensen PS, Blinkenberg M, Parker GJ, Dogonowski AM, et al. Secondary progressive and relapsing remitting multiple sclerosis leads to motor-related decreased anatomical connectivity. PLoS One. 2014;9(4):e95540.

173. Mamoei S, Jensen HB, Pedersen AK, Nygaard MKE, Eskildsen SF, Dalgas U, et al. Clinical, Neurophysiological, and MRI Markers of Fampridine Responsiveness in Multiple Sclerosis-An Explorative Study. Front Neurol. 2021;12:758710.

174. Margoni M, Franciotta S, Poggiali D, Riccardi A, Rinaldi F, Nosadini M, et al. Cerebellar gray matter lesions are common in pediatric multiple sclerosis at clinical onset. J Neurol. 2020;267(6):1824-9.

175. Markowitz CE, Spitsin S, Zimmerman V, Jacobs D, Udupa JK, Hooper DC, et al. The treatment of multiple sclerosis with inosine. J Altern Complement Med. 2009;15(6):619-25.

176. Mistri D, Cacciaguerra L, Storelli L, Meani A, Cordani C, Rocca MA, et al. The association between cognition and motor performance is beyond structural damage in relapsing-remitting multiple sclerosis. J Neurol. 2022;269(8):4213-21.

177. Mohamed A-AB, Algahalan HA, Thabit MN. Correlation between functional MRI techniques and early disability in ambulatory patients with relapsing–remitting MS. The Egyptian Journal of Neurology, Psychiatry and Neurosurgery. 2022;58(1):1-11.

178. Nair G, Absinta M, Reich DS. Optimized T1-MPRAGE sequence for better visualization of spinal cord multiple sclerosis lesions at 3T. AJNR Am J Neuroradiol. 2013;34(11):2215-22.

179. Nakamura Y, Gaetano L, Matsushita T, Anna A, Sprenger T, Radue EW, et al. A comparison of brain magnetic resonance imaging lesions in multiple sclerosis by race with reference to disability progression. J Neuroinflammation. 2018;15(1):255.

180. Nazarov V, Makshakov G, Kalinin I, Lapin S, Surkova E, Mikhailova L, et al. Concentrations of immunoglobulin free light chains in cerebrospinal fluid predict increased level of brain atrophy in multiple sclerosis. Immunol Res. 2018;66(6):761-7.

181. Nylund M, Sucksdorff M, Matilainen M, Polvinen E, Tuisku J, Airas L. Phenotyping of multiple sclerosis lesions according to innate immune cell activation using 18 kDa translocator protein-PET. Brain Commun. 2022;4(1):fcab301.

182. Okada K, Kakeda S, Tahara M. Olfactory identification associates with cognitive function and the third ventricle width in patients with relapsing-remitting multiple sclerosis. Mult Scler Relat Disord. 2020;38:101507.

183. Orbach L, Menascu S, Hoffmann C, Miron S, Achiron A. Focal cortical thinning in patients with stable relapsing-remitting multiple sclerosis: cross-sectional-based novel estimation of gray matter kinetics. Neuroradiology. 2018;60(2):179-87.

184. Oreja-Guevara C, Charil A, Caputo D, Cavarretta R, Sormani MP, Filippi M. Magnetization transfer magnetic resonance imaging and clinical changes in patients with relapsing-remitting multiple sclerosis. Arch Neurol. 2006;63(5):736-40.

185. Ouellette R, Mangeat G, Polyak I, Warntjes M, Forslin Y, Bergendal Å, et al. Validation of Rapid Magnetic Resonance Myelin Imaging in Multiple Sclerosis. Ann Neurol. 2020;87(5):710-24.

186. Paling D, Solanky BS, Riemer F, Tozer DJ, Wheeler-Kingshott CA, Kapoor R, et al. Sodium accumulation is associated with disability and a progressive course in multiple sclerosis. Brain. 2013;136(Pt 7):2305-17.

187. Paty DW, Li DK. Interferon beta-1b is effective in relapsing-remitting multiple sclerosis. II. MRI analysis results of a multicenter, randomized, double-blind, placebo-controlled trial. UBC MS/MRI Study Group and the IFNB Multiple Sclerosis Study Group. Neurology. 1993;43(4):662-7.

188. Pérez CA, Salehbeiki A, Zhu L, Wolinsky JS, Lincoln JA. Assessment of Racial/Ethnic Disparities in Volumetric MRI Correlates of Clinical Disability in Multiple Sclerosis: A Preliminary Study. J Neuroimaging. 2021;31(1):115-23.

189. Petracca M, Vancea RO, Fleysher L, Jonkman LE, Oesingmann N, Inglese M. Brain intra- and extracellular sodium concentration in multiple sclerosis: a 7 T MRI study. Brain. 2016;139(Pt 3):795-806.

190. Pietroboni AM, Colombi A, Contarino VE, Russo FML, Conte G, Morabito A, et al. Quantitative susceptibility mapping of the normal-appearing white matter as a potential new marker of disability progression in multiple sclerosis. Eur Radiol. 2023;33(8):5368-77.

191. Platten M, Brusini I, Andersson O, Ouellette R, Piehl F, Wang C, et al. Deep Learning Corpus Callosum Segmentation as a Neurodegenerative Marker in Multiple Sclerosis. J Neuroimaging. 2021;31(3):493-500.

192. Ramanathan S, Lenton K, Burke T, Gomes L, Storchenegger K, Yiannikas C, et al. The utility of multimodal evoked potentials in multiple sclerosis prognostication. J Clin Neurosci. 2013;20(11):1576-81.

193. Rasoanandrianina H, Demortière S, Trabelsi A, Ranjeva JP, Girard O, Duhamel G, et al. Sensitivity of the Inhomogeneous Magnetization Transfer Imaging Technique to Spinal Cord Damage in Multiple Sclerosis. AJNR Am J Neuroradiol. 2020;41(5):929-37.

194. Righart R, Biberacher V, Jonkman LE, Klaver R, Schmidt P, Buck D, et al. Cortical pathology in multiple sclerosis detected by the T1/T2-weighted ratio from routine magnetic resonance imaging. Ann Neurol. 2017;82(4):519-29.

195. Rocca MA, Horsfield MA, Sala S, Copetti M, Valsasina P, Mesaros S, et al. A multicenter assessment of cervical cord atrophy among MS clinical phenotypes. Neurology. 2011;76(24):2096-102.

196. Rovaris M, Comi G, Ladkani D, Wolinsky JS, Filippi M. Short-term correlations between clinical and MR imaging findings in relapsing-remitting multiple sclerosis. AJNR Am J Neuroradiol. 2003;24(1):75-81.

197. Ruggieri S, Bharti K, Prosperini L, Giannì C, Petsas N, Tommasin S, et al. A Comprehensive Approach to Disentangle the Effect of Cerebellar Damage on Physical Disability in Multiple Sclerosis. Front Neurol. 2020;11:529.

198. Sailer M, Losseff NA, Wang L, Gawne-Cain ML, Thompson AJ, Miller DH. T1 lesion load and cerebral atrophy as a marker for clinical progression in patients with multiple sclerosis. A prospective 18 months follow-up study. Eur J Neurol. 2001;8(1):37-42.

199. Saini A, Bach K, Poliakov I, Knox KB, Levin MC. Magnetic Resonance Imaging of Spinal Cord Lesions in Patients with Multiple Sclerosis in Saskatchewan, Canada. Int J MS Care. 2021;23(2):47-52.

200. Sbardella E, Petsas N, Tona F, Prosperini L, Raz E, Pace G, et al. Assessing the correlation between grey and white matter damage with motor and cognitive impairment in multiple sclerosis patients. PLoS One. 2013;8(5):e63250.

201. Schmalbrock P, Prakash RS, Schirda B, Janssen A, Yang GK, Russell M, et al. Basal Ganglia Iron in Patients with Multiple Sclerosis Measured with 7T Quantitative Susceptibility Mapping Correlates with Inhibitory Control. AJNR Am J Neuroradiol. 2016;37(3):439-46.

202. Schoonheim MM, Pinter D, Prouskas SE, Broeders TA, Pirpamer L, Khalil M, et al. Disability in multiple sclerosis is related to thalamic connectivity and cortical network atrophy. Mult Scler. 2022;28(1):61-70.

203. Schreiber K, Sørensen PS, Koch-Henriksen N, Wagner A, Blinkenberg M, Svarer C, et al. Correlations of brain MRI parameters to disability in multiple sclerosis. Acta Neurol Scand. 2001;104(1):24-30.

204. Sharma J, Sanfilipo MP, Benedict RH, Weinstock-Guttman B, Munschauer FE, 3rd, Bakshi R. Whole-brain atrophy in multiple sclerosis measured by automated versus semiautomated MR imaging segmentation. AJNR Am J Neuroradiol. 2004;25(6):985-96.

205. Shinoda K, Matsushita T, Nakamura Y, Masaki K, Yamasaki R, Yamaguchi H, et al. HLA-DRB1*04:05 allele is associated with intracortical lesions on three-dimensional double inversion recovery images in Japanese patients with multiple sclerosis. Mult Scler. 2018;24(6):710-20.

206. Has Silemek AC, Fischer L, Pöttgen J, Penner IK, Engel AK, Heesen C, et al. Functional and structural connectivity substrates of cognitive performance in relapsing remitting multiple sclerosis with mild disability. Neuroimage Clin. 2020;25:102177.

207. Simon JH, Jacobs LD, Campion M, Wende K, Simonian N, Cookfair DL, et al. Magnetic resonance studies of intramuscular interferon beta-1a for relapsing multiple sclerosis. The Multiple Sclerosis Collaborative Research Group. Ann Neurol. 1998;43(1):79-87.

208. Spanò B, Cercignani M, Basile B, Romano S, Mannu R, Centonze D, et al. Multiparametric MR investigation of the motor pyramidal system in patients with 'truly benign' multiple sclerosis. Mult Scler. 2010;16(2):178-88.

209. Stankiewicz JM, Neema M, Alsop DC, Healy BC, Arora A, Buckle GJ, et al. Spinal cord lesions and clinical status in multiple sclerosis: A 1.5 T and 3 T MRI study. J Neurol Sci. 2009;279(1-2):99-105.

210. Stevenson VL, Leary SM, Losseff NA, Parker GJ, Barker GJ, Husmani Y, et al. Spinal cord atrophy and disability in MS: a longitudinal study. Neurology. 1998;51(1):234-8.

211. Strik M, Chard DT, Dekker I, Meijer KA, Eijlers AJ, Pardini M, et al. Increased functional sensorimotor network efficiency relates to disability in multiple sclerosis. Mult Scler. 2021;27(9):1364-73.

212. Strik M, Shanahan CJ, van der Walt A, Boonstra FMC, Glarin R, Galea MP, et al. Functional correlates of motor control impairments in multiple sclerosis: A 7 Tesla task functional MRI study. Hum Brain Mapp. 2021;42(8):2569-82.

213. Sugijono SE, Mulyadi R, Firdausia S, Prihartono J, Estiasari R. Corpus callosum index correlates with brain volumetry and disability in multiple sclerosis patients. Neurosciences (Riyadh). 2020;25(3):193-9.

214. Thaler C, Faizy T, Sedlacik J, Holst B, Stellmann JP, Young KL, et al. T1- Thresholds in Black Holes Increase Clinical-Radiological Correlation in Multiple Sclerosis Patients. PLoS One. 2015;10(12):e0144693.

215. Theodorsdottir A, Larsen PV, Nielsen HH, Illes Z, Ravnborg MH. Multiple sclerosis impairment scale and brain MRI in secondary progressive multiple sclerosis. Acta Neurol Scand. 2022;145(3):332-47.

216. Tommasin S, Cocozza S, Taloni A, Giannì C, Petsas N, Pontillo G, et al. Machine learning classifier to identify clinical and radiological features relevant to disability progression in multiple sclerosis. J Neurol. 2021;268(12):4834-45.

217. Tommasin S, De Giglio L, Ruggieri S, Petsas N, Giannì C, Pozzilli C, et al. Multi-scale resting state functional reorganization in response to multiple sclerosis damage. Neuroradiology. 2020;62(6):693-704.

218. Treaba CA, Granberg TE, Sormani MP, Herranz E, Ouellette RA, Louapre C, et al. Longitudinal Characterization of Cortical Lesion Development and Evolution in Multiple Sclerosis with 7.0-T MRI. Radiology. 2019;291(3):740-9.

219. Treaba CA, Herranz E, Barletta VT, Mehndiratta A, Ouellette R, Sloane JA, et al. The relevance of multiple sclerosis cortical lesions on cortical thinning and their clinical impact as assessed by 7.0-T MRI. J Neurol. 2021;268(7):2473-81.

220. Truyen L, Van Waesberghe J, Van Walderveen M, Van Oosten B, Polman C, Hommes O, et al. Accumulation of hypointense lesions (" black holes") on T1 spin-echo MRI correlates with disease progression in multiple sclerosis. Neurology. 1996;47(6):1469-76.

221. Udupa JK, Nyúl LG, Ge Y, Grossman RI. Multiprotocol MR image segmentation in multiple sclerosis: experience with over 1,000 studies. Acad Radiol. 2001;8(11):1116-26.

222. Ukkonen M, Dastidar P, Heinonen T, Laasonen E, Elovaara I. Volumetric quantitation by MRI in primary progressive multiple sclerosis: volumes of plaques and atrophy correlated with neurological disability. Eur J Neurol. 2003;10(6):663-9.

223. Vaithianathar L, Tench CR, Morgan PS, Wilson M, Blumhardt LD. T1 relaxation time mapping of white matter tracts in multiple sclerosis defined by diffusion tensor imaging. J Neurol. 2002;249(9):1272-8.

224. Valcarcel AM, Linn KA, Khalid F, Vandekar SN, Tauhid S, Satterthwaite TD, et al. A dual modeling approach to automatic segmentation of cerebral T2 hyperintensities and T1 black holes in multiple sclerosis. Neuroimage Clin. 2018;20:1211-21.

225. van Walderveen MA, Barkhof F, Hommes OR, Polman CH, Tobi H, Frequin ST, et al. Correlating MRI and clinical disease activity in multiple sclerosis: relevance of hypointense lesions on short-TR/short-TE (T1-weighted) spin-echo images. Neurology. 1995;45(9):1684-90.

226. van Walderveen MA, Lycklama ANGJ, Adèr HJ, Jongen PJ, Polman CH, Castelijns JA, et al. Hypointense lesions on T1-weighted spin-echo magnetic resonance imaging: relation to clinical characteristics in subgroups of patients with multiple sclerosis. Arch Neurol. 2001;58(1):76-81.

227. Vaneckova M, Piredda GF, Andelova M, Krasensky J, Uher T, Srpova B, et al. Periventricular gradient of T(1) tissue alterations in multiple sclerosis. Neuroimage Clin. 2022;34:103009.

228. Versijpt J, Debruyne JC, Van Laere KJ, De Vos F, Keppens J, Strijckmans K, et al. Microglial imaging with positron emission tomography and atrophy measurements with magnetic resonance imaging in multiple sclerosis: a correlative study. Mult Scler. 2005;11(2):127-34.

229. Vidal-Jordana A, Pareto D, Cabello S, Alberich M, Rio J, Tintore M, et al. Optical coherence tomography measures correlate with brain and spinal cord atrophy and multiple sclerosis disease-related disability. Eur J Neurol. 2020;27(11):2225-32.

230. Voskuhl RR, Patel K, Paul F, Gold SM, Scheel M, Kuchling J, et al. Sex differences in brain atrophy in multiple sclerosis. Biol Sex Differ. 2020;11(1):49.

231. Weier K, Mazraeh J, Naegelin Y, Thoeni A, Hirsch JG, Fabbro T, et al. Biplanar MRI for the assessment of the spinal cord in multiple sclerosis. Mult Scler. 2012;18(11):1560-9.

232. Wen J, Yablonskiy DA, Luo J, Lancia S, Hildebolt C, Cross AH. Detection and quantification of regional cortical gray matter damage in multiple sclerosis utilizing gradient echo MRI. Neuroimage Clin. 2015;9:164-75.

233. Wetter NC, Hubbard EA, Motl RW, Sutton BP. Fully automated open-source lesion mapping of T2-FLAIR images with FSL correlates with clinical disability in MS. Brain Behav. 2016;6(3):e00440.

234. Xiang B, Wen J, Cross AH, Yablonskiy DA. Single scan quantitative gradient recalled echo MRI for evaluation of tissue damage in lesions and normal appearing gray and white matter in multiple sclerosis. J Magn Reson Imaging. 2019;49(2):487-98.

235. Xiang B, Wen J, Schmidt RE, Sukstanskii AL, Mamah D, Yablonskiy DA, et al. Evaluating brain damage in multiple sclerosis with simultaneous multi-angular-relaxometry of tissue. Ann Clin Transl Neurol. 2022;9(10):1514-27.

236. Yaldizli Ö, Penner IK, Frontzek K, Naegelin Y, Amann M, Papadopoulou A, et al. The relationship between total and regional corpus callosum atrophy, cognitive impairment and fatigue in multiple sclerosis patients. Mult Scler. 2014;20(3):356-64.

237. Yarnykh VL, Bowen JD, Samsonov A, Repovic P, Mayadev A, Qian P, et al. Fast whole-brain three-dimensional macromolecular proton fraction mapping in multiple sclerosis. Radiology. 2015;274(1):210-20.

238. Yarnykh VL, Krutenkova EP, Aitmagambetova G, Repovic P, Mayadev A, Qian P, et al. Iron-Insensitive Quantitative Assessment of Subcortical Gray Matter Demyelination in Multiple Sclerosis Using the Macromolecular Proton Fraction. AJNR Am J Neuroradiol. 2018;39(4):618-25.

239. Yousuf F, Kim G, Tauhid S, Glanz BI, Chu R, Tummala S, et al. The Contribution of Cortical Lesions to a Composite MRI Scale of Disease Severity in Multiple Sclerosis. Front Neurol. 2016;7:99.

240. Zacharzewska-Gondek A, Pokryszko-Dragan A, Gondek TM, Kołtowska A, Gruszka E, Budrewicz S, et al. Apparent diffusion coefficient measurements in normal appearing white matter may support the differential diagnosis between multiple sclerosis lesions and other white matter hyperintensities. J Neurol Sci. 2019;397:24-30.

241. Zhang L, Wen B, Chen T, Tian H, Xue H, Ren H, et al. A comparison study of inhomogeneous magnetization transfer (ihMT) and magnetization transfer (MT) in multiple sclerosis based on whole brain acquisition at 3.0 T. Magn Reson Imaging. 2020;70:43-9.

242. Zhang Y, Zabad R, Wei X, Metz L, Hill M, Mitchell J. Deep grey matter "black T2" on 3 tesla magnetic resonance imaging correlates with disability in multiple sclerosis. Mult Scler. 2007;13(7):880-3.

243. Zhou F, Zhuang Y, Wu L, Zhang N, Zeng X, Gong H, et al. Increased thalamic intrinsic oscillation amplitude in relapsing-remitting multiple sclerosis associated with the slowed cognitive processing. Clin Imaging. 2014;38(5):605-10.

244. Zhu Q, Zheng Q, Luo D, Peng Y, Yan Z, Wang X, et al. The Application of Diffusion Kurtosis Imaging on the Heterogeneous White Matter in Relapsing-Remitting Multiple Sclerosis. Front Neurosci. 2022;16:849425.

245. Zivadinov R, De Masi R, Nasuelli D, Bragadin LM, Ukmar M, Pozzi-Mucelli RS, et al. MRI techniques and cognitive impairment in the early phase of relapsing-remitting multiple sclerosis. Neuroradiology. 2001;43(4):272-8.

246. Zurawski J, Glanz BI, Healy BC, Tauhid S, Khalid F, Chitnis T, et al. The impact of cervical spinal cord atrophy on quality of life in multiple sclerosis. J Neurol Sci. 2019;403:38-43.

247. Nygaard GO, de Rodez Benavent SA, Harbo HF, Laeng B, Sowa P, Damangir S, et al. Eye and hand motor interactions with the Symbol Digit Modalities Test in early multiple sclerosis. Mult Scler Relat Disord. 2015;4(6):585-9.

248. ErtaŞoĞlu Toydemİr H, GÖkyİĞİt M, Kiymaz Seleker F, GÜndoĞdu Çelebİ L, Uysal E, BaŞak M. Long-latency Reflexes and Area Measurements of Corpus Callosum in Patients with Multiple Sclerosis. Bezmialem Science. 2016;4(1).

249. Pitombeira MS, Koole M, Campanholo KR, Souza AM, Duran FLS, Solla DJF, et al. Innate immune cells and myelin profile in multiple sclerosis: a multi-tracer PET/MR study. Eur J Nucl Med Mol Imaging. 2022;49(13):4551-66.

250. Hayton T, Furby J, Smith KJ, Altmann DR, Brenner R, Chataway J, et al. Clinical and imaging correlates of the multiple sclerosis impact scale in secondary progressive multiple sclerosis. J Neurol. 2012;259(2):237-45.

251. Griffin CM, Chard DT, Ciccarelli O, Kapoor B, Barker GJ, Thompson AI, et al. Diffusion tensor imaging in early relapsing-remitting multiple sclerosis. Mult Scler. 2001;7(5):290-7.

252. Dehmeshki J, Ruto AC, Arridge S, Silver NC, Miller DH, Tofts PS. Analysis of MTR histograms in multiple sclerosis using principal components and multiple discriminant analysis. Magn Reson Med. 2001;46(3):600-9.

253. Kalkers NF, Hintzen RQ, van Waesberghe JH, Lazeron RH, van Schijndel RA, Adèr HJ, et al. Magnetization transfer histogram parameters reflect all dimensions of MS pathology, including atrophy. J Neurol Sci. 2001;184(2):155-62.

254. Davies GR, Altmann DR, Rashid W, Chard DT, Griffin CM, Barker GJ, et al. Emergence of thalamic magnetization transfer ratio abnormality in early relapsing-remitting multiple sclerosis. Mult Scler. 2005;11(3):276-81.

255. Laule C, Vavasour IM, Zhao Y, Traboulsee AL, Oger J, Vavasour JD, et al. Two-year study of cervical cord volume and myelin water in primary progressive multiple sclerosis. Mult Scler. 2010;16(6):670-7.

256. Lazeron RH, Langdon DW, Filippi M, van Waesberghe JH, Stevenson VL, Boringa JB, et al. Neuropsychological impairment in multiple sclerosis patients: the role of (juxta)cortical lesion on FLAIR. Mult Scler. 2000;6(4):280-5.

257. Lee CY, Mak HK, Chiu PW, Chang HC, Barkhof F, Chan KH. Differential brainstem atrophy patterns in multiple sclerosis and neuromyelitis optica spectrum disorders. J Magn Reson Imaging. 2018;47(6):1601-9.

258. Lema A, Bishop C, Malik O, Mattoscio M, Ali R, Nicholas R, et al. A Comparison of Magnetization Transfer Methods to Assess Brain and Cervical Cord Microstructure in Multiple Sclerosis. J Neuroimaging. 2017;27(2):221-6.

259. Lin X, Blumhardt LD, Constantinescu CS. The relationship of brain and cervical cord volume to disability in clinical subtypes of multiple sclerosis: a three-dimensional MRI study. Acta Neurol Scand. 2003;108(6):401-6.

260. Liptak Z, Berger AM, Sampat MP, Charil A, Felsovalyi O, Healy BC, et al. Medulla oblongata volume: a biomarker of spinal cord damage and disability in multiple sclerosis. AJNR Am J Neuroradiol. 2008;29(8):1465-70.

261. Liu Y, Wang J, Daams M, Weiler F, Hahn HK, Duan Y, et al. Differential patterns of spinal cord and brain atrophy in NMO and MS. Neurology. 2015;84(14):1465-72.

262. Ljubisavljevic S, Stojanovic I, Cvetkovic T, Vojinovic S, Stojanov D, Stojanovic D, et al. Erythrocytes' antioxidative capacity as a potential marker of oxidative stress intensity in neuroinflammation. J Neurol Sci. 2014;337(1-2):8-13.

263. Locatelli L, Zivadinov R, Grop A, Zorzon M. Frontal parenchymal atrophy measures in multiple sclerosis. Mult Scler. 2004;10(5):562-8.

264. Lund H, Jønsson A, Andresen J, Rostrup E, Paulson OB, Sørensen PS. Cognitive deficits in multiple sclerosis: correlations with T2 changes in normal appearing brain tissue. Acta Neurol Scand. 2012;125(5):338-44.

265. Maarouf A, Audoin B, Gherib S, El Mendili MM, Viout P, Pariollaud F, et al. Grey-matter sodium concentration as an individual marker of multiple sclerosis severity. Mult Scler. 2022;28(12):1903-12.

266. MacKenzie-Graham A, Kurth F, Itoh Y, Wang HJ, Montag MJ, Elashoff R, et al. Disability-Specific Atlases of Gray Matter Loss in Relapsing-Remitting Multiple Sclerosis. JAMA Neurol. 2016;73(8):944-53.

267. Magon S, Chakravarty MM, Amann M, Weier K, Naegelin Y, Andelova M, et al. Label-fusion-segmentation and deformation-based shape analysis of deep gray matter in multiple sclerosis: the impact of thalamic subnuclei on disability. Hum Brain Mapp. 2014;35(8):4193-203.

268. Mainero C, De Stefano N, Iannucci G, Sormani M, Guidi L, Federico A, et al. Correlates of MS disability assessed in vivo using aggregates of MR quantities. Neurology. 2001;56(10):1331-4.

269. Minneboo A, Jasperse B, Barkhof F, Uitdehaag BM, Knol DL, de Groot V, et al. Predicting short-term disability progression in early multiple sclerosis: added value of MRI parameters. J Neurol Neurosurg Psychiatry. 2008;79(8):917-23.

270. Molyneux P, Barker G, Barkhof F, Beckmann K, Dahlke F, Filippi M, et al. Clinical–MRI correlations in a European trial of interferon beta-1b in secondary progressive MS. Neurology. 2001;57(12):2191-7.

271. Motl RW, Hubbard EA, Sreekumar N, Wetter NC, Sutton BP, Pilutti LA, et al. Pallidal and caudate volumes correlate with walking function in multiple sclerosis. J Neurol Sci. 2015;354(1-2):33-6.

272. Motl RW, Zivadinov R, Bergsland N, Benedict RH. Thalamus volume and ambulation in multiple sclerosis: a cross-sectional study. Neurodegenerative disease management. 2016;6(1):23-9.

273. Nakamura Y, Liu Z, Fukumoto S, Shinoda K, Sakoda A, Matsushita T, et al. Spinal cord involvement by atrophy and associations with disability are different between multiple sclerosis and neuromyelitis optica spectrum disorder. Eur J Neurol. 2020;27(1):92-9.

274. Nielsen AS, Kinkel RP, Madigan N, Tinelli E, Benner T, Mainero C. Contribution of cortical lesion subtypes at 7T MRI to physical and cognitive performance in MS. Neurology. 2013;81(7):641-9.

275. Nielsen JF, Bech E, Gadeberg P, Sinkjaer T. No correlation between number of MRI-evident lesions in cerebrum and the soleus stretch reflex in multiple sclerosis patients. Eur J Neurol. 2004;11(11):760-6.

276. Nijeholt GJ, van Walderveen MA, Castelijns JA, van Waesberghe JH, Polman C, Scheltens P, et al. Brain and spinal cord abnormalities in multiple sclerosis. Correlation between MRI parameters, clinical subtypes and symptoms. Brain. 1998;121 ( Pt 4):687-97.

277. Oh J, Seigo M, Saidha S, Sotirchos E, Zackowski K, Chen M, et al. Spinal cord normalization in multiple sclerosis. J Neuroimaging. 2014;24(6):577-84.

278. Ozturk A, Smith SA, Gordon-Lipkin EM, Harrison DM, Shiee N, Pham DL, et al. MRI of the corpus callosum in multiple sclerosis: association with disability. Mult Scler. 2010;16(2):166-77.

279. OZTURK O, OZTURK S, GUL S, BUNUL SD, EFENDI H, CAM I. The relationship between cerebellar volume, clinical disability and cognitive changes in multiple sclerosis patients. Neurology Asia. 2021;26(2).

280. Paolillo A, Pozzilli C, Giugni E, Tomassini V, Gasperini C, Fiorelli M, et al. A 6-year clinical and MRI follow-up study of patients with relapsing-remitting multiple sclerosis treated with Interferon-beta. Eur J Neurol. 2002;9(6):645-55.

281. Papadopoulou A, Müller-Lenke N, Naegelin Y, Kalt G, Bendfeldt K, Kuster P, et al. Contribution of cortical and white matter lesions to cognitive impairment in multiple sclerosis. Mult Scler. 2013;19(10):1290-6.

282. Pardini M, Yaldizli Ö, Sethi V, Muhlert N, Liu Z, Samson RS, et al. Motor network efficiency and disability in multiple sclerosis. Neurology. 2015;85(13):1115-22.

283. Pichler A, Enzinger C, Fuchs S, Plecko-Startinig B, Gruber-Sedlmayr U, Linortner P, et al. Differences and similarities in the evolution of morphologic brain abnormalities between paediatric and adult-onset multiple sclerosis. Mult Scler. 2013;19(2):167-72.

284. Piras MR, Magnano I, Canu ED, Paulus KS, Satta WM, Soddu A, et al. Longitudinal study of cognitive dysfunction in multiple sclerosis: neuropsychological, neuroradiological, and neurophysiological findings. J Neurol Neurosurg Psychiatry. 2003;74(7):878-85.

285. Poonawalla AH, Datta S, Juneja V, Nelson F, Wolinsky JS, Cutter G, et al. Composite MRI scores improve correlation with EDSS in multiple sclerosis. Mult Scler. 2010;16(9):1117-25.

286. Quattrocchi CC, Cherubini A, Luccichenti G, Grasso MG, Nocentini U, Beomonte Zobel B, et al. Infratentorial lesion volume correlates with sensory functional system in multiple sclerosis patients: a 3.0-Tesla MRI study. Radiol Med. 2010;115(1):115-24.

287. Ramió-Torrentà L, Sastre-Garriga J, Ingle GT, Davies GR, Ameen V, Miller DH, et al. Abnormalities in normal appearing tissues in early primary progressive multiple sclerosis and their relation to disability: a tissue specific magnetisation transfer study. J Neurol Neurosurg Psychiatry. 2006;77(1):40-5.

288. Rashid W, Hadjiprocopis A, Griffin CM, Chard DT, Davies GR, Barker GJ, et al. Diffusion tensor imaging of early relapsing-remitting multiple sclerosis with histogram analysis using automated segmentation and brain volume correction. Mult Scler. 2004;10(1):9-15.

289. Riahi F, Zijdenbos A, Narayanan S, Arnold D, Francis G, Antel J, et al. Improved correlation between scores on the expanded disability status scale and cerebral lesion load in relapsing-remitting multiple sclerosis. Results of the application of new imaging methods. Brain. 1998;121 ( Pt 7):1305-12.

290. Roosendaal SD, Bendfeldt K, Vrenken H, Polman CH, Borgwardt S, Radue EW, et al. Grey matter volume in a large cohort of MS patients: relation to MRI parameters and disability. Mult Scler. 2011;17(9):1098-106.

291. Rovaris M, Judica E, Sastre-Garriga J, Rovira A, Sormani MP, Benedetti B, et al. Large-scale, multicentre, quantitative MRI study of brain and cord damage in primary progressive multiple sclerosis. Mult Scler. 2008;14(4):455-64.

292. Rovaris M, Rocca MA, Barkhof F, Calabrese M, De Stefano N, Khalil M, et al. Relationship between brain MRI lesion load and short-term disease evolution in non-disabling MS: a large-scale, multicentre study. Mult Scler. 2011;17(3):319-26.

293. Rudick RA, Lee JC, Simon J, Fisher E. Significance of T2 lesions in multiple sclerosis: A 13-year longitudinal study. Ann Neurol. 2006;60(2):236-42.

294. Rudick RA, Lee JC, Nakamura K, Fisher E. Gray matter atrophy correlates with MS disability progression measured with MSFC but not EDSS. J Neurol Sci. 2009;282(1-2):106-11.

295. Ruggieri S, Petracca M, Miller A, Krieger S, Ghassemi R, Bencosme Y, et al. Association of Deep Gray Matter Damage With Cortical and Spinal Cord Degeneration in Primary Progressive Multiple Sclerosis. JAMA Neurol. 2015;72(12):1466-74.

296. Rusz J, Vaneckova M, Benova B, Tykalova T, Novotny M, Ruzickova H, et al. Brain volumetric correlates of dysarthria in multiple sclerosis. Brain Lang. 2019;194:58-64.

297. Sailer M, Fischl B, Salat D, Tempelmann C, Schönfeld MA, Busa E, et al. Focal thinning of the cerebral cortex in multiple sclerosis. Brain. 2003;126(Pt 8):1734-44.

298. Samson RS, Muhlert N, Sethi V, Wheeler-Kingshott CA, Ron MA, Miller DH, et al. Sulcal and gyral crown cortical grey matter involvement in multiple sclerosis: A magnetisation transfer ratio study. Mult Scler Relat Disord. 2013;2(3):204-12.

299. Sanfilipo MP, Benedict RH, Sharma J, Weinstock-Guttman B, Bakshi R. The relationship between whole brain volume and disability in multiple sclerosis: a comparison of normalized gray vs. white matter with misclassification correction. Neuroimage. 2005;26(4):1068-77.

300. Saraste M, Bezukladova S, Matilainen M, Tuisku J, Rissanen E, Sucksdorff M, et al. High serum neurofilament associates with diffuse white matter damage in MS. Neurol Neuroimmunol Neuroinflamm. 2021;8(1).

301. Sastre-Garriga J, Ingle GT, Chard DT, Ramió-Torrentà L, Miller DH, Thompson AJ. Grey and white matter atrophy in early clinical stages of primary progressive multiple sclerosis. Neuroimage. 2004;22(1):353-9.

302. Schlaeger R, Papinutto N, Panara V, Bevan C, Lobach IV, Bucci M, et al. Spinal cord gray matter atrophy correlates with multiple sclerosis disability. Ann Neurol. 2014;76(4):568-80.

303. Schlaeger R, Papinutto N, Zhu AH, Lobach IV, Bevan CJ, Bucci M, et al. Association Between Thoracic Spinal Cord Gray Matter Atrophy and Disability in Multiple Sclerosis. JAMA Neurol. 2015;72(8):897-904.

304. Shiee N, Bazin PL, Zackowski KM, Farrell SK, Harrison DM, Newsome SD, et al. Revisiting brain atrophy and its relationship to disability in multiple sclerosis. PLoS One. 2012;7(5):e37049.

305. Song F, Huan Y, Yin H, Ge Y, Wei G, Chang Y, et al. Normalized upper cervical spinal cord atrophy in multiple sclerosis. J Neuroimaging. 2008;18(3):320-7.

306. Sormani MP, Rovaris M, Comi G, Filippi M. A reassessment of the plateauing relationship between T2 lesion load and disability in MS. Neurology. 2009;73(19):1538-42.

307. Sowa P, Harbo HF, White NS, Celius EG, Bartsch H, Berg-Hansen P, et al. Restriction spectrum imaging of white matter and its relation to neurological disability in multiple sclerosis. Multiple Sclerosis Journal. 2019;25(5):687-98.

308. Stankiewicz JM, Glanz BI, Healy BC, Arora A, Neema M, Benedict RH, et al. Brain MRI lesion load at 1.5T and 3T versus clinical status in multiple sclerosis. J Neuroimaging. 2011;21(2):e50-6.

309. Tam RC, Traboulsee A, Riddehough A, Sheikhzadeh F, Li DK. The impact of intensity variations in T1-hypointense lesions on clinical correlations in multiple sclerosis. Mult Scler. 2011;17(8):949-57.

310. Tartaglia MC, Narayanan S, Francis SJ, Santos AC, De Stefano N, Lapierre Y, et al. The relationship between diffuse axonal damage and fatigue in multiple sclerosis. Arch Neurol. 2004;61(2):201-7.

311. Tauhid S, Chu R, Sasane R, Glanz BI, Neema M, Miller JR, et al. Brain MRI lesions and atrophy are associated with employment status in patients with multiple sclerosis. J Neurol. 2015;262(11):2425-32.

312. Tavazzi E, Dwyer MG, Weinstock-Guttman B, Lema J, Bastianello S, Bergamaschi R, et al. Quantitative diffusion weighted imaging measures in patients with multiple sclerosis. Neuroimage. 2007;36(3):746-54.

313. Tjoa CW, Benedict RH, Weinstock-Guttman B, Fabiano AJ, Bakshi R. MRI T2 hypointensity of the dentate nucleus is related to ambulatory impairment in multiple sclerosis. J Neurol Sci. 2005;234(1-2):17-24.

314. Tovar-Moll F, Evangelou IE, Chiu AW, Auh S, Chen C, Ehrmantraut M, et al. Diffuse and focal corticospinal tract disease and its impact on patient disability in multiple sclerosis. J Neuroimaging. 2015;25(2):200-6.

315. Traboulsee A, Dehmeshki J, Peters KR, Griffin CM, Brex PA, Silver N, et al. Disability in multiple sclerosis is related to normal appearing brain tissue MTR histogram abnormalities. Mult Scler. 2003;9(6):566-73.

316. Trufanov A, Bisaga G, Skulyabin D, Temniy A, Poplyak M, Chakchir O, et al. Thalamic nuclei degeneration in multiple sclerosis. J Clin Neurosci. 2021;89:375-80.

317. Tur C, Khaleeli Z, Ciccarelli O, Altmann DR, Cercignani M, Miller DH, et al. Complementary roles of grey matter MTR and T2 lesions in predicting progression in early PPMS. J Neurol Neurosurg Psychiatry. 2011;82(4):423-8.

318. Uher T, Krasensky J, Sobisek L, Blahova Dusankova J, Seidl Z, Kubala Havrdova E, et al. Cognitive clinico-radiological paradox in early stages of multiple sclerosis. Ann Clin Transl Neurol. 2018;5(1):81-91.

319. Vaithianathar L, Tench CR, Morgan PS, Constantinescu CS. Magnetic resonance imaging of the cervical spinal cord in multiple sclerosis--a quantitative T1 relaxation time mapping approach. J Neurol. 2003;250(3):307-15.

320. Varoğlu AO, Odaci E, Gumus H, Keles ON, Unal B, Deniz O. Evaluation of patients with multiple sclerosis using a combination of morphometrical features and clinical scores. J Clin Neurosci. 2010;17(2):191-5.

321. Vellinga MM, Geurts JJ, Rostrup E, Uitdehaag BM, Polman CH, Barkhof F, et al. Clinical correlations of brain lesion distribution in multiple sclerosis. J Magn Reson Imaging. 2009;29(4):768-73.

322. Wilson M, Morgan PS, Lin X, Turner BP, Blumhardt LD. Quantitative diffusion weighted magnetic resonance imaging, cerebral atrophy, and disability in multiple sclerosis. J Neurol Neurosurg Psychiatry. 2001;70(3):318-22.

323. Wilson M, Tench CR, Morgan PS, Blumhardt LD. Pyramidal tract mapping by diffusion tensor magnetic resonance imaging in multiple sclerosis: improving correlations with disability. J Neurol Neurosurg Psychiatry. 2003;74(2):203-7.

324. Wu GF, Schwartz ED, Lei T, Souza A, Mishra S, Jacobs DA, et al. Relation of vision to global and regional brain MRI in multiple sclerosis. Neurology. 2007;69(23):2128-35.

325. Wuschek A, Bussas M, El Husseini M, Harabacz L, Pineker V, Pongratz V, et al. Somatosensory evoked potentials and magnetic resonance imaging of the central nervous system in early multiple sclerosis. J Neurol. 2023;270(2):824-30.

326. Yaldizli O, Atefy R, Gass A, Sturm D, Glassl S, Tettenborn B, et al. Corpus callosum index and long-term disability in multiple sclerosis patients. J Neurol. 2010;257(8):1256-64.

327. Yamamoto T, Mori M, Uzawa A, Uchiyama T, Sakakibara R, Yanagisawa M, et al. Urinary symptoms and neurological disabilities are differentially correlated between multiple sclerosis and neuromyelitis optica. Clinical and Experimental Neuroimmunology. 2016;7(1):52-8.

328. Yousuf F, Dupuy SL, Tauhid S, Chu R, Kim G, Tummala S, et al. A two-year study using cerebral gray matter volume to assess the response to fingolimod therapy in multiple sclerosis. J Neurol Sci. 2017;383:221-9.

329. Zivadinov R, Bagnato F, Nasuelli D, Bastianello S, Bratina A, Locatelli L, et al. Short-term brain atrophy changes in relapsing-remitting multiple sclerosis. J Neurol Sci. 2004;223(2):185-93.

330. Zivadinov R, Banas AC, Yella V, Abdelrahman N, Weinstock-Guttman B, Dwyer MG. Comparison of three different methods for measurement of cervical cord atrophy in multiple sclerosis. AJNR Am J Neuroradiol. 2008;29(2):319-25.

331. Zivadinov R, Locatelli L, Stival B, Bratina A, Grop A, Nasuelli D, et al. Normalized regional brain atrophy measurements in multiple sclerosis. Neuroradiology. 2003;45(11):793-8.

332. Li DK, Held U, Petkau J, Daumer M, Barkhof F, Fazekas F, et al. MRI T2 lesion burden in multiple sclerosis: a plateauing relationship with clinical disability. Neurology. 2006;66(9):1384-9.

333. Papini GDE, Di Leo G, Zanardo M, Fedeli MP, Merli I, Sardanelli F. Measurement of jugular foramen diameter using MRI in multiple sclerosis patients compared to control subjects. Eur Radiol Exp. 2017;1(1):4.

334. Barreiro-González A, Sanz MT, Carratalà-Boscà S, Pérez-Miralles F, Alcalá C, Carreres-Polo J, et al. Design and Validation of an Expanded Disability Status Scale Model in Multiple Sclerosis. Eur Neurol. 2022;85(2):112-21.

335. Kalkers NF, Bergers L, de Groot V, Lazeron RH, van Walderveen MA, Uitdehaag BM, et al. Concurrent validity of the MS Functional Composite using MRI as a biological disease marker. Neurology. 2001;56(2):215-9.

336. Bross M, Hackett M, Bernitsas MM, Bao F, Carla Santiago M, Bernitsas E. Cortical surface thickness, subcortical volumes and disability between races in relapsing-remitting multiple sclerosis. Mult Scler Relat Disord. 2021;53:103025.

337. Clarke MA, Archer D, Yoon K, Oguz I, Smith SA, Xu J, et al. White matter tracts that overlap with the thalamus and the putamen are protected against multiple sclerosis pathology. Mult Scler Relat Disord. 2022;57:103430.

338. Coffman CH, White R, Subramanian K, Buch S, Bernitsas E, Haacke EM. Quantitative susceptibility mapping of both ring and non-ring white matter lesions in relapsing remitting multiple sclerosis. Magn Reson Imaging. 2022;91:45-51.

339. Filippi P, Vestenická V, Siarnik P, Sivakova M, Čopíková-Cudráková D, Belan V, et al. Neurofilament light chain and MRI volume parameters as markers of neurodegeneration in multiple sclerosis. Neuro Endocrinol Lett. 2020;41(1):17-26.

340. Lukas C, Sombekke MH, Bellenberg B, Hahn HK, Popescu V, Bendfeldt K, et al. Relevance of spinal cord abnormalities to clinical disability in multiple sclerosis: MR imaging findings in a large cohort of patients. Radiology. 2013;269(2):542-52.

341. Zivadinov R, Grop A, Sharma J, Bratina A, Tjoa CW, Dwyer M, et al. Reproducibility and accuracy of quantitative magnetic resonance imaging techniques of whole-brain atrophy measurement in multiple sclerosis. J Neuroimaging. 2005;15(1):27-36.

342. Cappelle S, Pareto D, Tintoré M, Vidal-Jordana A, Alyafeai R, Alberich M, et al. A validation study of manual atrophy measures in patients with Multiple Sclerosis. Neuroradiology. 2020;62(8):955-64.

343. O'Connor P, Marchetti P, Lee L, Perera M. Evoked potential abnormality scores are a useful measure of disease burden in relapsing-remitting multiple sclerosis. Ann Neurol. 1998;44(3):404-7.

344. Spampinato MV, Kocher MR, Jensen JH, Helpern JA, Collins HR, Hatch NU. Diffusional Kurtosis Imaging of the Corticospinal Tract in Multiple Sclerosis: Association with Neurologic Disability. AJNR Am J Neuroradiol. 2017;38(8):1494-500.

345. Simon JH, Jacobs LD, Campion MK, Rudick RA, Cookfair DL, Herndon RM, et al. A longitudinal study of brain atrophy in relapsing multiple sclerosis. The Multiple Sclerosis Collaborative Research Group (MSCRG). Neurology. 1999;53(1):139-48.

346. Madsen MAJ, Wiggermann V, Marques MFM, Lundell H, Cerri S, Puonti O, et al. Linking lesions in sensorimotor cortex to contralateral hand function in multiple sclerosis: a 7 T MRI study. Brain. 2022;145(10):3522-35.

347. Giugni E, Pozzilli C, Bastianello S, Gasperini C, Paolillo A, Koudriavtseva T, et al. MRI measures and their relations with clinical disability in relapsing-remitting and secondary progressive multiple sclerosis. Mult Scler. 1997;3(4):221-5.

348. Hasan KM, Walimuni IS, Abid H, Datta S, Wolinsky JS, Narayana PA. Human brain atlas-based multimodal MRI analysis of volumetry, diffusimetry, relaxometry and lesion distribution in multiple sclerosis patients and healthy adult controls: implications for understanding the pathogenesis of multiple sclerosis and consolidation of quantitative MRI results in MS. J Neurol Sci. 2012;313(1-2):99-109.

349. Iannucci G, Minicucci L, Rodegher M, Sormani MP, Comi G, Filippi M. Correlations between clinical and MRI involvement in multiple sclerosis: assessment using T(1), T(2) and MT histograms. J Neurol Sci. 1999;171(2):121-9.

350. Maghzi AH, Revirajan N, Julian LJ, Spain R, Mowry EM, Liu S, et al. Magnetic resonance imaging correlates of clinical outcomes in early multiple sclerosis. Mult Scler Relat Disord. 2014;3(6):720-7.

351. Hasan KM, Halphen C, Kamali A, Nelson FM, Wolinsky JS, Narayana PA. Caudate nuclei volume, diffusion tensor metrics, and T(2) relaxation in healthy adults and relapsing-remitting multiple sclerosis patients: implications for understanding gray matter degeneration. J Magn Reson Imaging. 2009;29(1):70-7.

352. Boaventura M, Sastre-Garriga J, Garcia-Vidal A, Vidal-Jordana A, Quartana D, Carvajal R, et al. T1/T2-weighted ratio in multiple sclerosis: A longitudinal study with clinical associations. Neuroimage Clin. 2022;34:102967.

353. Mammi S, Filippi M, Martinelli V, Campi A, Colombo B, Scotti G, et al. Correlation between brain MRI lesion volume and disability in patients with multiple sclerosis. Acta Neurol Scand. 1996;94(2):93-6.

354. Miki Y, Grossman RI, Udupa JK, Wei L, Polansky M, Mannon LJ, et al. Relapsing-remitting multiple sclerosis: longitudinal analysis of MR images--lack of correlation between changes in T2 lesion volume and clinical findings. Radiology. 1999;213(2):395-9.

355. Molyneux PD, Filippi M, Barkhof F, Gasperini C, Yousry TA, Truyen L, et al. Correlations between monthly enhanced MRI lesion rate and changes in T2 lesion volume in multiple sclerosis. Ann Neurol. 1998;43(3):332-9.

356. Rizkallah M, Hefida M, Khalil M, Dawoud RM. Automated quantification of deep grey matter structures and white matter lesions using magnetic resonance imaging in relapsing remission multiple sclerosis. Egyptian Journal of Radiology and Nuclear Medicine. 2021;52:1-17.

357. Yokote H, Toru S, Nishida Y, Hattori T, Sanjo N, Yokota T. Serum amyloid A level correlates with T2 lesion volume and cortical volume in patients with multiple sclerosis. J Neuroimmunol. 2021;351:577466.

358. Bateman GA, Lechner-Scott J, Carey MF, Bateman AR, Lea RA. Possible Markers of Venous Sinus Pressure Elevation in Multiple Sclerosis: Correlations with Gender and Disease Progression. Mult Scler Relat Disord. 2021;55:103207.

359. Till C, Ghassemi R, Aubert-Broche B, Kerbrat A, Collins DL, Narayanan S, et al. MRI correlates of cognitive impairment in childhood-onset multiple sclerosis. Neuropsychology. 2011;25(3):319-32.

360. Yokote H, Kamata T, Toru S, Sanjo N, Yokota T. Serum retinol levels are associated with brain volume loss in patients with multiple sclerosis. Mult Scler J Exp Transl Clin. 2017;3(3):2055217317729688.

361. Zhang Y, Metz LM, Yong VW, Mitchell JR. 3T deep gray matter T2 hypointensity correlates with disability over time in stable relapsing-remitting multiple sclerosis: a 3-year pilot study. J Neurol Sci. 2010;297(1-2):76-81.

362. Pareto D, Garcia-Vidal A, Alberich M, Auger C, Montalban X, Tintoré M, et al. Ratio of T1-Weighted to T2-Weighted Signal Intensity as a Measure of Tissue Integrity: Comparison with Magnetization Transfer Ratio in Patients with Multiple Sclerosis. AJNR Am J Neuroradiol. 2020;41(3):461-3.

363. Ge Y, Grossman RI, Udupa JK, Babb JS, Nyúl LG, Kolson DL. Brain atrophy in relapsing-remitting multiple sclerosis: fractional volumetric analysis of gray matter and white matter. Radiology. 2001;220(3):606-10.

364. Bergsland N, Horakova D, Dwyer MG, Dolezal O, Seidl ZK, Vaneckova M, et al. Subcortical and Cortical Gray Matter Atrophy in a Large Sample of Patients with Clinically Isolated Syndrome and Early Relapsing-Remitting Multiple Sclerosis. American Journal of Neuroradiology. 2012;33(8):1573-8.

365. Parry A, Clare S, Jenkinson M, Smith S, Palace J, Matthews PM. White matter and lesion T1 relaxation times increase in parallel and correlate with disability in multiple sclerosis. J Neurol. 2002;249(9):1279-86.

366. Rudko DA, Solovey I, Gati JS, Kremenchutzky M, Menon RS. Multiple sclerosis: improved identification of disease-relevant changes in gray and white matter by using susceptibility-based MR imaging. Radiology. 2014;272(3):851-64.

367. Akaishi T, Nakashima I, Mugikura S, Aoki M, Fujihara K. Whole brain and grey matter volume of Japanese patients with multiple sclerosis. J Neuroimmunol. 2017;306:68-75.

368. Harrison DM, Oh J, Roy S, Wood ET, Whetstone A, Seigo MA, et al. Thalamic lesions in multiple sclerosis by 7T MRI: Clinical implications and relationship to cortical pathology. Mult Scler. 2015;21(9):1139-50.

369. Edwards SG, Liu C, Blumhardt LD. Cognitive correlates of supratentorial atrophy on MRI in multiple sclerosis. Acta Neurol Scand. 2001;104(4):214-23.

370. Houtchens MK, Benedict RH, Killiany R, Sharma J, Jaisani Z, Singh B, et al. Thalamic atrophy and cognition in multiple sclerosis. Neurology. 2007;69(12):1213-23.

371. Thaler C, Hartramph I, Stellmann JP, Heesen C, Bester M, Fiehler J, et al. T1 Relaxation Times in the Cortex and Thalamus Are Associated With Working Memory and Information Processing Speed in Patients With Multiple Sclerosis. Front Neurol. 2021;12:789812.

372. Lashkari A, Davoodi-Bojd E, Fahmy L, Li L, Nejad-Davarani SP, Chopp M, et al. Impairments of white matter tracts and connectivity alterations in five cognitive networks of patients with multiple sclerosis. Clinical Neurology and Neurosurgery. 2021;201:106424.

373. Grimaud J, Barker GJ, Wang L, Lai M, MacManus DG, Webb SL, et al. Correlation of magnetic resonance imaging parameters with clinical disability in multiple sclerosis: a preliminary study. J Neurol. 1999;246(10):961-7.

374. Simon JH, Lull J, Jacobs LD, Rudick RA, Cookfair DL, Herndon RM, et al. A longitudinal study of T1 hypointense lesions in relapsing MS: MSCRG trial of interferon beta-1a. Multiple Sclerosis Collaborative Research Group. Neurology. 2000;55(2):185-92.

375. Cader S, Johansen-Berg H, Wylezinska M, Palace J, Behrens TE, Smith S, et al. Discordant white matter N-acetylasparate and diffusion MRI measures suggest that chronic metabolic dysfunction contributes to axonal pathology in multiple sclerosis. Neuroimage. 2007;36(1):19-27.

376. Matthews PM, Pioro E, Narayanan S, De Stefano N, Fu L, Francis G, et al. Assessment of lesion pathology in multiple sclerosis using quantitative MRI morphometry and magnetic resonance spectroscopy. Brain. 1996;119 ( Pt 3):715-22.

377. Kappos L, Städt D, Ratzka M, Keil W, Schneiderbanger-Grygier S, Heitzer T, et al. Magnetic resonance imaging in the evaluation of treatment in multiple sclerosis. Neuroradiology. 1988;30(4):299-302.

378. Chiang FL, Feng M, Romero RS, Price L, Franklin CG, Deng S, et al. Disruption of the Atrophy-based Functional Network in Multiple Sclerosis Is Associated with Clinical Disability: Validation of a Meta-Analytic Model in Resting-State Functional MRI. Radiology. 2021;299(1):159-66.

379. de la Peña MJ, Peña IC, García PG, Gavilán ML, Malpica N, Rubio M, et al. Early perfusion changes in multiple sclerosis patients as assessed by MRI using arterial spin labeling. Acta Radiol Open. 2019;8(12):2058460119894214.

380. Narayana PA, Govindarajan KA, Goel P, Datta S, Lincoln JA, Cofield SS, et al. Regional cortical thickness in relapsing remitting multiple sclerosis: A multi-center study. Neuroimage Clin. 2012;2:120-31.

381. Morgen K, Sammer G, Courtney SM, Wolters T, Melchior H, Blecker CR, et al. Distinct mechanisms of altered brain activation in patients with multiple sclerosis. Neuroimage. 2007;37(3):937-46.

382. Ramezani N, Davanian F, Naghavi S, Riahi R, Zandieh G, Danesh-Mobarhan S, et al. Thalamic asymmetry in Multiple Sclerosis. Mult Scler Relat Disord. 2023;77:104853.

383. Tauhid S, Neema M, Healy BC, Weiner HL, Bakshi R. MRI phenotypes based on cerebral lesions and atrophy in patients with multiple sclerosis. J Neurol Sci. 2014;346(1-2):250-4.
