# Supplementary material for: Neuroimaging markers and disability scales in multiple sclerosis: A systematic review and meta-analysis
Source: PLoS One. 2024 Dec 5;19(12):e0312421. doi: 10.1371/journal.pone.0312421 (PMC11620670; doi:10.1371/journal.pone.0312421)
Supplement: S3 File — (DOCX) [file pone.0312421.s004.docx]

Supplementary 3. Results of meta-analyses of disability and MRI measurements correlation in pwMS.

Table S1. Results of meta-analyses of EDSS and MRI measurements in pwMS.

| **EDSS** | | | | | | | | | | | | | | | |
| --- | --- | --- | --- | --- | --- | --- | --- | --- | --- | --- | --- | --- | --- | --- | --- |
|  | **Overall** | | | | | **RRMS** | | | | | **PMS** | | | | |
|  | Studies | Patients | Pooled correlation coefficient | z-score | *p*-value | Studies | Patients | Pooled correlation coefficient | z-score | *p*-value | Studies | Patients | Pooled correlation coefficient | z-score | *p*-value |
| **Volume of brain structure** |  |  |  |  |  |  |  |  |  |  |  |  |  |  |  |
| BPF | 43 | 4193 | -0.37 [-0.41; -0.32] | -13.95 | <0.01 | 13 | 1811 | -0.34 [-0.40; -0.28] | -10.26 | <0.01 |  |  |  |  |  |
| Brain volume | 14 | 1179 | -0.40 [-0.47; -0.33] | -10.46 | <0.01 | 4 | 527 | 0.50 [-0.56; -0.43] | -12.49 | <0.01 |  |  |  |  |  |
| Corpus callosum area | 5 | 390 | -0.29 [-0.44; -0.13] | -3.4 | <0.01 |  |  |  |  |  |  |  |  |  |  |
| Corpus callosum index | 8 | 619 | -0.36 [-0.52; -0.17] | -3.57 | <0.01 | 3 | 185 | -0.11 [-0.43; 0.22] | -0.65 | 0.51 |  |  |  |  |  |
| Corpus callosum volume | 6 | 436 | -0.21 [-0.35; -0.06] | -2.81 | <0.01 |  |  |  |  |  |  |  |  |  |  |
| Cortical thickness | 10 | 746 | -0.33 [-0.45; -0.21] | -4.98 | <0.01 | 4 | 418 | -0.18 [-0.33; -0.02] | -2.2 | 0.02 |  |  |  |  |  |
| GMF | 15 | 1509 | -0.3 [-0.39; -0.20] | -5.84 | <0.01 | 3 | 156 | -0.19 [-0.44; 0.09] | -1.35 | 0.17 |  |  |  |  |  |
| GMV | 15 | 1024 | -0.36 [-0.41; -0.30] | -11.79 | <0.01 | 5 | 218 | -0.36 [-0.49; -0.21] | -4.67 | <0.01 |  |  |  |  |  |
| Normalized BPV | 9 | 442 | -0.22 [-0.39; -0.03] | -2.23 | 0/03 | 4 | 206 | -0.01 [-0.32; 0.30] | -0.07 | 0.94 |  |  |  |  |  |
| Normalized brain stem volume | 5 | 329 | -0.32 [-0.42; -0.22] | -5.94 | <0.01 |  |  |  |  |  |  |  |  |  |  |
| Normalized brain volume | 24 | 3003 | -0.26 [-0.33; -0.18] | -6.1 | <0.01 | 8 | 1095 | -0.38 [-0.46; -0.29] | -7.82 | <0.01 | 3 | 456 | 0.02 [-0.32; 0.35] | 0.1 | 0.91 |
| Normalized caudate volume | 9 | 372 | -0.21 [-0.31; -0.11] | -4.01 | <0.01 | 3 | 92 | -0.15 [-0.35; 0.07] | -1.34 | 0.18 |  |  |  |  |  |
| Normalized cerebellar volume | 6 | 721 | -0.23 [-0.33; -0.12] | -3.97 | <0.01 |  |  |  |  |  |  |  |  |  |  |
| Normalized corpus callosum volume | 5 | 521 | -0.19 [-0.27; -0.10] | -4.27 | <0.01 |  |  |  |  |  |  |  |  |  |  |
| Normalized cortical GMV | 10 | 1053 | -0.24 [-0.36; -0.12] | -3.78 | <0.01 |  |  |  |  |  |  |  |  |  |  |
| Normalized cortical volume | 7 | 1064 | -0.31 [-0.40; -0.20] | -5.67 | <0.01 | 4 | 349 | -0.21 [-0.31; -0.11] | -3.97 | <0.01 |  |  |  |  |  |
| Normalized GMV | 21 | 2683 | -0.23 [-0.32; -0.13] | -4.49 | <0.01 | 7 | 635 | -0.17 [-0.33; 0.00] | -1.96 | 0.5 |  |  |  |  |  |
| Normalized deep GMV | 6 | 1089 | -0.19 [-0.51; 0.18] | -0.99 | 0.32 | 4 | 841 | -0.09 [-0.55; 0.40] | -0.36 | 0.72 |  |  |  |  |  |
| Normalized globus pallidus volume | 4 | 182 | -0.26 [-0.39; -0.11] | -3.42 | <0.01 |  |  |  |  |  |  |  |  |  |  |
| Normalized putamen volume | 7 | 310 | -0.25 [-0.41; -0.08] | -2.8 | <0.01 | 3 | 90 | -0.26 [-0.45; -0.05] | 2.43 | 0.02 |  |  |  |  |  |
| Normalized thalamus volume | 20 | 2413 | -0.26 [-0.34; -0.17] | -5.96 | <0.01 | 5 | 577 | -0.31 [-0.40;-0.22] | -6.68 | <0.01 |  |  |  |  |  |
| Normalized WMV | 22 | 2658 | -0.22 [-0.29; -0.14] | -5.37 | <0.01 | 4 | 674 | -0.17 [-0.24; -0.09] | -4.38 | <0.01 |  |  |  |  |  |
| Putamen volume | 5 | 369 | -0.12 [-0.32; 0.10] | -1.1 | 0.29 |  |  |  |  |  |  |  |  |  |  |
| Thalamus volume | 7 | 1413 | -0.21 [-0.30; -0.12] | -4.45 | <0.01 | 4 | 1178 | -0.17 [-0.25; -0.09] | -4.03 | <0.01 |  |  |  |  |  |
| Third ventricular width | 12 | 961 | 0.35 [0.29; 0.40] | 10/97 | <0.01 | 3 | 409 | 0.34 [0.21; 0.46] | 5 | <0.01 |  |  |  |  |  |
| WMF | 12 | 1082 | -0.21 [-0.44; 0.04] | -1.66 | 0.1 | 3 | 156 | -0.02 [-0.18; 0.14] | -0.29 | 0.77 |  |  |  |  |  |
| WMV | 9 | 483 | -0.25 [-0.36; -0.14] | -4/39 | <0.01 | 4 | 169 | -0.27 [-0.43; -0.09] | -2.96 | <0.01 |  |  |  |  |  |
| **Lesion of brain structure** |  |  |  |  |  |  |  |  |  |  |  |  |  |  |  |
| Brain lesion count | 6 | 417 | 0.12 [0.00; 0.24] | 1.97 | 0.05 |  |  |  |  |  |  |  |  |  |  |
| Brain lesion volume | 45 | 3686 | 0.29 [0.24; 0.34] | 11.65 | <0.01 | 11 | 906 | 0.39 [0.33; 0.44] | 12.05 | <0.01 | 5 | 139 | 0.08 [-0.09; 0.25] | 0.94 | 0.35 |
| Cortical lesion count | 12 | 1016 | 0.37 [0.24; 0.48] | 5.38 | <0.01 |  |  |  |  |  |  |  |  |  |  |
| Cortical lesion volume | 11 | 1091 | 0.45 [0.36; 0.53] | 8.82 | <0.01 | 3 | 286 | 0.43 [0.28; 0.56] | 5.24 | <0.01 |  |  |  |  |  |
| FLAIR lesion volume | 8 | 456 | 0.28 [0.19; 0.36] | 5.99 | <0.01 |  |  |  |  |  |  |  |  |  |  |
| Normalized brain lesion volume | 8 | 683 | 0.24 [0.02; 0.43] | 2.17 | 0.03 |  |  |  |  |  |  |  |  |  |  |
| T1 lesion count | 7 | 467 | 0.39 [0.27; 0.49] | 6.3 | <0.01 |  |  |  |  |  |  |  |  |  |  |
| T1 lesion volume | 50 | 4536 | 0.35 [0.31; 0.40] | 13.91 | <0.01 | 15 | 2147 | 0.35 [0.27; 0.43] | 7.76 | <0.01 | 4 | 177 | 0.10 [-0.24; 0.42] | 0.59 | 0.56 |
| T1LV/T2LV | 10 | 647 | 0.19 [0.09; 0.28] | 3.75 | <0.01 |  |  |  |  |  |  |  |  |  |  |
| T2 lesion count | 6 | 1022 | 0.26 [0.14; 0.37] | 4.09 | <0.01 |  |  |  |  |  |  |  |  |  |  |
| T2 lesion volume | 87 | 9966 | 0.28 [0.25; 0.31] | 16.9 | <0.01 | 28 | 3239 | 0.30 [0.25; 0.35] | 11.06 | <0.01 | 6 | 254 | 0.17 [-0.03; 0.36] | 1.65 | 0.1 |
| WM lesion volume | 15 | 1456 | 0.43 [0.34; 0.51] | 8.65 | <0.01 | 6 | 516 | 0.37 [0.19; 0.53] | 3.82 | <0.01 |  |  |  |  |  |
| **Volume of spinal cord structure** |  |  |  |  |  |  |  |  |  |  |  |  |  |  |  |
| Cervical cord volume | 5 | 167 | -0.51 [-0.62; -0.38] | -6.94 | <0.01 |  |  |  |  |  |  |  |  |  |  |
| Spinal cord cross-sectional area | 7 | 478 | -0.46 [-0.58; -0.33] | -6 | <0.01 |  |  |  |  |  |  |  |  |  |  |
| Spinal cord volume | 6 | 378 | -0.2 [-0.32; -0.08] | -3.16 | <0.01 |  |  |  |  |  |  |  |  |  |  |
| Upper cervical cord area at C2/C3 | 5 | 668 | -0.31 [-0.42; -0.18] | -4/72 | <0.01 |  |  |  |  |  |  |  |  |  |  |
| Upper cervical cord area | 9 | 1094 | -0.37 [0.42; -0.32] | -12.65 | <0.01 |  |  |  |  |  |  |  |  |  |  |
| **MTR** |  |  |  |  |  |  |  |  |  |  |  |  |  |  |  |
| MTR histogram peak height | 7 | 390 | -0.29 [-0.52; -0.02] | -2.07 | 0.04 |  |  |  |  |  |  |  |  |  |  |
| NAWM MTR | 12 | 790 | -0.27 [-0.36; -0.17] | -5.11 | <0.01 |  |  |  |  |  |  |  |  |  |  |

BPF: Brain Parenchymal Fraction, BPV: Brain Parenchymal Volume, EDSS: Expanded Disability Status Scale, FLAIR: Fluid-attenuated Inversion Recovery, GMF: Grey Matter Fraction, GMV: Grey Matter Volume, MTR: Magnetic Transfer Ratio, NAWM: Normal-appearing White Matter, PwMS: People with Multiple Sclerosis, T1LV: T1 Lesion Volume, T2LV: T2 Lesion Volume, WM: White Matter, WMF: White Matter Fraction, WMV: White Matter Volume.

Table S2. Results of meta-analyses of T25FW and MRI measurements in pwMS.

| **T25FW** | | | | | | | | | | | | | | | |
| --- | --- | --- | --- | --- | --- | --- | --- | --- | --- | --- | --- | --- | --- | --- | --- |
|  | **Overall** | | | | | **RRMS** | | | | | **PMS** | | | | |
|  | Studies | Patients | Pooled correlation coefficient | z-score | *p*-value | Studies | Patients | Pooled correlation coefficient | z-score | *p*-value | Studies | Patients | Pooled correlation coefficient | z-score | *p*-value |
| **Volume of brain structure** |  |  |  |  |  |  |  |  |  |  |  |  |  |  |  |
| BPF | 15 | 1006 | -0.15 [-0.26; -0.03] | -2.52 | 0.01 |  |  |  |  |  |  |  |  |  |  |
| GMF | 6 | 324 | -0.32 [-0.43; -0.20] | -4.97 | <0.01 |  |  |  |  |  |  |  |  |  |  |
| GMV | 4 | 210 | -0.21 [-0.49; 0.10] | -1.34 | 0.18 |  |  |  |  |  |  |  |  |  |  |
| Normalized BPV | 3 | 107 | -0.33 [-0.63; 0.05] | -1.7 | 0.09 |  |  |  |  |  |  |  |  |  |  |
| Normalized brain volume | 8 | 989 | -0.20 [-0.29; -0.10] | -3.92 | <0.01 |  |  |  |  |  |  |  |  |  |  |
| Normalized GMV | 11 | 825 | -0.23 [-0.35; -0.09] | -3.19 | <0.01 | 3 | 279 | -0.22 [-0.38; -0.05] | -2.49 | 0.01 |  |  |  |  |  |
| Normalized thalamus volume | 5 | 435 | -0.10 [-0.29; 0.08] | -1.1 | 0.27 |  |  |  |  |  |  |  |  |  |  |
| Normalized WMV | 11 | 789 | -0.05 [-0.23; 0.13] | -0.52 | 0.6 |  |  |  |  |  |  |  |  |  |  |
| Thalamus volume | 3 | 239 | -0.37 [-0.47; -0.25] | -5.86 | <0.01 |  |  |  |  |  |  |  |  |  |  |
| WMF | 6 | 324 | -0.12 [-0.28; 0.05] | -1.41 | 0.16 |  |  |  |  |  |  |  |  |  |  |
| WMV | 3 | 152 | -0.03 [-0.55; 0.50] | -0.11 | 0.91 |  |  |  |  |  |  |  |  |  |  |
| **Lesion of brain structure** |  |  |  |  |  |  |  |  |  |  |  |  |  |  |  |
| Brain lesion volume | 7 | 642 | 0.27 [0.11; 0.42] | 3.21 | <0.01 |  |  |  |  |  |  |  |  |  |  |
| FLAIR lesion volume | 3 | 120 | 0.14 [-0.16; 0.42] | 0.92 | 0.36 |  |  |  |  |  |  |  |  |  |  |
| T1 lesion volume | 12 | 584 | 0.26 [0.13; 0.38] | 3.87 | <0.01 | 3 | 197 | 0.31 [0.18; 0.43] | 4.41 | <0.01 |  |  |  |  |  |
| T2 lesion volume | 18 | 1678 | 0.16 [0.04; 0.28] | 2.59 | <0.01 | 6 | 322 | 0.26 [0.15; 0.36] | 4.72 | <0.01 | 3 | 180 | 0.03 [-0.36; 0.41] | 0.13 | 0.9 |
| WM lesion volume | 3 | 166 | 0.22 [-0.09; 0.49] | 1.4 | 0.16 |  |  |  |  |  |  |  |  |  |  |
| **Volume of spinal cord structure** |  |  |  |  |  |  |  |  |  |  |  |  |  |  |  |
| Upper cervical cord area at C2/C3 | 3 | 242 | -0.33 [-0.45; -0.20] | -4/74 | <0.01 |  |  |  |  |  |  |  |  |  |  |
| Upper cervical cord area | 4 | 914 | -0.12 [-0.35; 0.13] | -0.93 | 0.35 |  |  |  |  |  |  |  |  |  |  |
| **MTR** |  |  |  |  |  |  |  |  |  |  |  |  |  |  |  |
| NAWM MTR | 5 | 454 | -0.11 [-0.40; 0.20] | -0.69 | 0.49 |  |  |  |  |  |  |  |  |  |  |

BPF: Brain Parenchymal Fraction, BPV: Brain Parenchymal Volume, FLAIR: Fluid-attenuated Inversion Recovery, GMF: Grey Matter Fraction, GMV: Grey Matter Volume, MTR: Magnetic Transfer Ratio, NAWM: Normal-appearing White Matter, PwMS: People with Multiple Sclerosis, T25FW: Timed 25-Foot Walk, WM: White Matter, WMF: White Matter Fraction, WMV: White Matter Volume.

Table S3. Results of meta-analyses of 9HPT and MRI measurements in pwMS.

| **9HPT** | | | | | | | | | | | | | | | |
| --- | --- | --- | --- | --- | --- | --- | --- | --- | --- | --- | --- | --- | --- | --- | --- |
|  | **Overall** | | | | | **RRMS** | | | | | **PMS** | | | | |
|  | Studies | Patients | Pooled correlation coefficient | z-score | *p*-value | Studies | Patients | Pooled correlation coefficient | z-score | *p*-value | Studies | Patients | Pooled correlation coefficient | z-score | *p*-value |
| **Volume of brain structure** |  |  |  |  |  |  |  |  |  |  |  |  |  |  |  |
| BPF | 11 | 1143 | -0.05 [-0.33; 0.24] | -0.35 | 0.73 |  |  |  |  |  |  |  |  |  |  |
| GMF | 4 | 619 | 0.07 [-0.31; 0.43] | 0.35 | 0.73 |  |  |  |  |  |  |  |  |  |  |
| Normalized brain volume | 8 | 832 | -0.32 [-0.45; -0.18] | -4.45 | <0.01 |  |  |  |  |  |  |  |  |  |  |
| Normalized WMV | 8 | 589 | -0.16 [-0.37; 0.07] | -1.38 | 0.17 |  |  |  |  |  |  |  |  |  |  |
| Putamen volume | 3 | 220 | -0.31 [-0.42; -0.18] | -4.59 | <0.01 |  |  |  |  |  |  |  |  |  |  |
| Thalamus volume | 4 | 328 | -0.4 [-0.49; -0.30] | -7.18 | <0.01 |  |  |  |  |  |  |  |  |  |  |
| WMF | 5 | 689 | 0.17 [-0.27; 0.55] | 0.75 | 0.45 |  |  |  |  |  |  |  |  |  |  |
| **Lesion of brain structure** |  |  |  |  |  |  |  |  |  |  |  |  |  |  |  |
| Brain lesion volume | 6 | 671 | 0.29 [0.19; 0.39] | 5.49 | <0.01 |  |  |  |  |  |  |  |  |  |  |
| T1 lesion volume | 6 | 843 | 0.24 [-0.01; 0.47] | 1.86 | 0.06 | 3 | 187 | 0.05 [-0.58; 0.64] | 0.14 | 0.89 |  |  |  |  |  |
| T2 lesion volume | 15 | 1882 | 0.30 [0.19; 0.40] | 5.1 | <0.01 | 4 | 254 | 0.37 [0.26; 0.47] | 6.05 | <0.01 |  |  |  |  |  |
| WM lesion volume | 3 | 150 | 0.46 [0.25; 0.63] | 4 | <0.01 |  |  |  |  |  |  |  |  |  |  |
| **Volume of spinal cord structure** |  |  |  |  |  |  |  |  |  |  |  |  |  |  |  |
| Upper cervical cord area at C2/C3 | 3 | 242 | -0.27 [-0.39; -0.15] | -4.29 | <0.01 |  |  |  |  |  |  |  |  |  |  |
| Upper cervical cord area | 4 | 914 | -0.09 [-0.35; 0.19] | -0.64 | 0.52 |  |  |  |  |  |  |  |  |  |  |
| **MTR** |  |  |  |  |  |  |  |  |  |  |  |  |  |  |  |
| NAWM MTR | 4 | 320 | 0.07 [-0.35; 0.46] | 0.3 | 0.76 |  |  |  |  |  |  |  |  |  |  |

BPF: Brain Parenchymal Fraction, BPV: Brain Parenchymal Volume, GMF: Grey Matter Fraction, 9HPT: 9-Hole Peg Test, MTR: Magnetic Transfer Ratio, NAWM: Normal-appearing White Matter, PwMS: People with Multiple Sclerosis, WMF: White Matter Fraction, WMV: White Matter Volume.

Table S4. Results of meta-analyses of MSFC and MRI measurements in pwMS.

|  | **MSFC** | | | | | | | | | | | | | | |
| --- | --- | --- | --- | --- | --- | --- | --- | --- | --- | --- | --- | --- | --- | --- | --- |
|  | **Overall** | | | | | **RRMS** | | | | | **PMS** | | | | |
|  | Studies | Patients | Pooled correlation coefficient | z-score | *p*-value | Studies | Patients | Pooled correlation coefficient | z-score | *p*-value | Studies | Patients | Pooled correlation coefficient | z-score | *p*-value |
| **Volume of brain structure** |  |  |  |  |  |  |  |  |  |  |  |  |  |  |  |
| BPF | 7 | 455 | 0.38 [0.30; 0.46] | 8.33 | <0.01 |  |  |  |  |  |  |  |  |  |  |
| GMF | 3 | 154 | 0.47 [0.33; 0.59] | 6.11 | <0.01 |  |  |  |  |  |  |  |  |  |  |
| Normalized brain volume | 3 | 290 | 0.45 [0.35; 0.54] | 8.05 | <0.01 |  |  |  |  |  |  |  |  |  |  |
| Normalized GMV | 5 | 391 | 0.32 [0.19; 0.45] | 4.51 | <0.01 |  |  |  |  |  |  |  |  |  |  |
| WMV | 5 | 288 | 0.37 [0.24; 0.49] | 5.34 | <0.01 |  |  |  |  |  |  |  |  |  |  |
| **Lesion of brain structure** |  |  |  |  |  |  |  |  |  |  |  |  |  |  |  |
| T1 lesion volume | 8 | 576 | -0.36 [-0.44; -0.27] | -7.96 | <0.01 | 3 | 111 | -0.36 [-0.52; -0.18] | -3/82 | <0.01 |  |  |  |  |  |
| T2 lesion volume | 11 | 797 | -0.33 [-0.39; -0.26] | -9.38 | <0.01 | 4 | 172 | -0.30 [-0.44; -0.16] | -3/95 | <0.01 |  |  |  |  |  |

BPF: Brain Parenchymal Fraction, BPV: Brain Parenchymal Volume, GMF: Grey Matter Fraction, GMV: Grey Matter Volume, MSFC: Multiple Sclerosis Functional Composite, MTR: Magnetic Transfer Ratio, NAWM: Normal-appearing White Matter, PwMS: People with Multiple Sclerosis, WMV: White Matter Volume.
