# Supplementary material for: Neuroimaging markers and disability scales in multiple sclerosis: A systematic review and meta-analysis
Source: PLoS One. 2024 Dec 5;19(12):e0312421. doi: 10.1371/journal.pone.0312421 (PMC11620670; doi:10.1371/journal.pone.0312421)
Supplement: S4 File — (DOCX) [file pone.0312421.s005.docx]

Supplementary 4. Forest plots of disabilitiy and MRI measurements in pwMS.


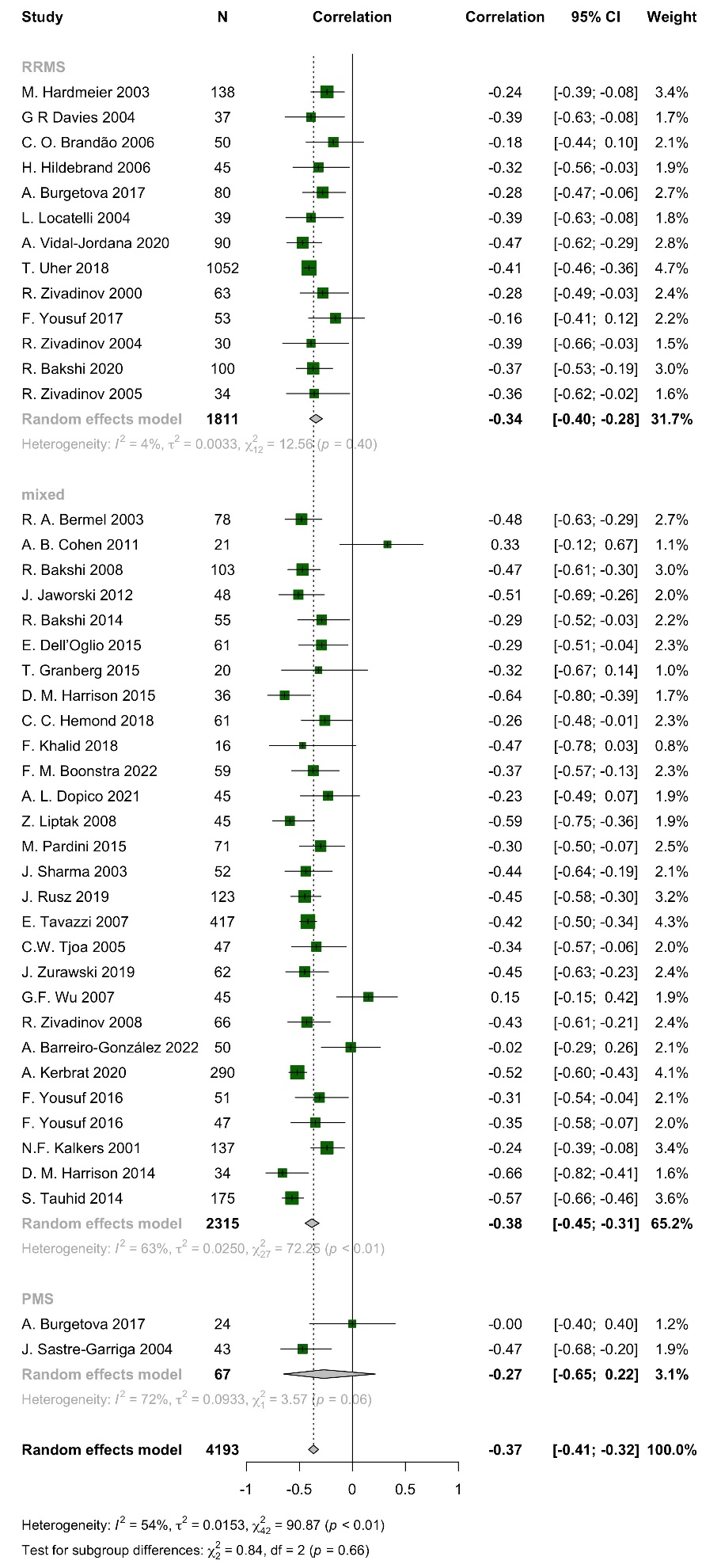


Figure S1. Forest plot of EDSS and BPF correlation in pwMS.


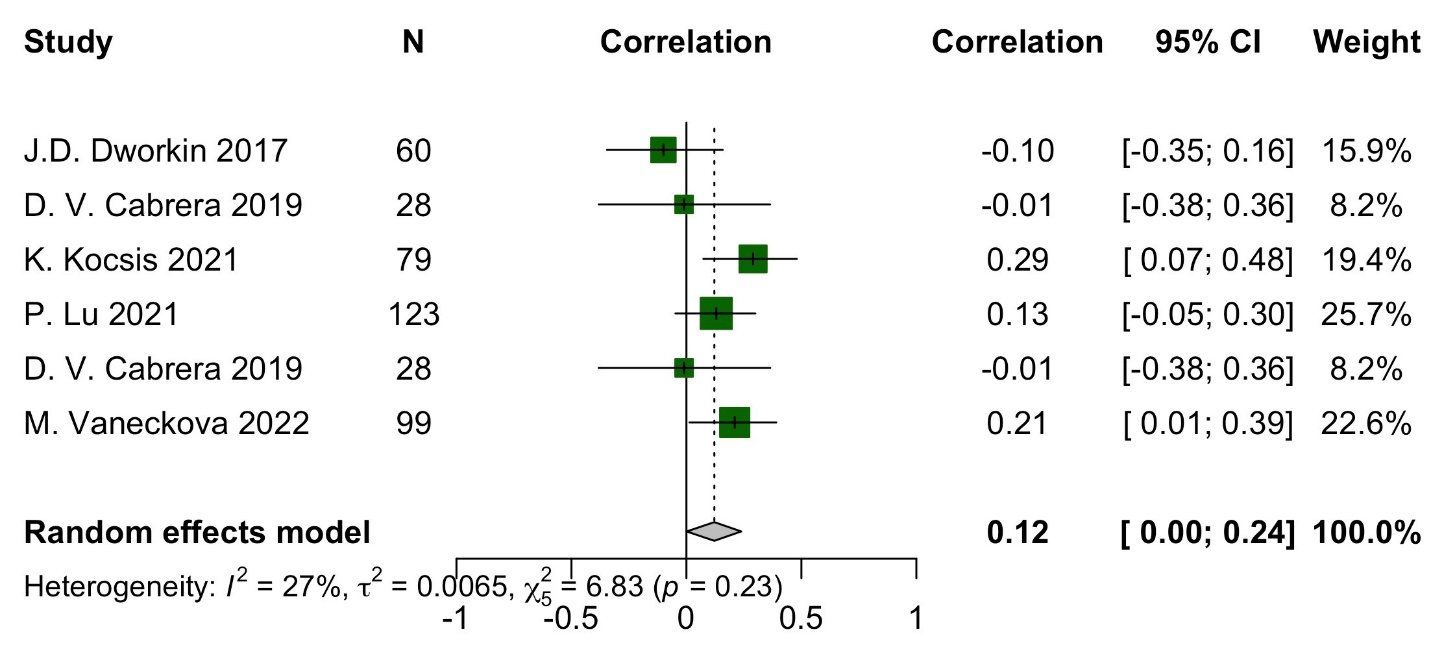


Figure S2. Forest plot of EDSS and brain lesion count correlation in pwMS.


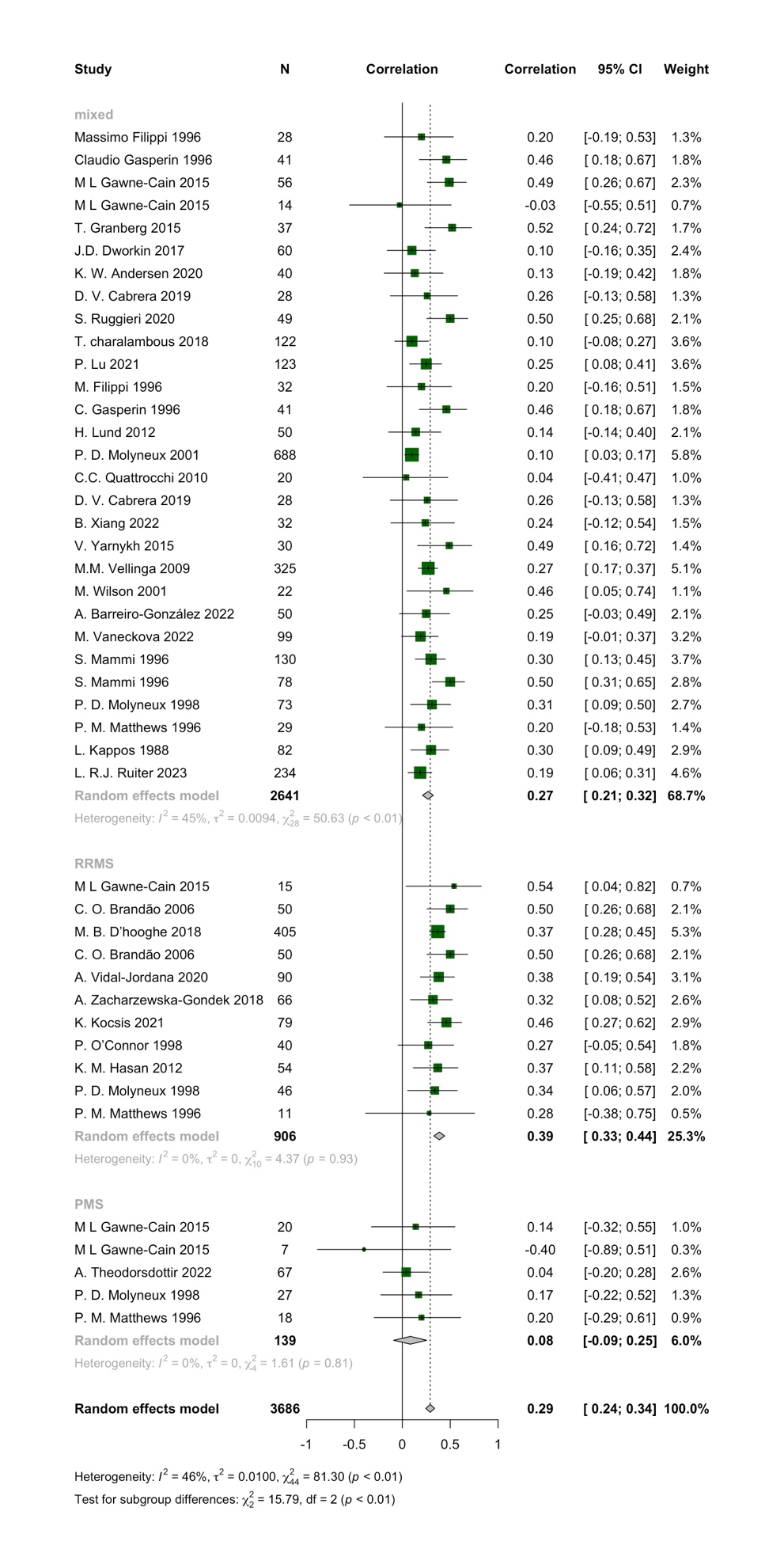


Figure S3. Forest plot of EDSS and brain lesion volume correlation in pwMS.


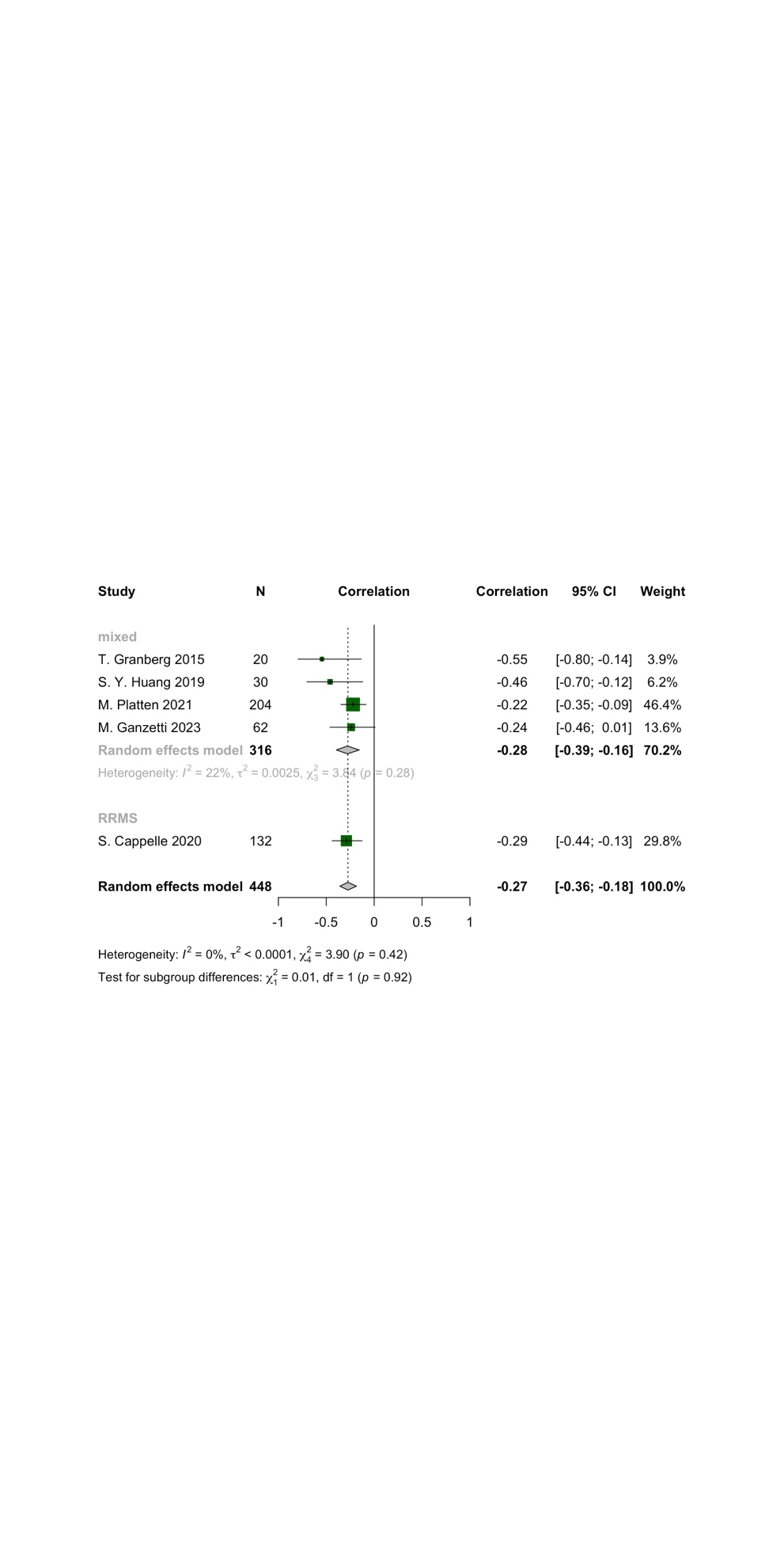


Figure S4. Forest plot of EDSS and corpus callosum area correlation in pwMS.


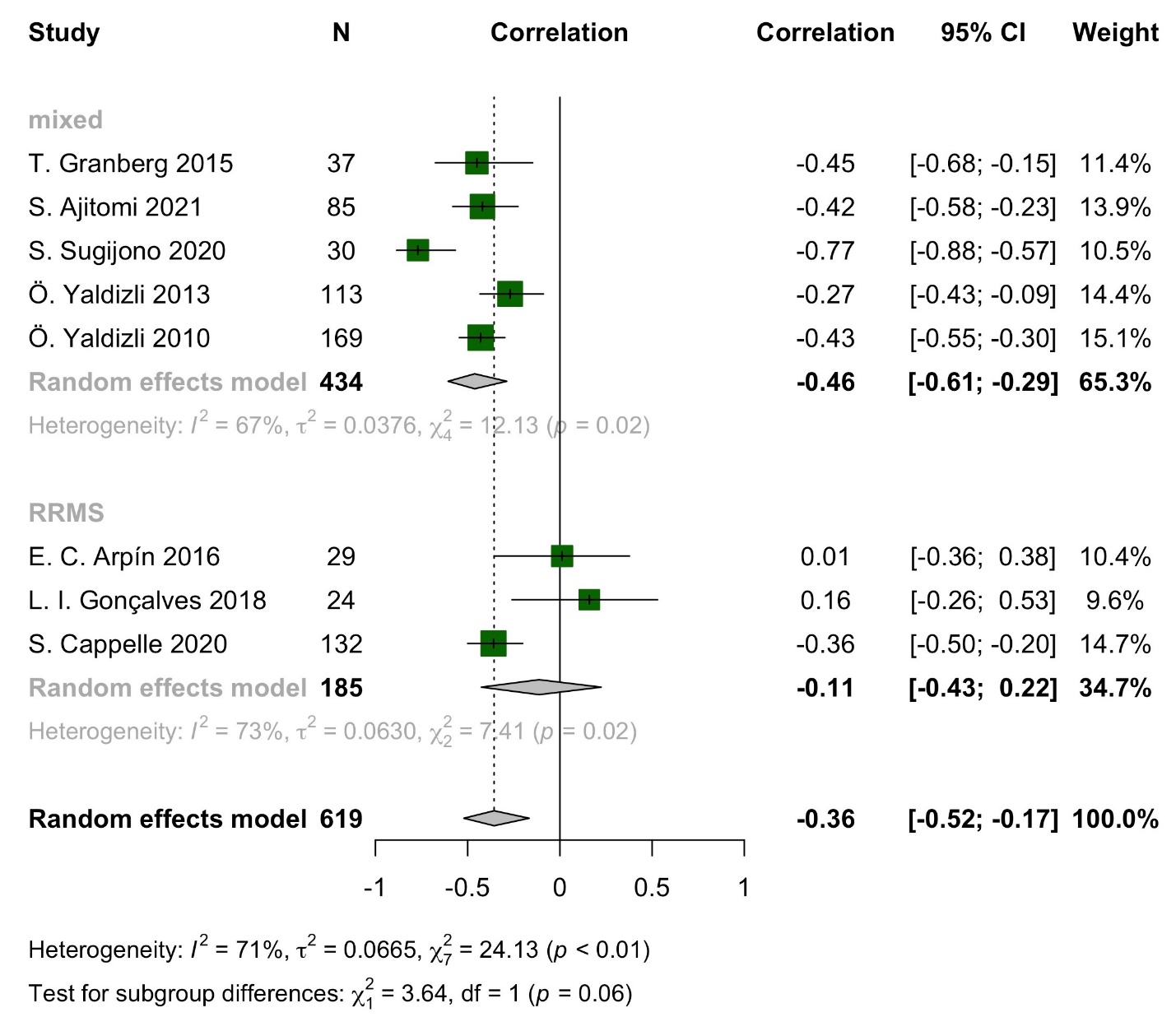


Figure S5. Forest plot of EDSS and corpus callosum index correlation in pwMS.


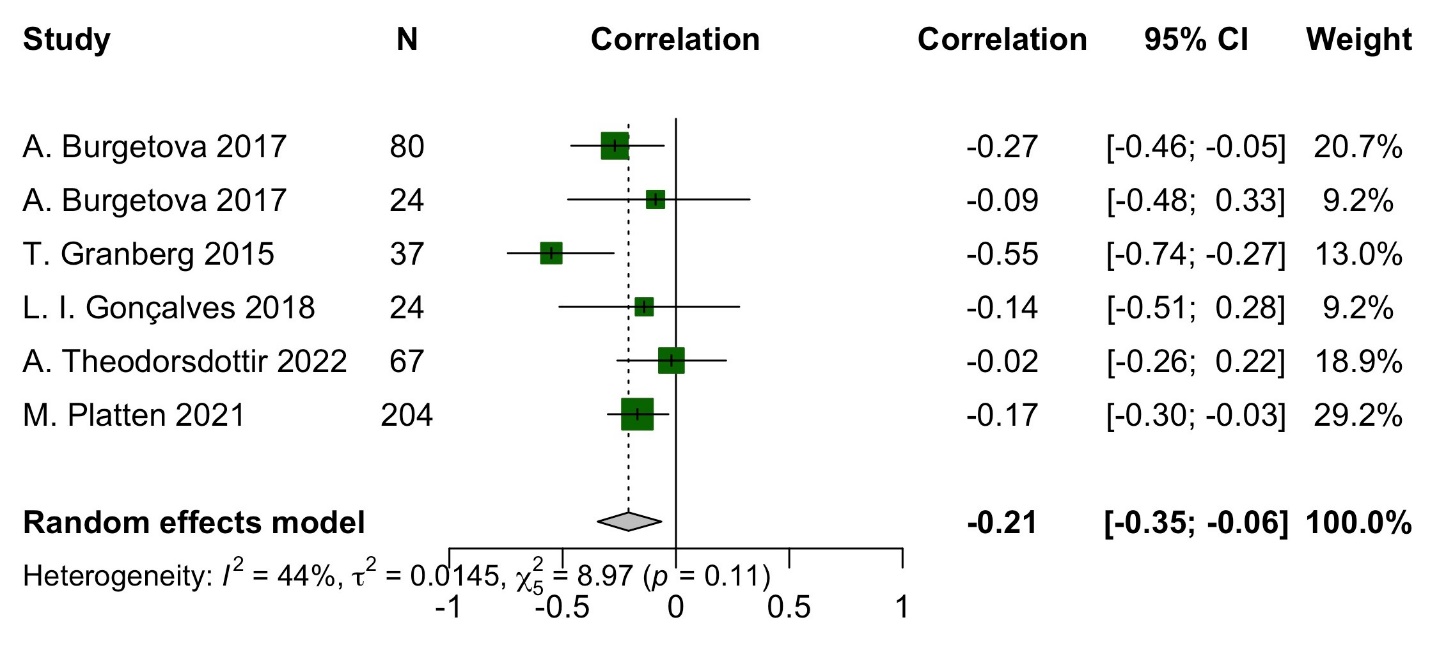


Figure S6. Forest plot of EDSS and corpus callosum volume correlation in pwMS.


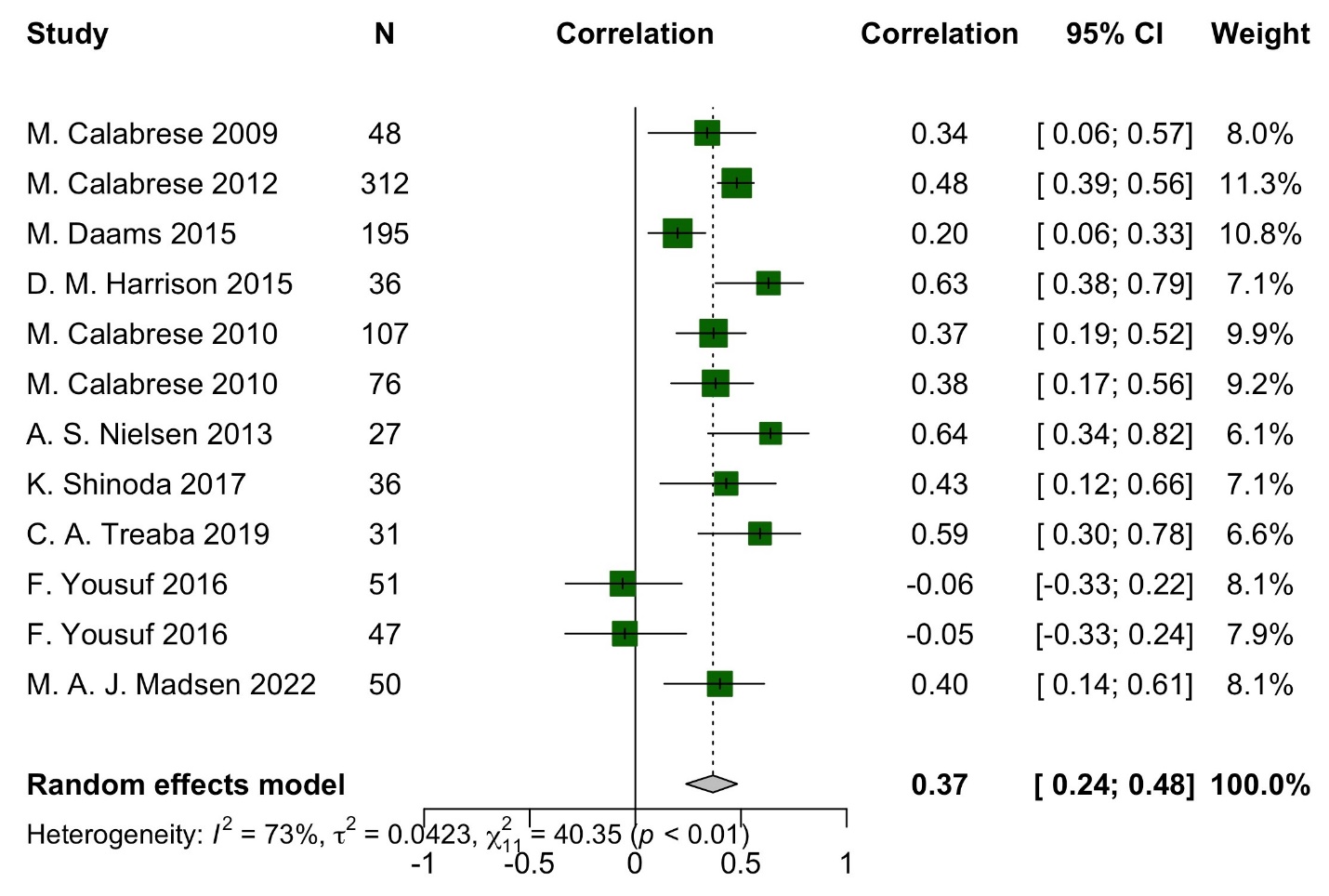


Figure S7. Forest plot of EDSS and cortical lesion count correlation in pwMS.


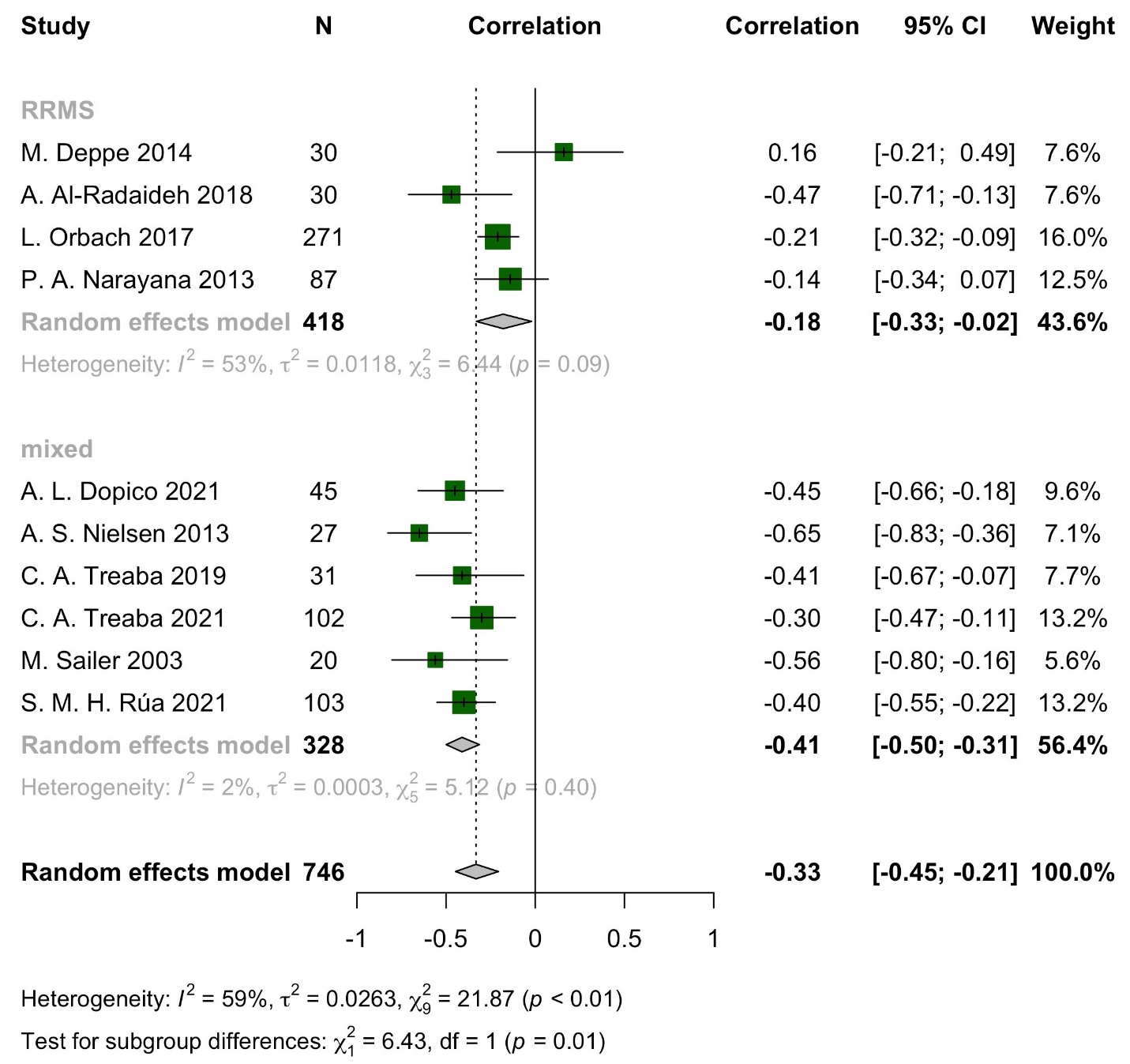


Figure S8. Forest plot of EDSS and cortical thickness correlation in pwMS.


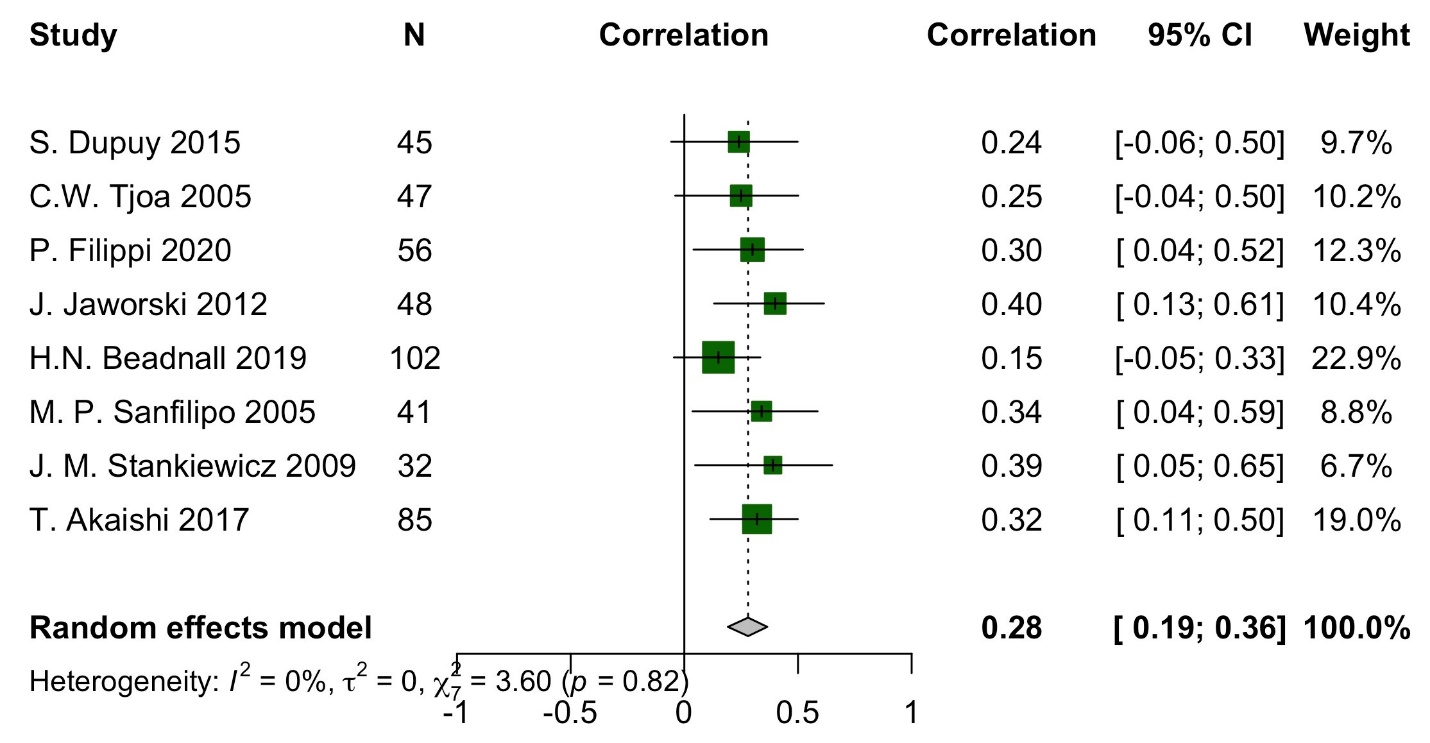


Figure S9. Forest plot of EDSS and FLAIR lesion volume correlation in pwMS.


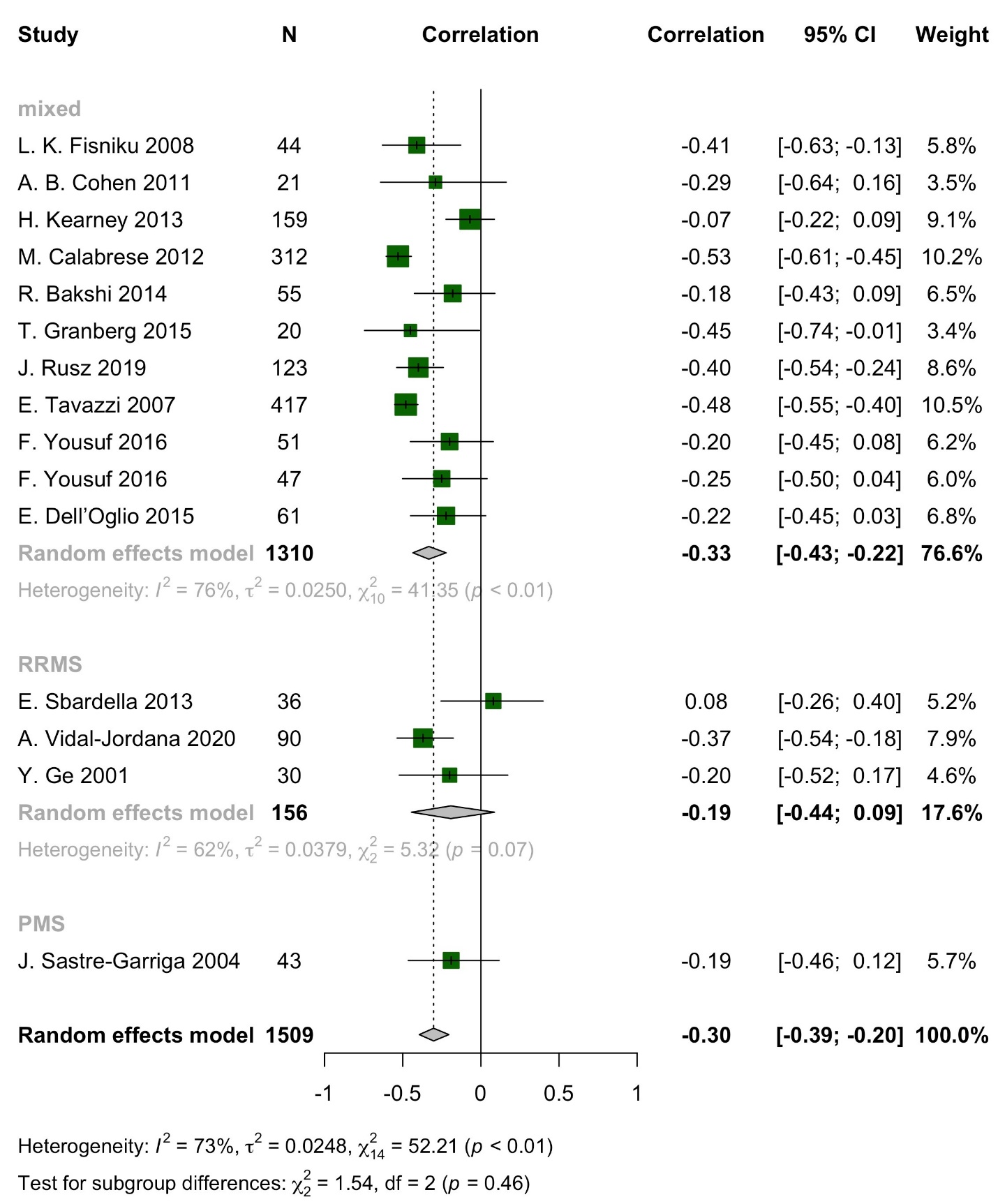


Figure S10. Forest plot of EDSS and grey matter fraction correlation in pwMS.


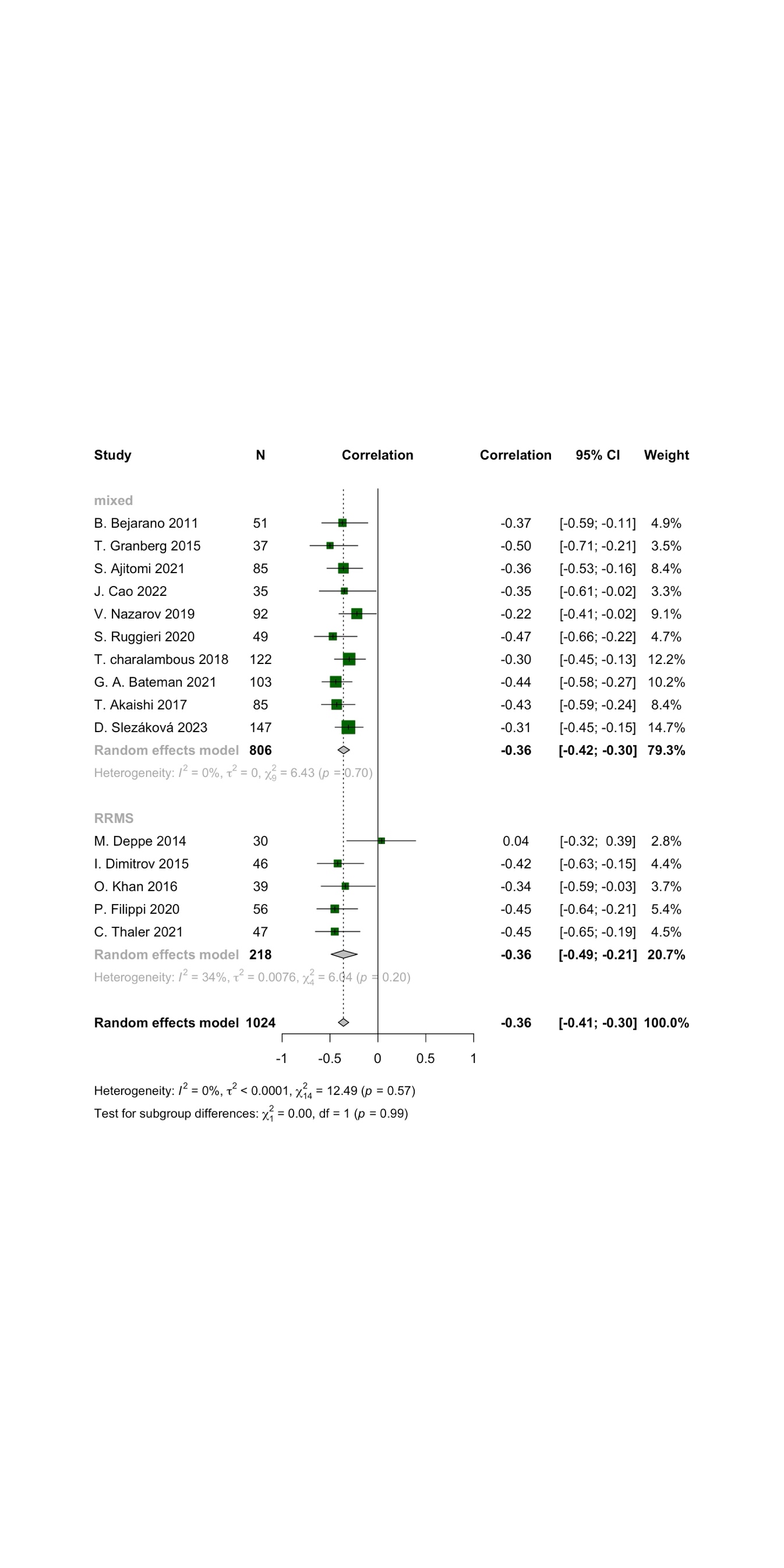


Figure S11. Forest plot of EDSS and grey matter volume correlation in pwMS.


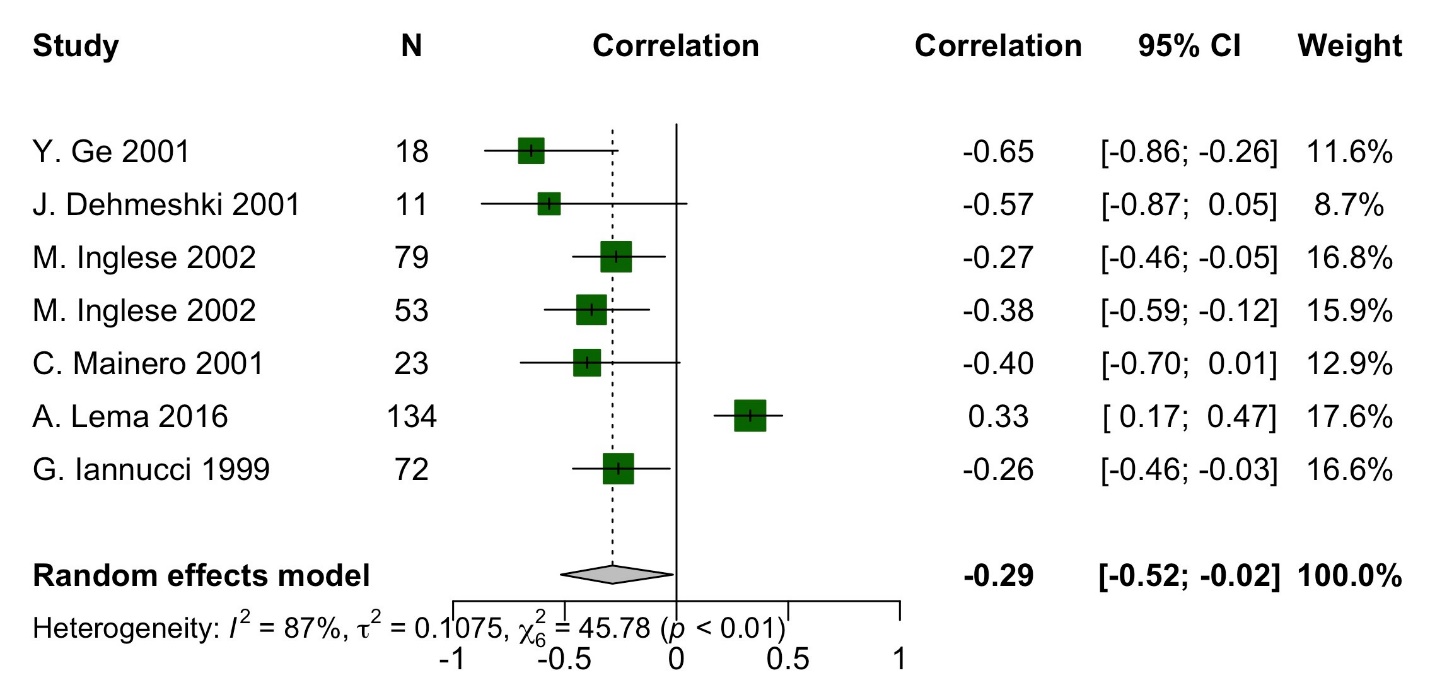


Figure S12. Forest plot of EDSS and MTR histogram peak height correlation in pwMS.


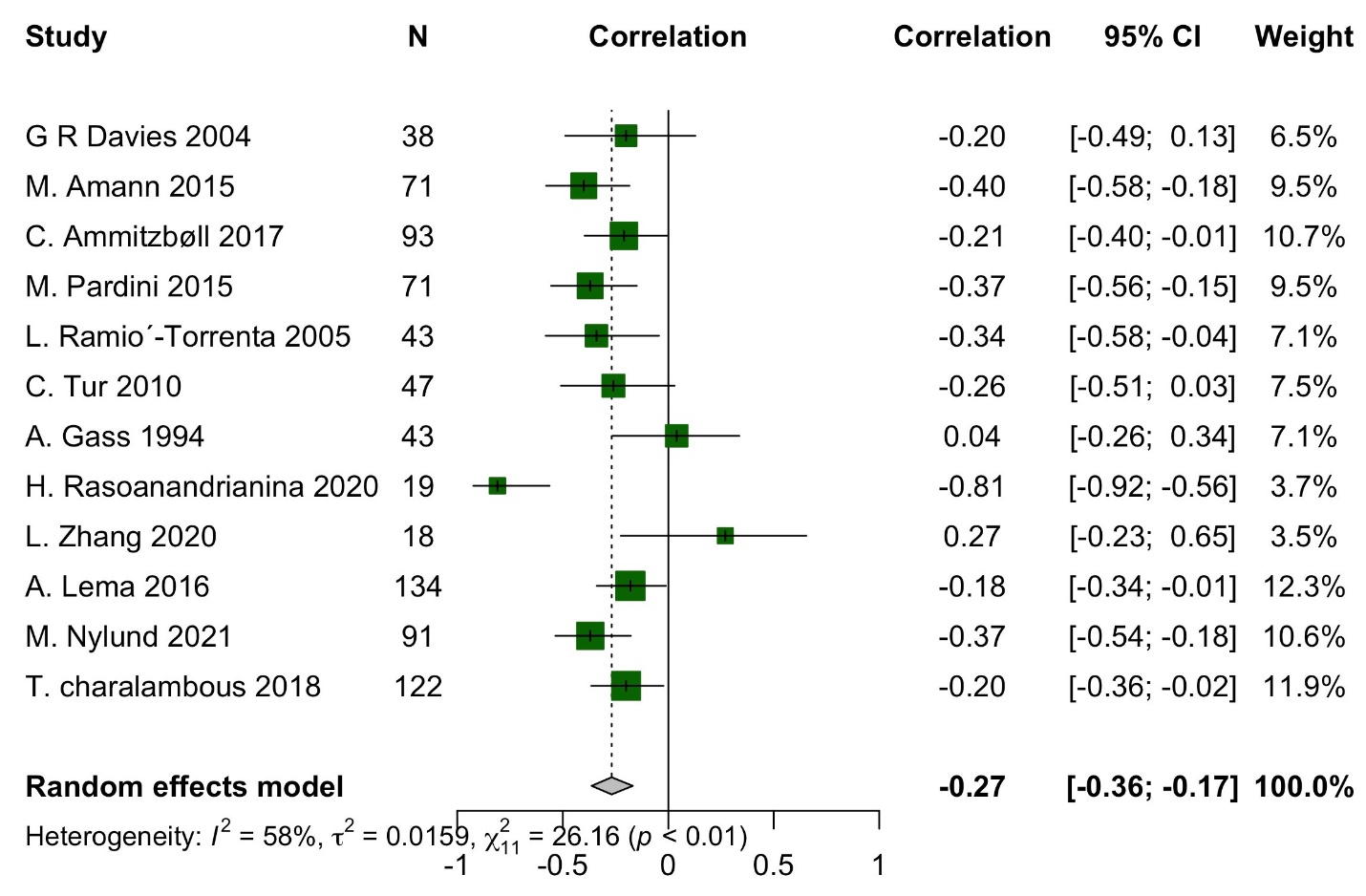


Figure S13. Forest plot of EDSS and normal-appearing white matter MTR correlation in pwMS.


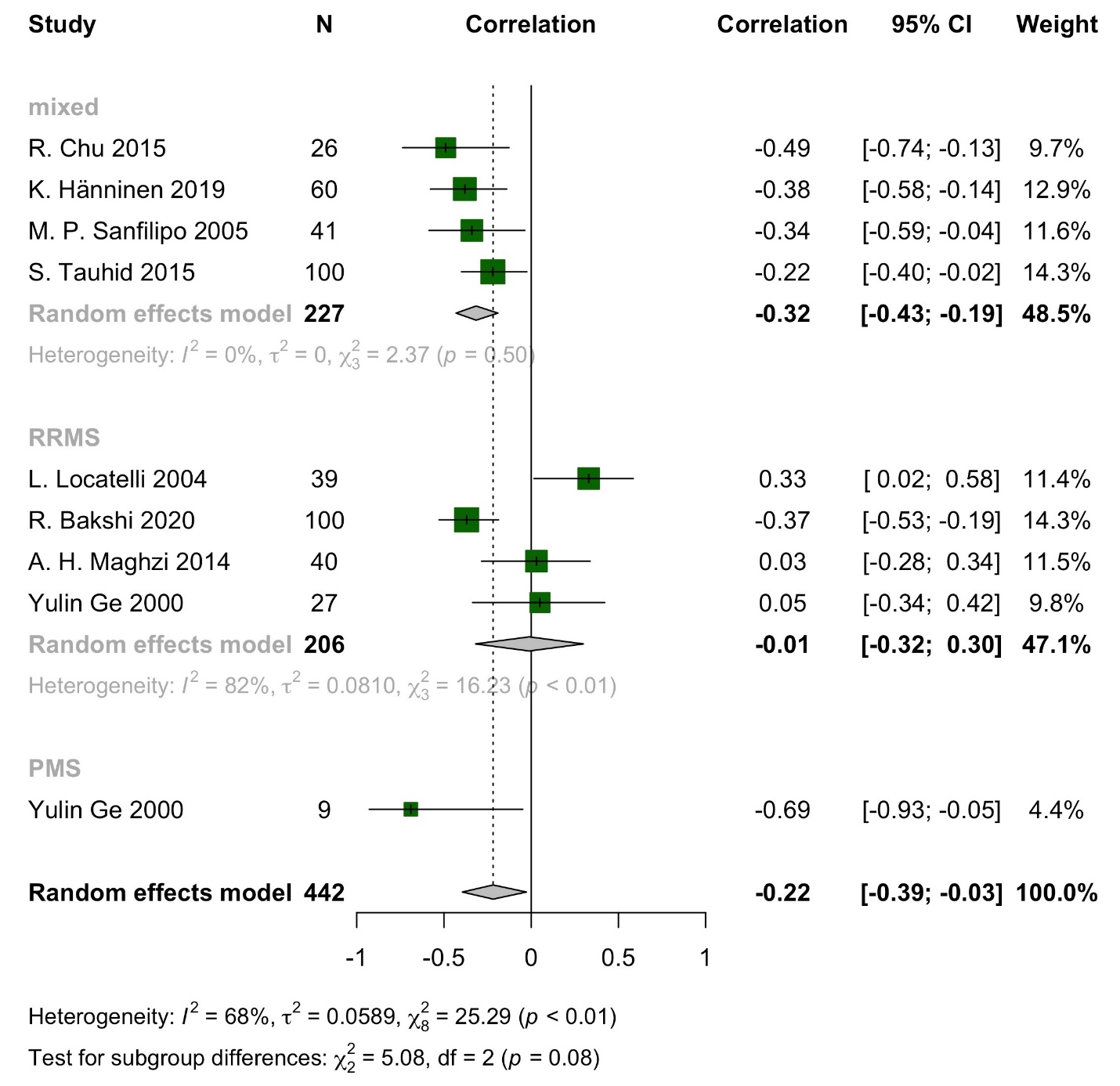


Figure S14. Forest plot of EDSS and normalized brain parenchymal volume correlation in pwMS.


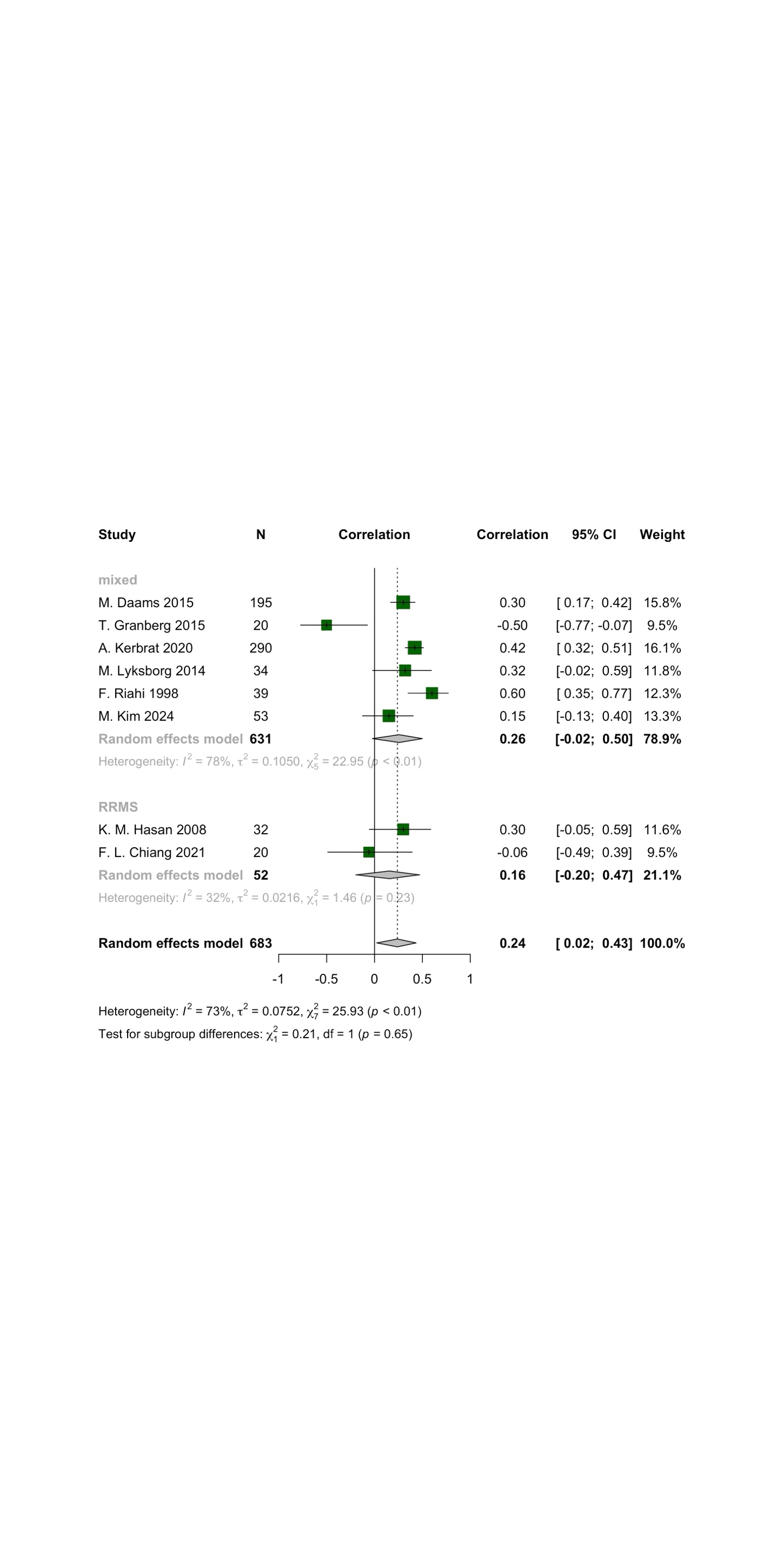


Figure S15. Forest plot of EDSS and normalized brain lesion volume correlation in pwMS.


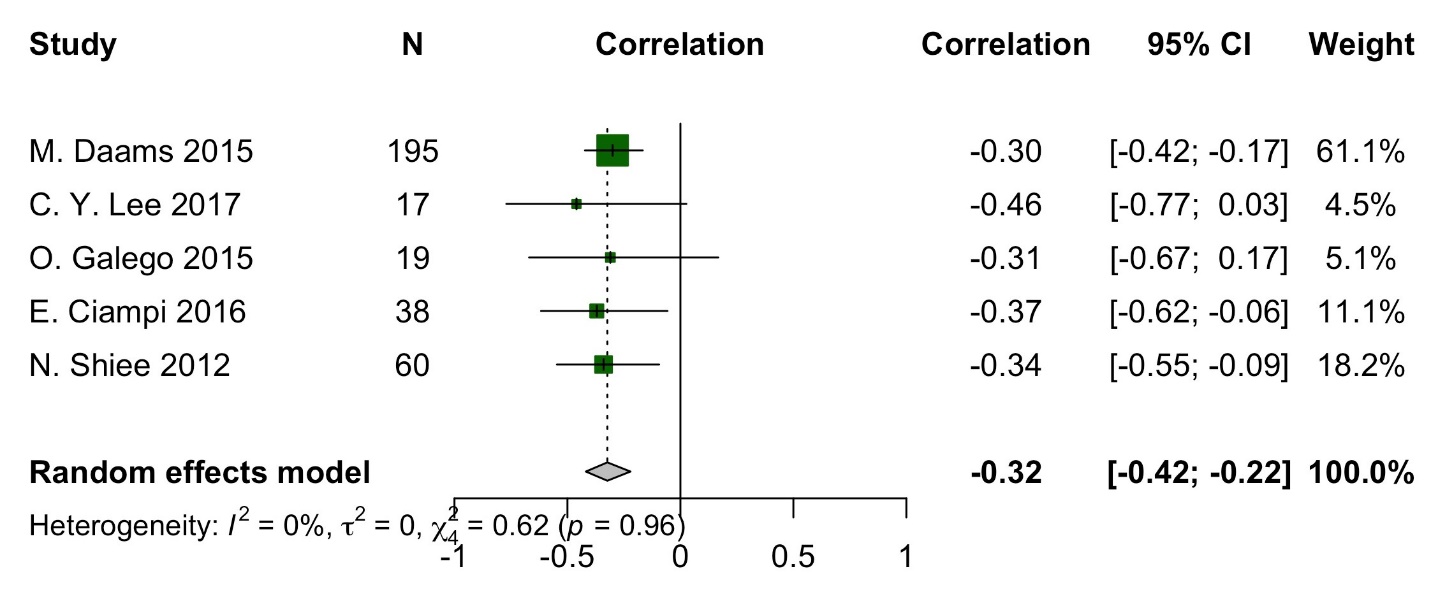


Figure S16. Forest plot of EDSS and normalized brain stem volume correlation in pwMS.


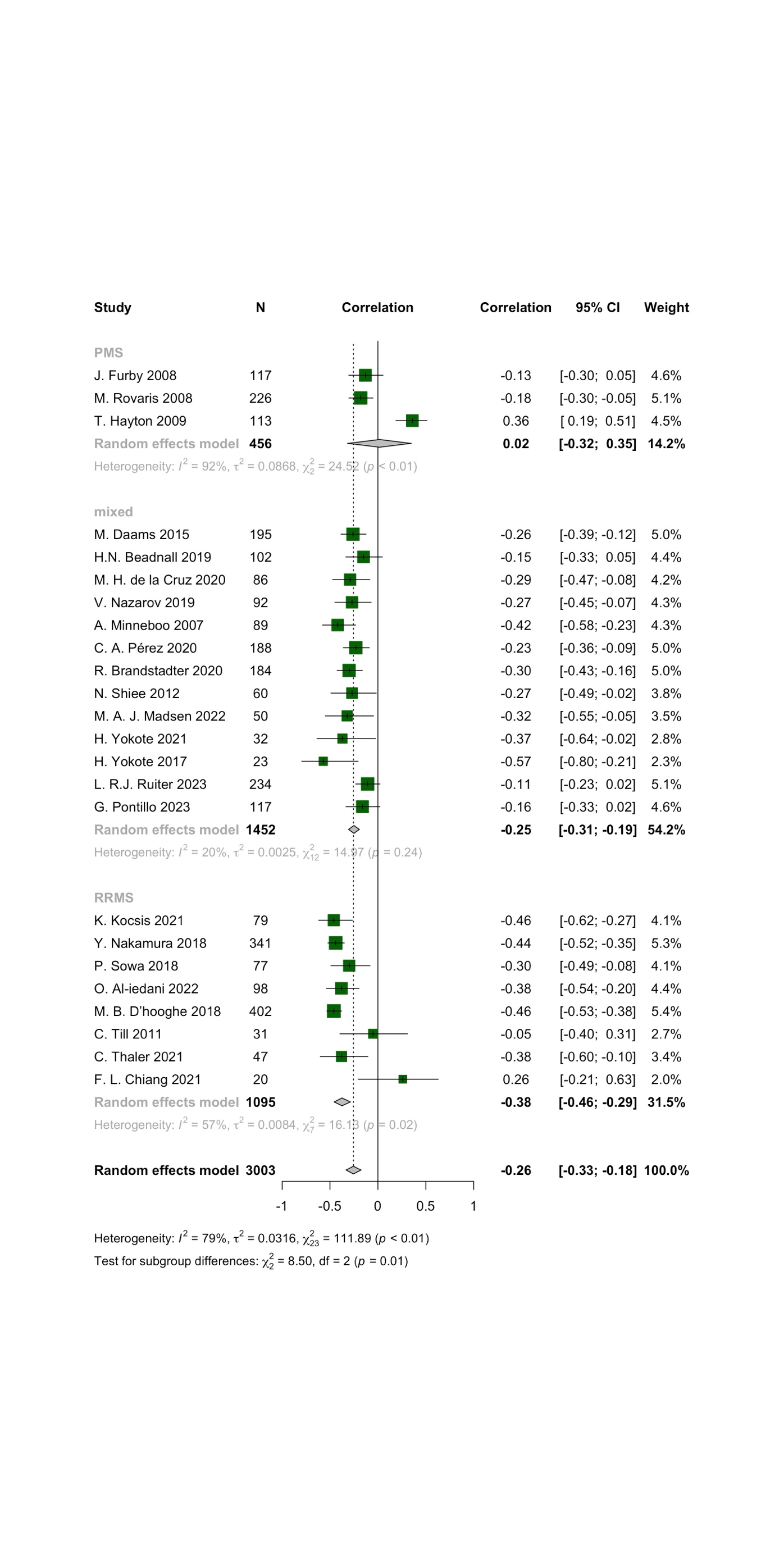


Figure S17. Forest plot of EDSS and normalized brain volume correlation in pwMS.


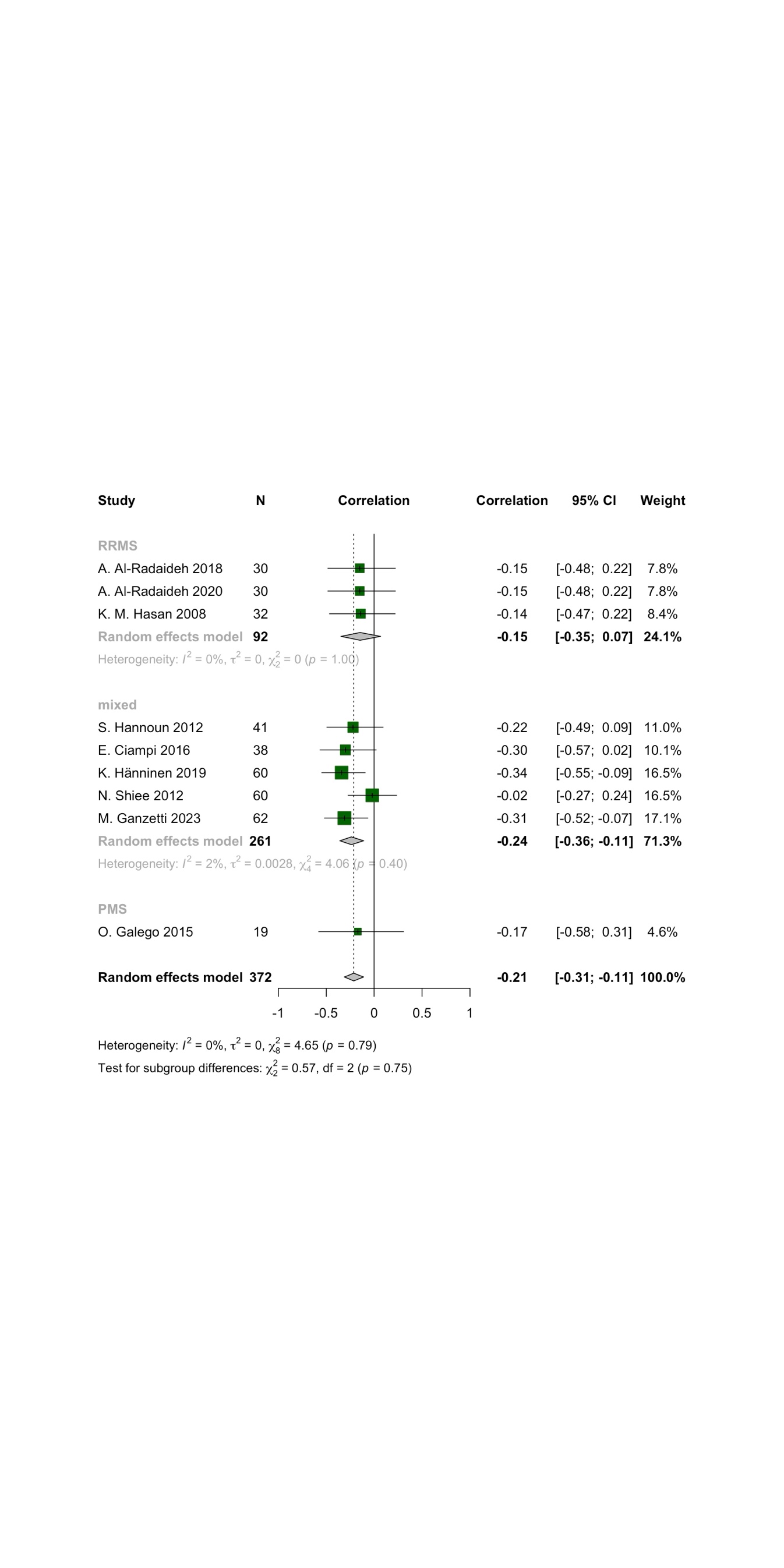


Figure S18. Forest plot of EDSS and normalized caudate volume correlation in pwMS.


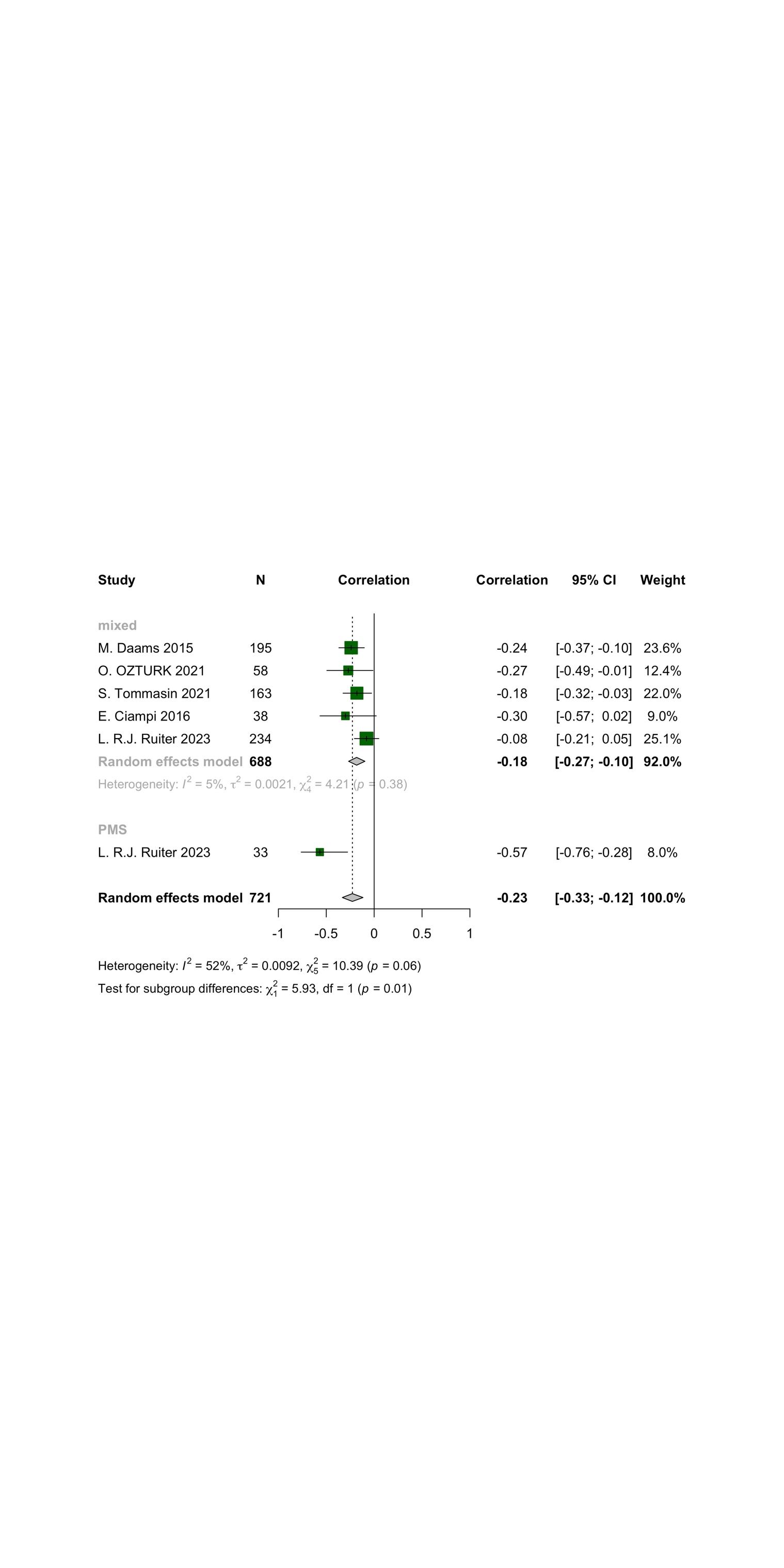


Figure S19. Forest plot of EDSS and normalized cerebellar volume correlation in pwMS.


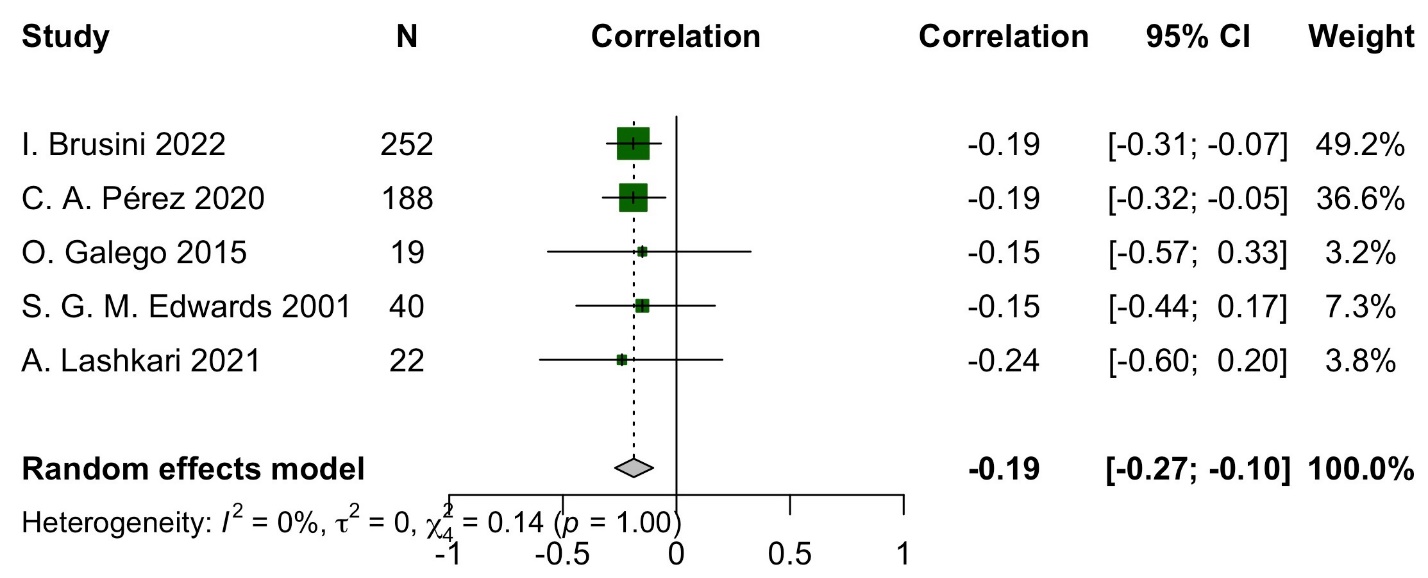


Figure S20. Forest plot of EDSS and normalized corpus callosum volume correlation in pwMS.


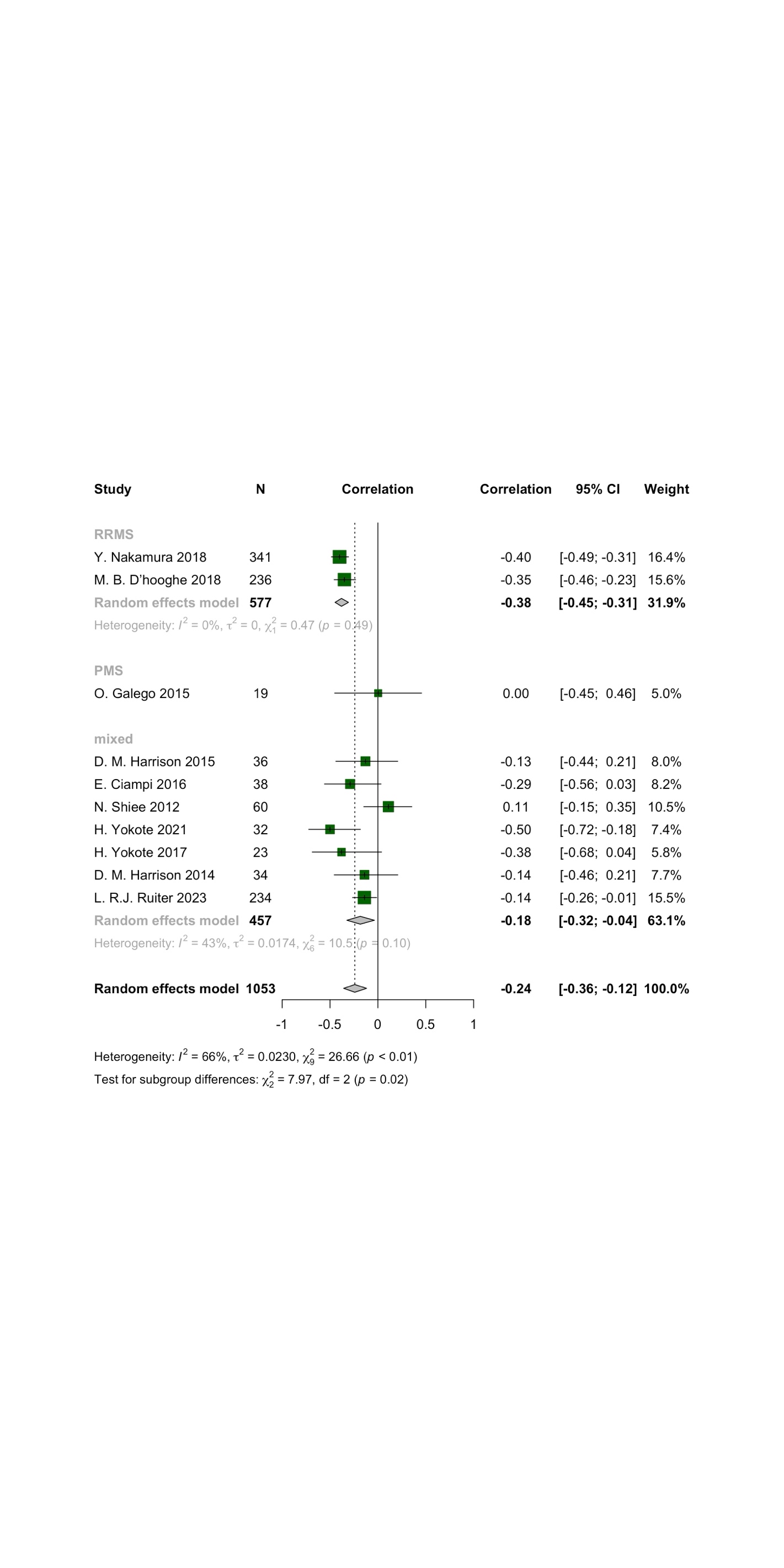


Figure S21. Forest plot of EDSS and normalized cortical grey matter volume correlation in pwMS.


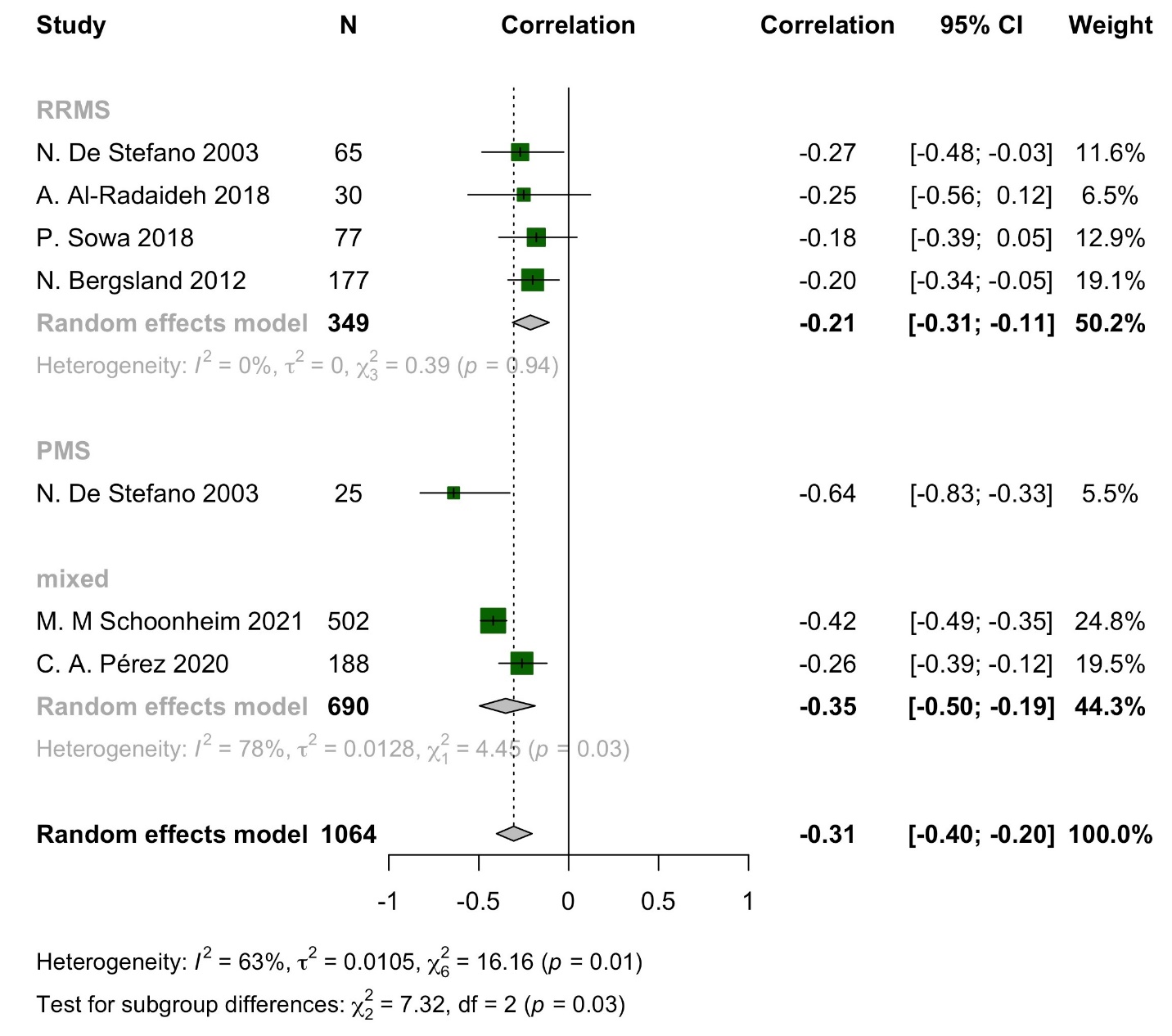


Figure S22. Forest plot of EDSS and normalized cortical volume correlation in pwMS.


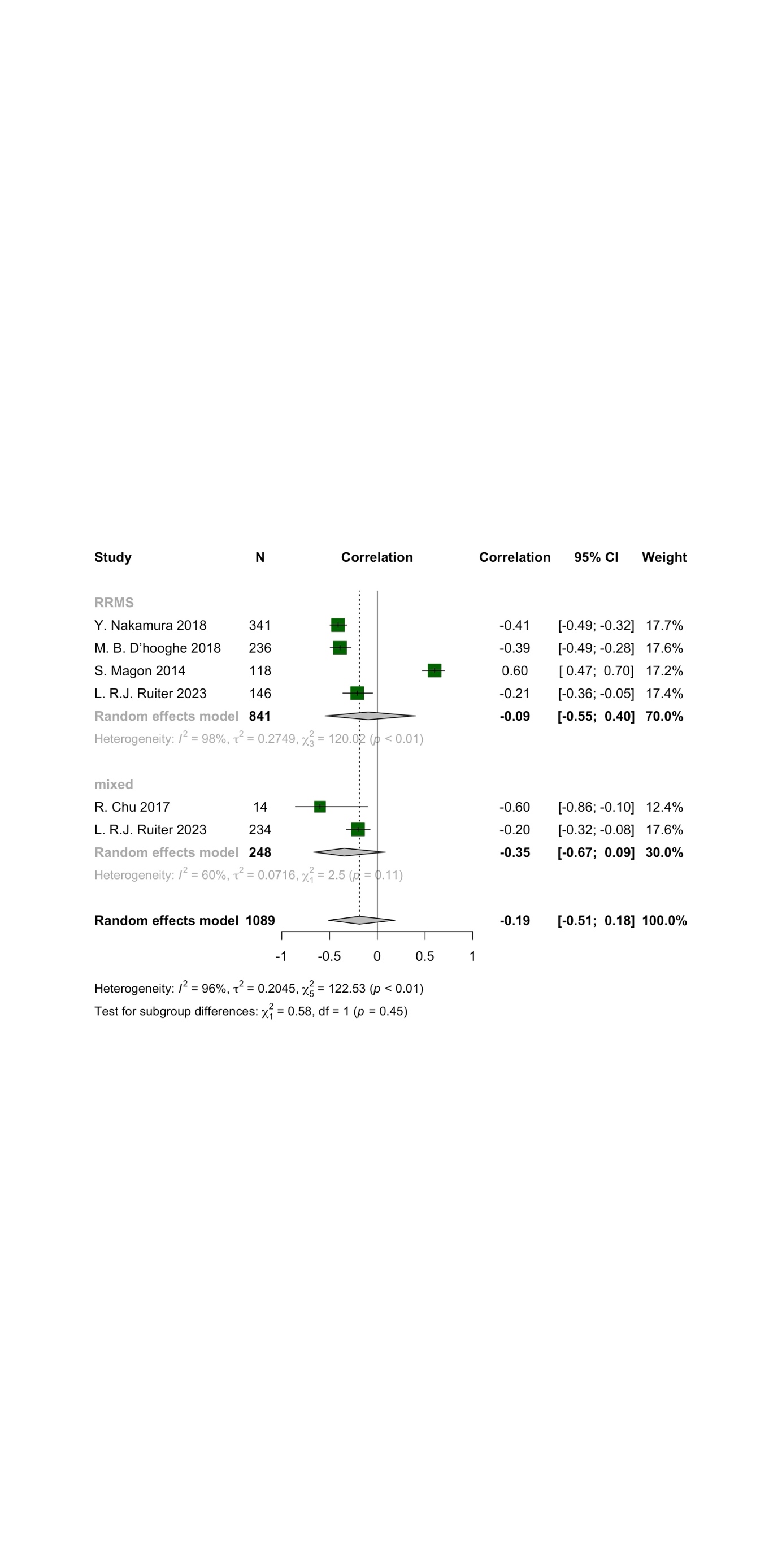


Figure S23. Forest plot of EDSS and normalized deep gray matter volume correlation in pwMS.


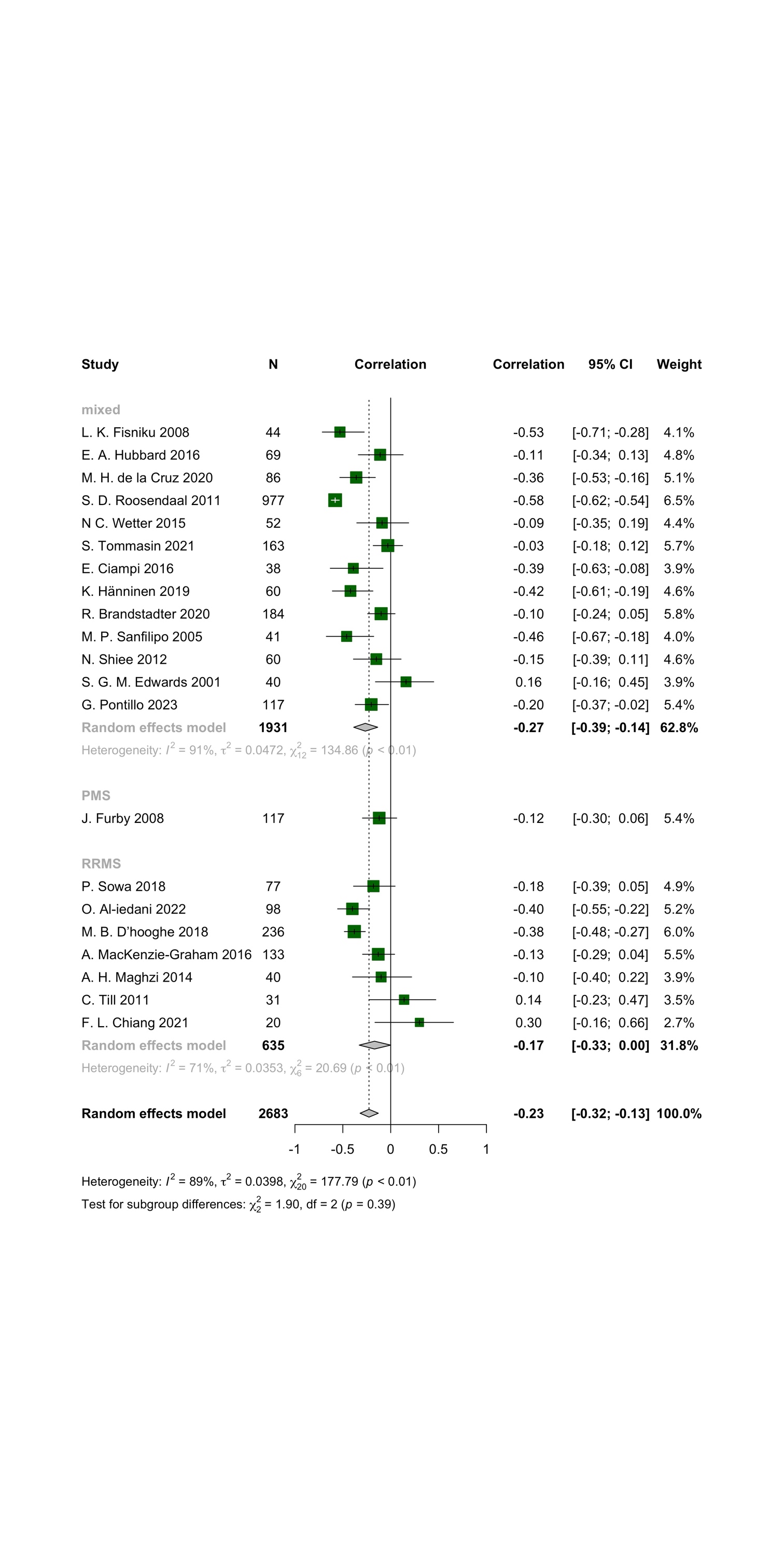


Figure S24. Forest plot of EDSS and normalized grey matter volume correlation in pwMS.


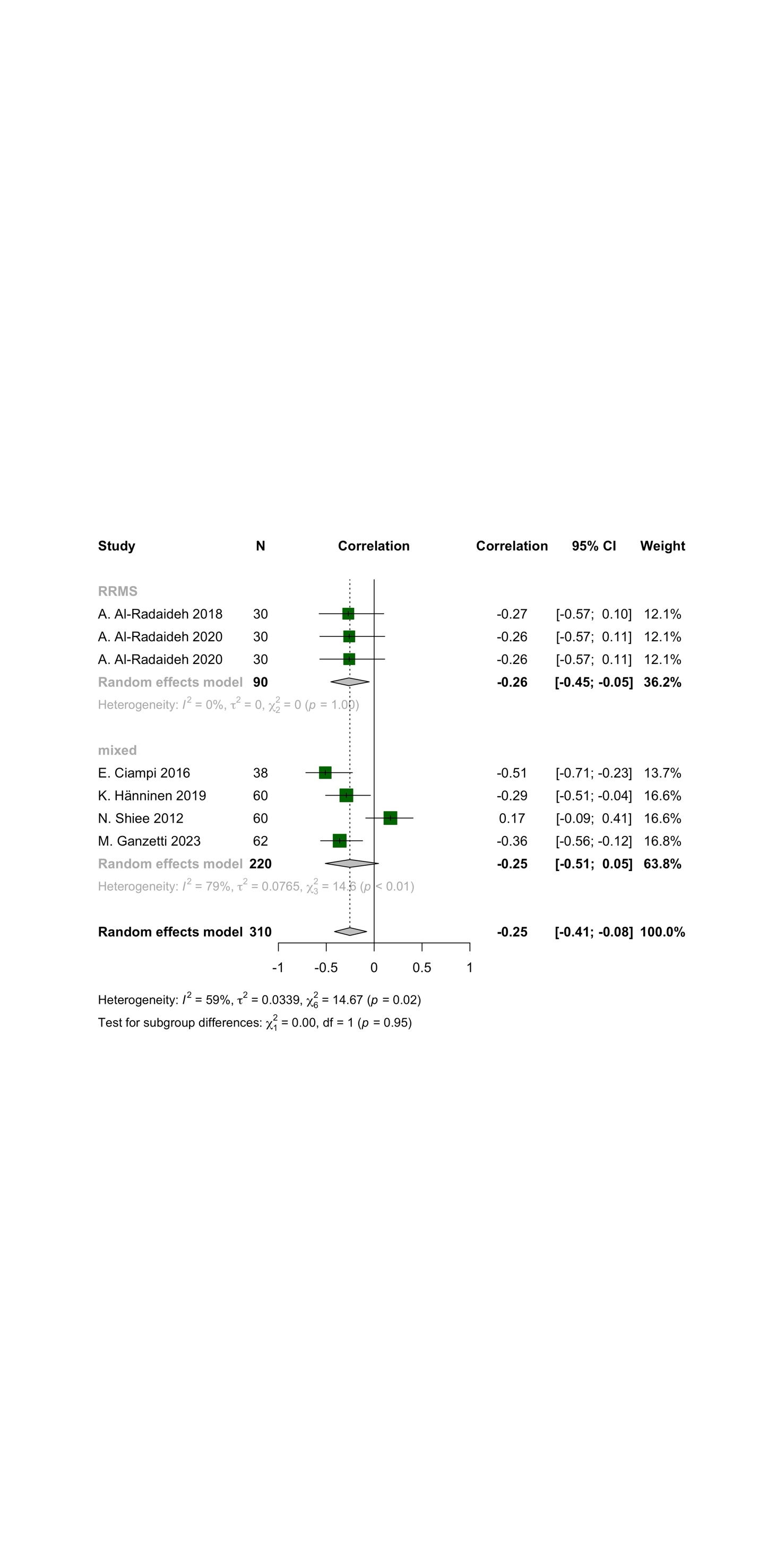


Figure S25. Forest plot of EDSS and normalized putamen volume correlation in pwMS.


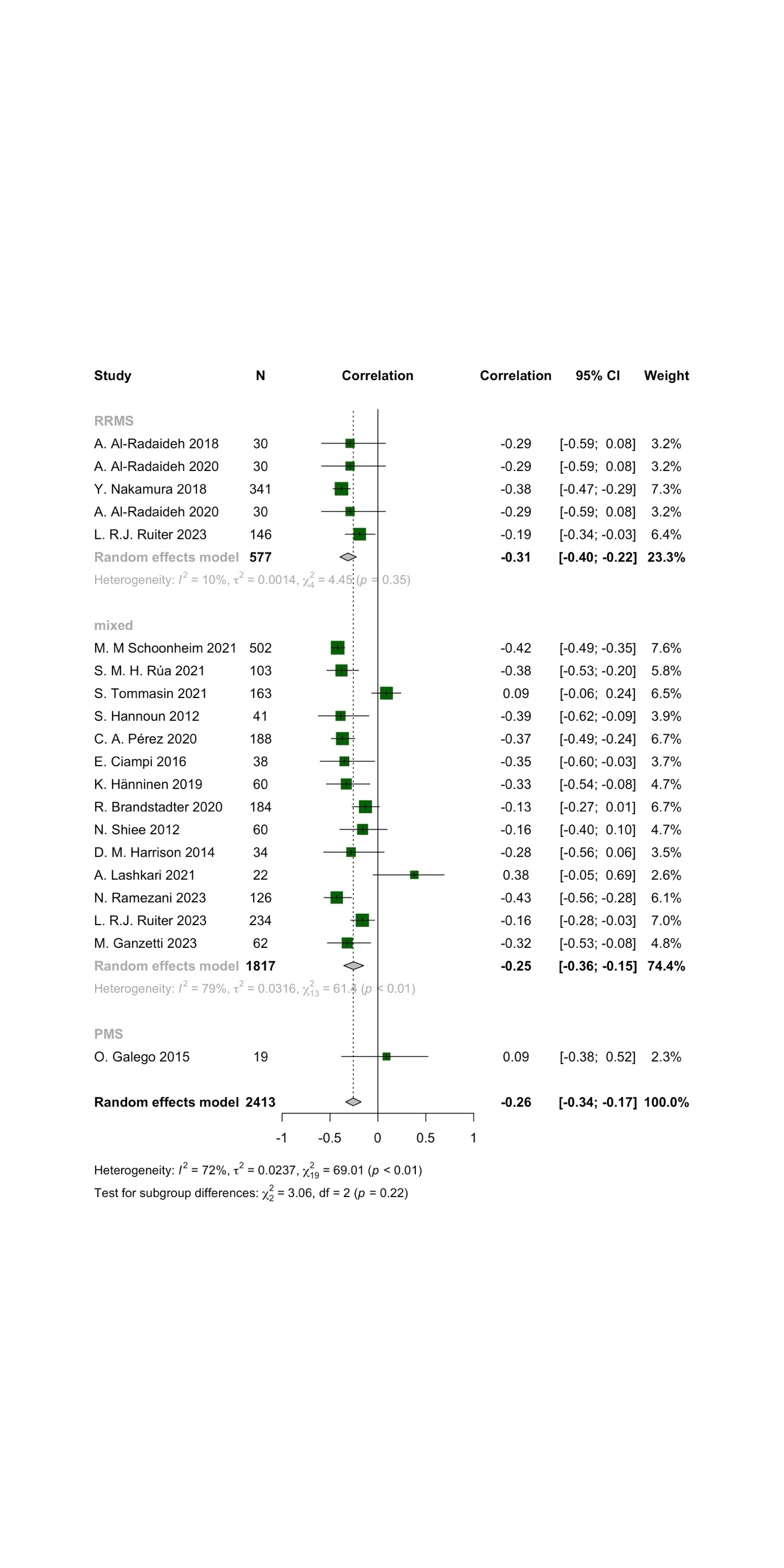


Figure S26. Forest plot of EDSS and normalized thalamus volume correlation in pwMS.


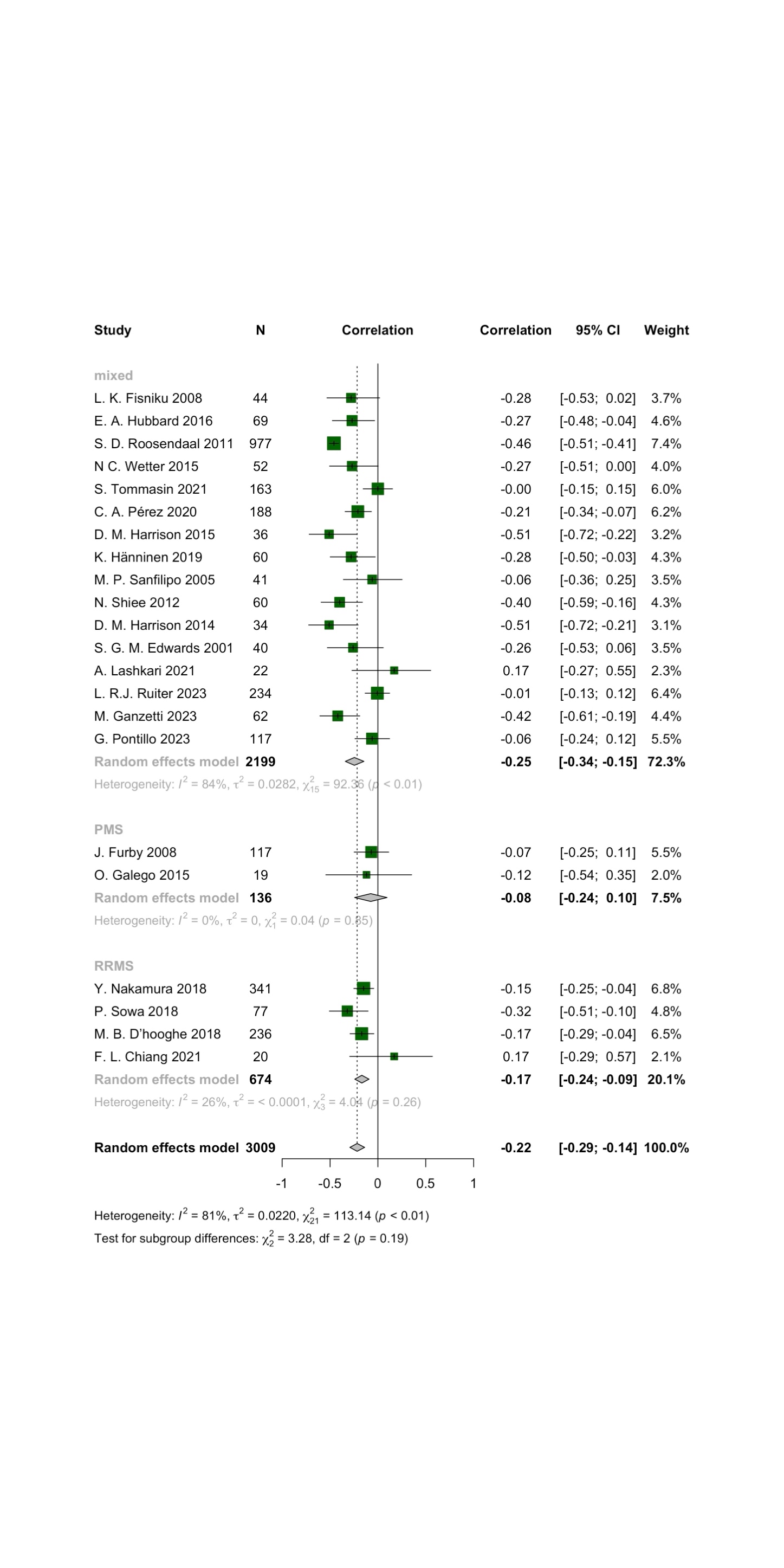


Figure S27. Forest plot of EDSS and normalized white matter volume correlation in pwMS.


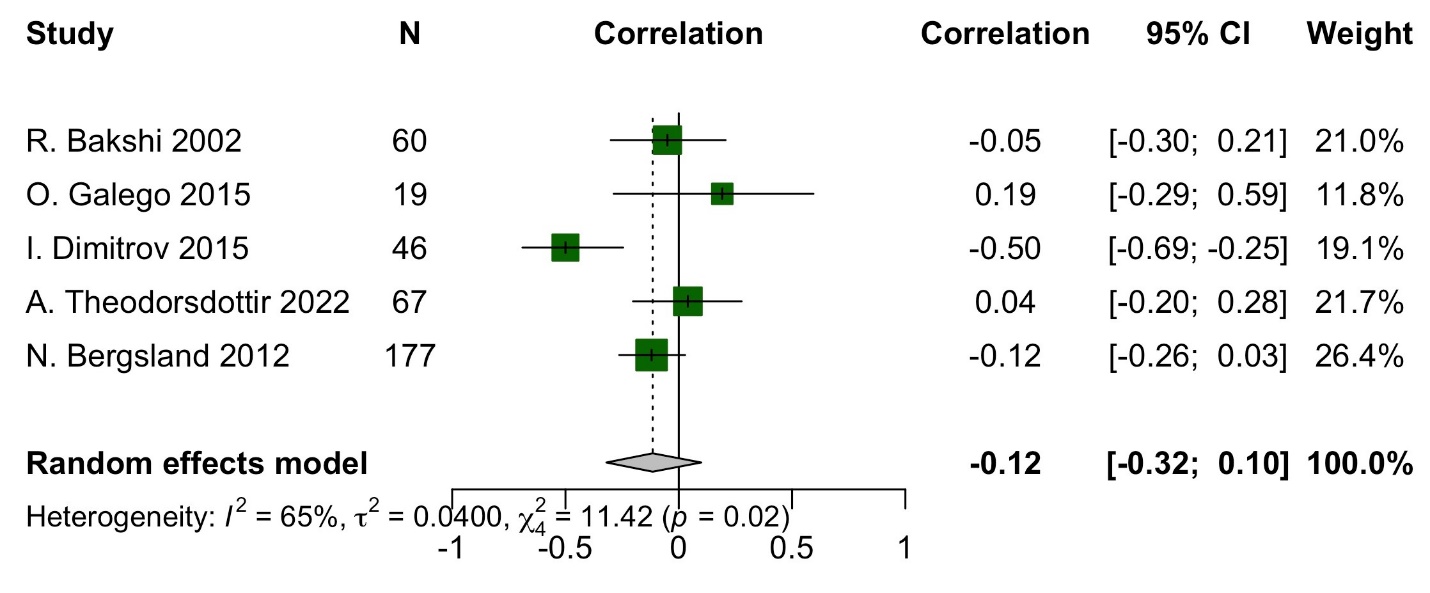


Figure S28. Forest plot of EDSS and putamen volume correlation in pwMS.


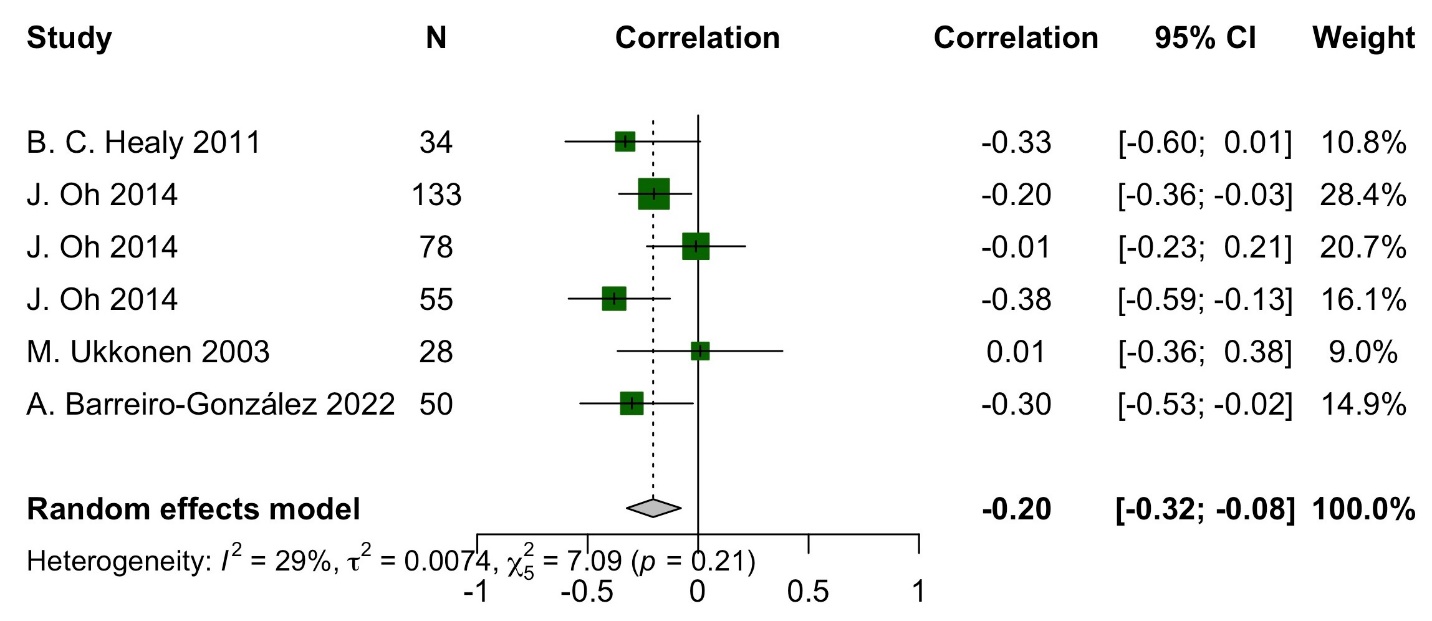


Figure S29. Forest plot of EDSS and spinal cord volume correlation in pwMS.


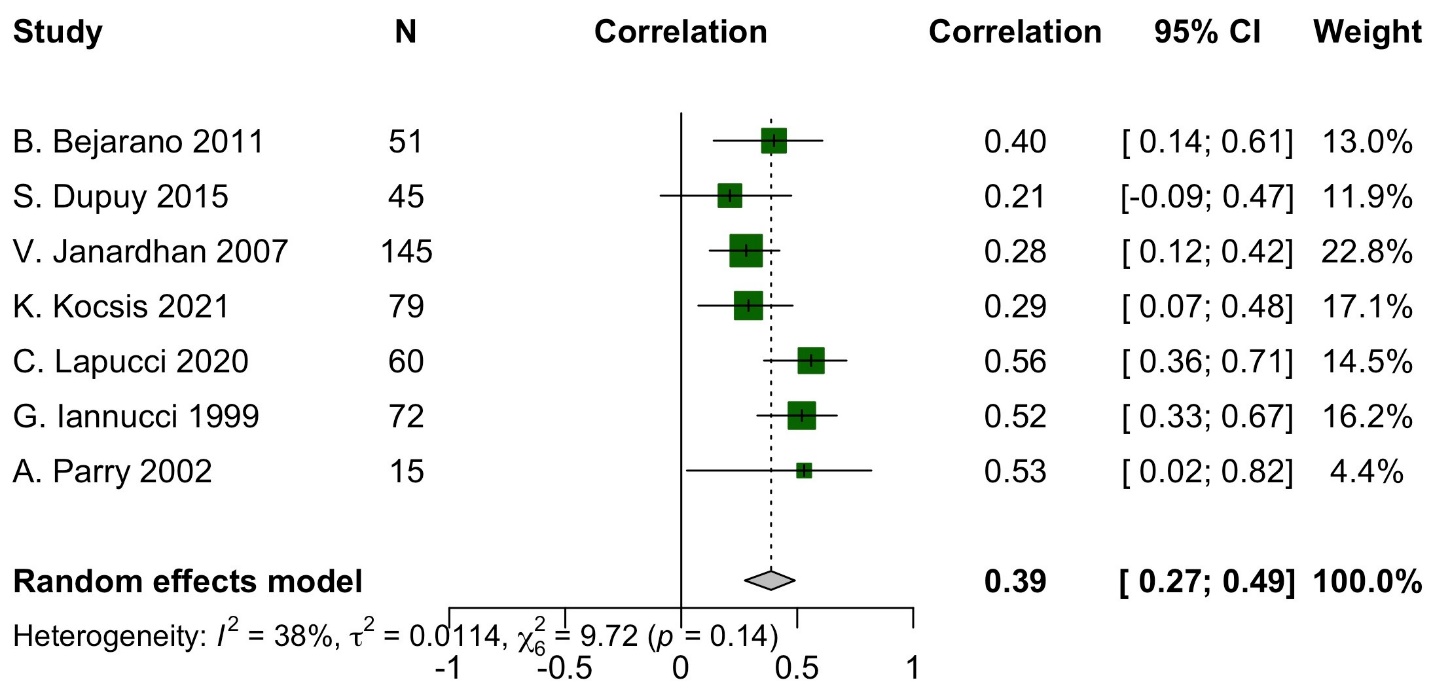


Figure S30. Forest plot of EDSS and T1 lesion count correlation in pwMS.


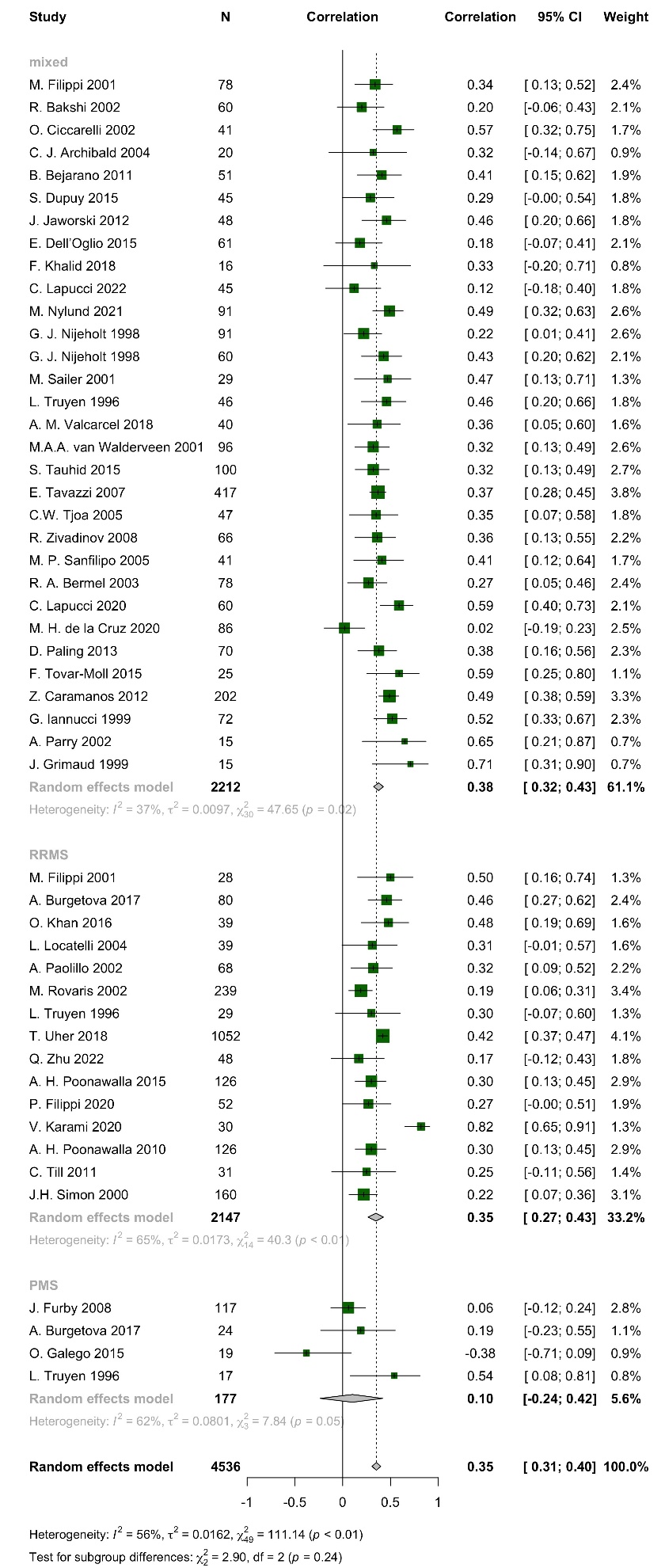


Figure S31. Forest plot of EDSS and T1 lesion volume correlation in pwMS.


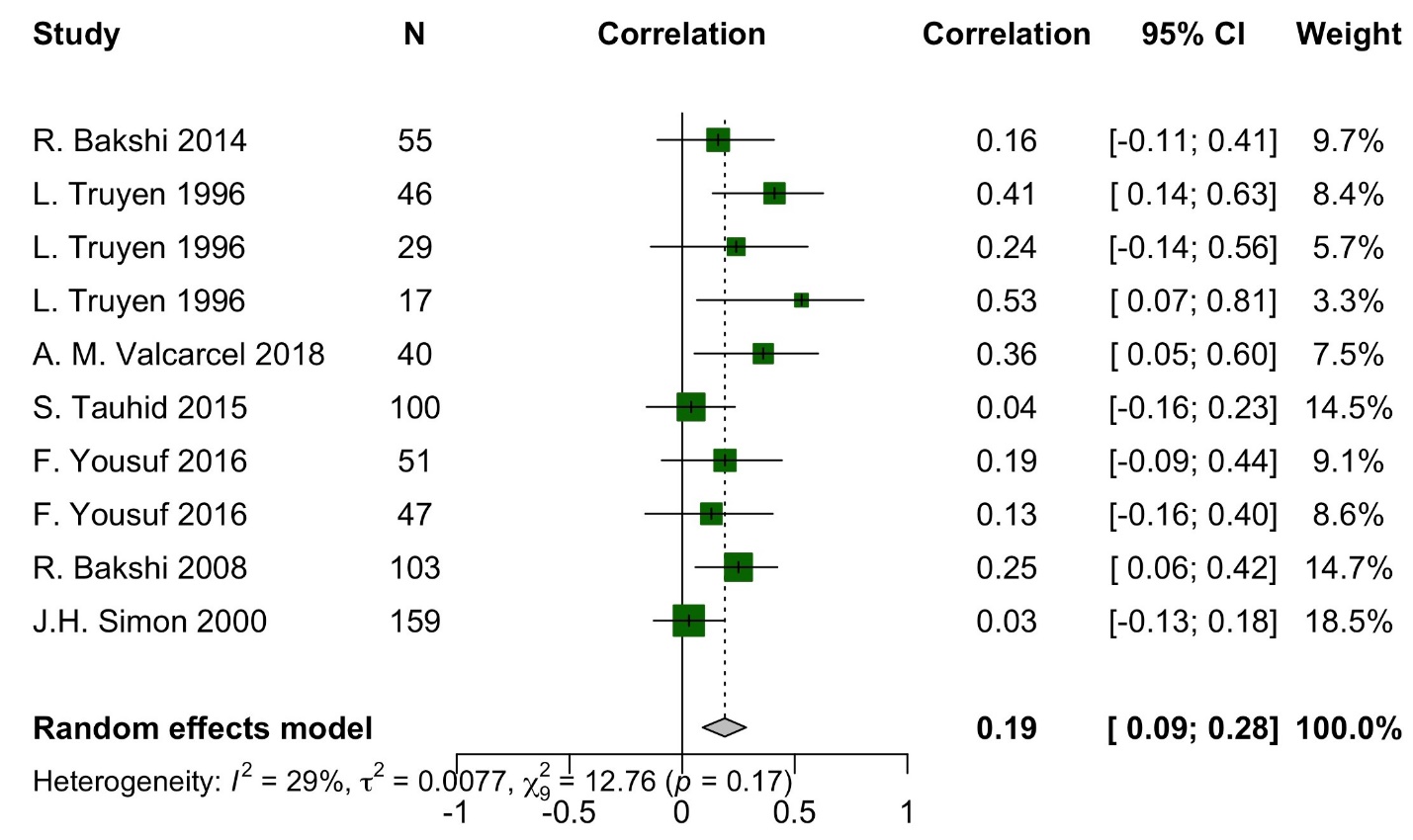


Figure S32. Forest plot of EDSS and T1LV/T2LV correlation in pwMS.


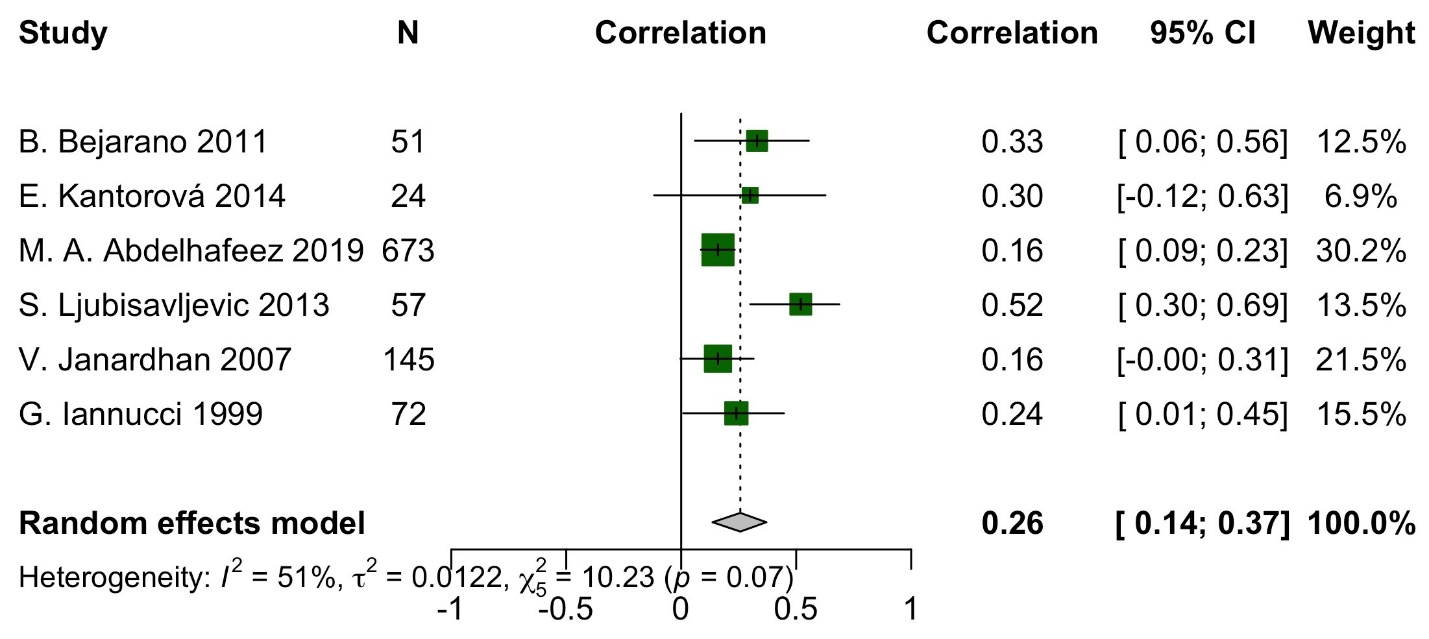


Figure S33. Forest plot of EDSS and T2 lesion count correlation in pwMS.


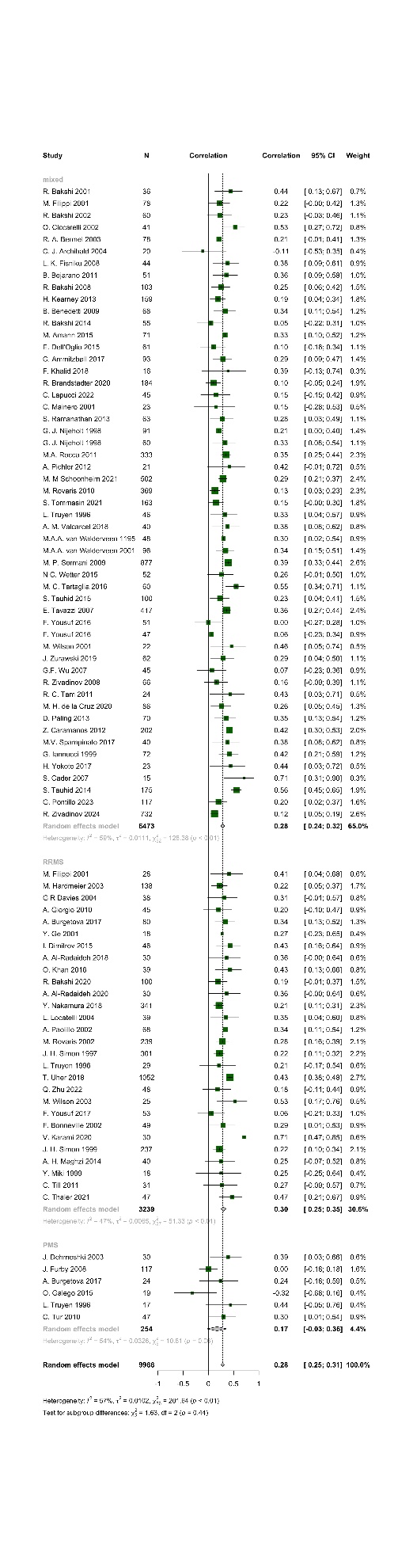


Figure S34. Forest plot of EDSS and T2 lesion volume correlation in pwMS.


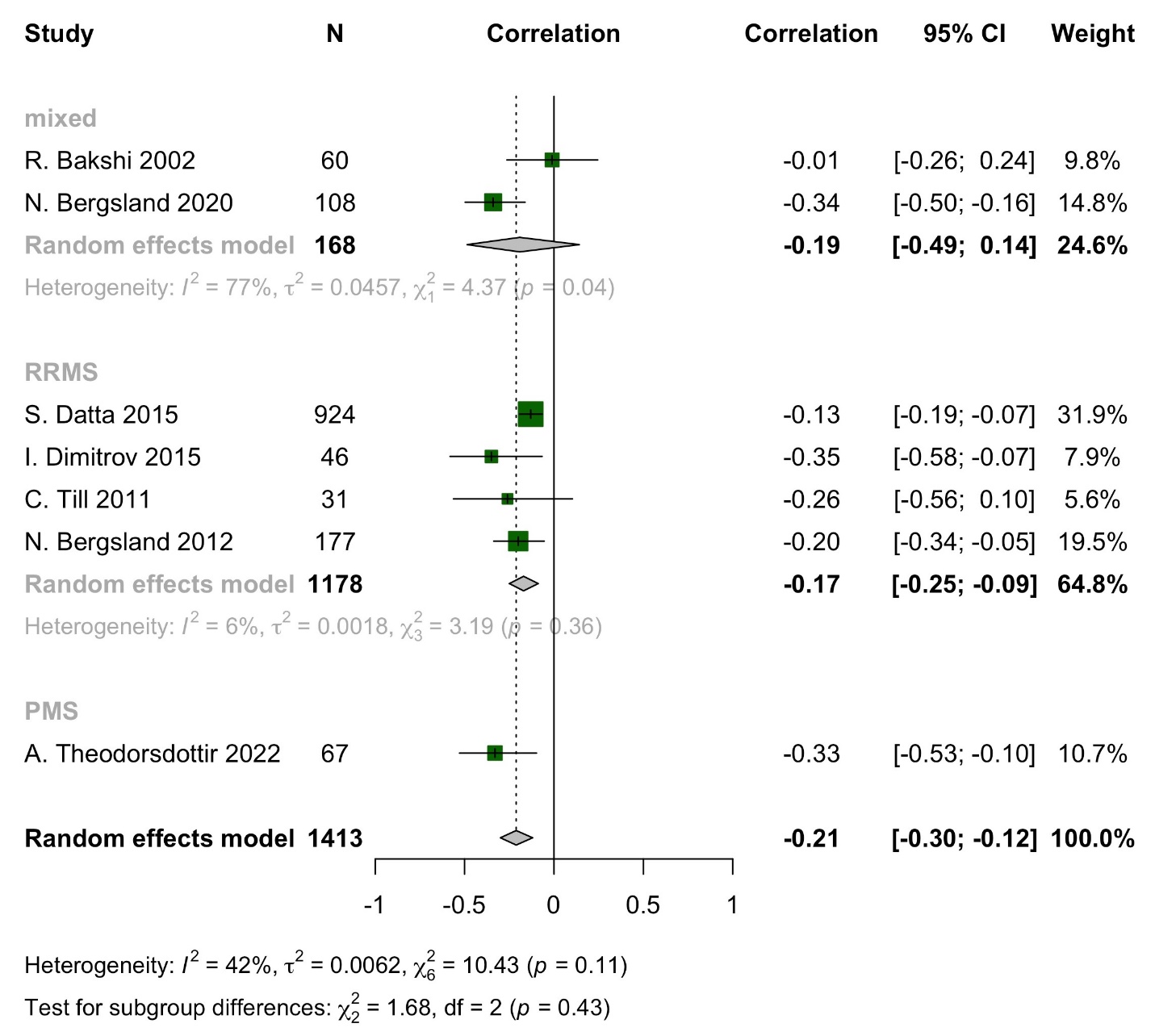


Figure S35. Forest plot of EDSS and thalamus volume correlation in pwMS.


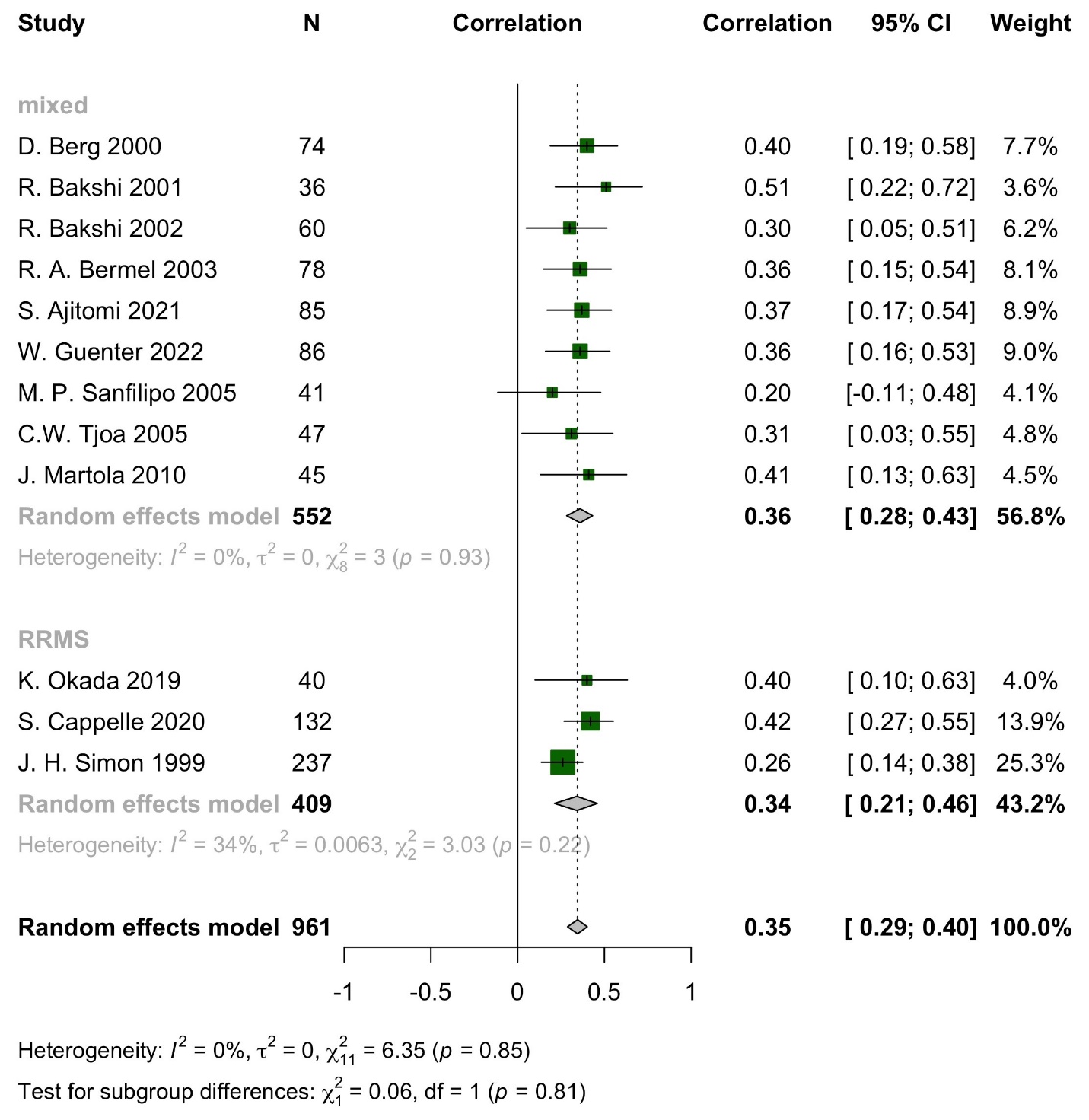


Figure S36. Forest plot of EDSS and third ventricular width correlation in pwMS.


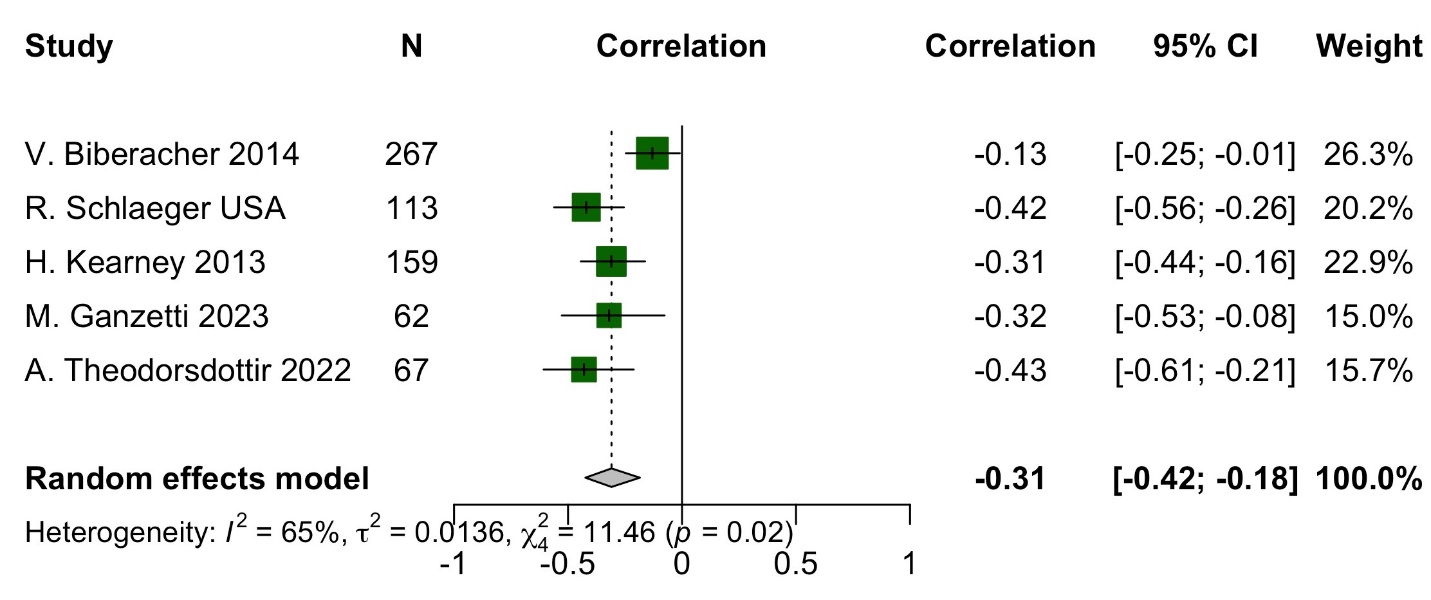


Figure S37. Forest plot of EDSS and upper cervical cord area at C2/C3 correlation in pwMS.


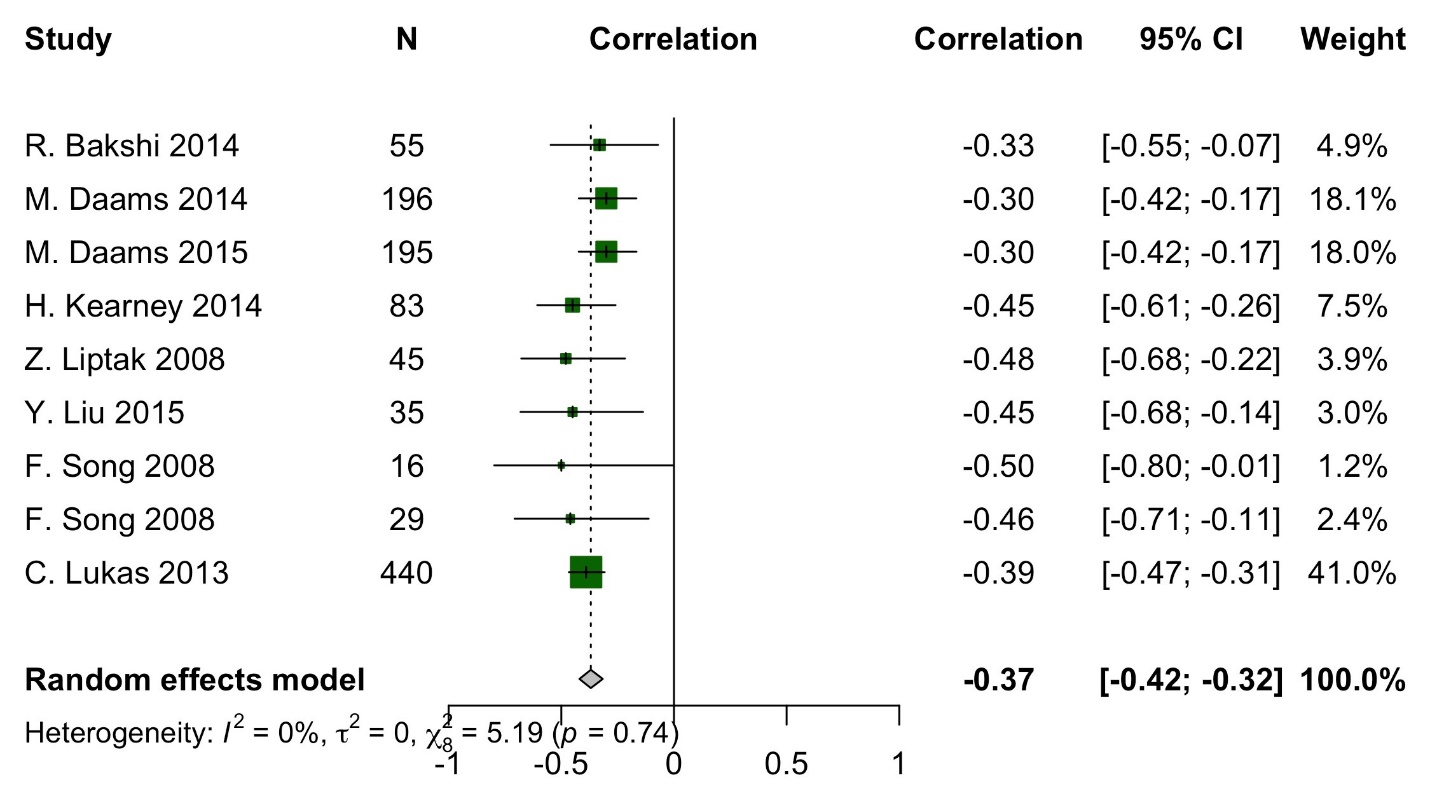


Figure S38. Forest plot of EDSS and upper cervical cord area correlation in pwMS.


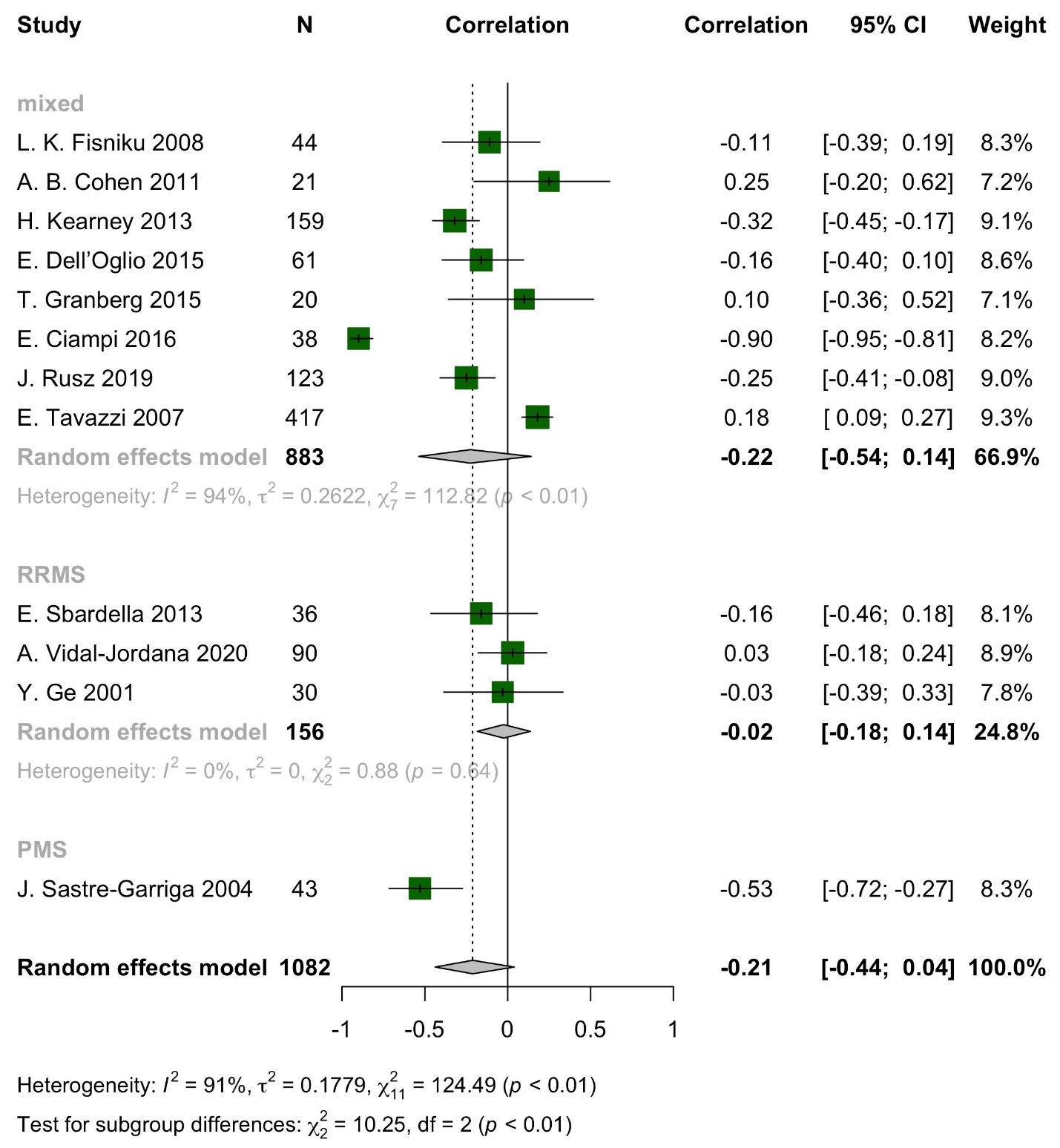


Figure S39. Forest plot of EDSS and white matter fraction correlation in pwMS.


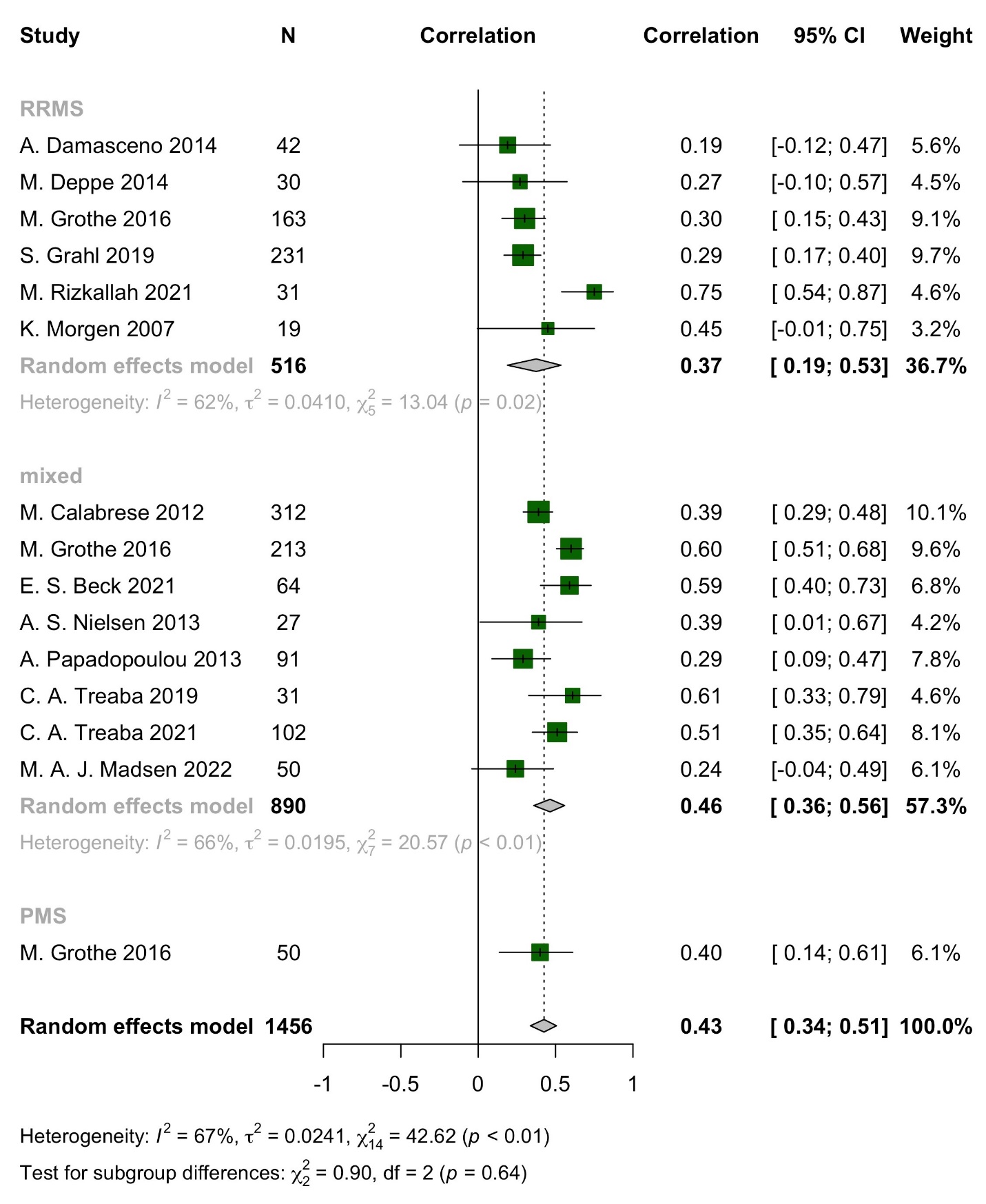


Figure S40. Forest plot of EDSS and white matter lesion volume correlation in pwMS.


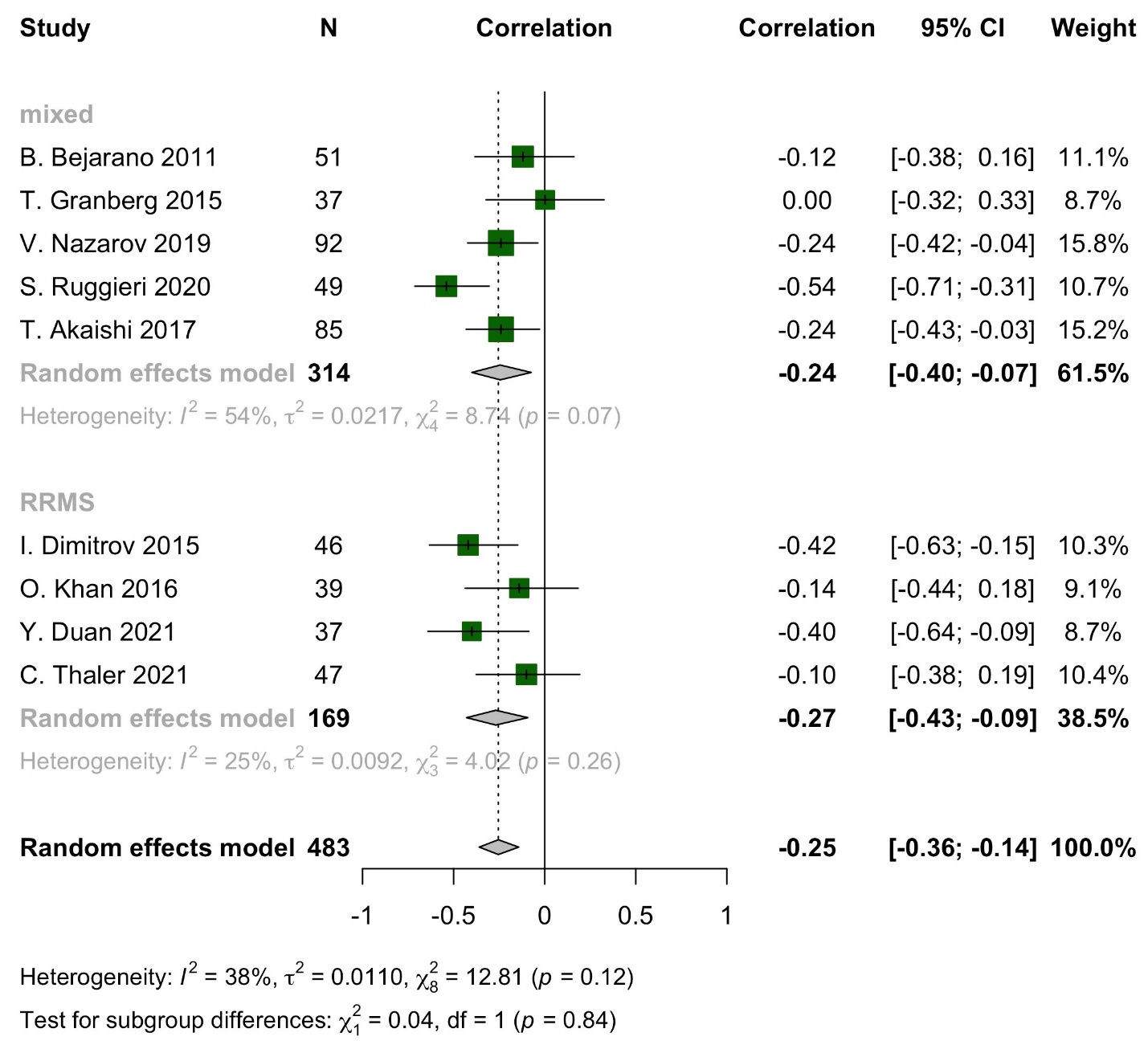


Figure S41. Forest plot of EDSS and white matter volume correlation in pwMS.


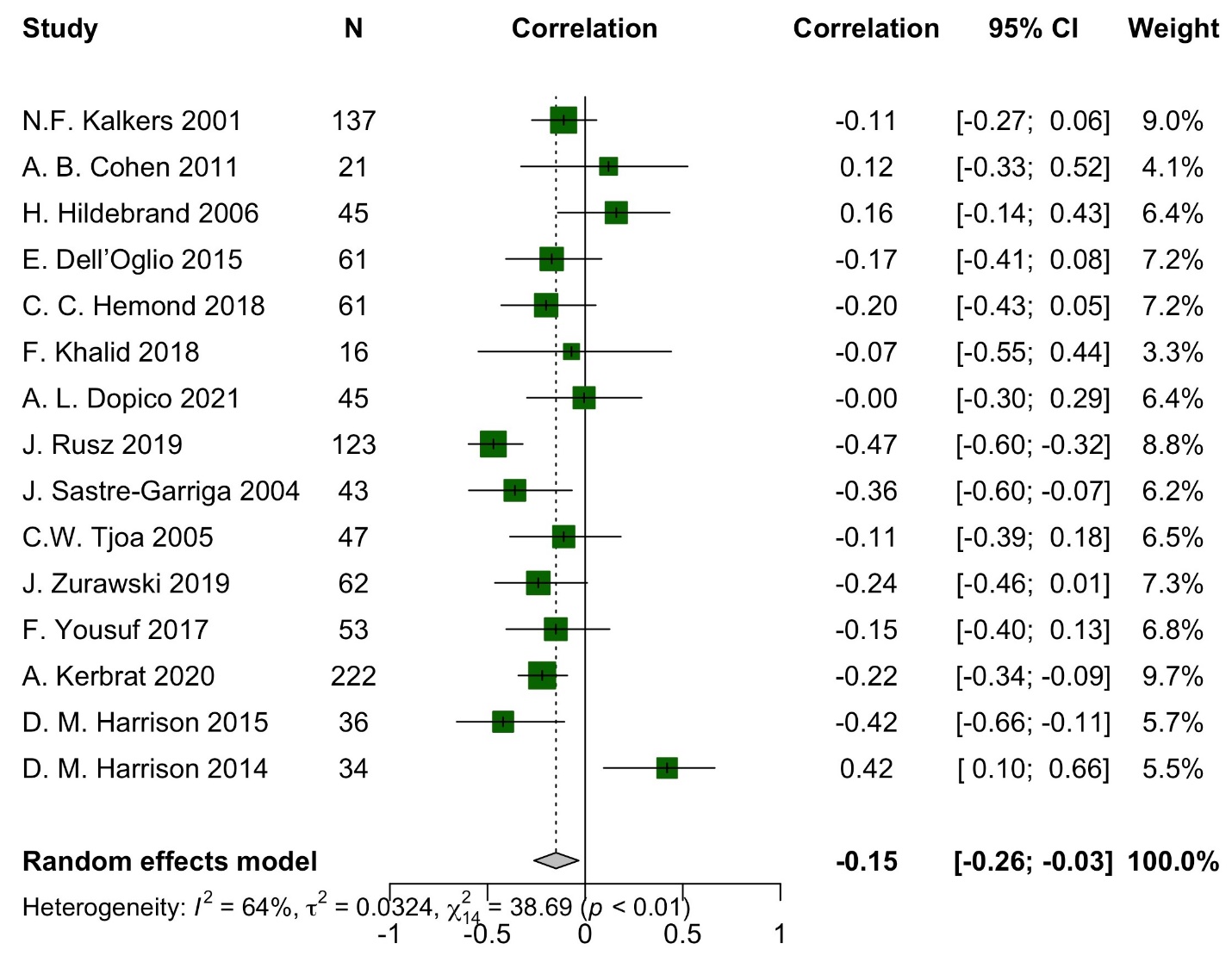


Figure S42. Forest plot of T25FW and BPF correlation in pwMS.


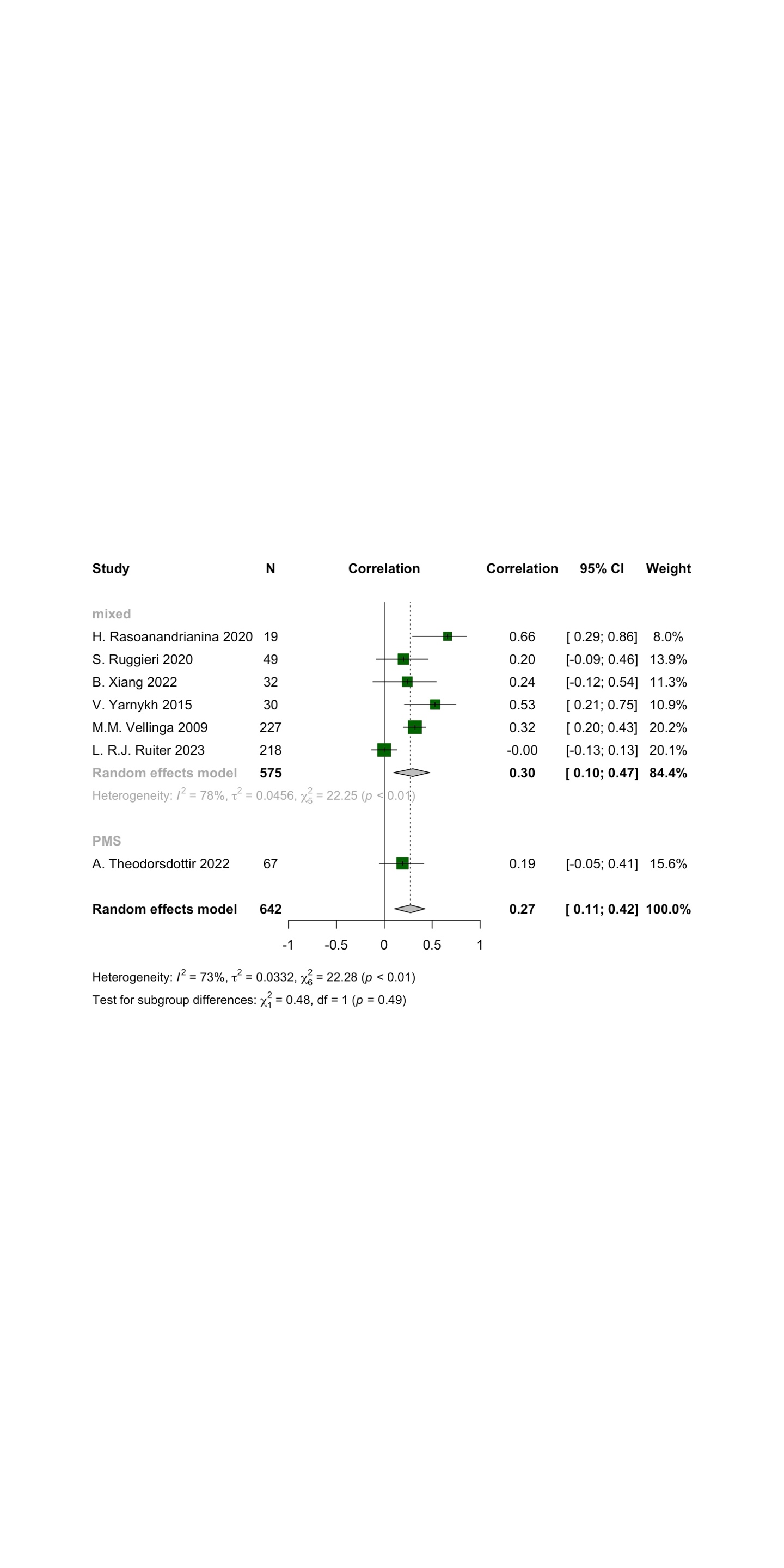


Figure S43. Forest plot of T25FW and brain lesion volume correlation in pwMS.


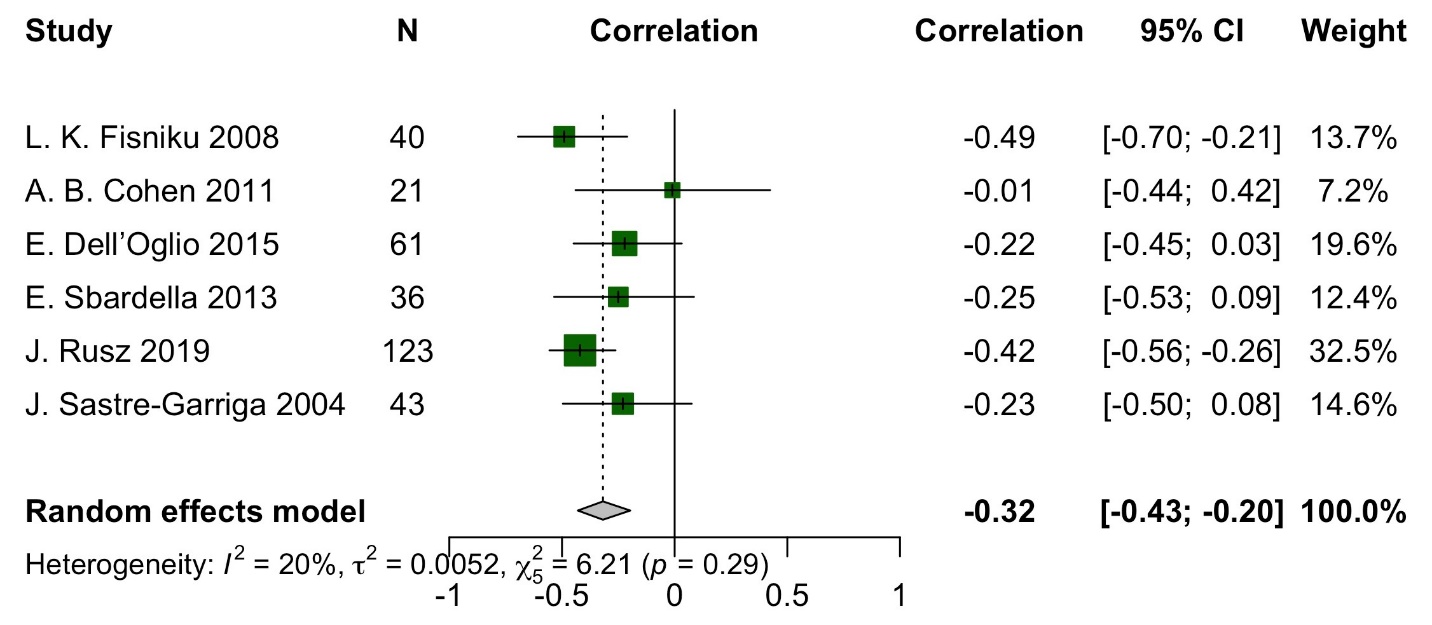


Figure S44. Forest plot of T25FW and grey matter fraction correlation in pwMS.


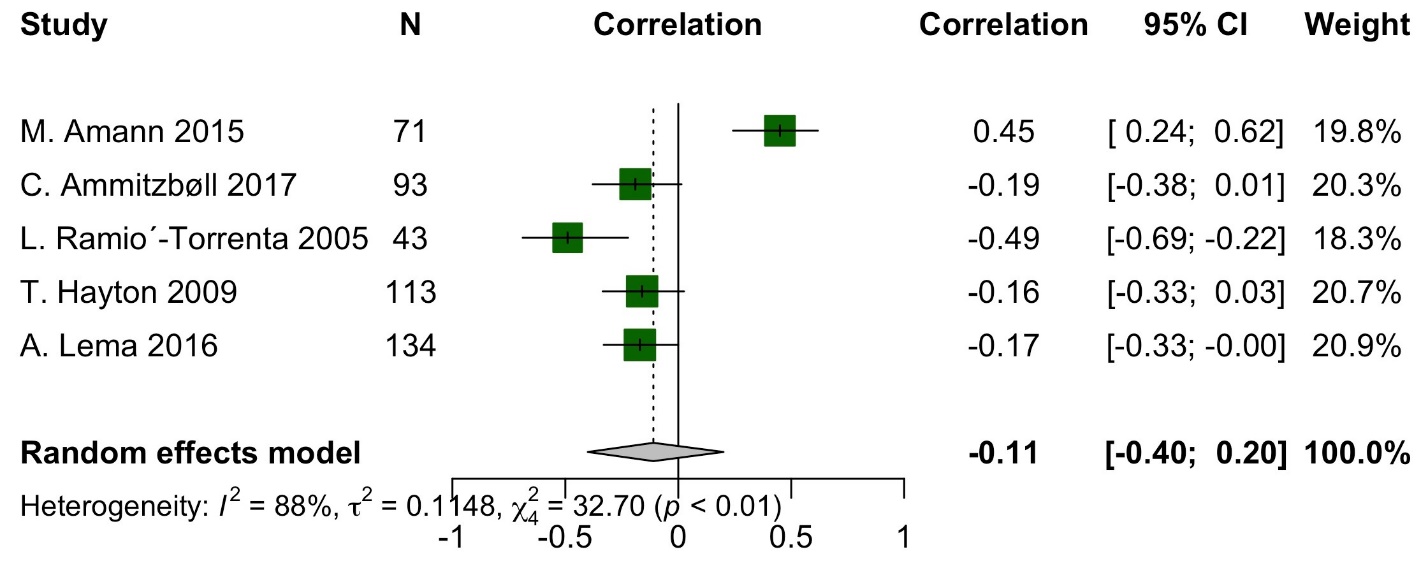


Figure S45. Forest plot of T25FW and normal-appearing white matter MTR correlation in pwMS.


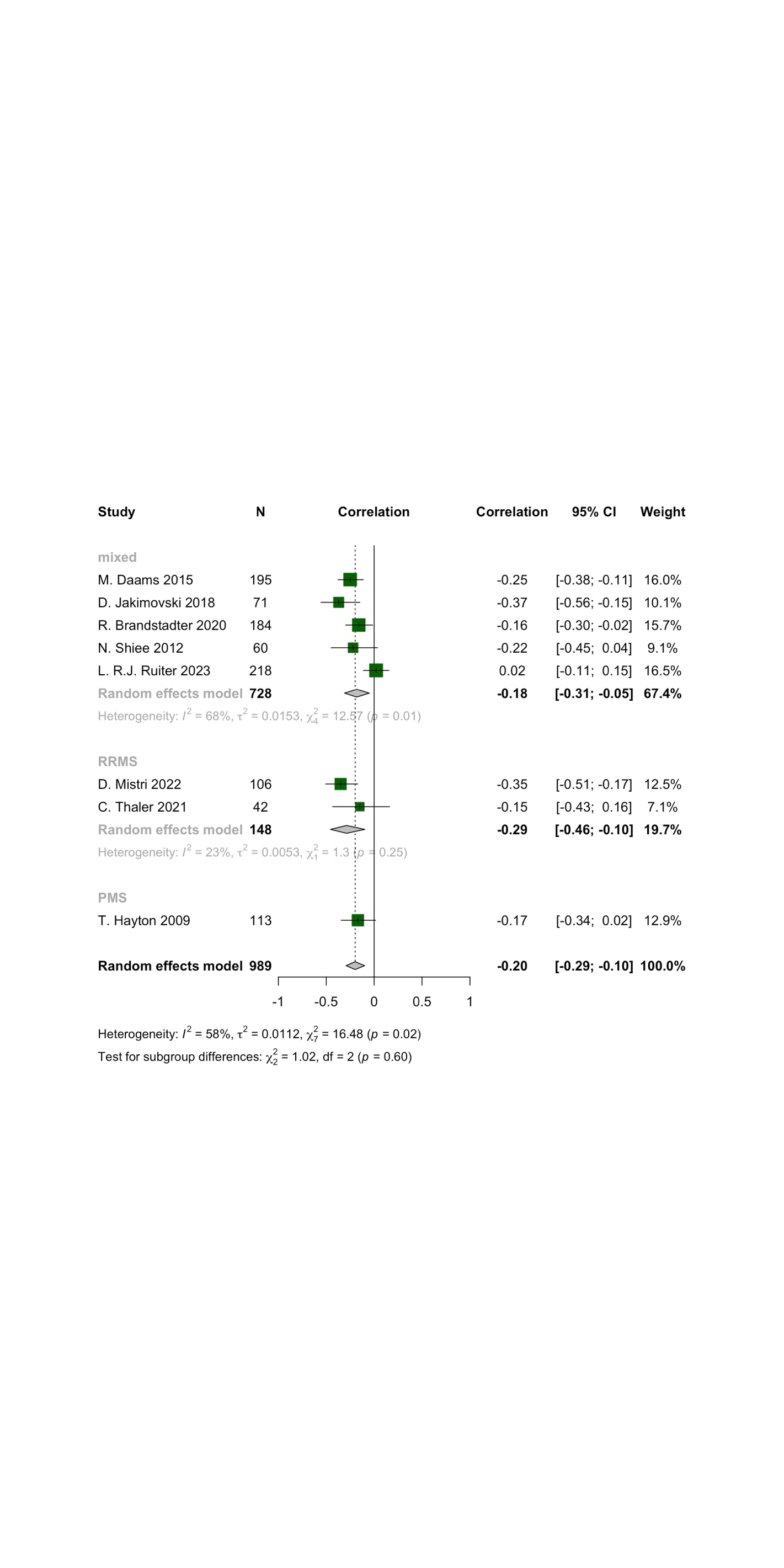


Figure S46. Forest plot of T25FW and normalized brain volume correlation in pwMS.


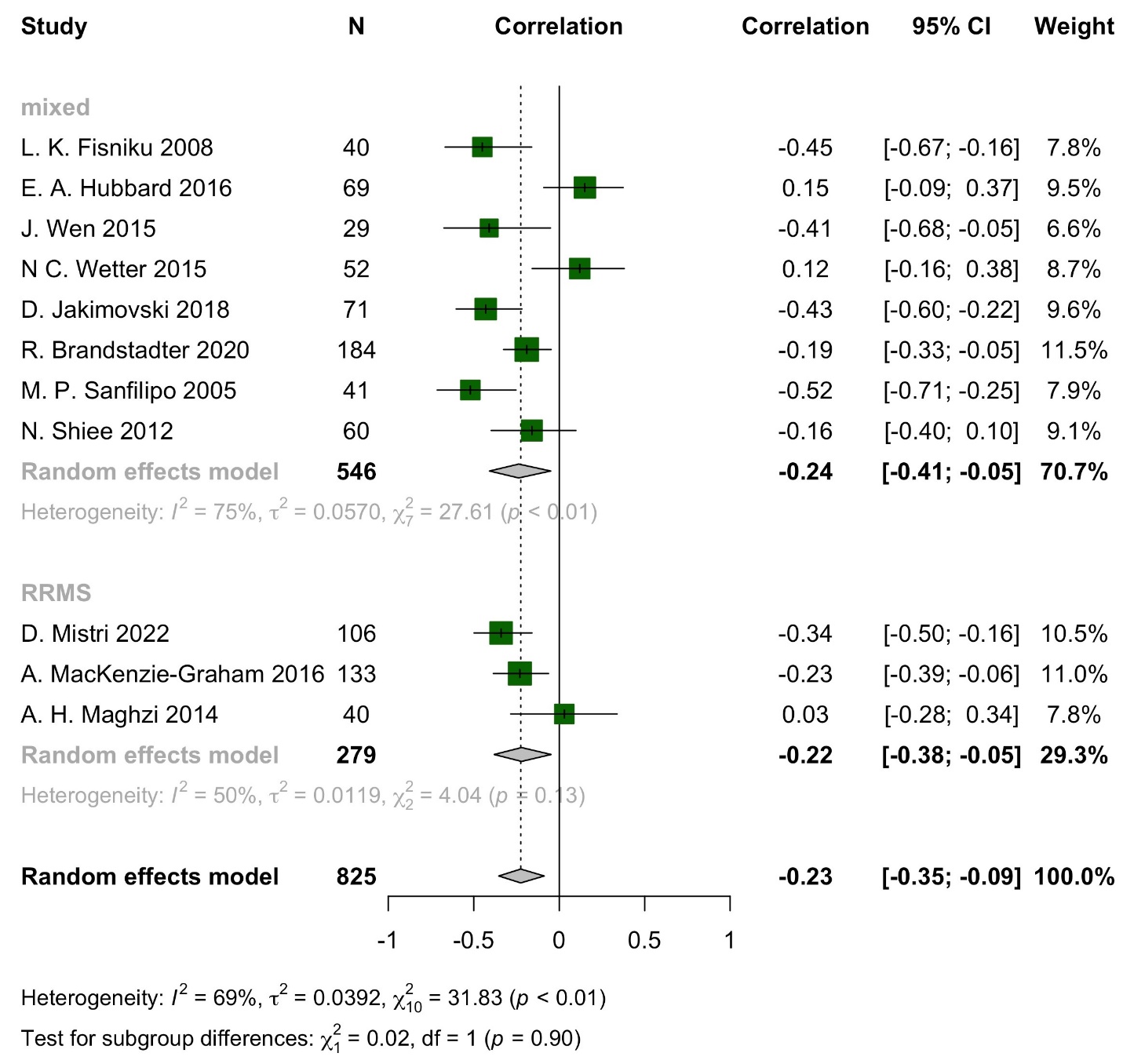


Figure S47. Forest plot of T25FW and normalized grey matter volume correlation in pwMS.


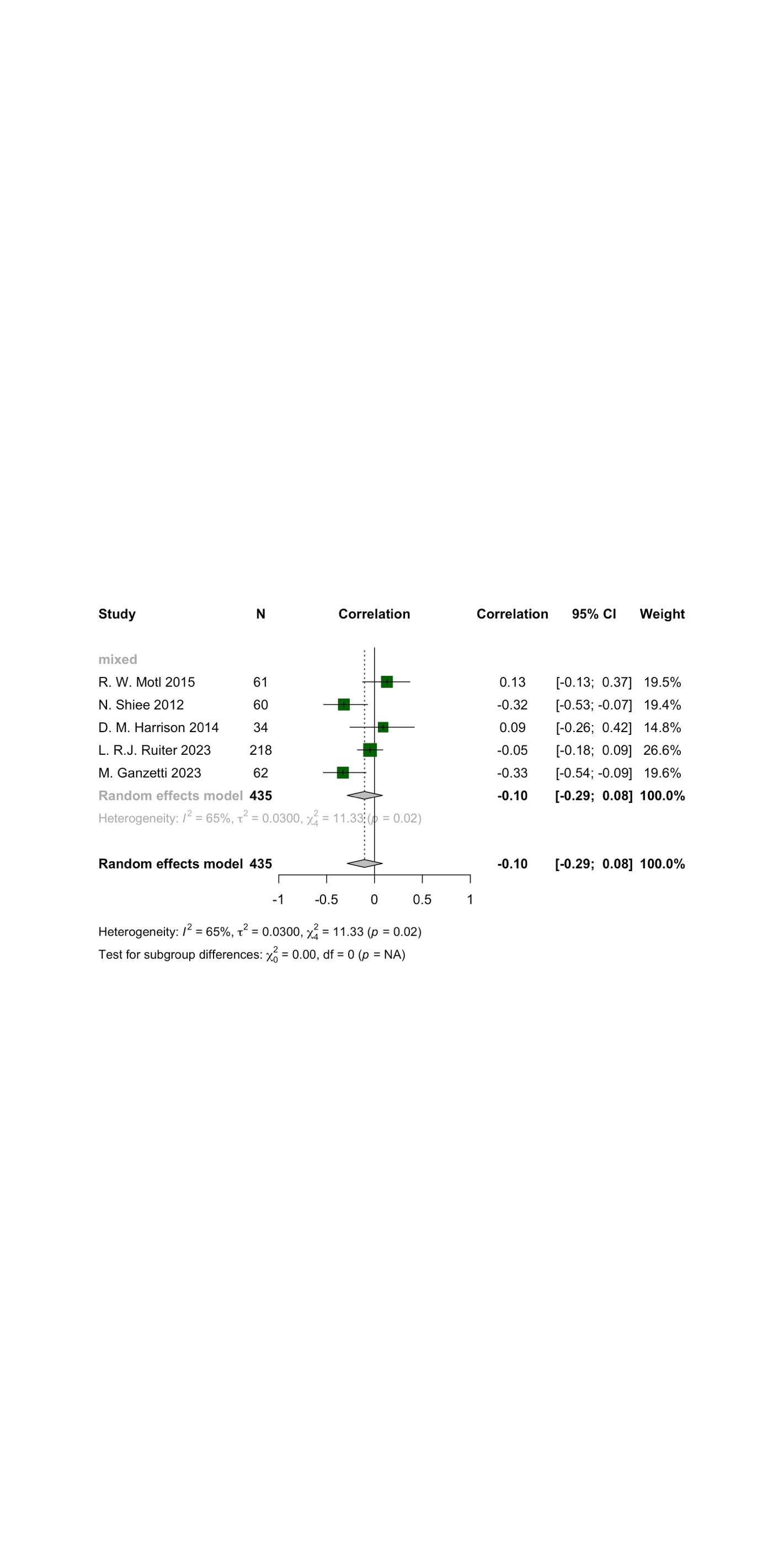


Figure S48. Forest plot of T25FW and normalized thalamus volume correlation in pwMS.


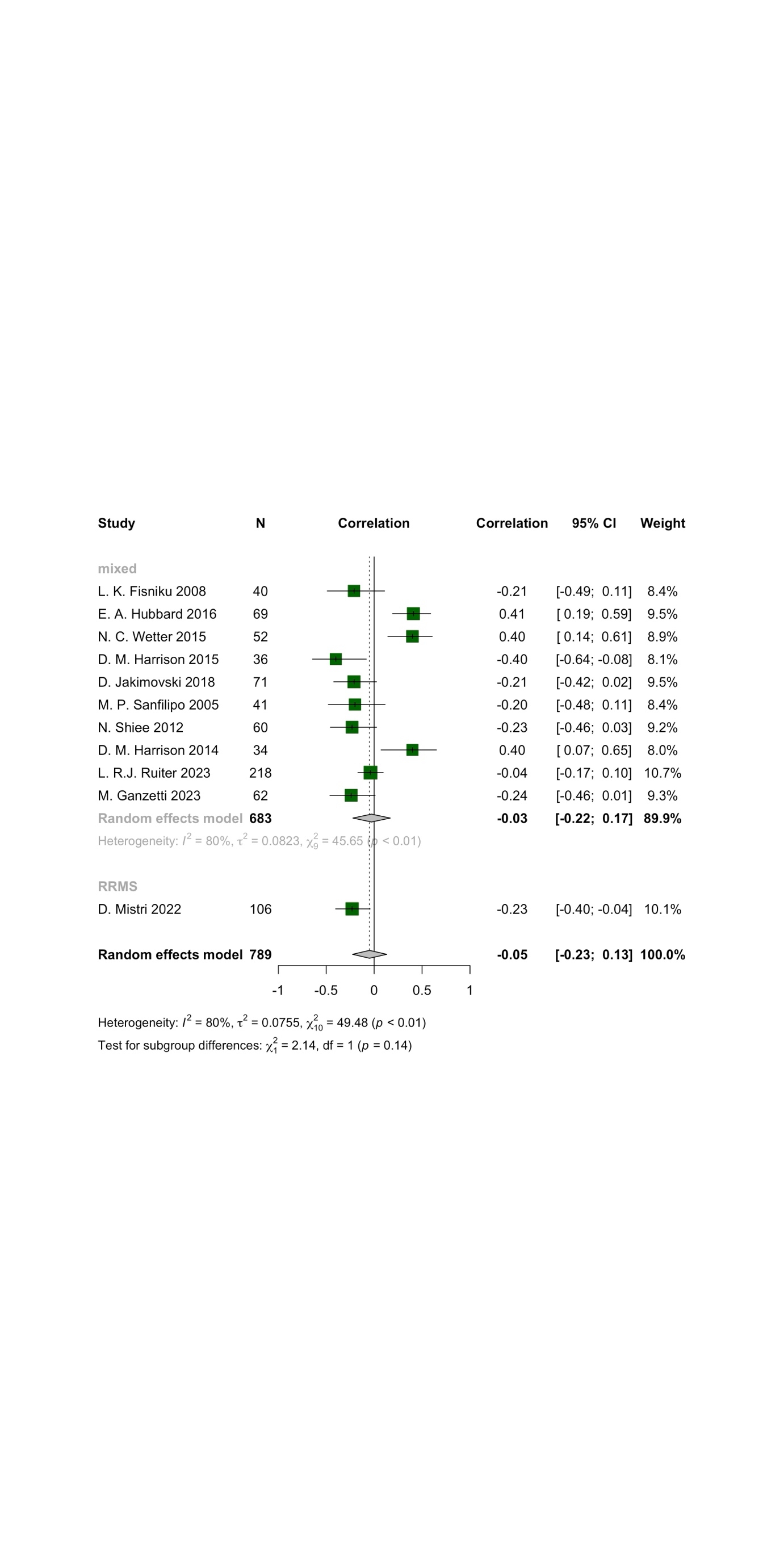


Figure S49. Forest plot of T25FW and normalized white matter volume correlation in pwMS.


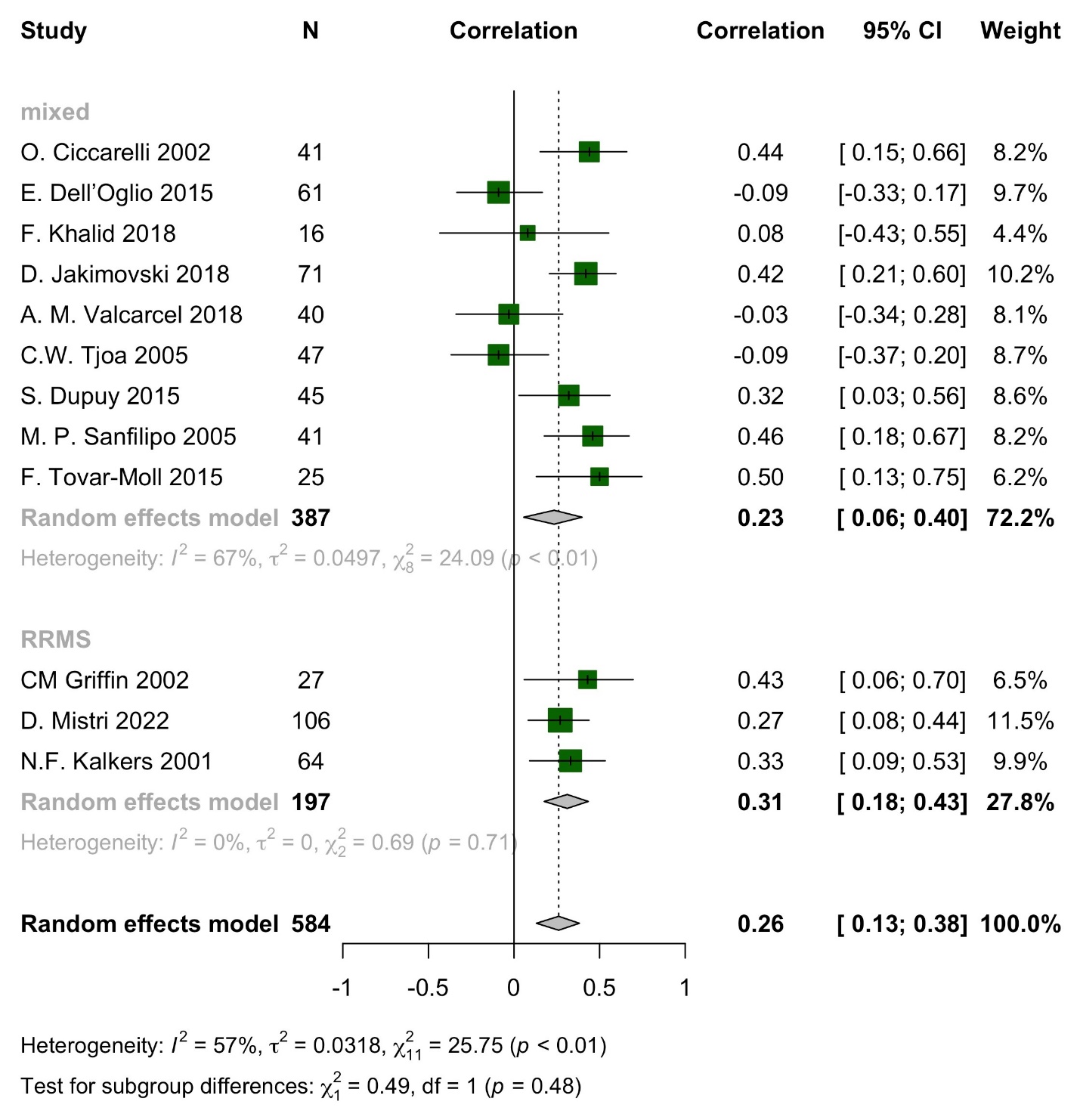


Figure S50. Forest plot of T25FW and T1 lesion volume correlation in pwMS.


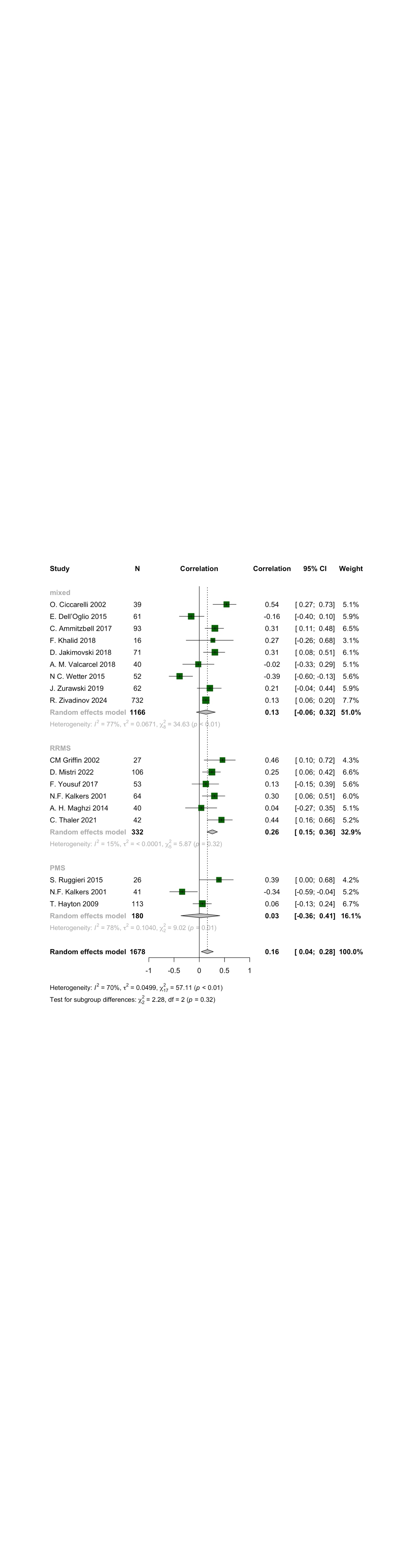


Figure S51. Forest plot of T25FW and T2 lesion volume correlation in pwMS.


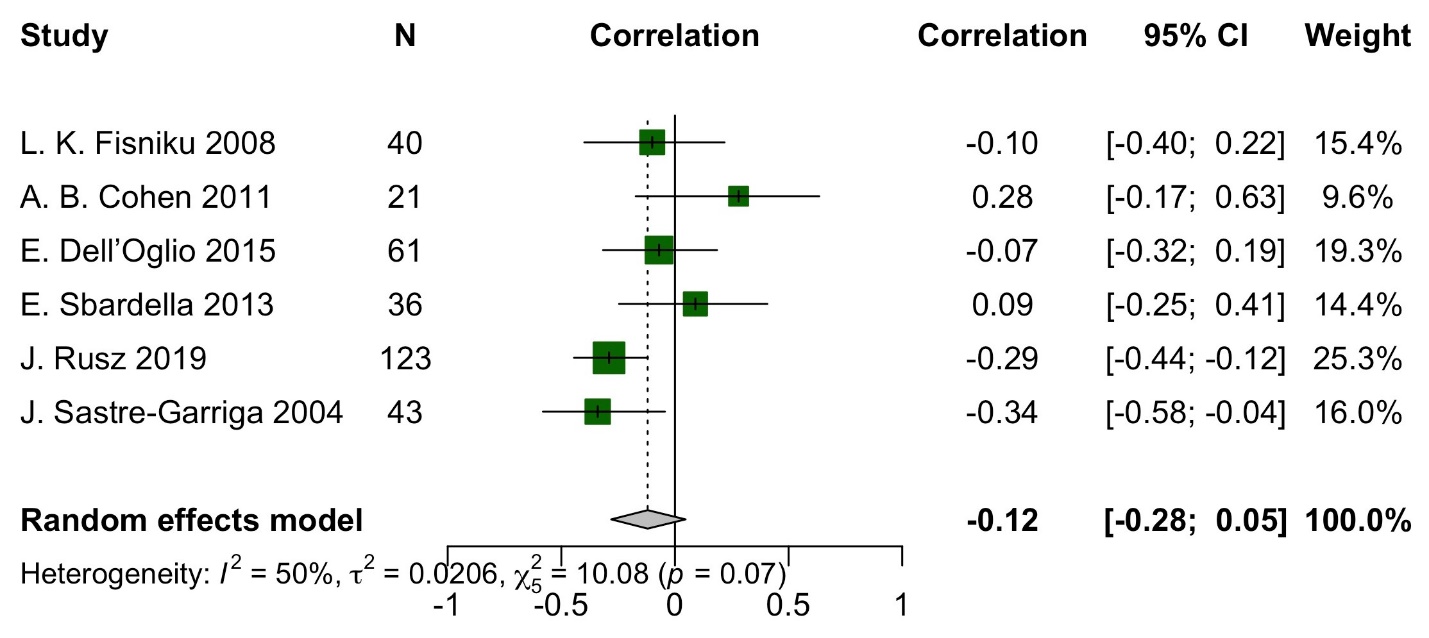


Figure S52. Forest plot of T25FW and white matter fraction correlation in pwMS.


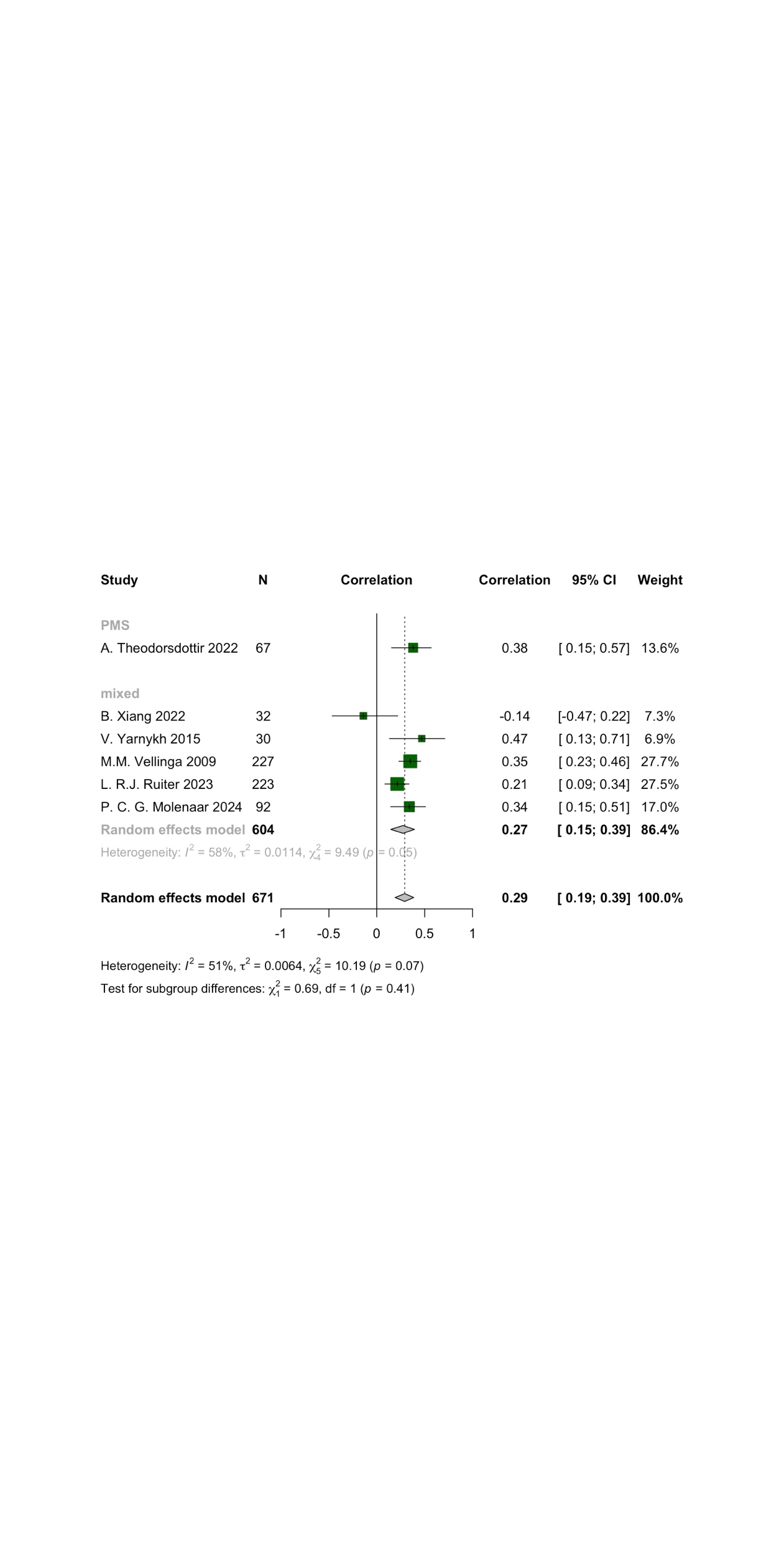


Figure S53. Forest plot of 9HPT and brain lesion volume correlation in pwMS.


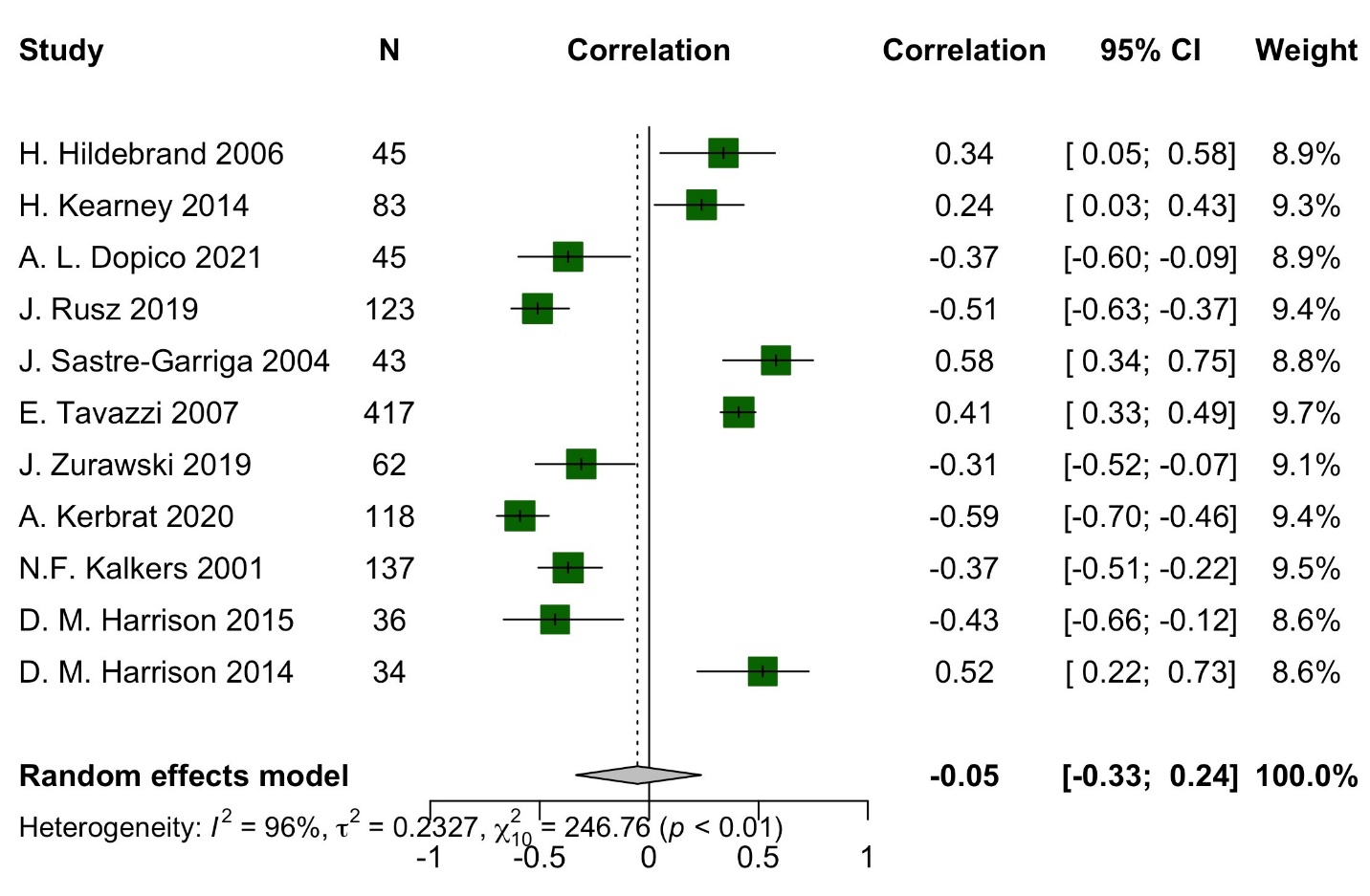


Figure S54. Forest plot of 9HPT and BPF correlation in pwMS.


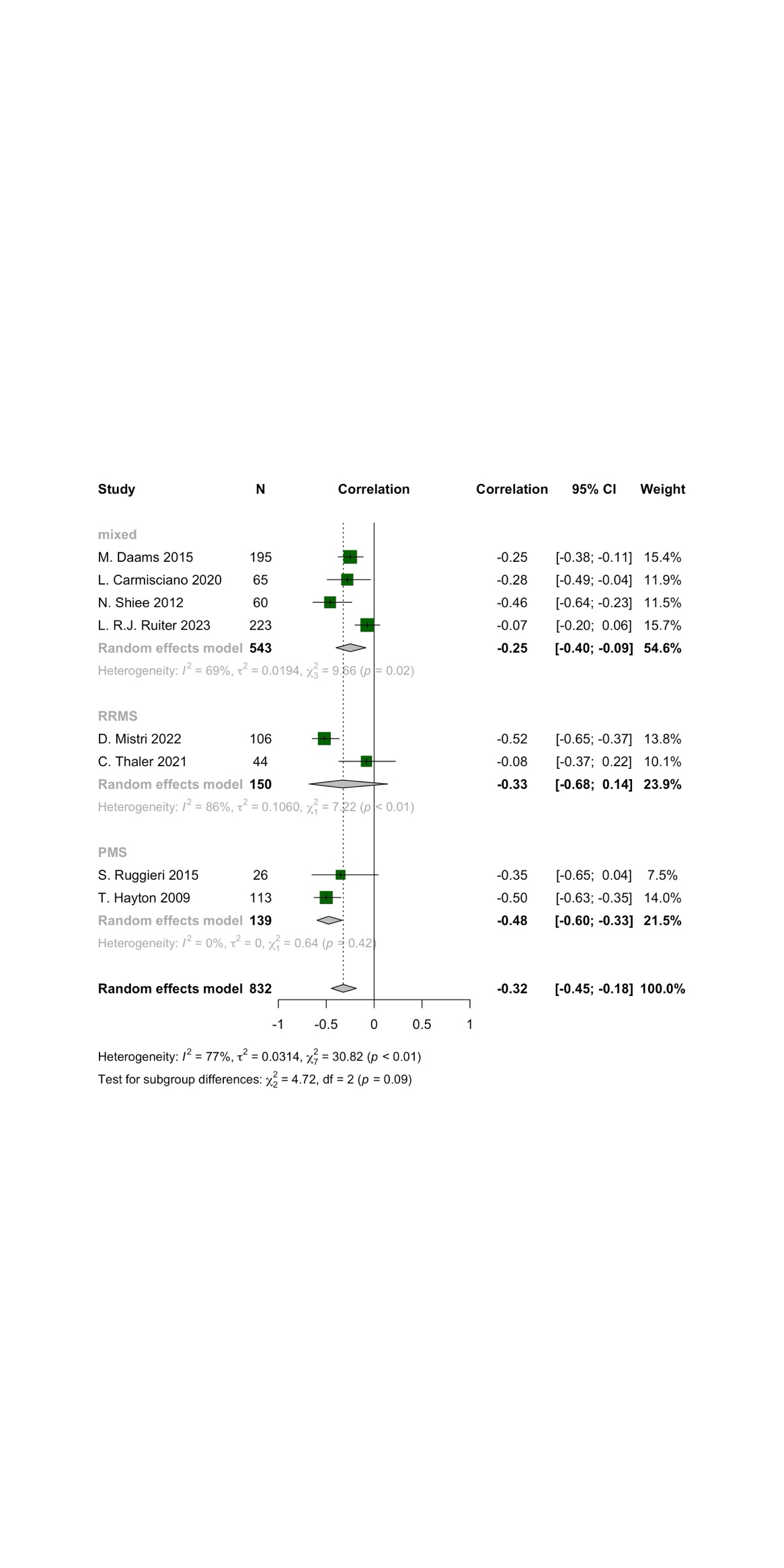


Figure S55. Forest plot of 9HPT and normalized brain volume correlation in pwMS.


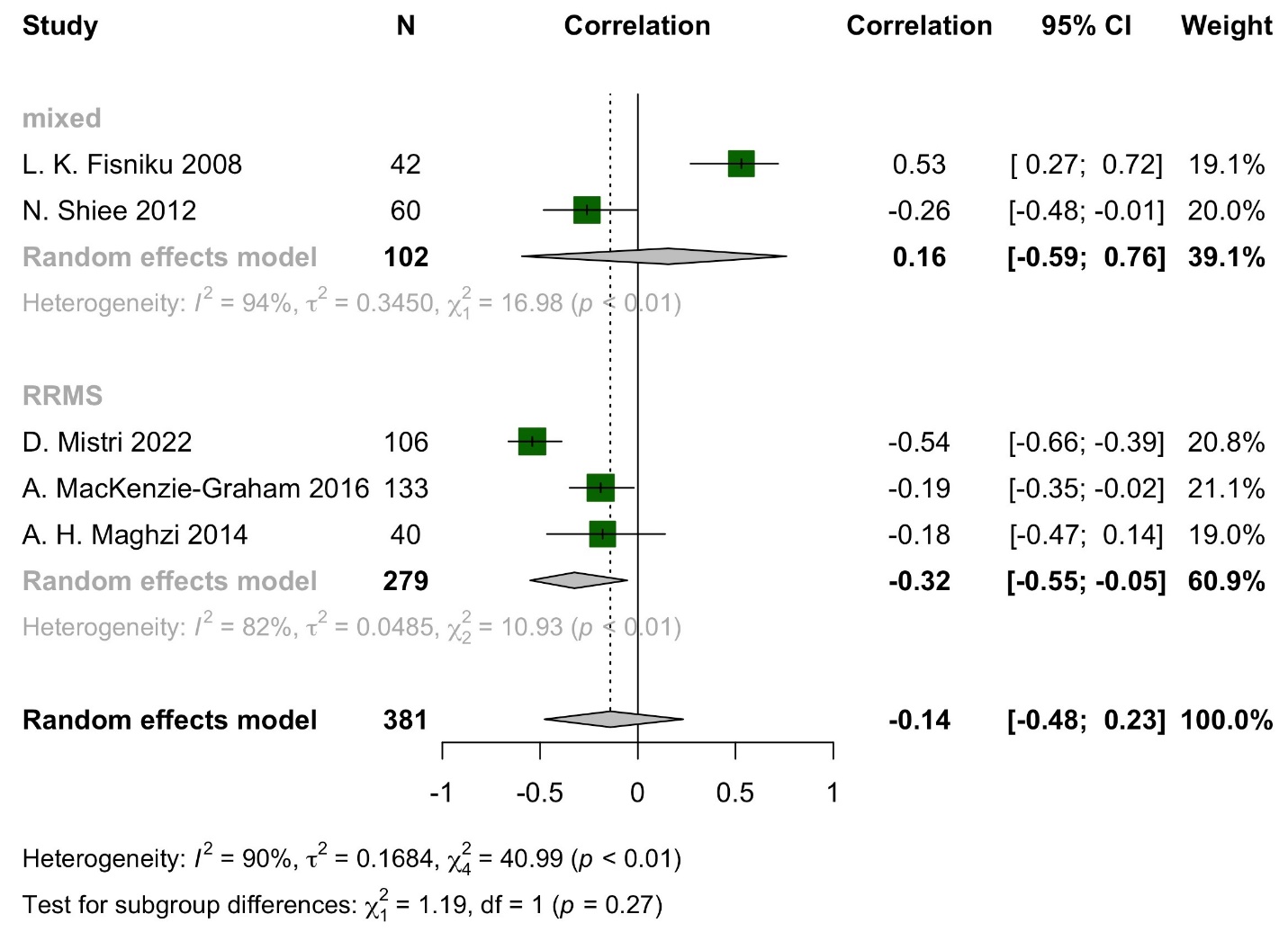


Figure S56. Forest plot of 9HPT and normalized grey matter volume correlation in pwMS.


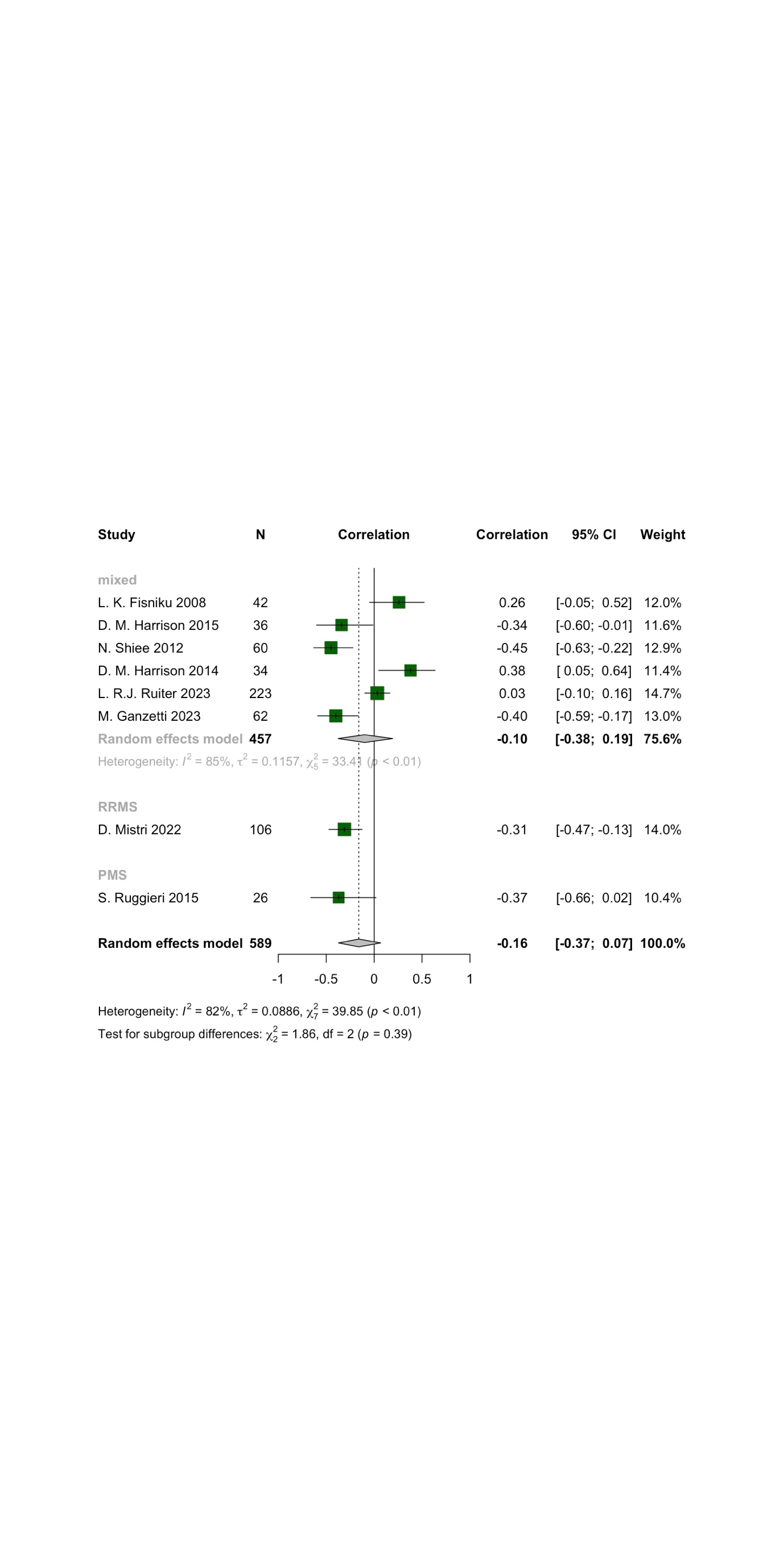


Figure S57. Forest plot of 9HPT and normalized white matter volume correlation in pwMS.


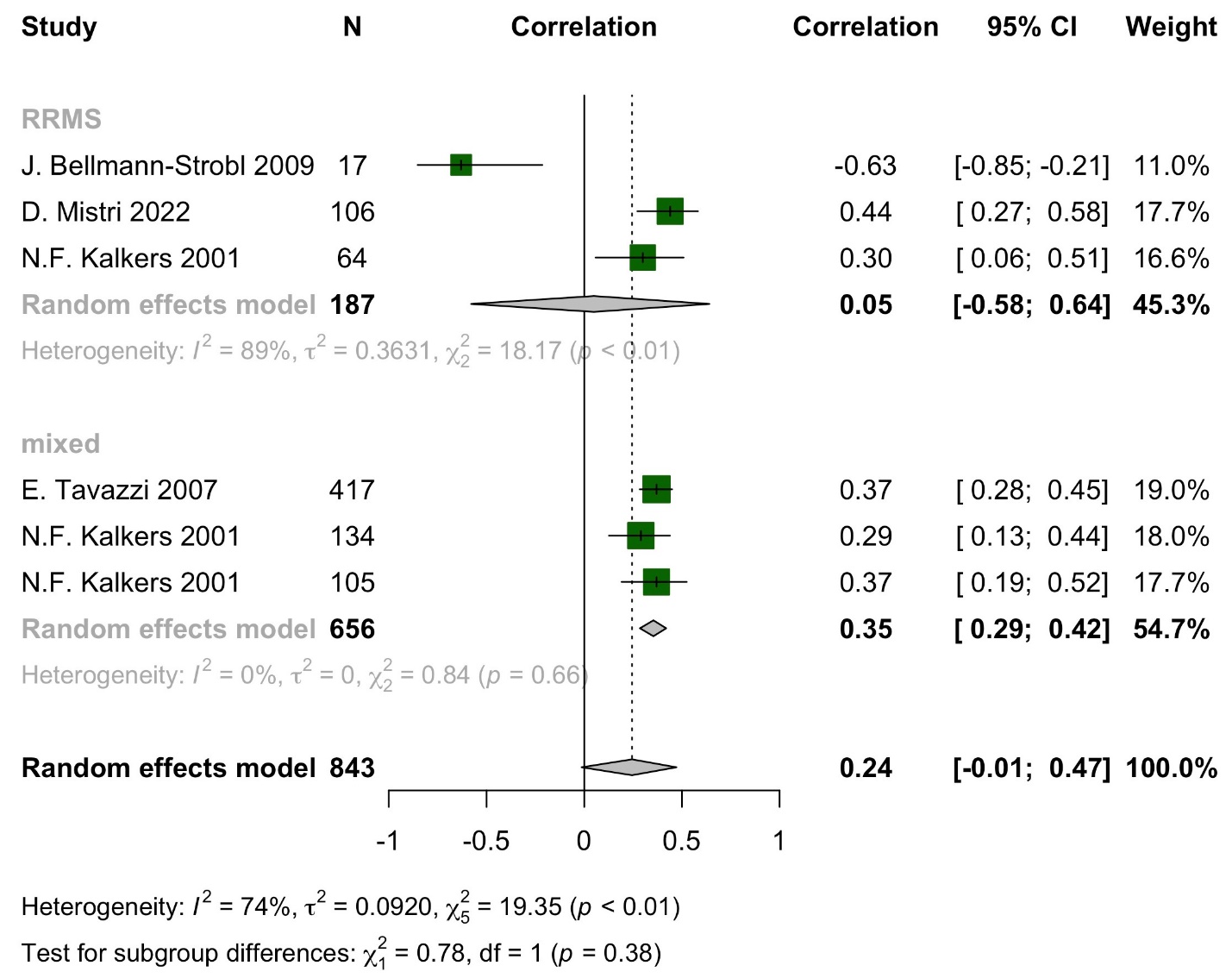


Figure S58. Forest plot of 9HPT and T1 lesion volume correlation in pwMS.


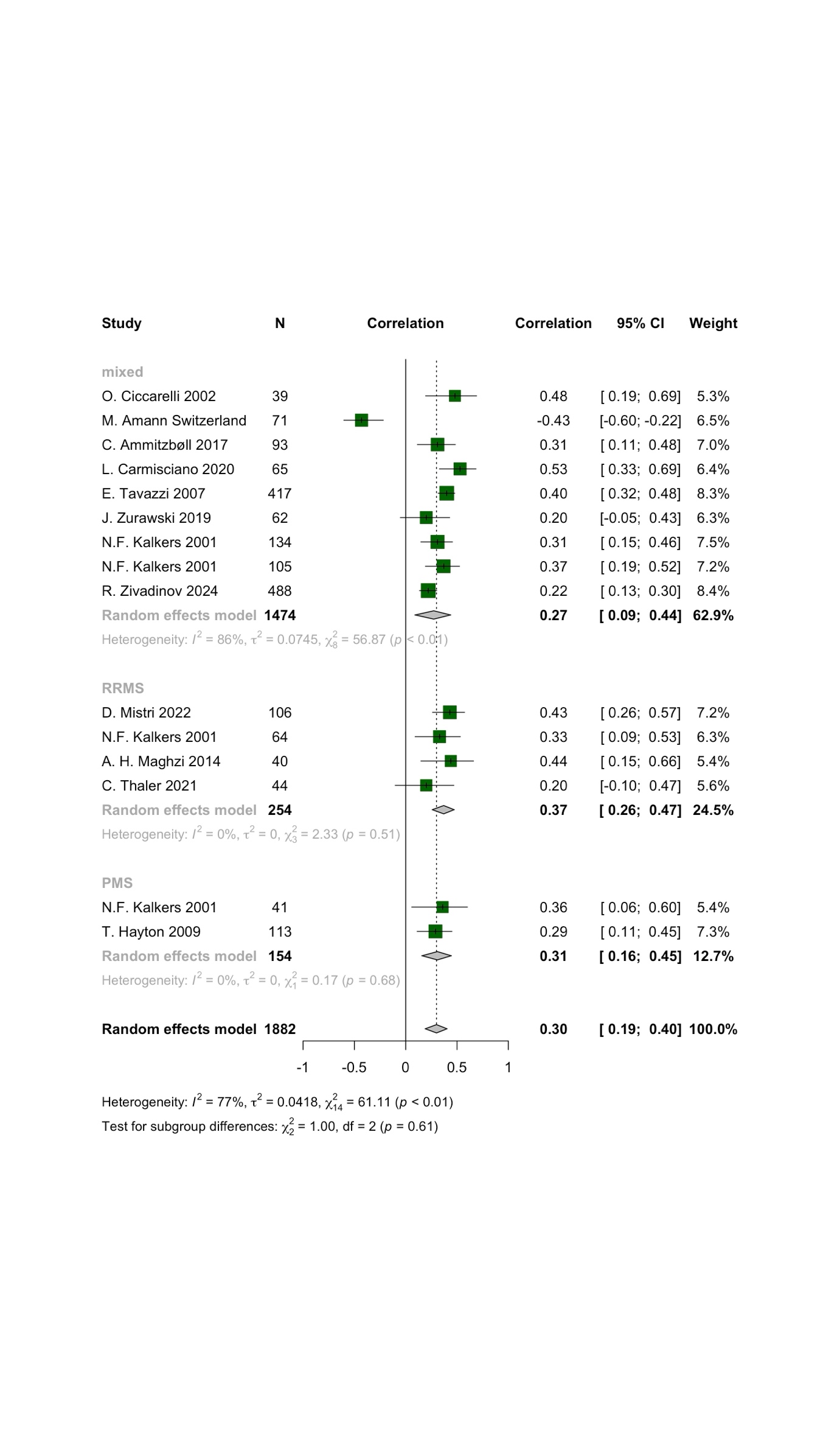


Figure S59. Forest plot of 9HPT and T2 lesion volume correlation in pwMS.


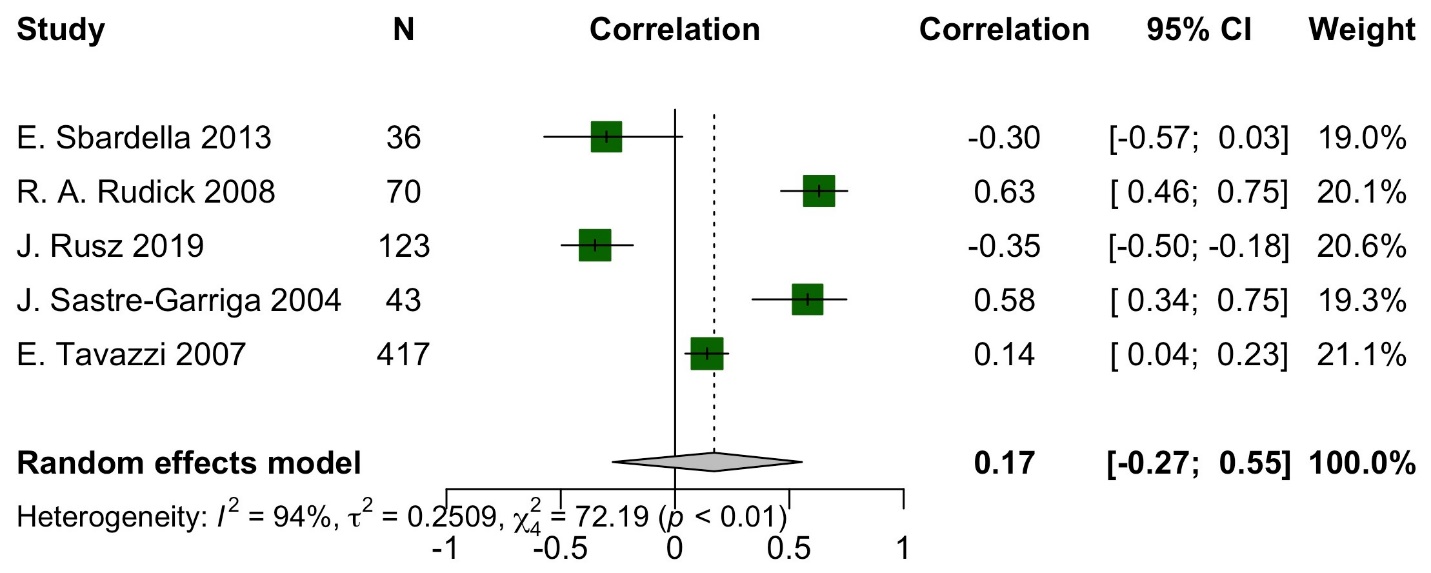


Figure S60. Forest plot of 9HPT and white matter fraction correlation in pwMS.


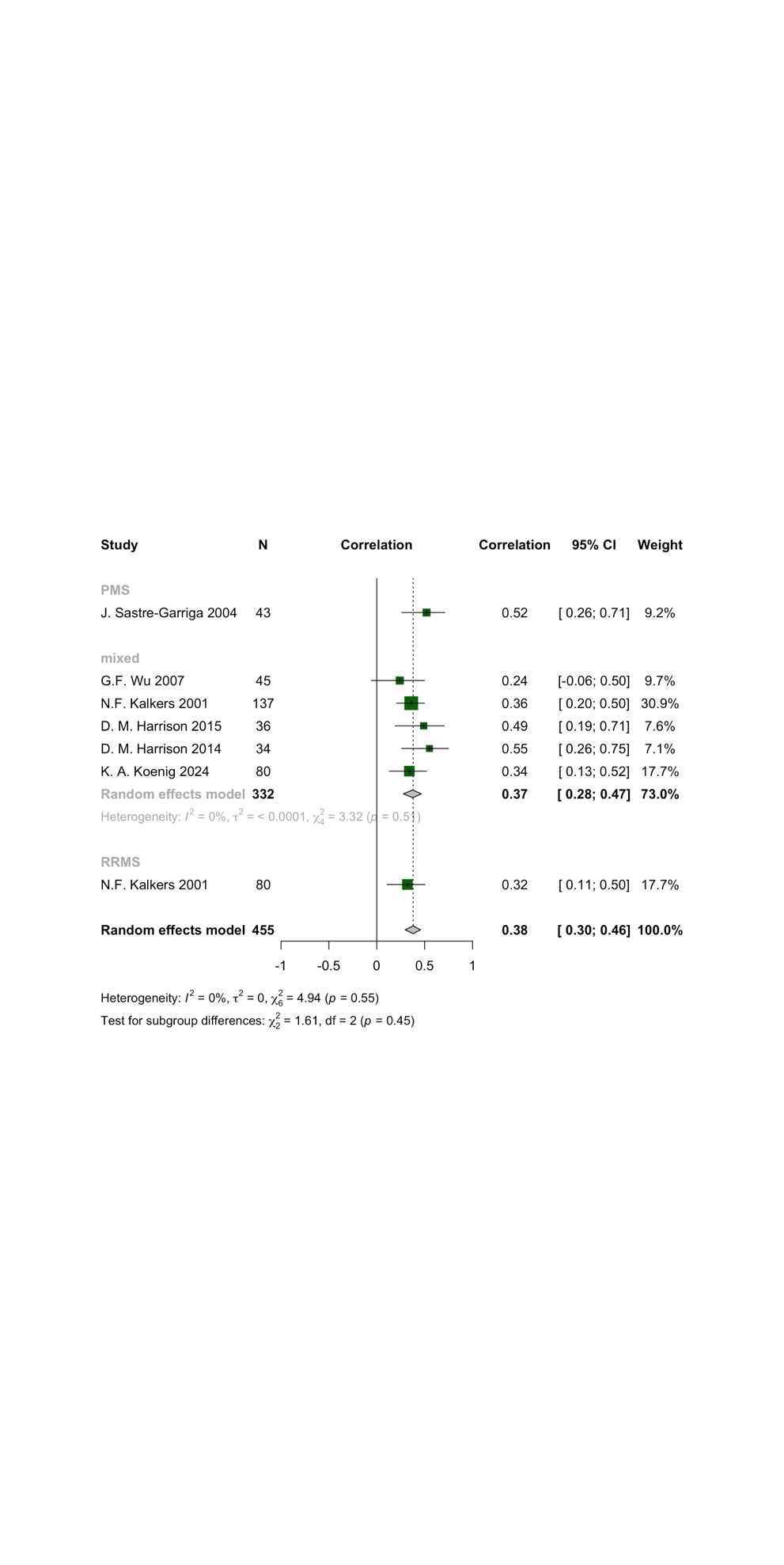


Figure S61. Forest plot of MSFC and BPF correlation in pwMS.


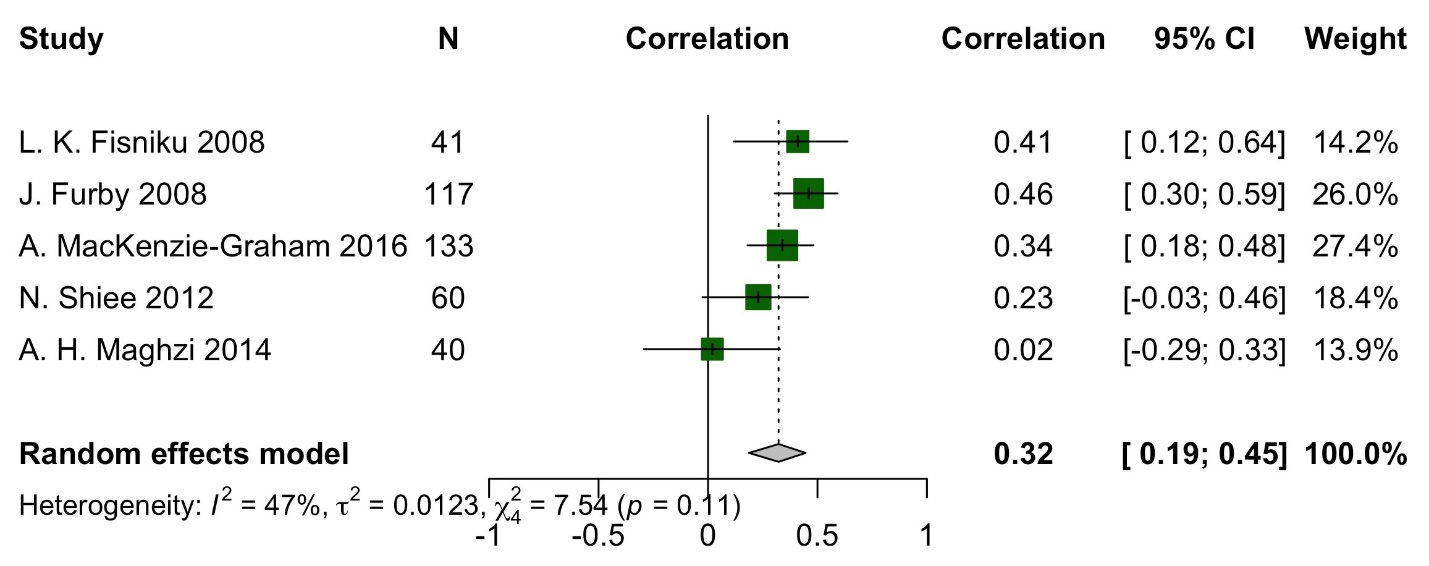


Figure S62. Forest plot of MSFC and normalized grey matter volume correlation in pwMS.


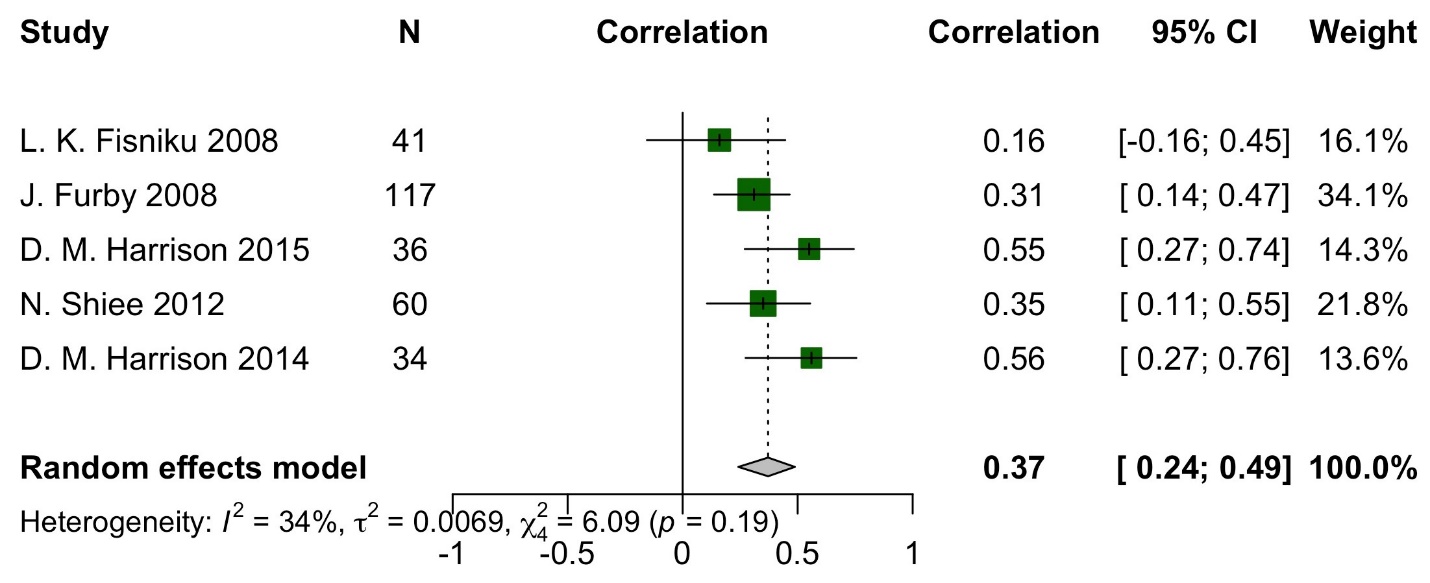


Figure S63. Forest plot of MSFC and normalized white matter volume correlation in pwMS.


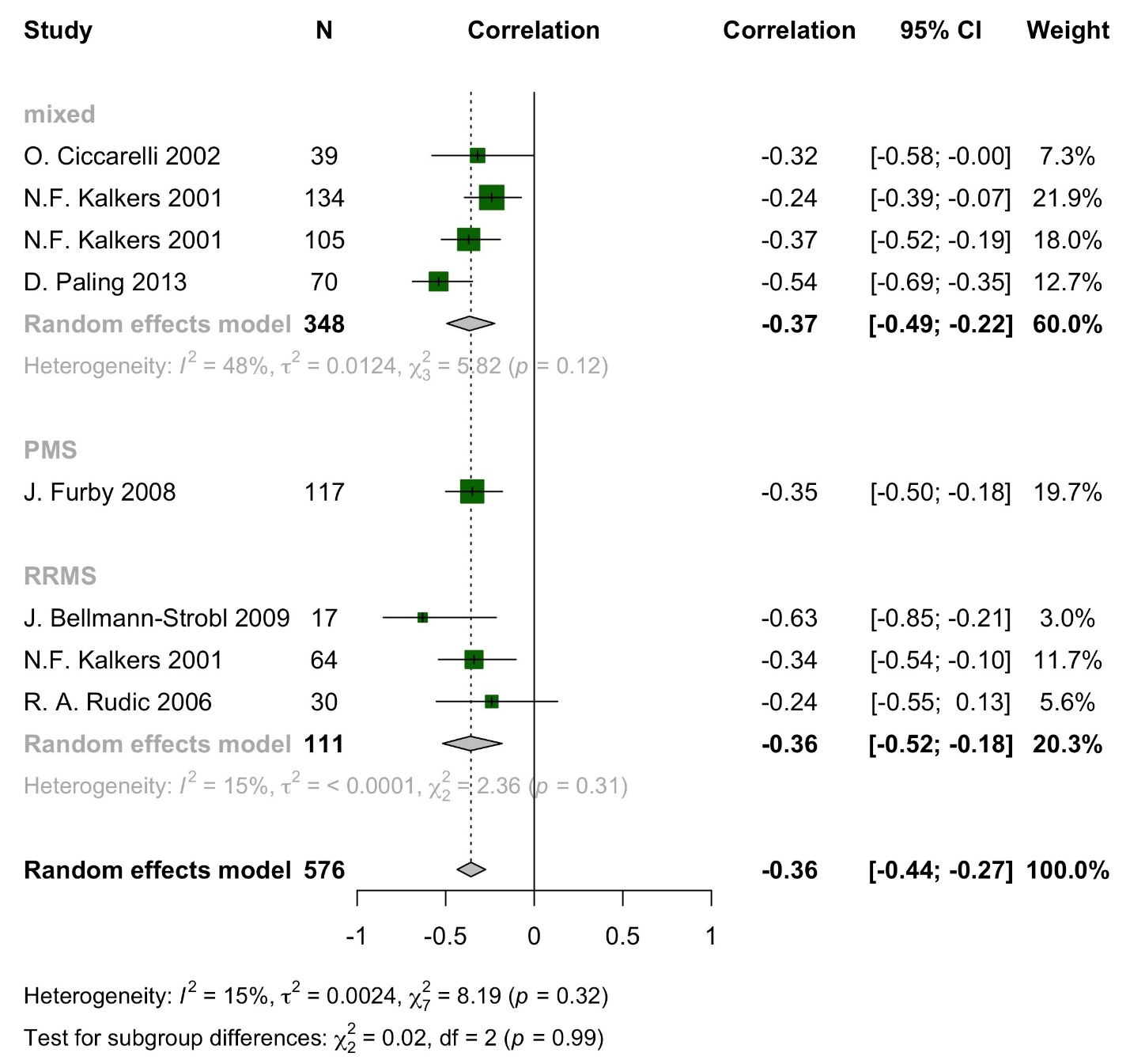


Figure S64. Forest plot of MSFC and T1 lesion volume correlation in pwMS.


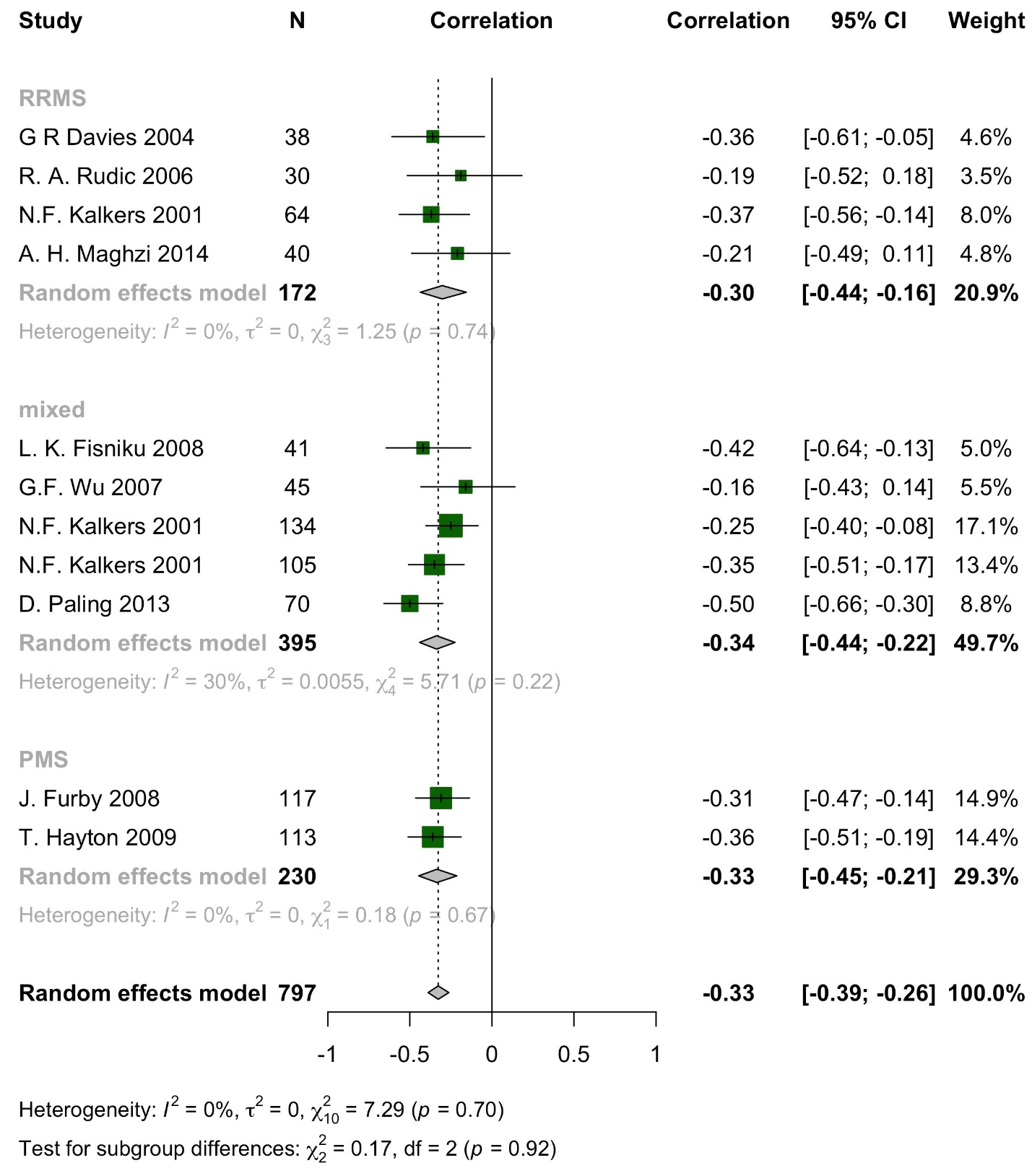


Figure S65. Forest plot of MSFC and T2 lesion volume correlation in pwMS.
