# Supplementary material for: Neuroimaging markers and disability scales in multiple sclerosis: A systematic review and meta-analysis
Source: PLoS One. 2024 Dec 5;19(12):e0312421. doi: 10.1371/journal.pone.0312421 (PMC11620670; doi:10.1371/journal.pone.0312421)
Supplement: S5 File — (DOCX) [file pone.0312421.s006.docx]

Supplementary 5. Sensitivity analyses of disabilitiy and MRI measurements in pwMS.


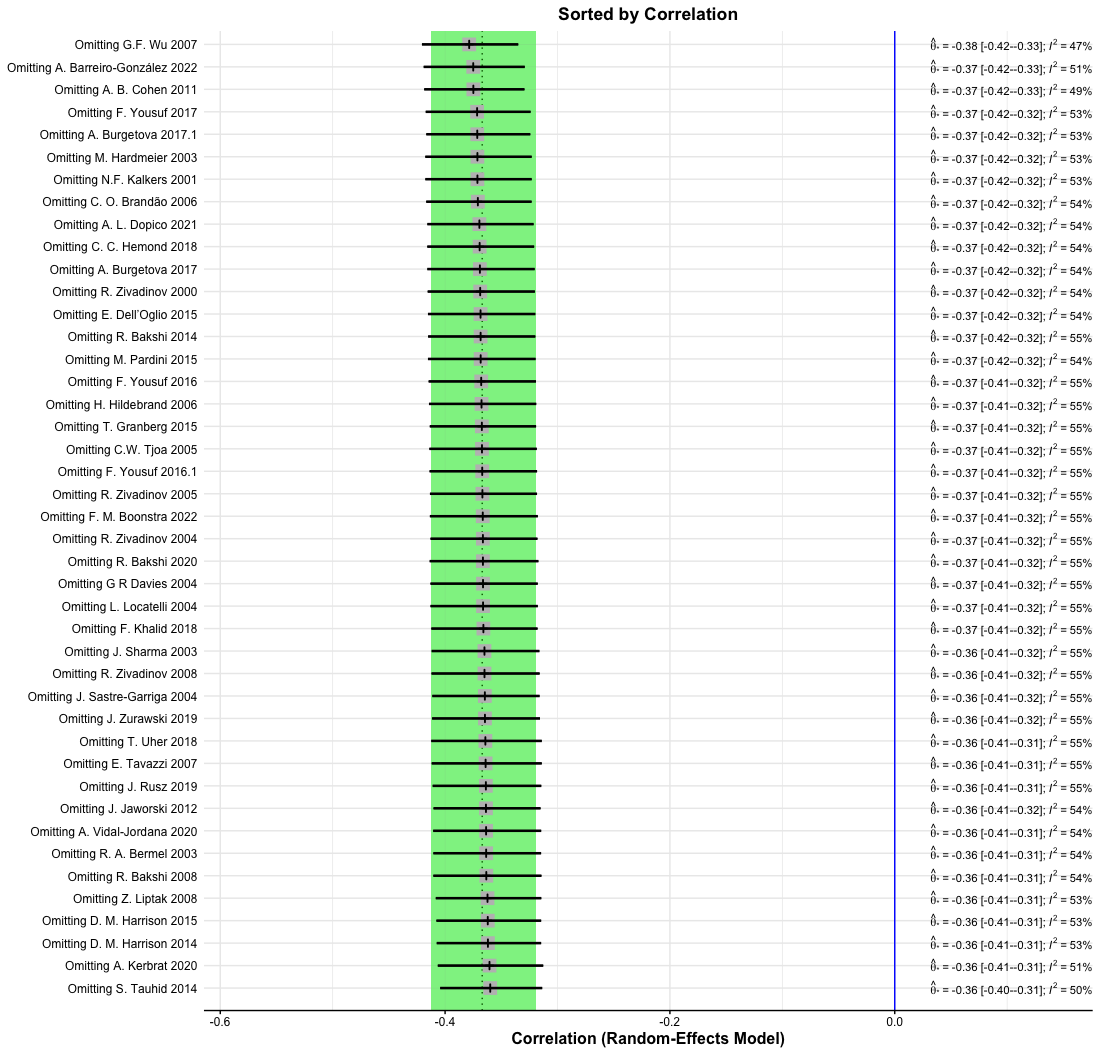


Figure S1. Sensitivity analysis of EDSS and BPF correlation in pwMS.


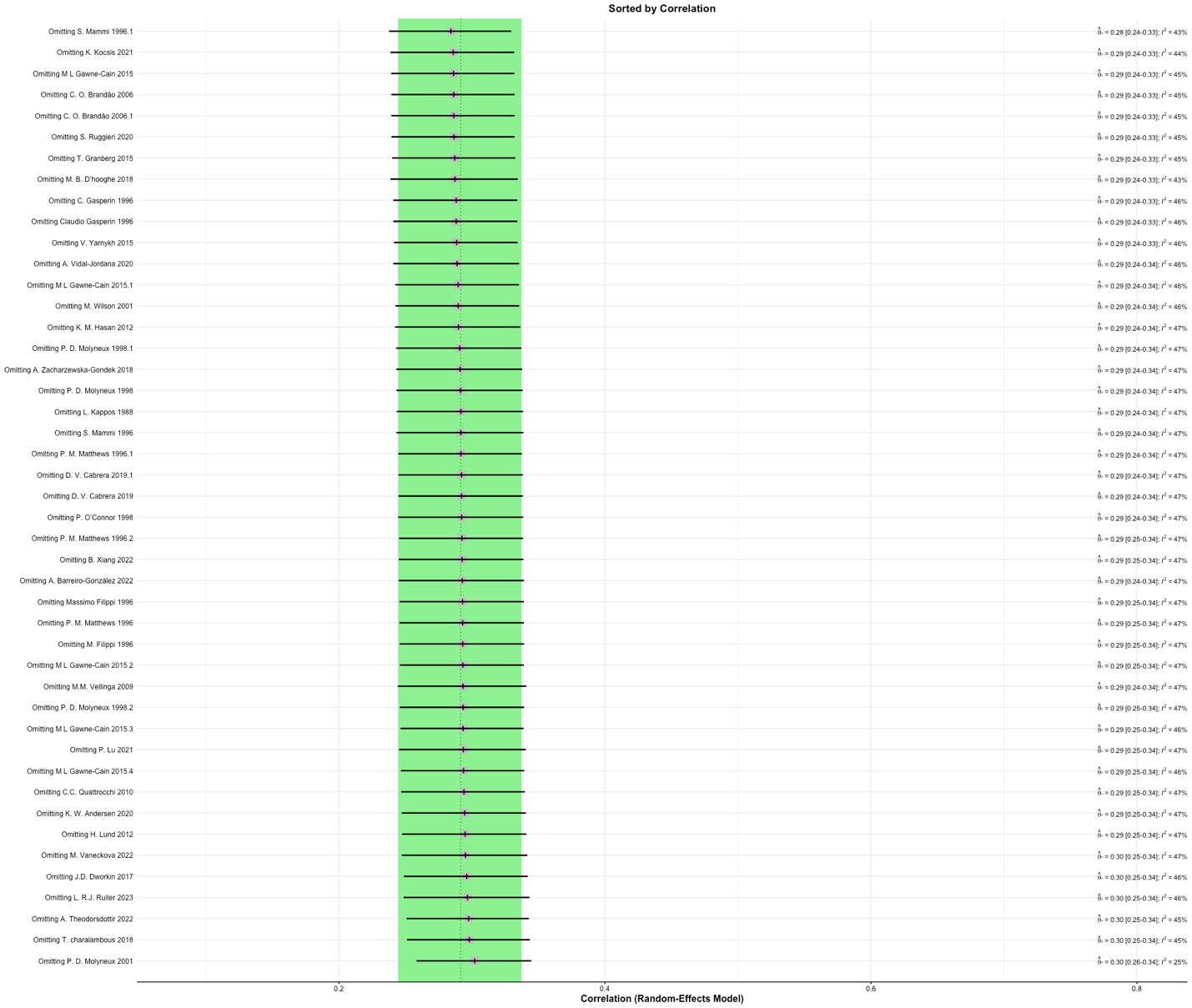


Figure S2. Sensitivity analysis of EDSS and brain lesion volume correlation in pwMS.


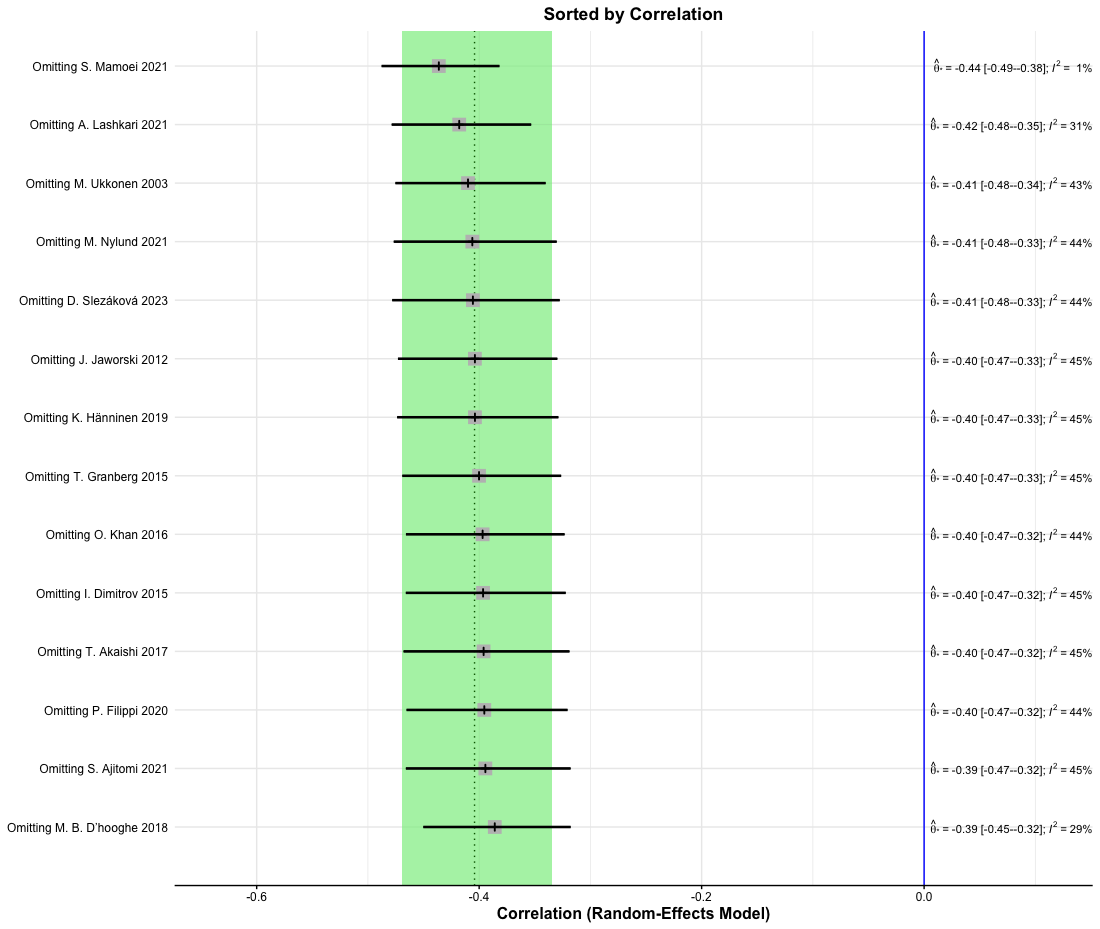


Figure S3. Forest plot of EDSS and brain volume correlation in pwMS.


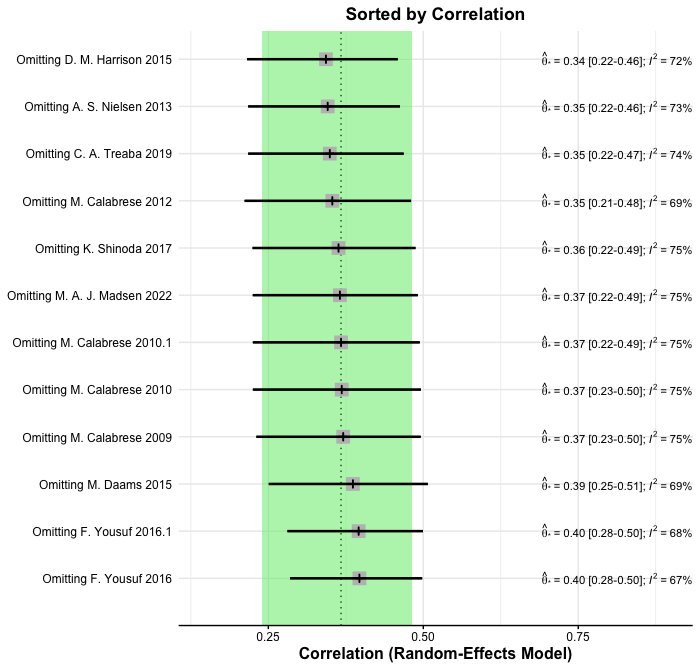


Figure S4. Sensitivity analysis of EDSS and cortical lesion count correlation in pwMS.


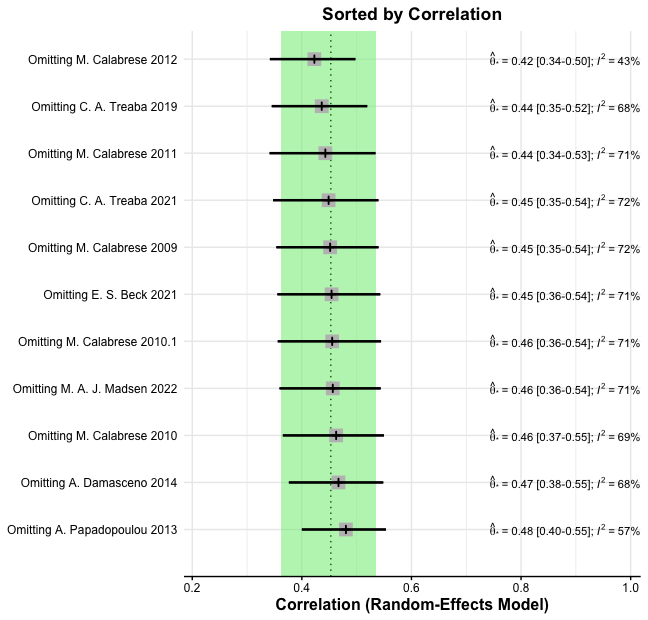


Figure S5. Sensitivity analysis of EDSS and cortical lesion volume correlation in pwMS.


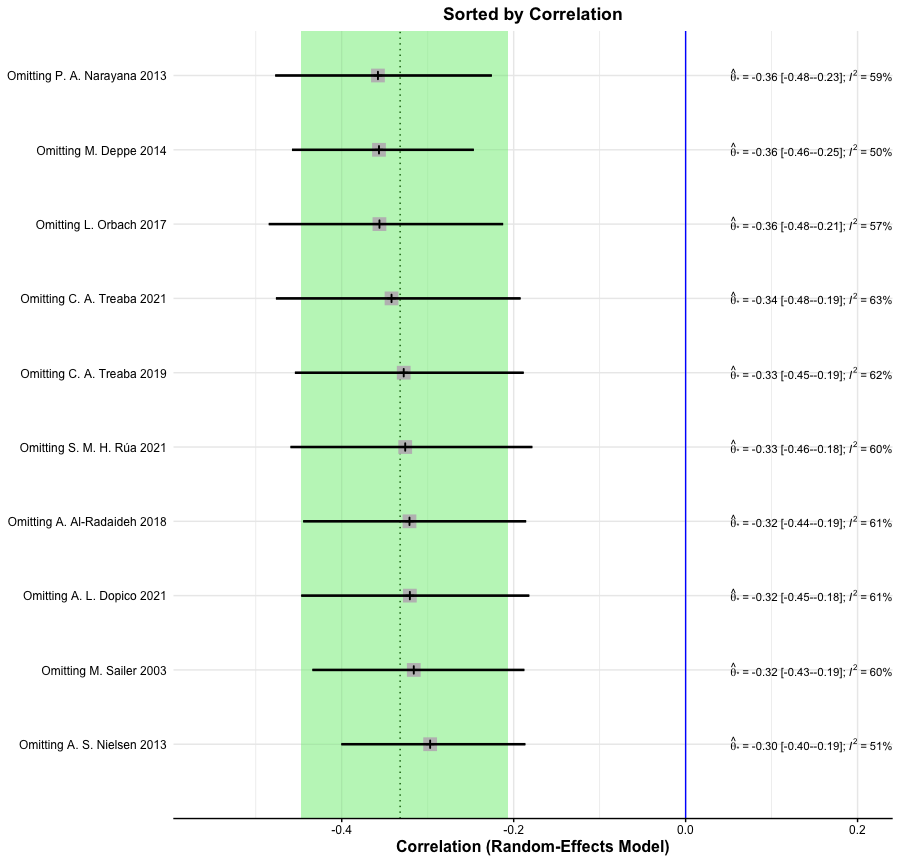


Figure S6. Sensitivity analysis of EDSS and cortical thickness correlation in pwMS.


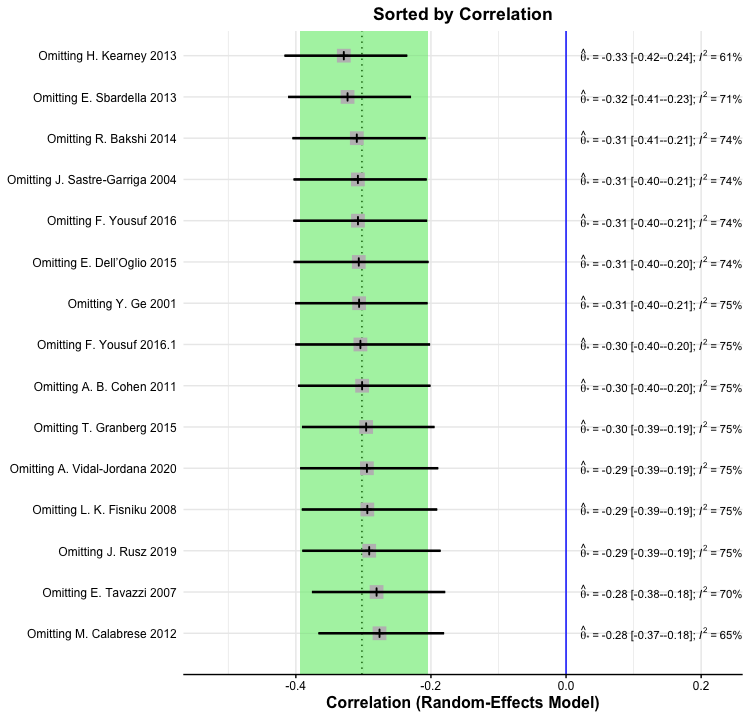


Figure S7. Sensitivity analysis of EDSS and grey matter fraction correlation in pwMS.


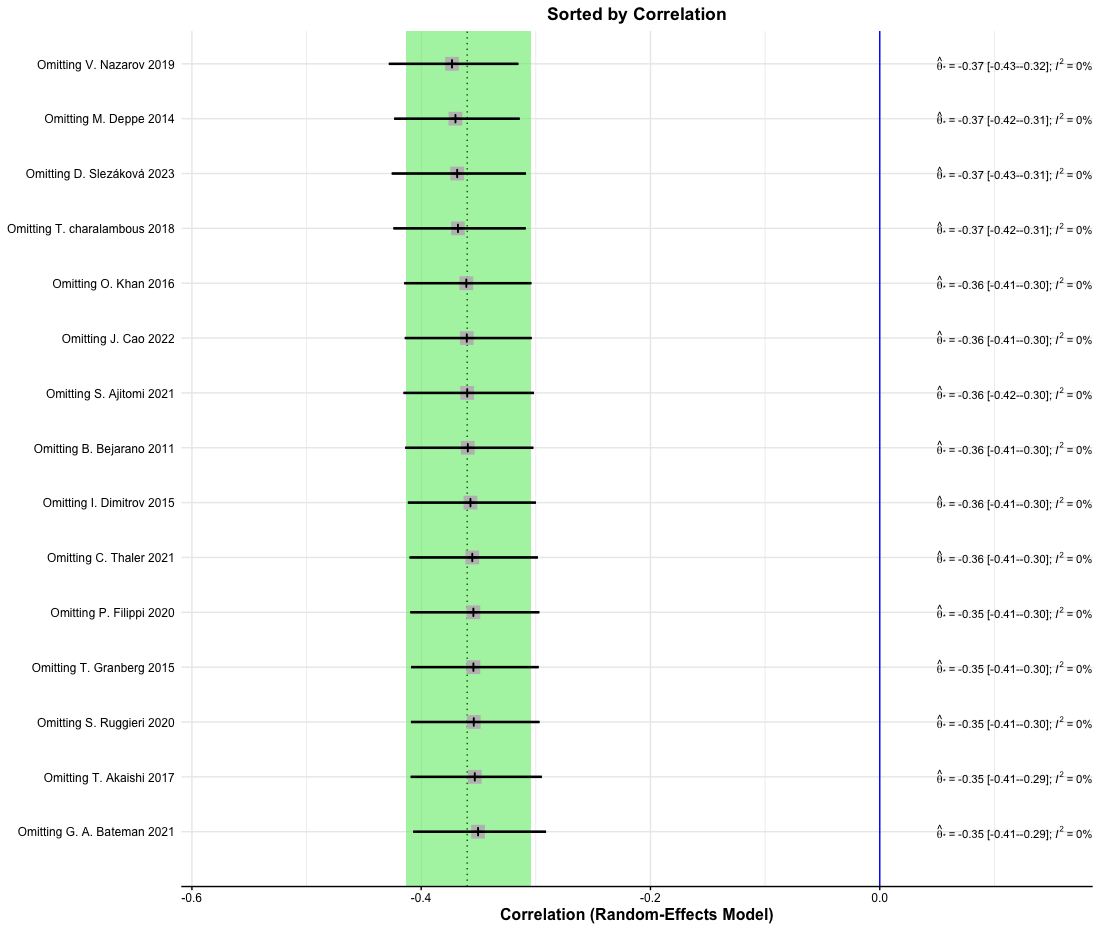


Figure S8. Sensitivity analysis of EDSS and grey matter volume correlation in pwMS.


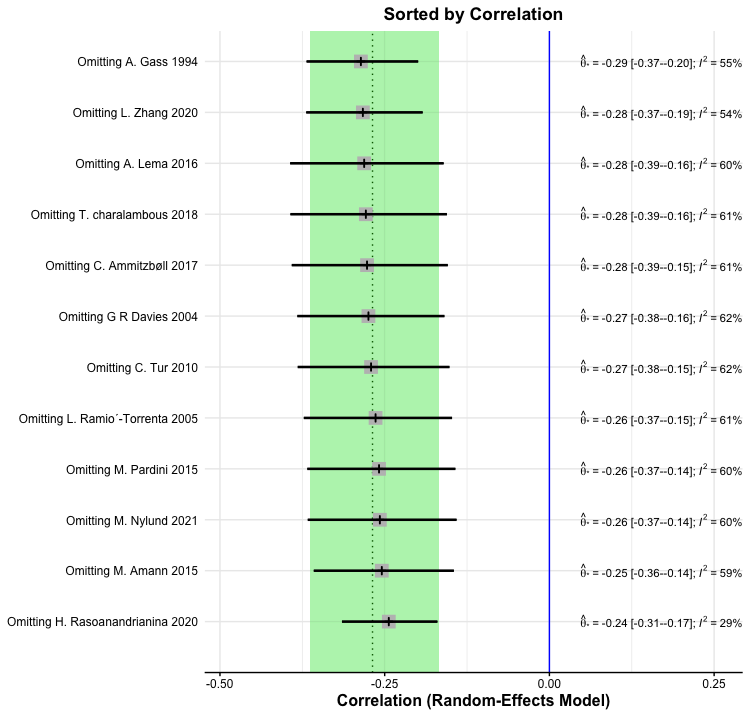


Figure S9. Sensitivity analysis of EDSS and normal-appearing white matter MTR correlation in pwMS.


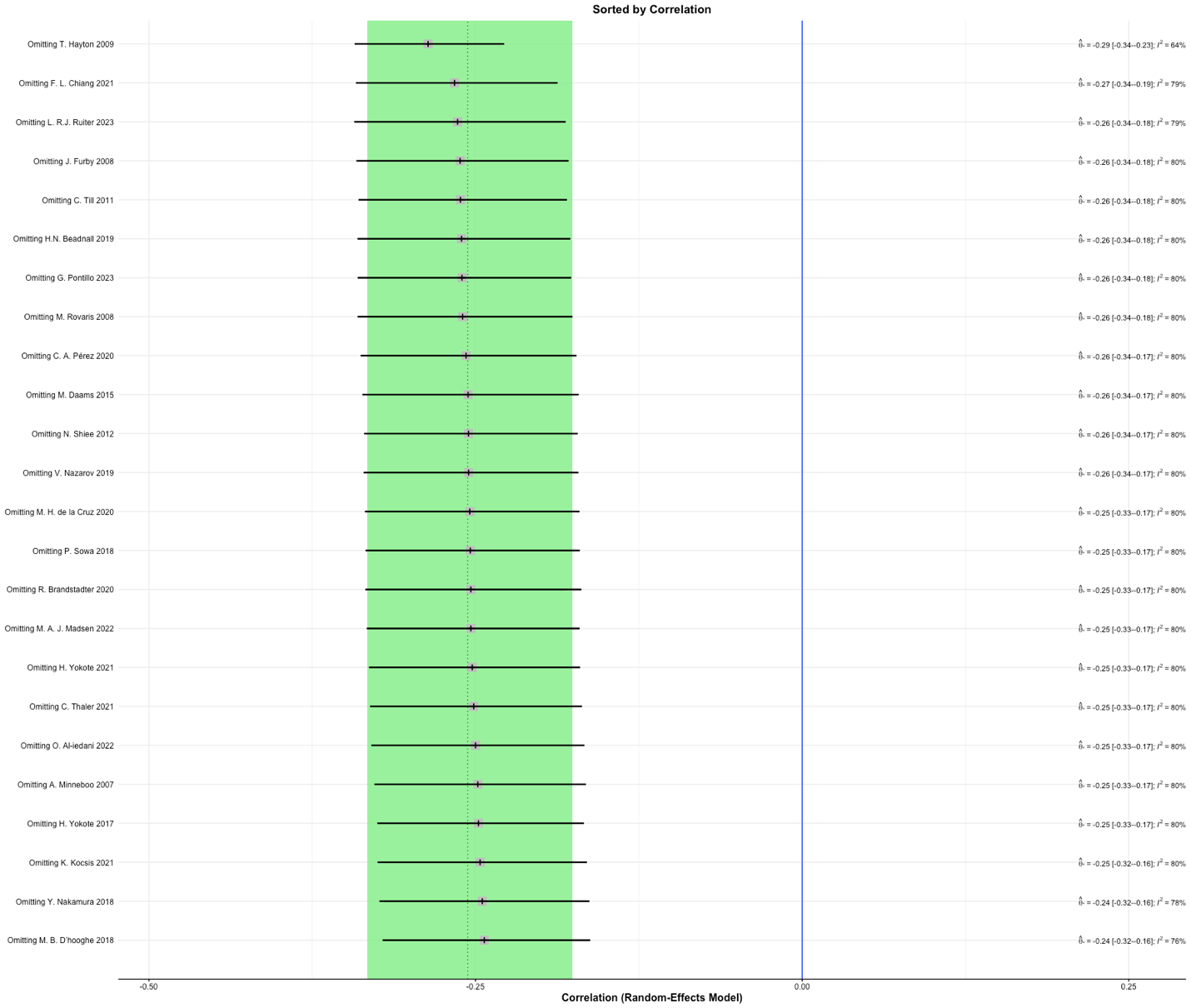


Figure S10. Sensitivity analysis of EDSS and normalized brain volume correlation in pwMS.


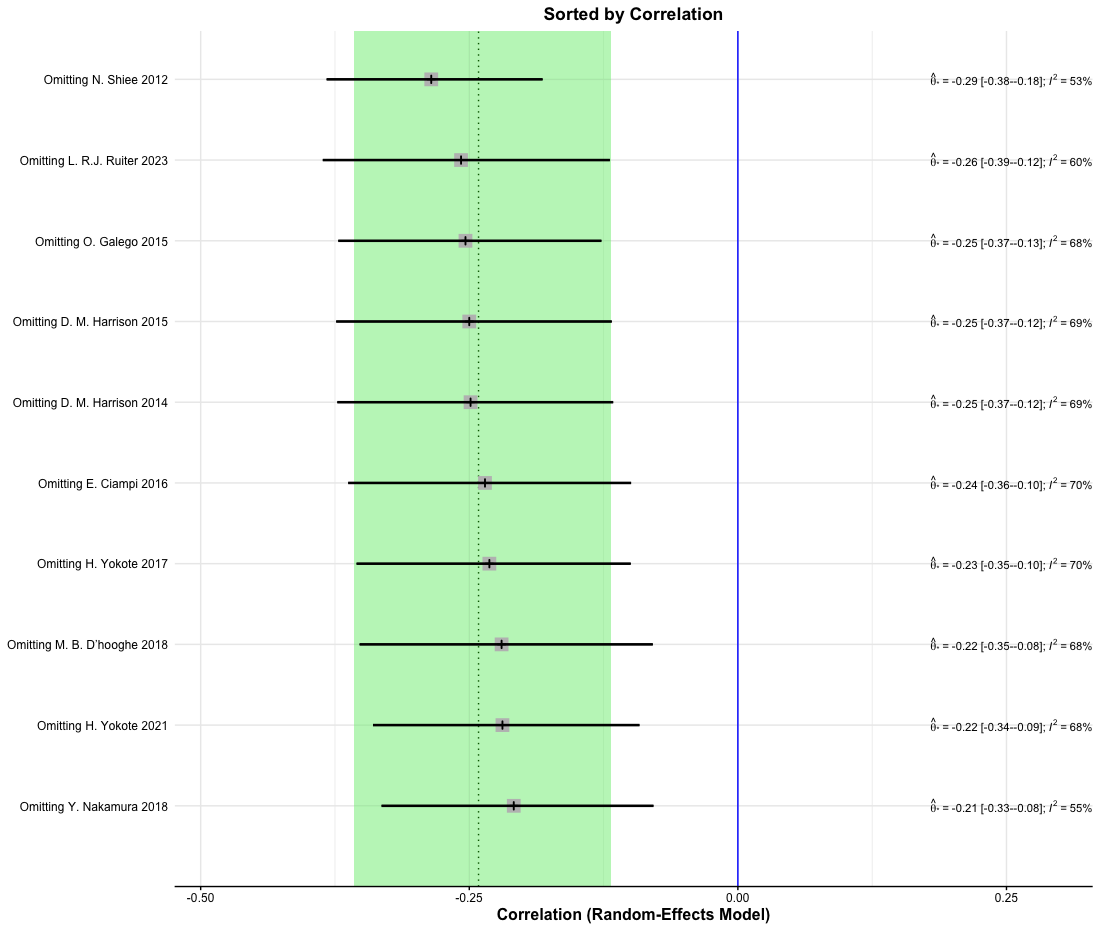


Figure S11. Sensitivity analysis of EDSS and normalized cortical gray matter volume correlation in pwMS.


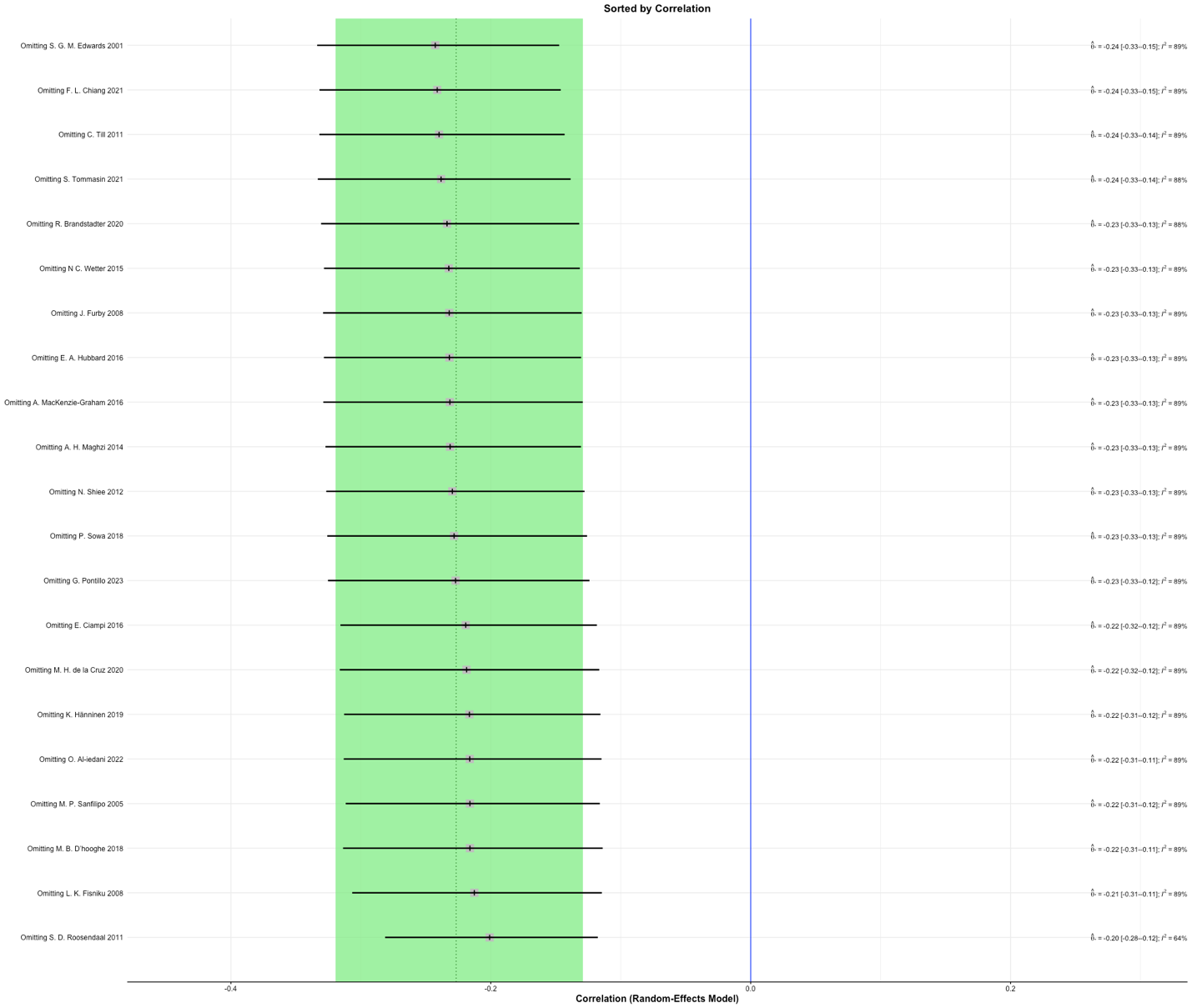


Figure S12. Sensitivity analysis of EDSS and normalized grey matter volume correlation in pwMS.


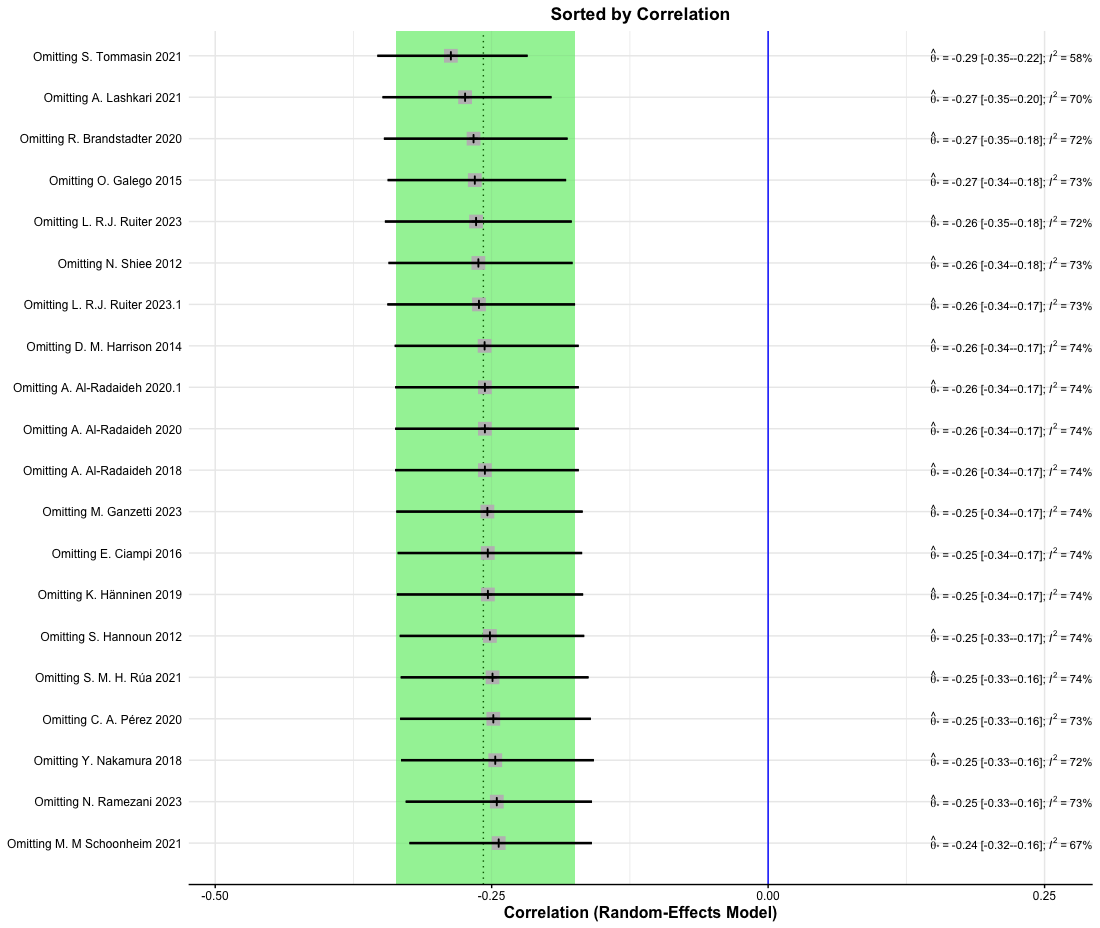


Figure S13. Sensitivity analysis of EDSS and normalized thalamus volume correlation in pwMS.


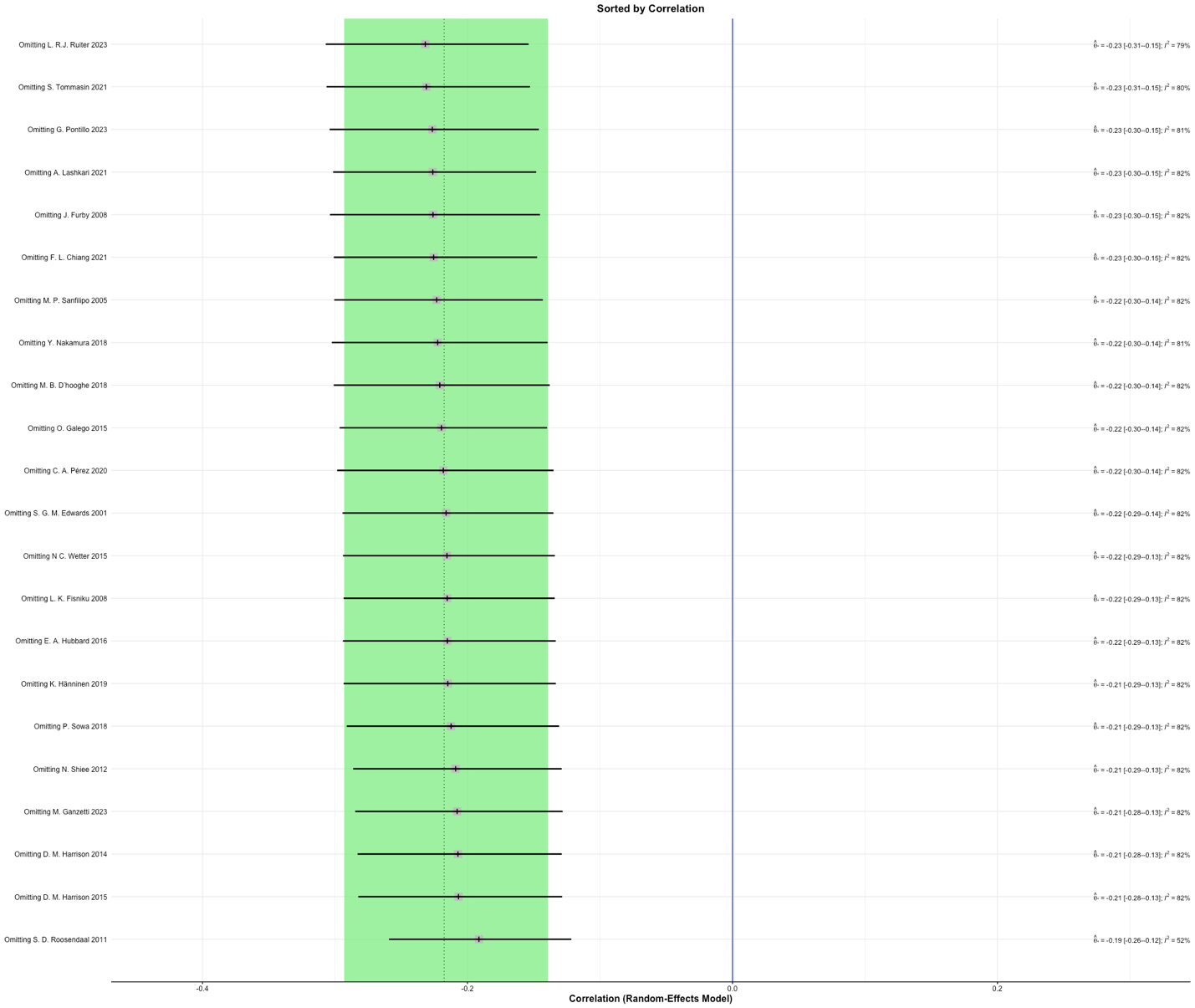


Figure S14. Sensitivity analysis of EDSS and normalized white matter volume correlation in pwMS.


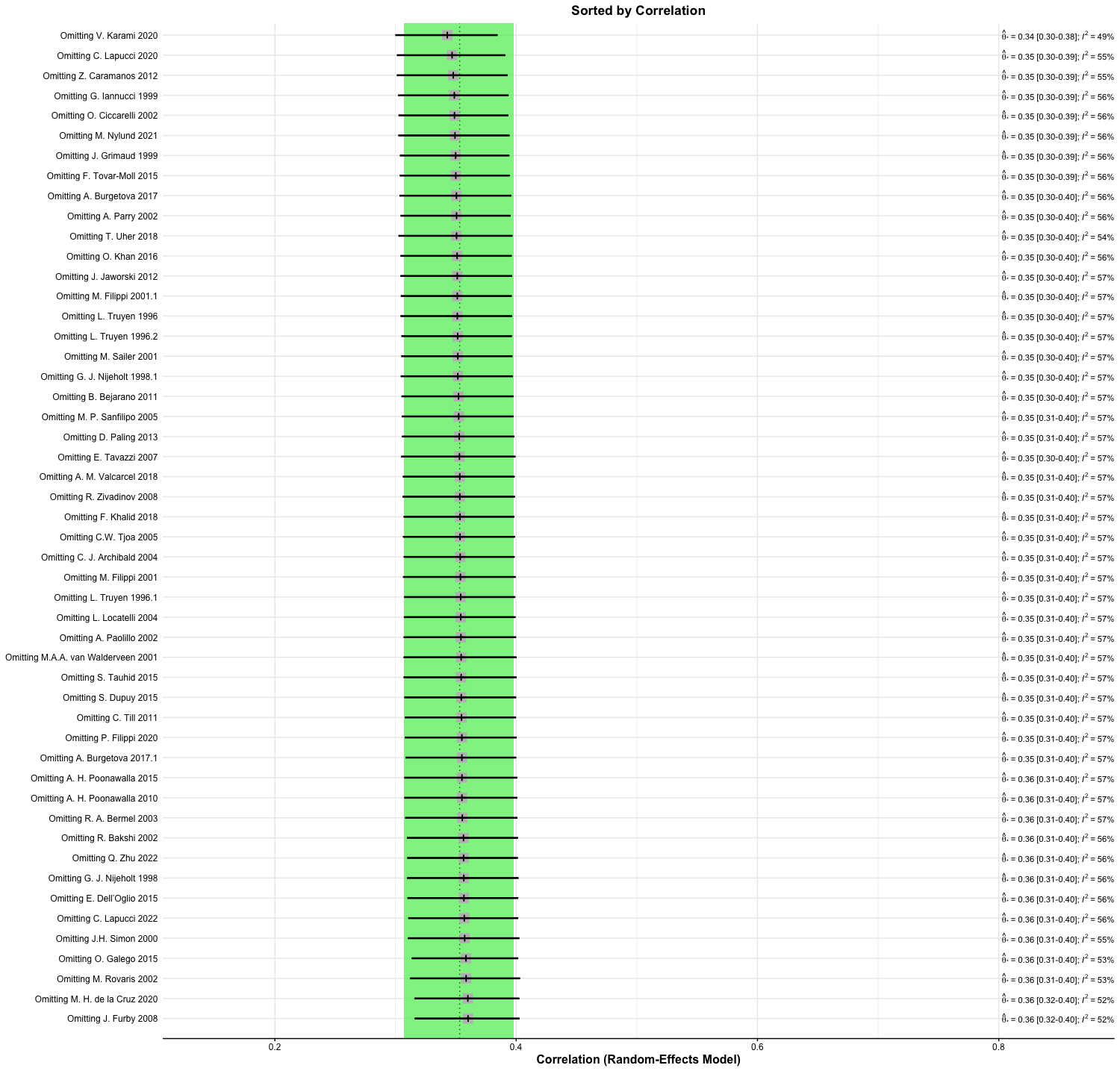


Figure S15. Sensitivity analysis of EDSS and T1 lesion volume correlation in pwMS.


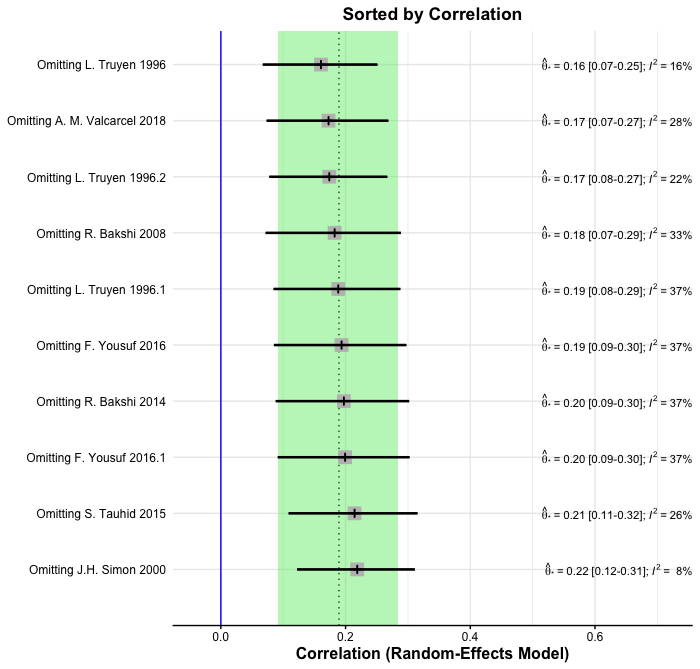


Figure S16. Sensitivity analysis of EDSS and T1LV/T2LV correlation in pwMS.


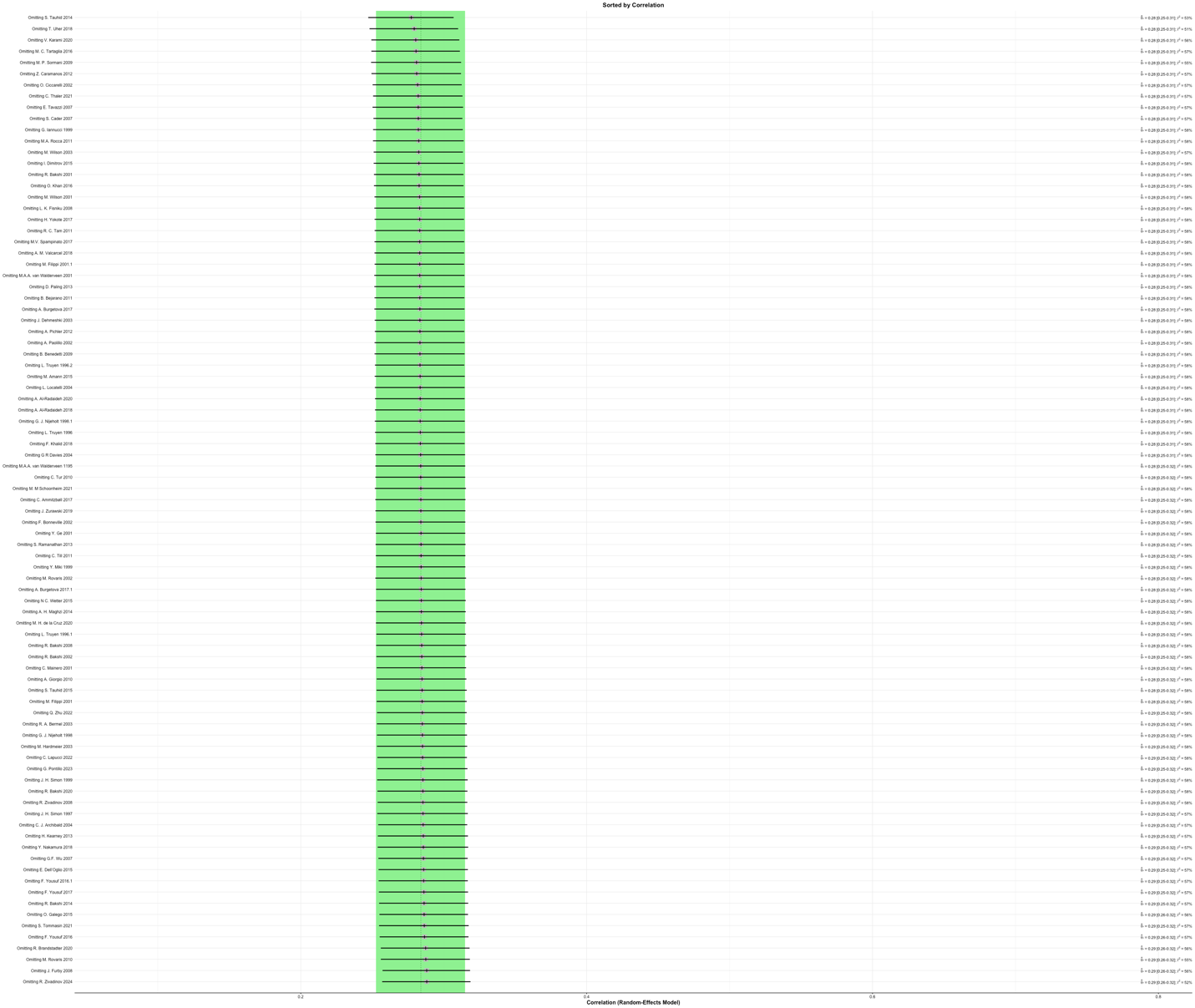


Figure S17. Sensitivity analysis of EDSS and T2 lesion volume correlation in pwMS.


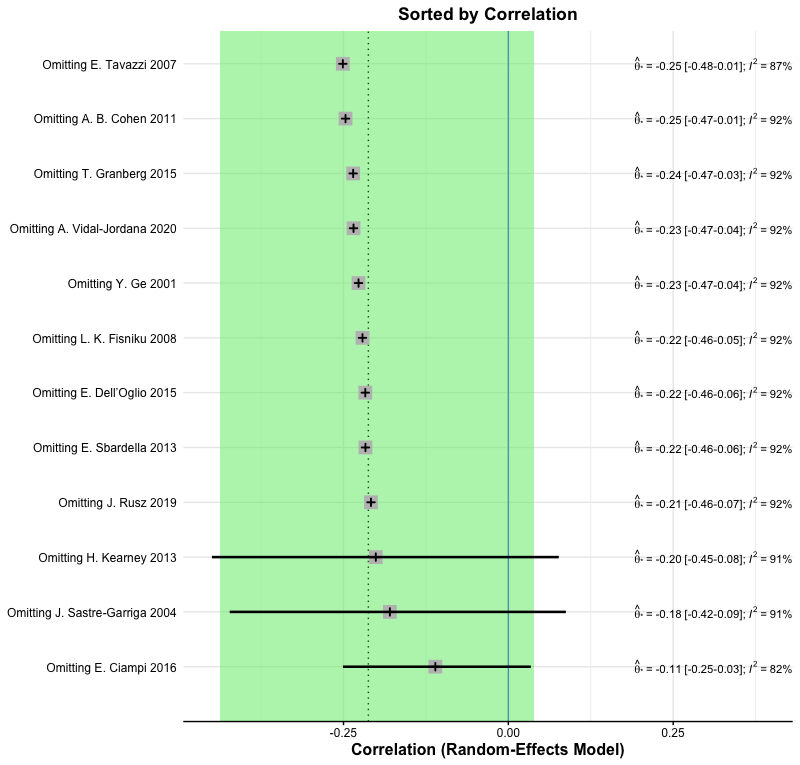


Figure S18. Sensitivity analysis of EDSS and white matter fraction correlation in pwMS.


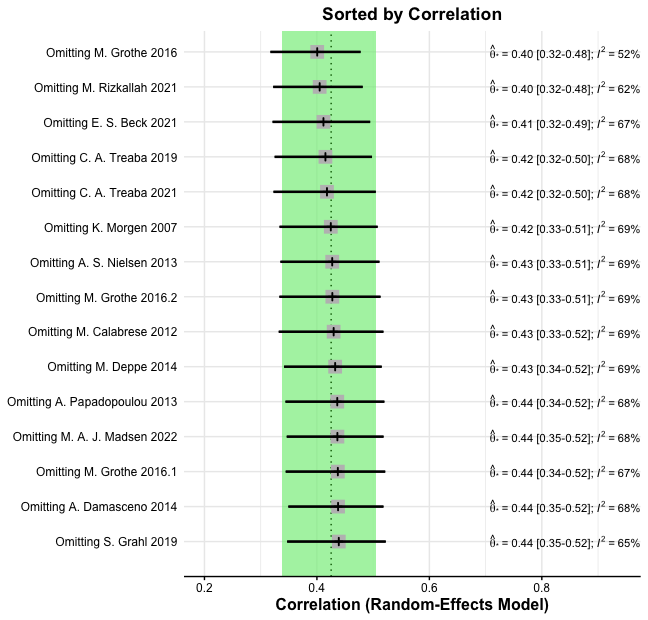


Figure S19. Sensitivity analysis of EDSS and white matter lesion volume correlation in pwMS.


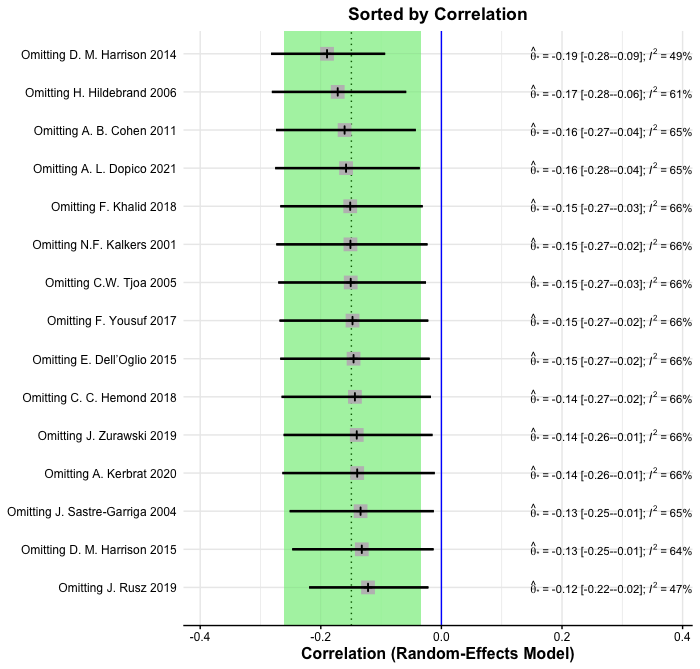


Figure S20. Sensitivity analysis of T25FW and BPF correlation in pwMS.


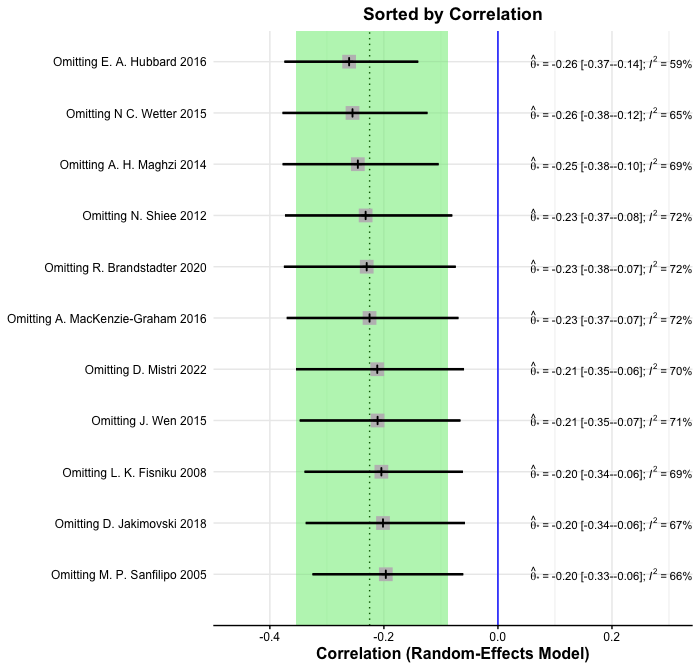


Figure S21. Sensitivity analysis of T25FW and normalized grey matter volume correlation in pwMS.


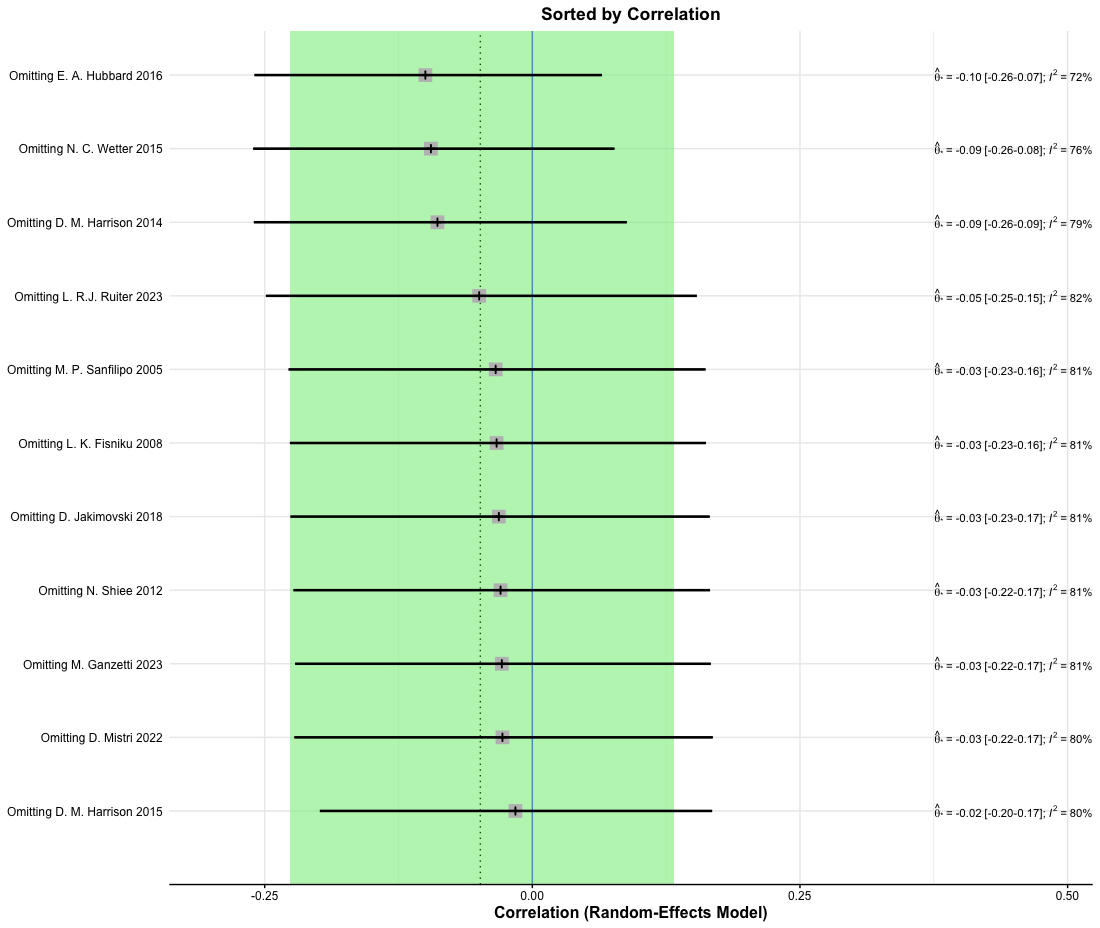


Figure S22. Sensitivity analysis of T25FW and normalized white matter volume correlation in pwMS.


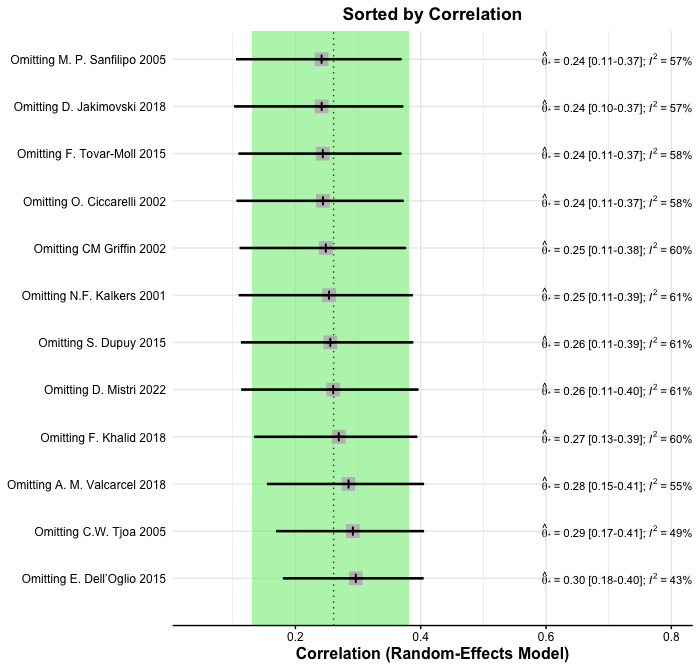


Figure S23. Sensitivity analysis of T25FW and T1 lesion volume correlation in pwMS.


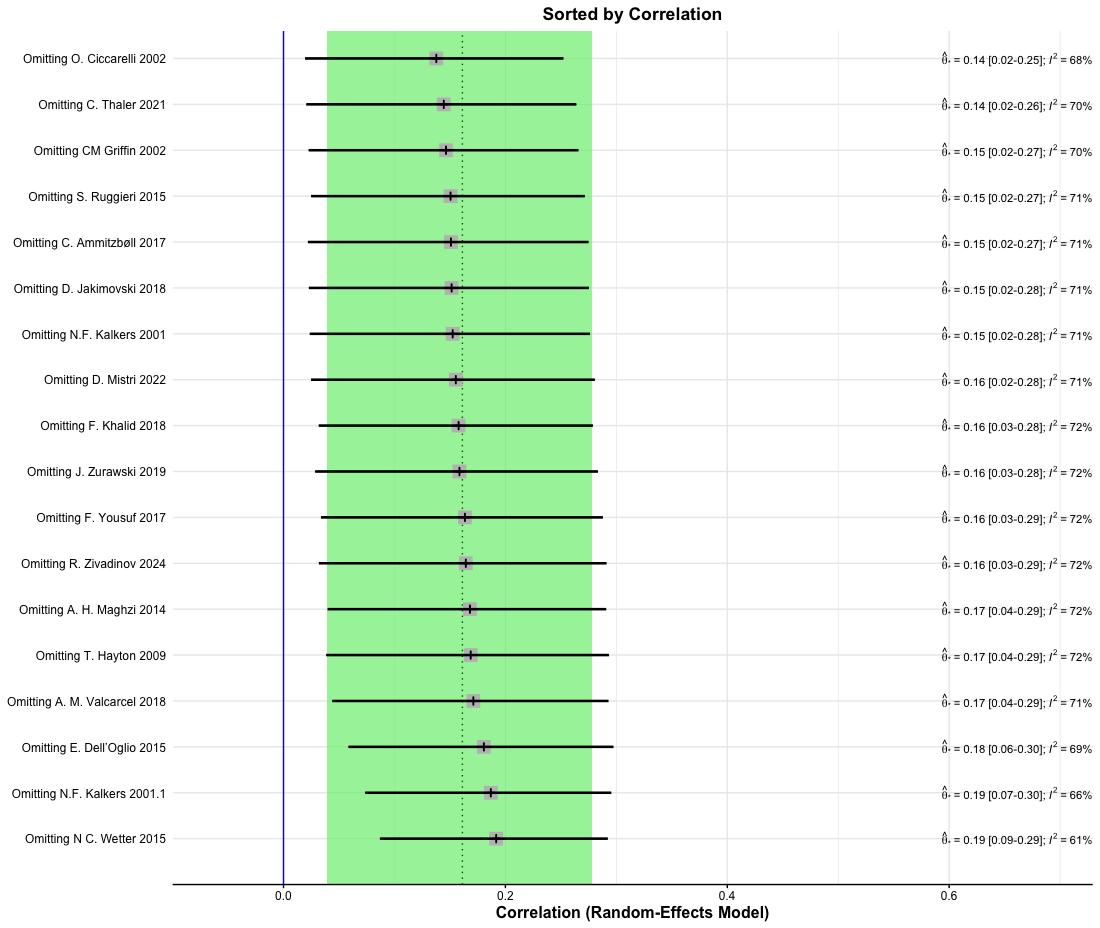


Figure S24. Sensitivity analysis of T25FW and T2 lesion volume correlation in pwMS.


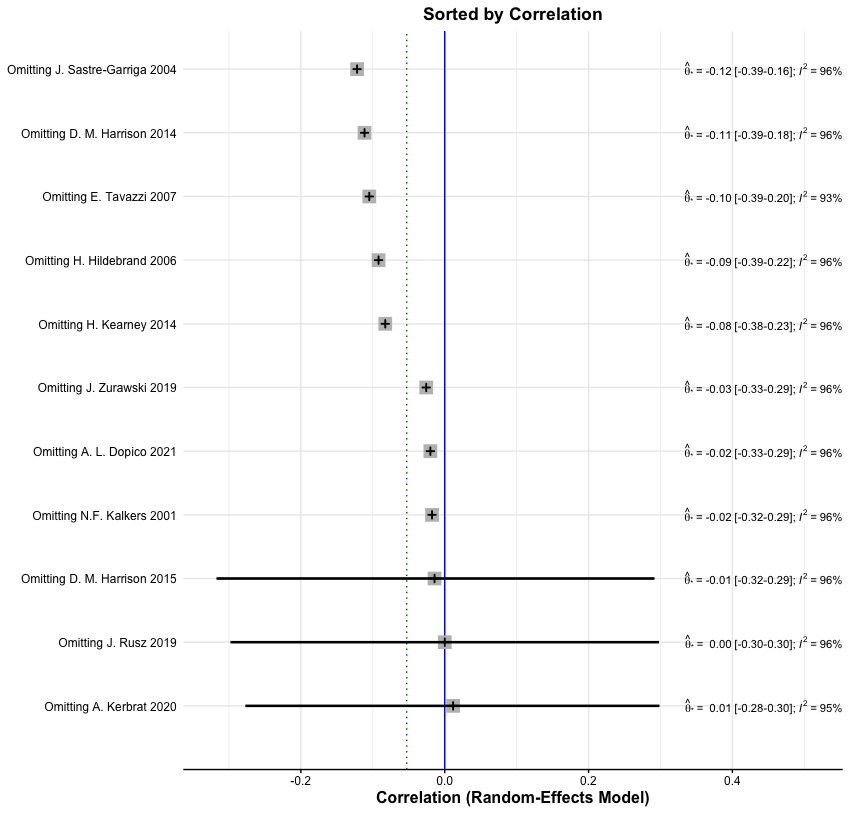


Figure S25. Sensitivity analysis of 9HPT and BPF correlation in pwMS.


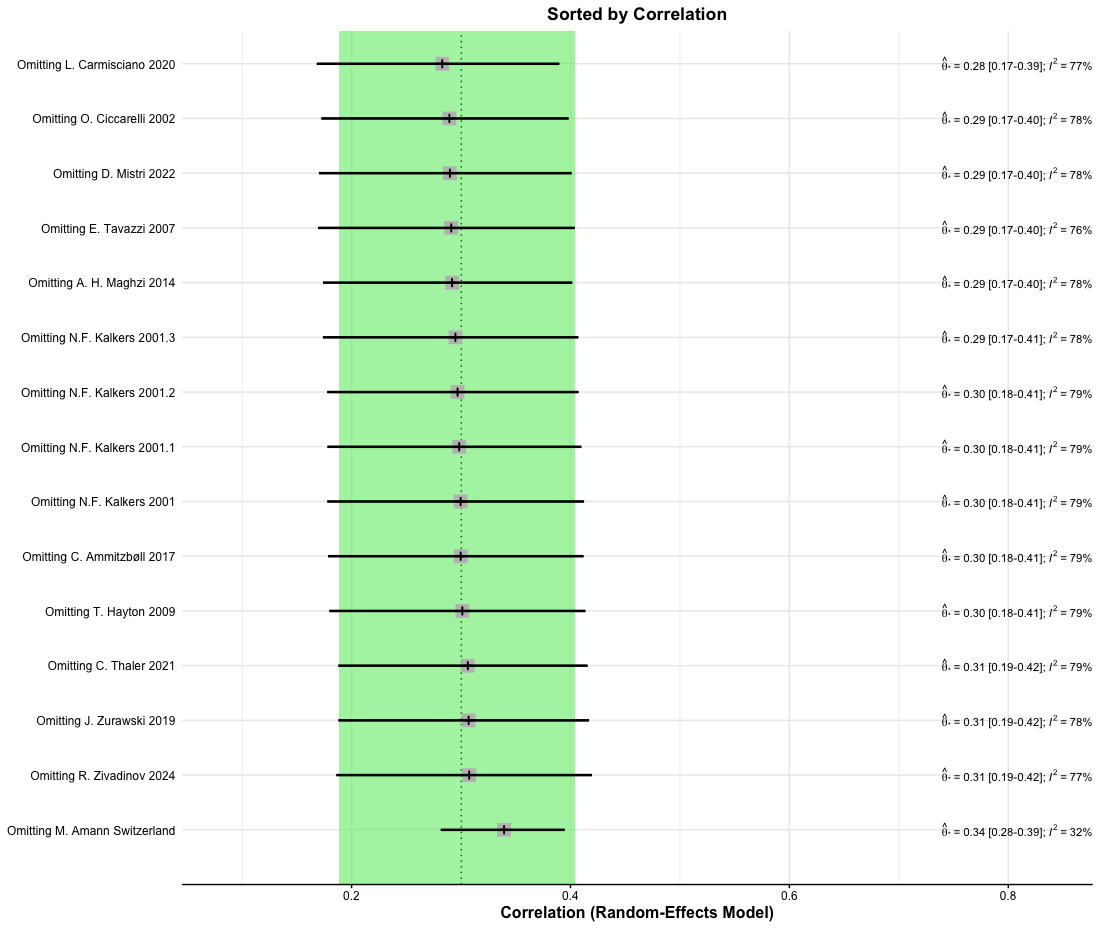


Figure S26. Sensitivity analysis of 9HPT and T2 lesion volume correlation in pwMS.


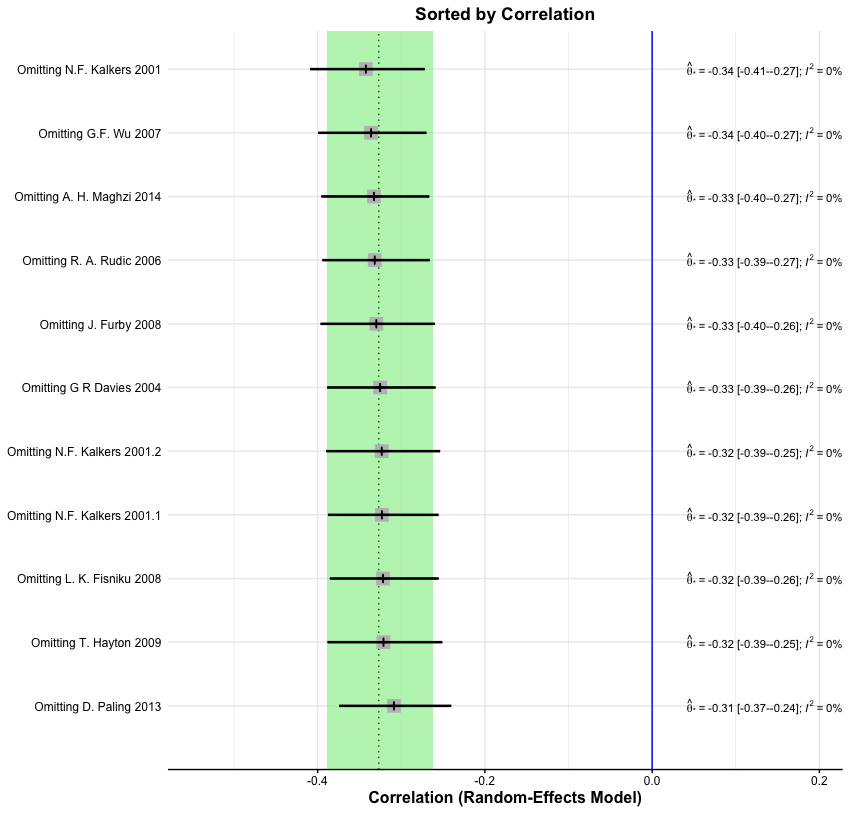


Figure S27. Sensitivity analysis of MSFC and T2 lesion volume correlation in pwMS.
