# Supplementary material for: Neuroimaging markers and disability scales in multiple sclerosis: A systematic review and meta-analysis
Source: PLoS One. 2024 Dec 5;19(12):e0312421. doi: 10.1371/journal.pone.0312421 (PMC11620670; doi:10.1371/journal.pone.0312421)
Supplement: S6 File — (DOCX) [file pone.0312421.s007.docx]

Supplementary 6. Results of publication bias within different meta-analysis.

| **Correlation coefficient** | | **Egger's test** | | | | **Begg's test** | |
| --- | --- | --- | --- | --- | --- | --- | --- |
| **EDSS vs.** |  | Intercept | 95% CI | t-value | *p*-value | Z | *p*-value |
|  | BPF | 0.89 | 0.04, 1.74 | 2.05 | **0.047** | 0.87 | 0.385 |
|  | Brain lesion volume | 0.69 | -0.08, 1.45 | 1.76 | 0.085 | -1.09 | 0.277 |
|  | Brain volume | 1.62 | 0.14, 3.09 | 2.14 | 0.053 | 0.88 | 0.38 |
|  | Cortical lesion count | -0.02 | -2.8, 2.76 | -0.01 | 0.991 | 1.17 | 0.243 |
|  | Cortical lesion volume | -2.55 | -5.34, 0.24 | -1.79 | 0.107 | -0.70 | 0.484 |
|  | Cortical thickness | -1.63 | -3.77, 0.51 | -1.49 | 0.174 | -1.26 | 0.209 |
|  | GMF | 2.39 | 0.65, 4.14 | 2.69 | **0.019** | 0.45 | 0.656 |
|  | GMV | -0.45 | -2.42, 1.51 | -0.45 | 0.66 | -0.10 | 0.921 |
|  | NAWM MTR | -0.70 | -3.73, 2.34 | -0.45 | 0.662 | -0.28 | 0.783 |
|  | Normalized brain volume | 1.27 | - 1.11, 3.65 | 1.04 | 0.308 | -0.72 | 0.472 |
|  | Normalized cortical GMV | 1.09 | -1.04, 3.21 | 1.01 | 0.344 | 0.45 | 0.655 |
|  | Normalized GM volume | 4.07 | 2.2, 5.93 | 4.26 | **<0.001** | -0.21 | 0.832 |
|  | Normalized thalamus volume | 1.27 | -0.52, 3.06 | 1.39 | 0.181 | 1.63 | 0.103 |
|  | Normalized WMV | 1.53 | -0.24, 3.3 | 1.69 | 0.107 | -1.10 | 0.271 |
|  | T1 lesion volume | -0.06 | - 0.9, 0.78 | -0.13 | 0.894 | 1.73 | 0.083 |
|  | T1LV.T2LV | 2.67 | 1, 4.35 | 3.13 | **0.014** | 2.06 | **0.040** |
|  | T2 lesion volume | -0.12 | - 0.72, 0.49 | -0.37 | 0.711 | 2.51 | **0.012** |
|  | WMF | -3.02 | -6.8, 0.76 | -1.57 | 0.148 | 0.69 | 0.493 |
|  | WM lesion volume | 0.44 | -1.69, 2.58 | 0.41 | 0.692 | 0.84 | 0.399 |
| **MSFC vs.** |  |  |  |  |  |  |  |
|  | T2 lesion volume | 0.27 | - 1.77, 2.32 | 0.26 | 0.800 | 0.08 | 0.938 |
| **NHPT vs.** |  |  |  |  |  |  |  |
|  | BPF | -3.16 | -10.44, 4.11 | -0.85 | 0.416 | 0.78 | 0.435 |
|  | T2 lesion volume | -0.03 | -2.61, 2.56 | -0.02 | 0.984 | 0.45 | 0.656 |
| **T25FW vs.** |  |  |  |  |  |  |  |
|  | BPF | 1.81 | -0.47, 4.09 | 1.55 | 0.144 | 1.74 | 0.083 |
|  | Normalized GMV | -0.47 | -4.3, 3.36 | 0.24 | 0.815 | -0.31 | 0.755 |
|  | Normalized WMV | 0.32 | -4.28, 4.92 | 0.14 | 0.895 | 0.08 | 0.938 |
|  | T1 lesion volume | 0.33 | -3.31, 3.97 | 0.18 | 0.862 | 0.34 | 0.731 |
|  | T2 lesion volume | 0.34 | - 1.35, 2.02 | 0.39 | 0.702 | 0.23 | 0.820 |

BPF: Brain Parenchymal Fraction, EDSS: Expanded Disability Status Scale, GMF: Grey Matter Fraction, GMV: Grey Matter Volume, 9HPT: 9-Hole Peg Test, MSFC: Multiple Sclerosis Functional Composite, MTR: Magnetic Transfer Ratio, NAWM: Normal-appearing White Matter, T25FW: Timed 25-Foot Walk, T1LV: T1 Lesion Volume, T2LV: T2 Lesion Volume, WM: White Matter, WMF: White Matter Fraction, WMV: White Matter Volume.
