# Supplementary material for: Neuroimaging markers and disability scales in multiple sclerosis: A systematic review and meta-analysis
Source: PLoS One. 2024 Dec 5;19(12):e0312421. doi: 10.1371/journal.pone.0312421 (PMC11620670; doi:10.1371/journal.pone.0312421)
Supplement: S7 File — (DOCX) [file pone.0312421.s008.docx]

Supplementary 7. Funnel plots of disability and MRI measurements in pwMS.


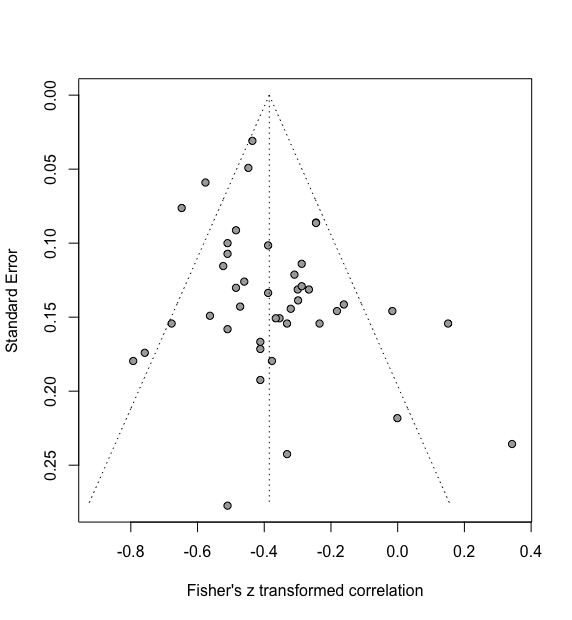


Figure S1. Funnel plot of EDSS and BPF correlation in pwMS.


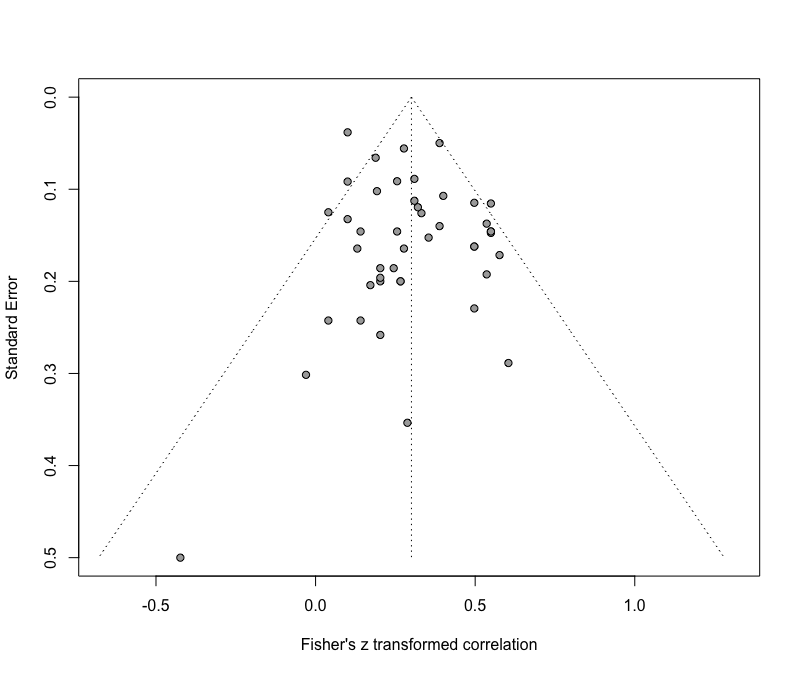


Figure S2. Funnel plot of EDSS and brain lesion volume correlation in pwMS.


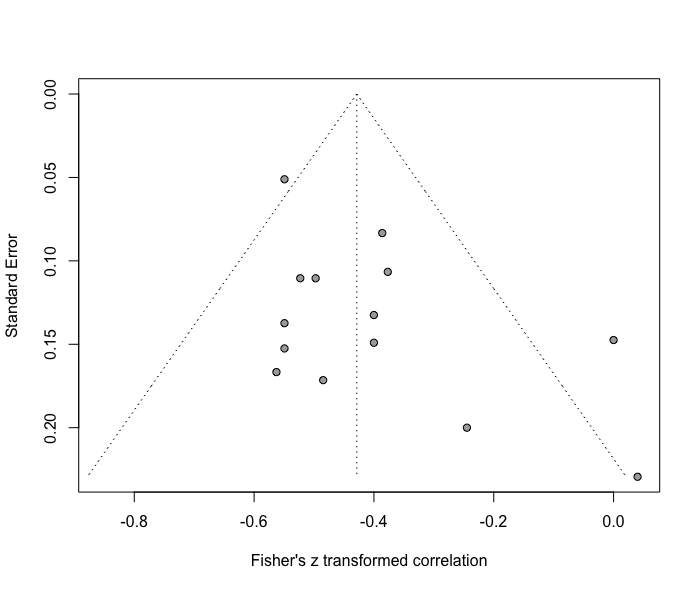


Figure S3. Funnel plot of EDSS and brain volume correlation in pwMS.


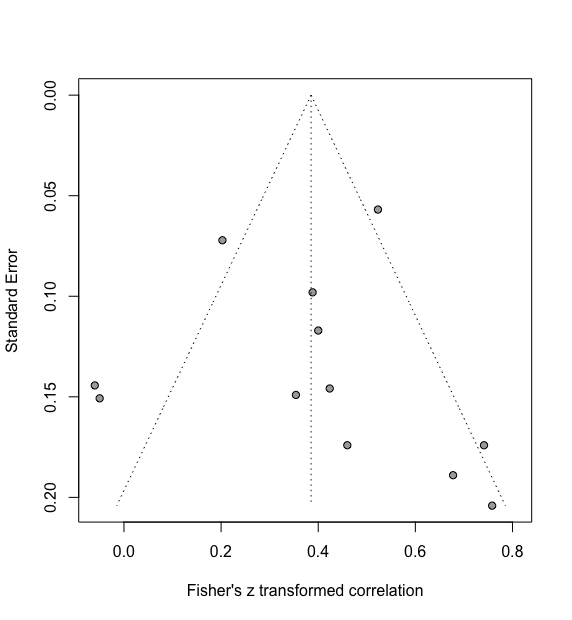


Figure S4. Funnel plot of EDSS and cortical lesion count correlation in pwMS.


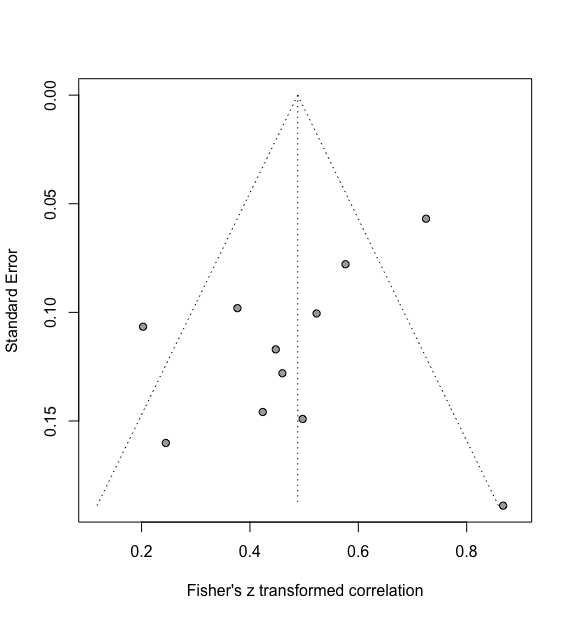


Figure S5. Funnel plot of EDSS and cortical lesion volume correlation in pwMS.


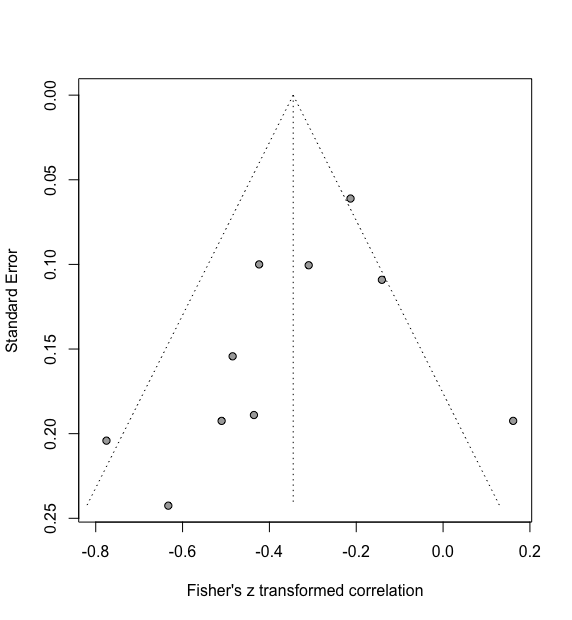


Figure S6. Funnel plot of EDSS and cortical thickness correlation in pwMS.


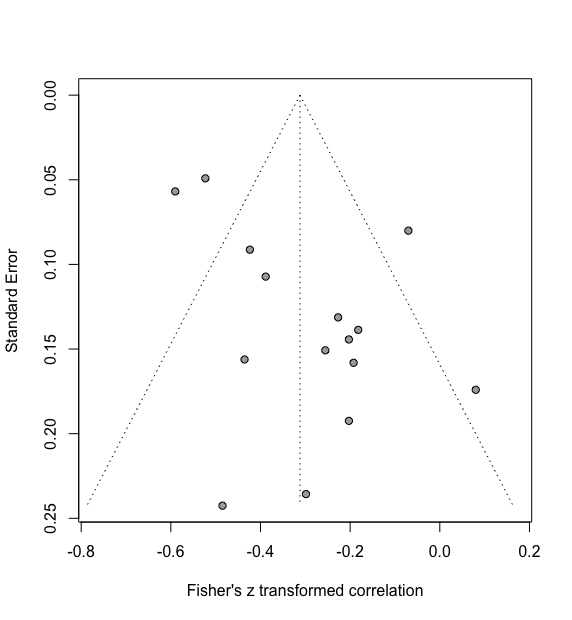


Figure S7. Funnel plot of EDSS and grey matter fraction correlation in pwMS.


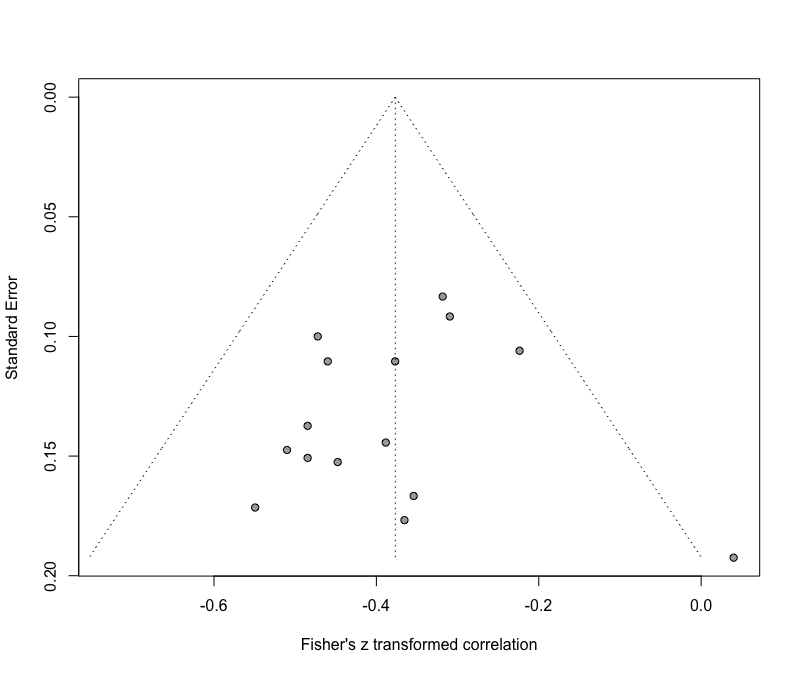


Figure S8. Funnel plot of EDSS and grey matter volume correlation in pwMS.


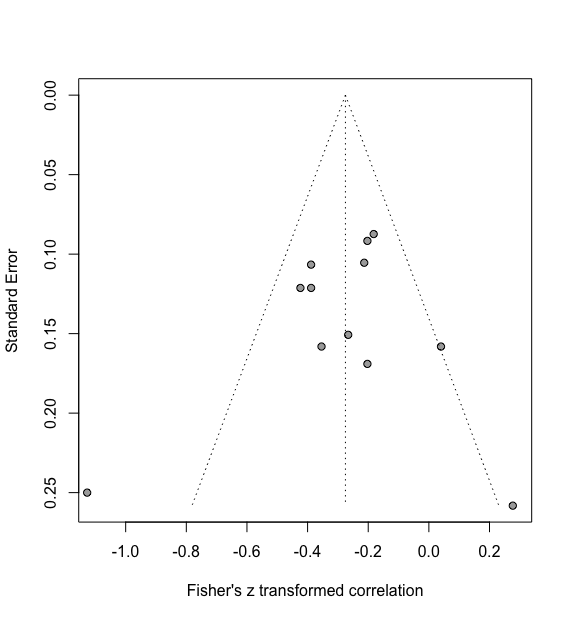


Figure S9. Funnel plot of EDSS and normal-appearing white matter MTR correlation in pwMS.


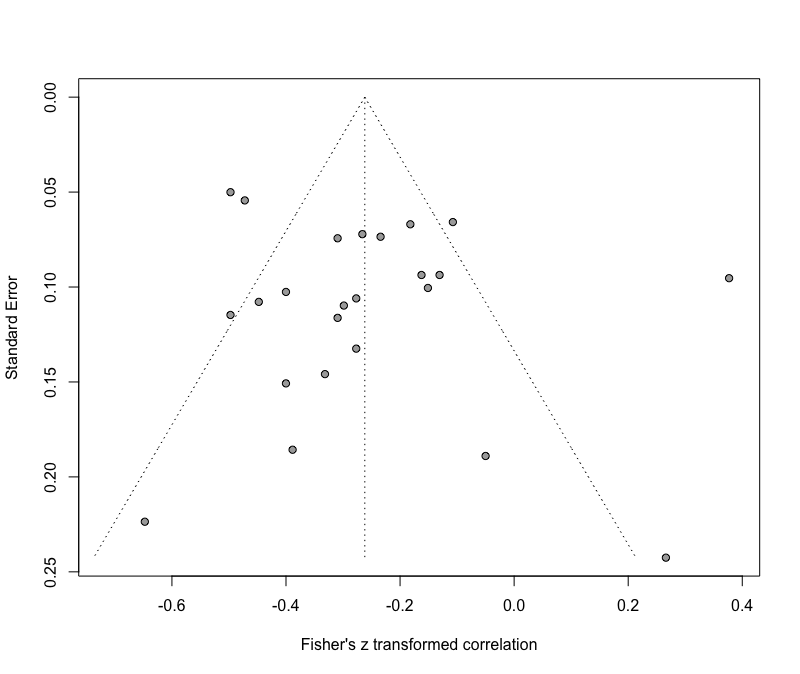


Figure S10. Funnel plot of EDSS and normalized brain volume correlation in pwMS.


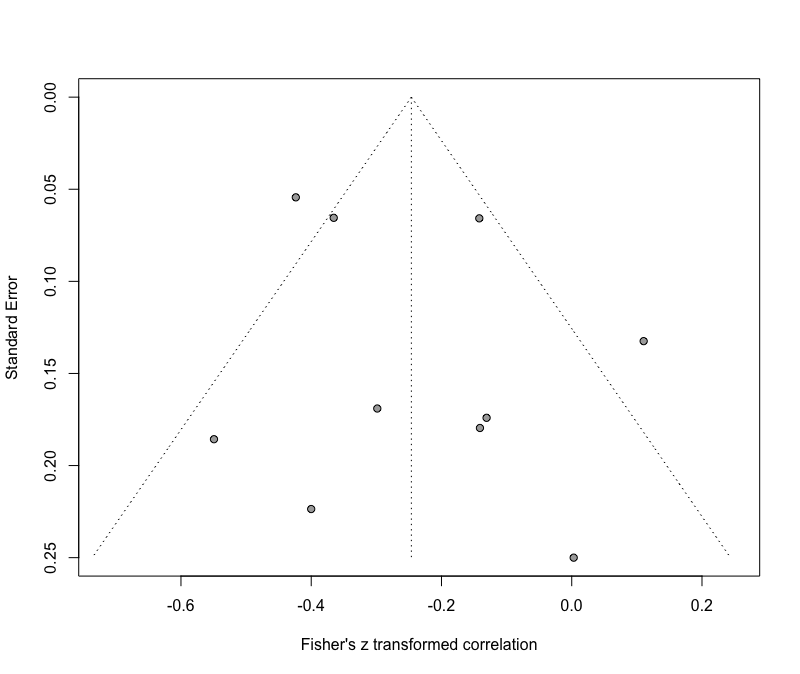


Figure S11. Funnel plot of EDSS and normalized cortical gray matter volume correlation in pwMS.


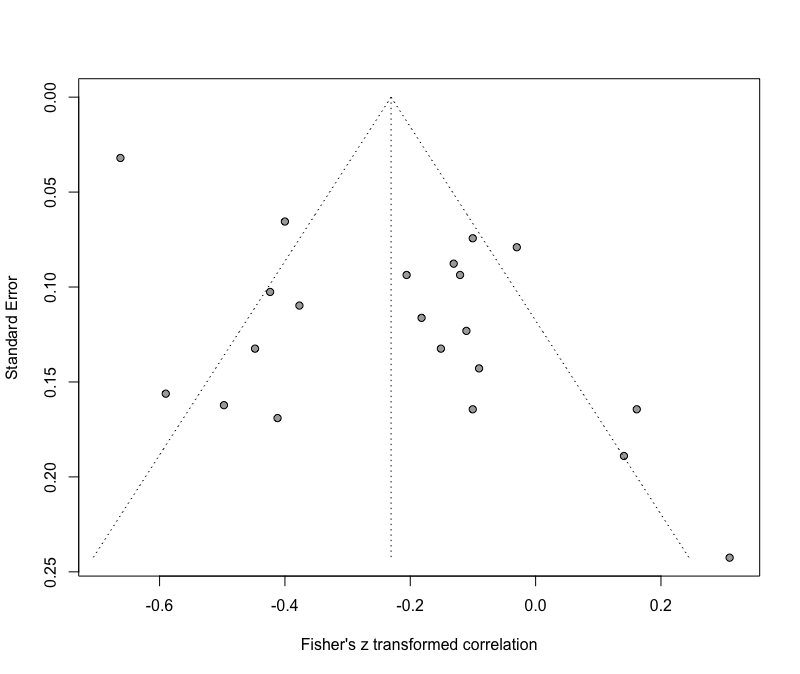


Figure S12. Funnel plot of EDSS and normalized grey matter volume correlation in pwMS.


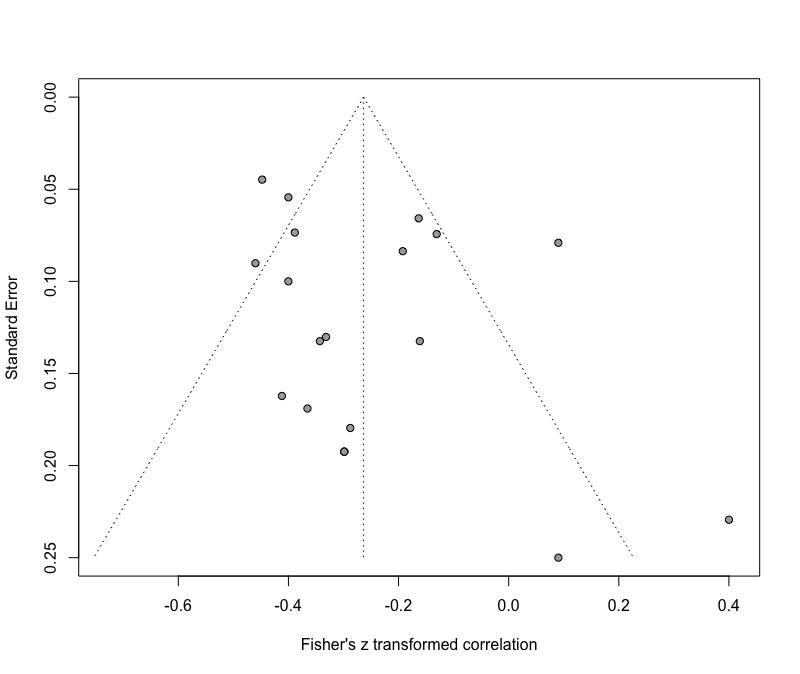


Figure S13. Funnel plot of EDSS and normalized thalamus volume correlation in pwMS.


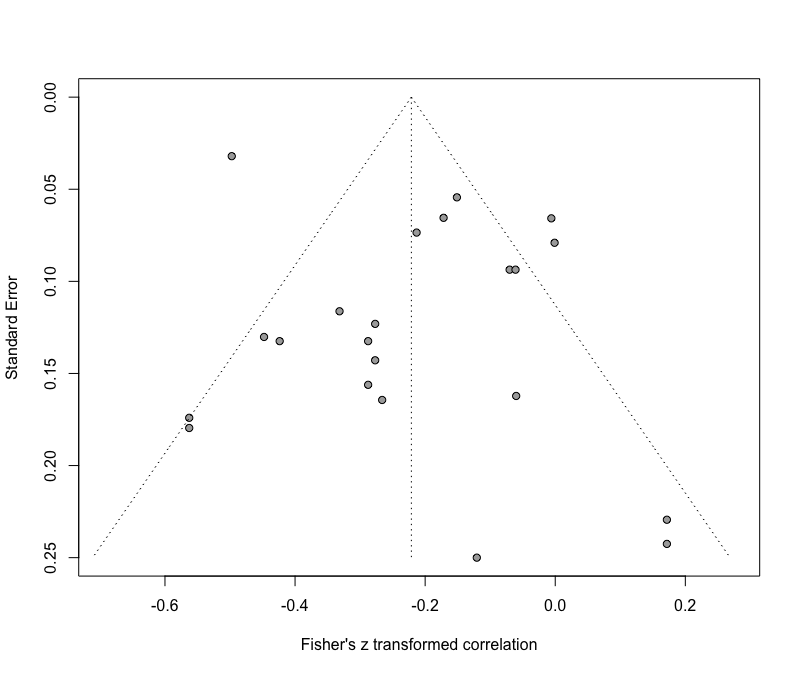


Figure S14. Funnel plot of EDSS and normalized white matter volume correlation in pwMS.


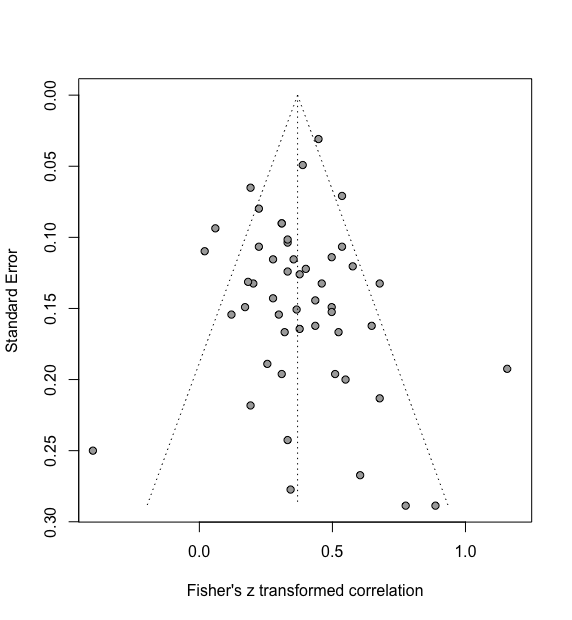


Figure S15. Funnel plot of EDSS and T1 lesion volume correlation in pwMS.


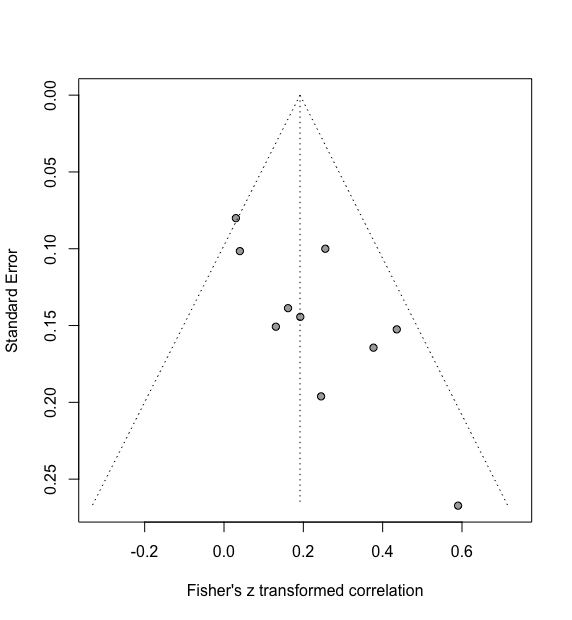


Figure S16. Funnel plot of EDSS and T1LV/T2LV correlation in pwMS.


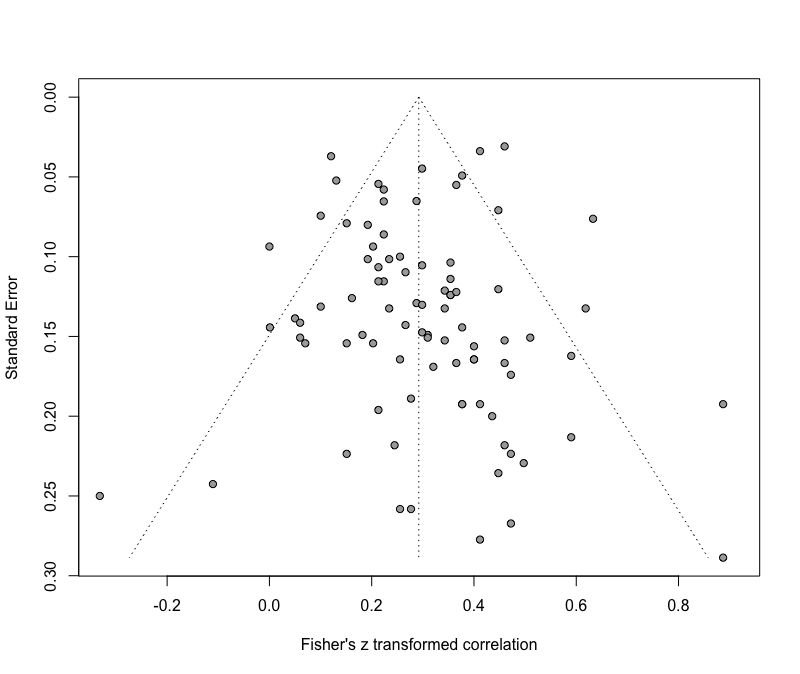


Figure S17. Funnel plot of EDSS and T2 lesion volume correlation in pwMS.


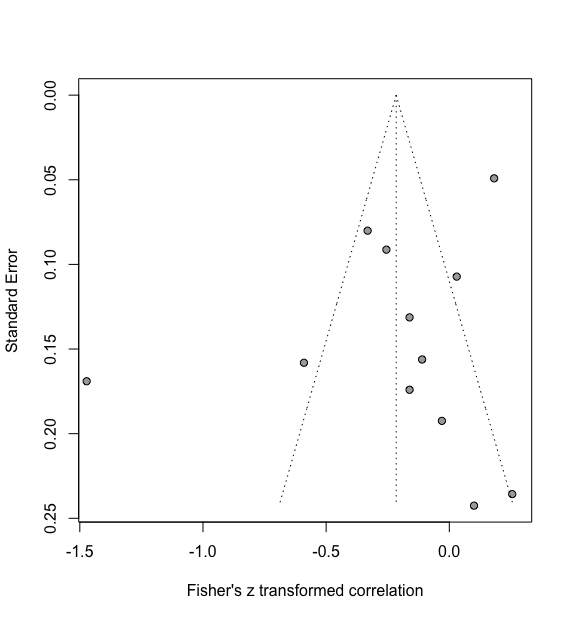


Figure S18. Funnel plot of EDSS and white matter fraction correlation in pwMS.


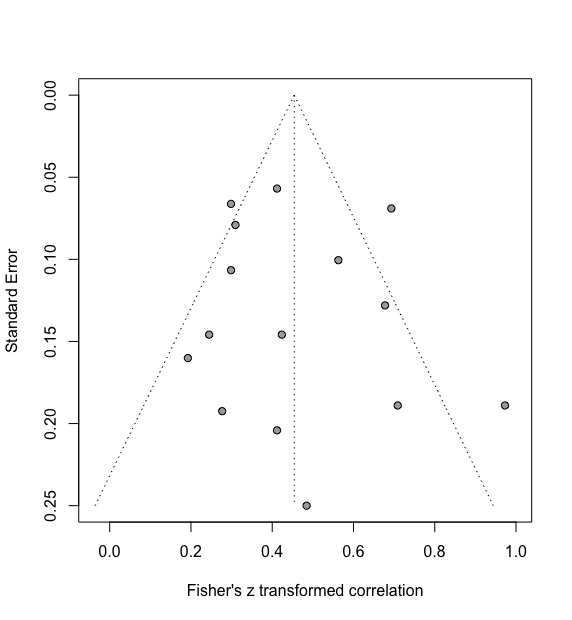


Figure S19. Funnel plot of EDSS and white matter lesion volume correlation in pwMS.


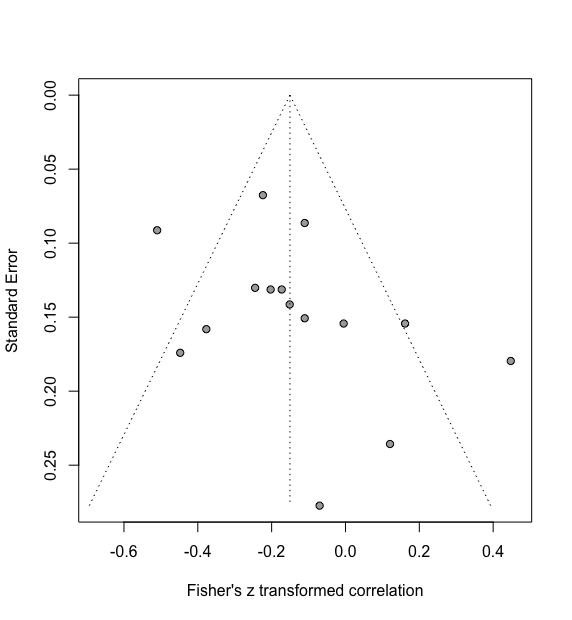


Figure S20. Funnel plot of T25FW and BPF correlation in pwMS.


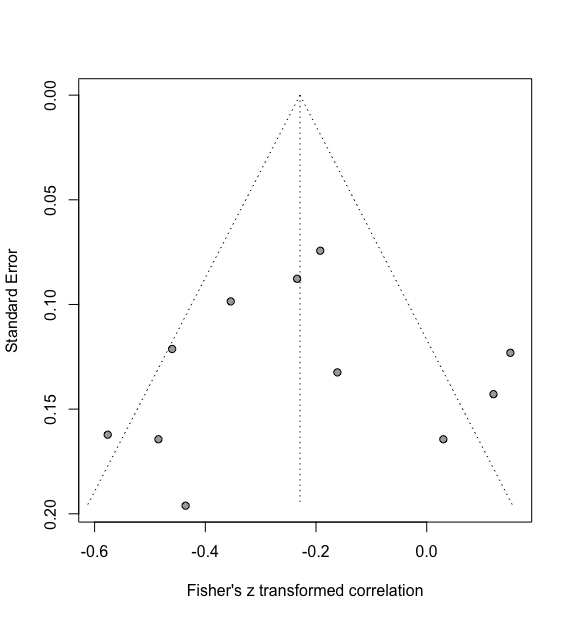


Figure S21. Funnel plot of T25FW and normalized grey matter volume correlation in pwMS.


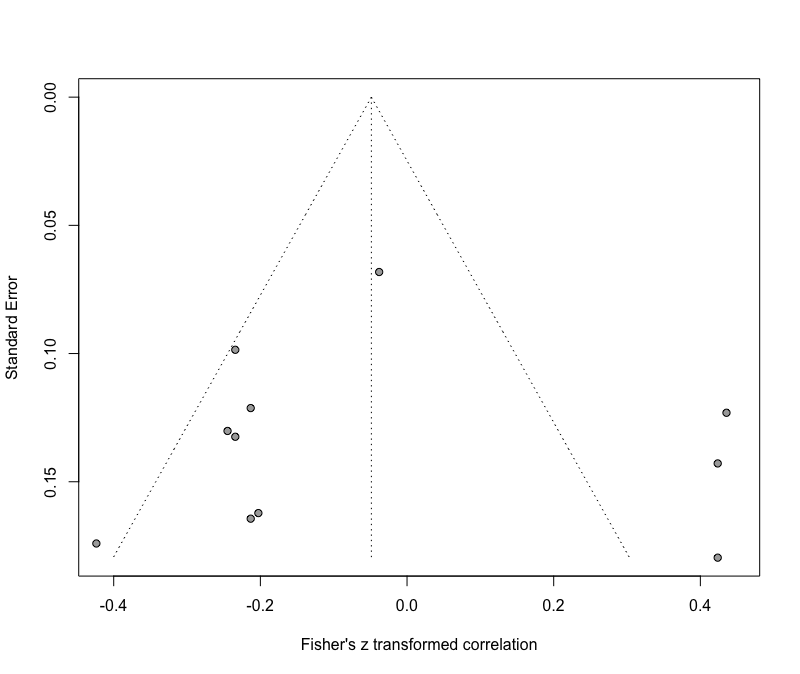


Figure S22. Funnel plot of T25FW and normalized white matter volume correlation in pwMS.


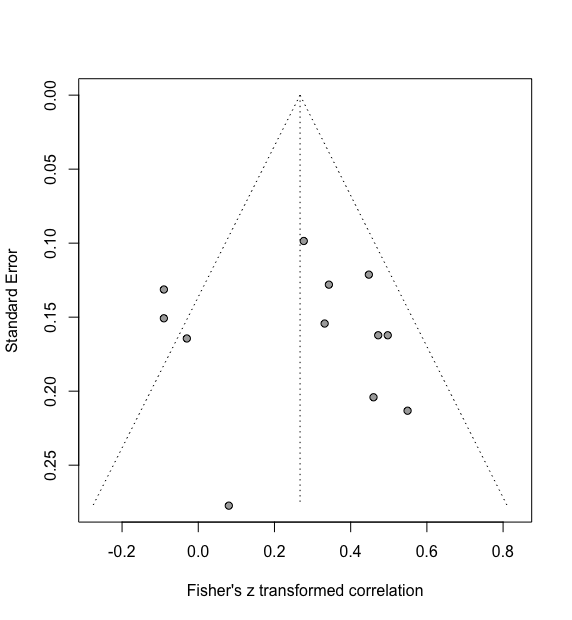


Figure S23. Funnel plot of T25FW and T1 lesion volume correlation in pwMS.


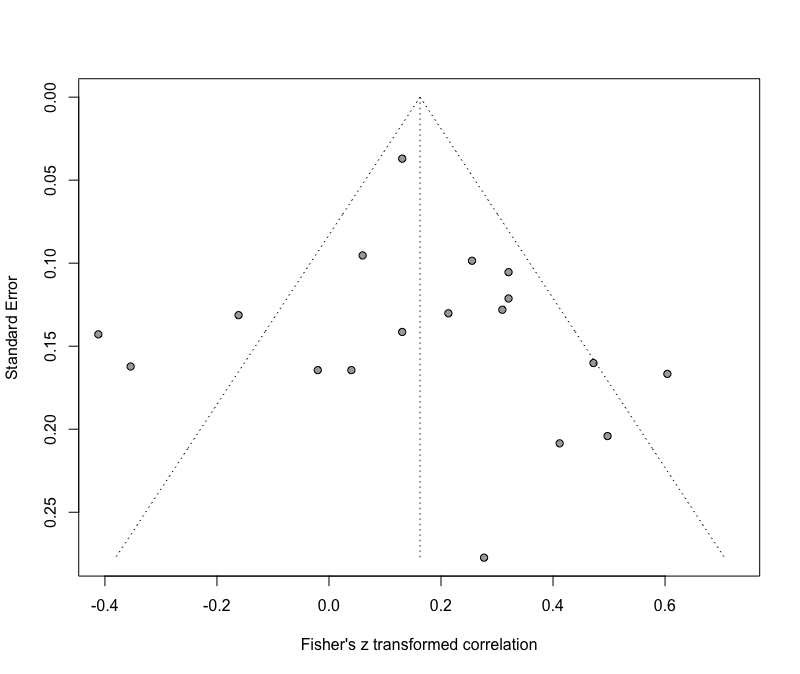


Figure S24. Funnel plot of T25FW and T2 lesion volume correlation in pwMS.


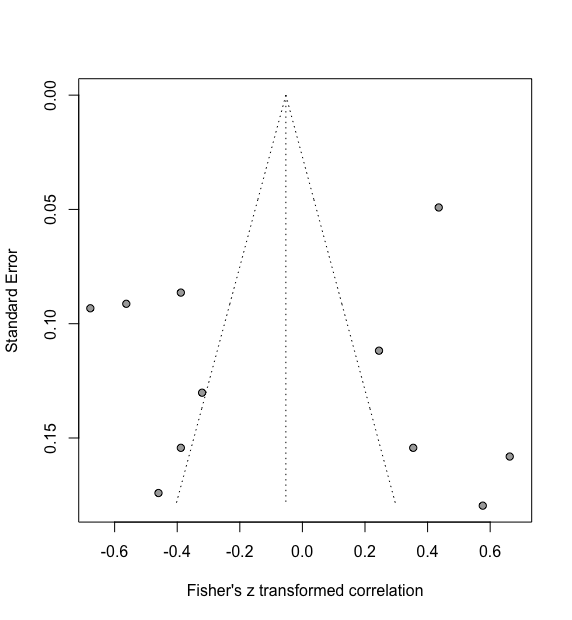


Figure S25. Funnel plot of 9HPT and BPF correlation in pwMS.


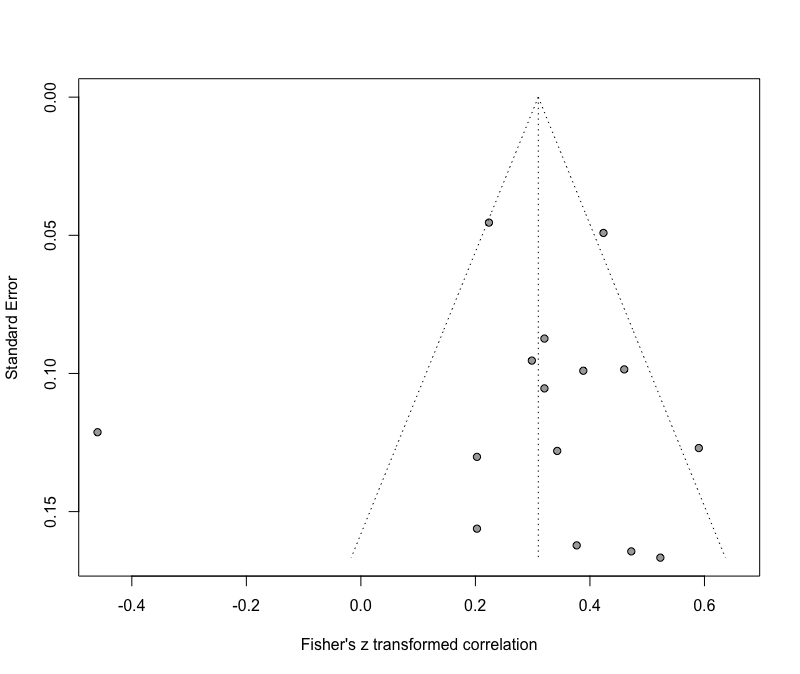


Figure S26. Funnel plot of 9HPT and T2 lesion volume correlation in pwMS.


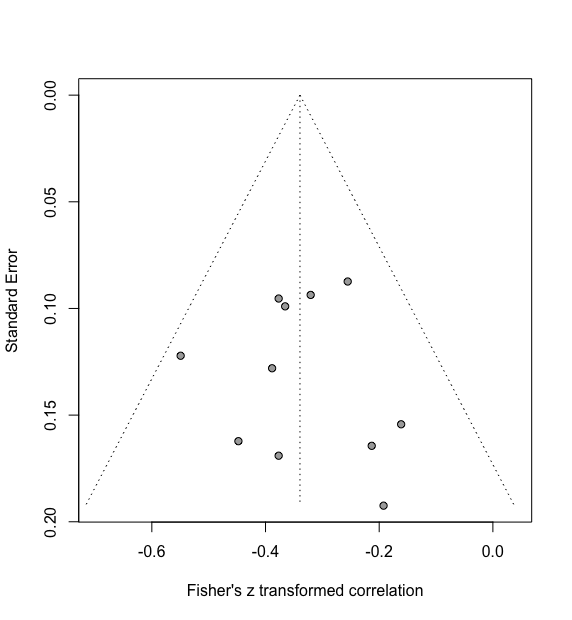


Figure S27. Funnel plot of MSFC and T2 lesion volume correlation in pwMS.
